# Triplet Vinylidenes Based on (Benz)imidazole and 1,2,3-Triazole N‑Heterocycles

**DOI:** 10.1021/jacsau.5c00491

**Published:** 2025-06-05

**Authors:** Yury Kutin, Justus Reitz, Maria Drosou, Patrick W. Antoni, Yijie He, Victor R. Selve, Sergius Boschmann, Anton Savitsky, Dimitrios A. Pantazis, Müge Kasanmascheff, Max M. Hansmann

**Affiliations:** † Department of Chemistry and Chemical Biology, 197367 Technische Universität Dortmund, Otto-Hahn-Str. 6, 44227 Dortmund, Germany; ‡ 28314 Max-Planck-Institut für Kohlenforschung, Kaiser-Wilhelm-Platz 1, 45470 Mülheim an der Ruhr, Germany; § Department of Physics, 197367 Technische Universität Dortmund, Otto-Hahn-Str. 6, 44227 Dortmund, Germany

**Keywords:** vinylidenes, reactive intermediates, EPR spectroscopy, diradicals, C−H activation

## Abstract

Triplet vinylidenes, a new class of carbon-centered diradicals containing a monosubstituted carbon atom, remain largely unexplored. A series of triplet vinylidenes based on five-membered heterocycles, featuring 2- and 4-imidazole, benzimidazole as well as 1,2,3-triazole backbones, are generated upon irradiation of stable diazoalkenes and are investigated by electron paramagnetic resonance (EPR) spectroscopy. While the calculated S/T gaps strongly vary (∼9.9–18.4 kcal/mol), the experimental zero-field splitting (ZFS) *D* values are positioned in a rather narrow and characteristic range of *D* ∼ 0.366–0.399 cm^–1^. Electron nuclear double resonance (ENDOR) studies with ^13^C-labeled samples combined with quantum chemical calculations reveal a common motif of *A*_iso_(^13^C) ≈ 50 MHz for the electronic structure of the vinylidene class. EPR decay experiments confirm that steric and electronic tuning of the heterocycle can hinder C–H activation pathways leading to the highest reported stabilities of up to 150 K. Quantum chemical studies elucidate and contrast plausible C–H insertion pathways, identifying an early triplet-to-singlet spin surface transition as the key factor that governs the stability of the vinylidenes.

## Graphical abstract

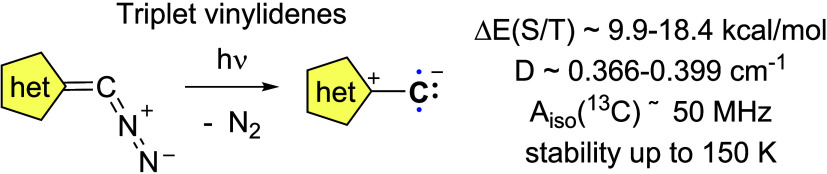

## Introduction

Vinylidenes (R_2_C=C:), also termed alkylidene carbenes, belong to the class of unsaturated carbenes, which are typically postulated as highly reactive intermediates in organic synthesis.^1^ While carbenes (R–C–R′) are well-known to feature singlet^2^ or triplet^3^ ground states depending on the R/R′-substituents,^4^ vinylidenes usually exhibit a singlet ground state. Indeed, H_2_C=C: features a ^1^A_1_ ground state, while the lowest excited triplet state (^3^B_2_) is significantly higher in energy (ca. 40 kcal/mol) followed by the ^3^A_2_ state (Scheme 1A).^5^ Note, H_2_C=C: (^1^A_1_) rearranges to acetylene on the picosecond time scale,^6^ while the metastable excited triplet state (^3^B_2_) rearranges much more slowly.^7^

**Scheme 1:**
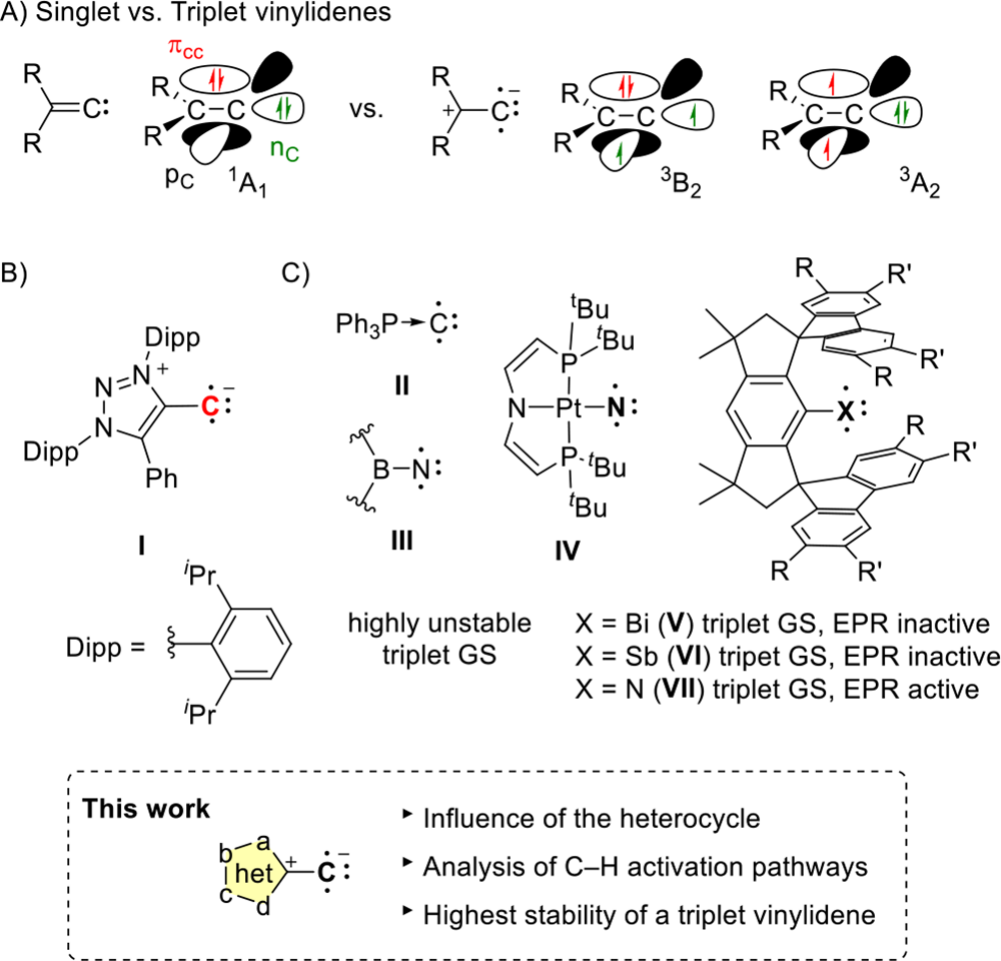
(A) Singlet and Triplet States of Vinylidenes; (B) First EPR-Characterized Triplet Vinylidene I; (C) Recently Accessed Triplet Compounds of Group 15 Elements

Due to their pronounced reactivity, singlet vinylidenes are highly challenging to detect in a direct fashion and are often postulated based on trapping experiments.^8^ Rare exceptions are the matrix isolation of F_2_C=C:,^9^ or the detection based upon ultrafast transient absorption spectroscopy.^10^ While singlet vinylidenes are rare, our groups reported in 2021 the detection of the first triplet vinylidene (**I**).^11^ Upon photochemical liberation of N_2_ from a stable diazoalkene,^12^ triplet vinylidene **I** was cleanly accessed and could be characterized by EPR/ENDOR spectroscopy at low temperatures (Scheme 1B). Due to the strong (mesoionic) polarization of the C–C bond,^13^ the ^3^A_2_ ground state was favored over the singlet state. Note that vinylidene **I** represents a carbon-centered diradical in which the terminal carbon is monosubstituted, as opposed to disubstituted carbenes. We recently, described a related monosubstituted carbon diradical which can be best described as an adduct of Ph_3_P to a C atom in its ^3^P electronic ground state (**II**; Scheme 1C).^14^ Isoelectronic to vinylidenes are boryl nitrenes **III**,^15^ which typically feature a highly reactive triplet ground state (Scheme 1C). A related electronic situation is found in other triplet pnictinidenes (R–X; X = N, P, As, Sb, Bi). Very recently, such heavy group 15 analogues have received significant attention. For instance, in 2020, Schneider, Holthausen and co-workers described a highly unstable platinum-based triplet nitrene (**IV**), formed upon the irradiation of a single crystal of the corresponding Pt-azide precursor.^16^ Due to a calculated high *D* value (73 cm^–1^), no X-band EPR could be measured. Mardyukov and co-workers recently described triplet arsinidines in matrix-isolation studies.^17^ In 2023, Cornella and co-workers,^18^ and independently Ye and Tan,^19^ described the first triplet bismuthinidene employing sterically encumbered hydrindacene ligands (Fluind-ligand) (**V**). Due to a giant ZFS, the compounds behave diamagnetically and show no EPR signal. The Fluind-ligand structure triggered a remarkable rush for the investigation of other analogs; Ye and Tan reported a triplet stibinidene (**VI**) which is also EPR-silent, however possesses a paramagnetic ground state.^20,21^ Beckmann and co-workers, as well as Ye and Tan, reported the synthesis of stable triplet nitrenes (**VII**).^22^ Aryl nitrenes typically feature a triplet ground state and are short-lived intermediates,^23^ while room-temperature-stable nitrenes and also triplet carbenes are rare.^22,24^

The recent rush on monosubstituted group 15 diradicals and the long list of triplet carbenes are in strong contrast to the single report of a triplet vinylidene,^11,25^ and the monosubstituted diradical Ph_3_PC.^14^ Importantly, while the current main-group diradicals with the Fluind-ligand structure appear very challenging to be structurally modified, the heterocyclic structure of the vinylidenes (heterocycle-C) should be highly flexible. Hence, it raises the question how many triplet vinylidenes exist and to what extent their electronic structure and stability can be tuned. Here, we report a detailed low-temperature EPR and isotope-sensitive ENDOR characterization of several new members demonstrating that triplet vinylidenes represent a new and general class of diradicals with the so far highest achieved stability up to ∼150 K.

## Results and Discussion

### Investigated Systems

Triplet vinylidenes were photochemically generated from a series of stable diazoalkenes **1** ^26^ featuring a selection of five-membered heterocycles (Scheme 2). We focused first on the systematic investigation of the 1,2,3-triazole heterocycle and synthesized three new diazoalkenes **1A** ^**iPr**^, **1A** ^**Br**^, **1A** ^**Cl**^, as well as a diazoalkene with *N*-alkyl substitution (**1B**)^27^ (for the synthesis, see SI). The new diazoalkenes feature *o*,*o*-dialkyl or *o*,*o*-dihalo substitution on the aryl system which forces the flanking aryl group to be positioned orthogonally to the triazole plane, as confirmed by an X-ray structure of **1A** ^**Br**^ (Scheme 2B). Based on the findings that triplet carbenes show increased stability with a *o*,*o*-Br and *o*,*o*-Cl substitution,^3,28^ we applied such a kinetic stabilization strategy for taming vinylidenes. Besides the 1,2,3-triazole heterocycle, we additionally investigated the literature-known diazoalkene precursors based on 4-imidazole (**1C**),^12a^ and 2-imidazole heterocycles featuring a small *N*-Me (**1D**) and bulky *N*-aryl (**1E**) fragments.^12b^ For such precursors the irradiation at room temperature has been investigated by product studies,^12^ however, no data exist on the vinylidene intermediates involved. Additionally, we investigated the influence of π-delocalization of the diradical by studying the benzimidazole diazo precursor **1F**.^29^

**Scheme 2:**
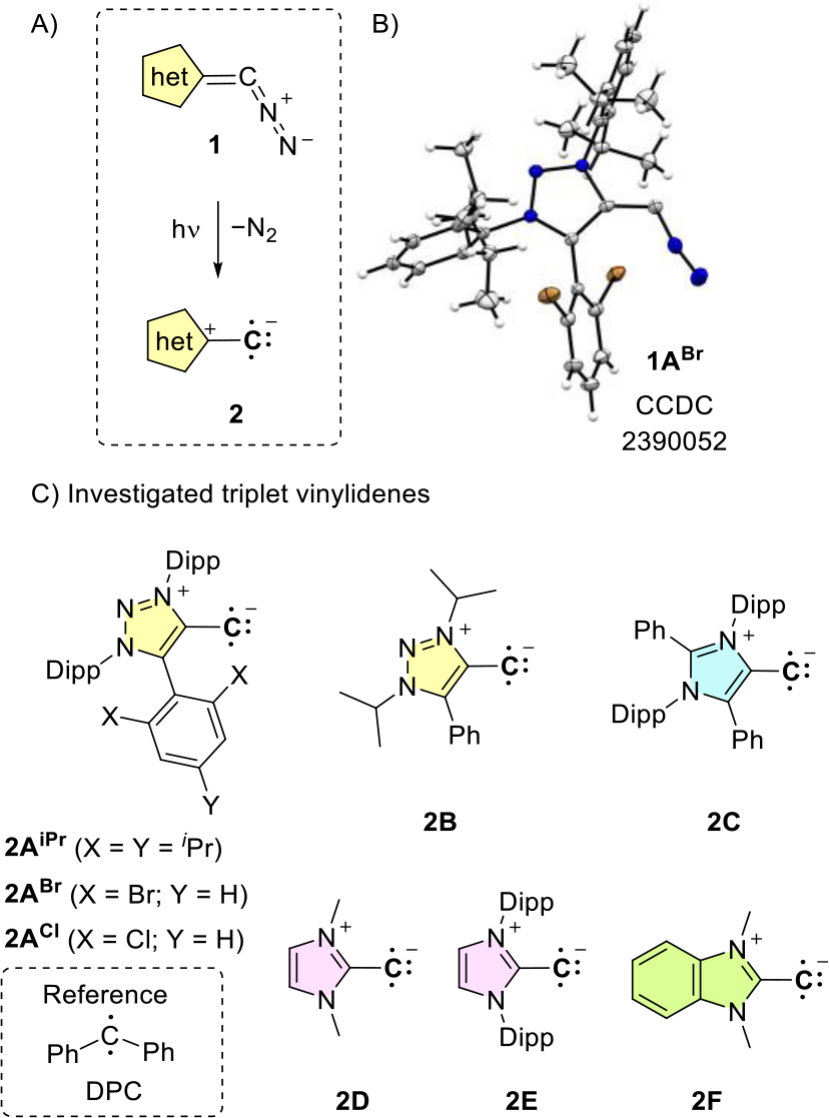
(A) Synthetic Access to Vinylidenes from Stable Diazoalkene Precursors; (B) X-ray Solid-State Structure of Diazoalkene 1A^Br^;^30^ and (C) Heterocyclic Frameworks Investigated in This Manuscript^a^

a Thermal ellipsoids are shown with 50% probability. Two toluene molecules omitted for clarity. Selected bond parameters in [Å] and [°]: C1–N6 1.276(3), C1–C2 1.400(3), N6–N5 1.152(3), C2–C3 1.407(3), N6–C1–C2 118.25(17), C1–C2–C3 134.44(17), N5–N6–C1 169.8(2). Dipp: 2,6-diisopropylphenyl.

### EPR and DFT Investigations

In order to generate vinylidenes, the diazoalkene precursors **1** (Scheme 2) were illuminated with UV light in a frozen toluene solution at 10 K. The photolysis products were then analyzed using pulsed Q-band (34 GHz) EPR spectroscopy. Pseudomodulated EPR spectra recorded at 6 K are shown with simulations in Figure 1; as-recorded FID-detected spectra are given in Figure S2.1 (see also Figure S2.2).

**Figure 1:**
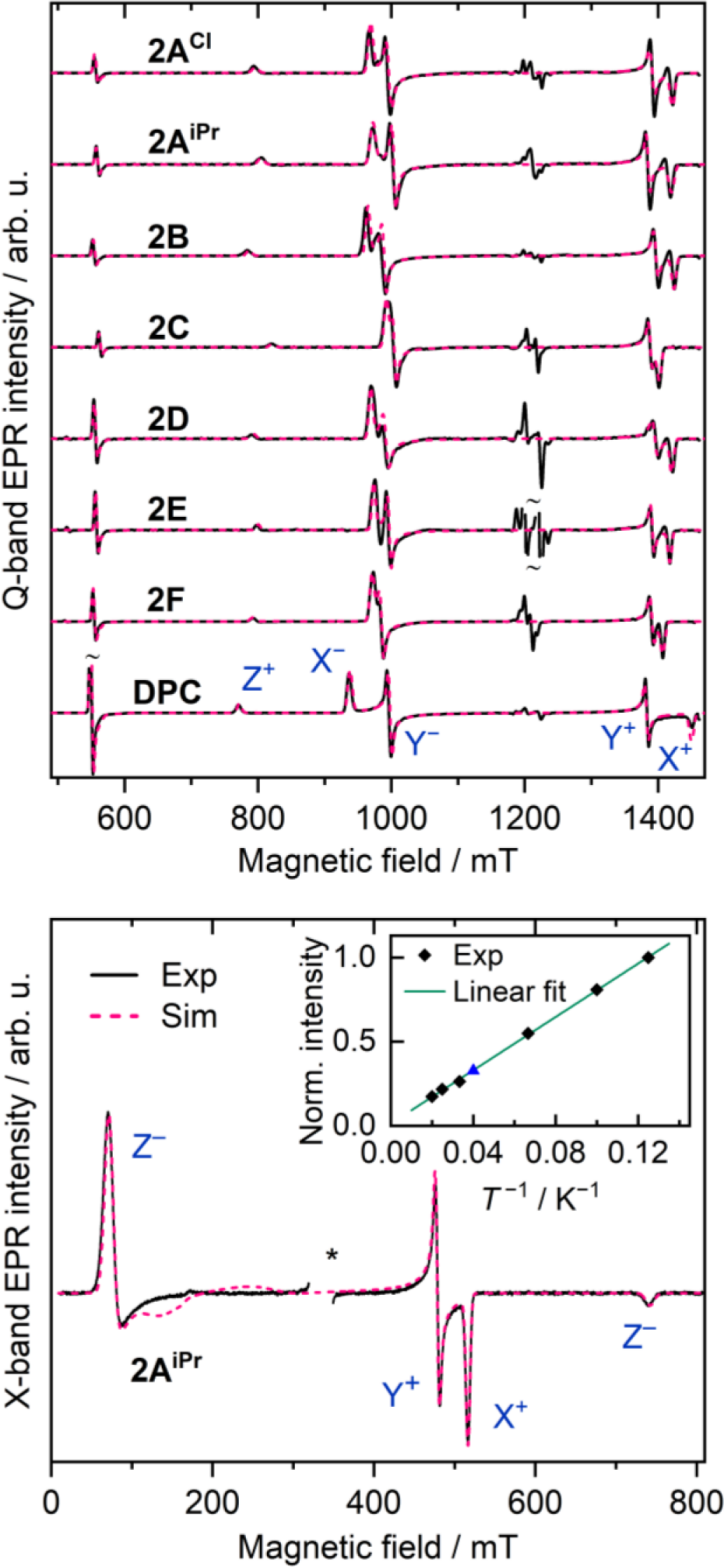
Top: pseudomodulated FID-detected Q-band EPR spectra (black solid lines) acquired at 6 K after the photolysis of the precursor series in frozen toluene solution. The spectra are overlaid with the best fits assuming an *S* = 1 species (pink dashed lines). The as-recorded pulsed spectra are shown in Figure S2.1; for **2A** ^**Br**^ see Figure S2.2. Bottom: CW X-band EPR spectrum of **2A** ^**iPr**^ (*T* = 10 K) with the best fit; the Curie–Weiss plot is shown in inset (black diamonds: temperature increased from 8 and 50 K; blue triangle: temperature decreased from 50 to 25 K); asterisk: a radical signal is omitted for clarity. Energy level diagrams with the corresponding transitions are shown in Figure S2.3.

The characteristic triplet-state spectra indicated the formation of triplet vinylidene species from all the precursors discussed here. The well-studied triplet diphenylcarbene (**DPC**),^31^ additionally generated from the corresponding diazoalkane precursor, is shown for comparison. To determine the ZFS parameters, we simulated all EPR spectra assuming an isotropic *g*-factor *g* = 2.0023 (Figures 1, S2.1, S2.2, and, Table 1). Spin-energy levels with X- and Q-band EPR transitions are shown in Figure S2.3. Our simulations revealed that the ZFS parameter *D* maintains a relatively consistent value within the vinylidene series, fluctuating only between +0.366 and +0.399 cm^–1^ (**2C** and **2B**, respectively). These values also fall within the typical range for triplet diphenylcarbenes (0.346–0.409 cm^–1^),^4^ well below the typical nitrene values at approximately 1 cm^–1^ for instance for phenylnitrene.^32^ Notably, the experimentally obtained vinylidene *D* values closely match those calculated with ORCA^33^ using the TPSSh functional,^34^ showing an agreement within 6%. For further details on EPR spectral artifacts and the determination of the sign of *D*, see ref 11.

**Table 1:** ZFS and ^13^C hf Parameters Providing the Best Global Fit to the Q-Band EPR and ENDOR Spectra of the Vinylidene Series and the DPC Carbene,^a^ Compared with DFT-Computed Vertical Singlet–Triplet Gaps and EPR Parameters^b^

compound		S/T gap (kcal mol^–1^)	*D* (cm^–1^)	|*E*|/*D*	*A*_*X*_ (MHz)	*A*_*Y*_ (MHz)	*A*_*Z*_ (MHz)	*A*_iso_ (MHz)
**I** ^11^	exp.		0.377	0.028	57.1	100.0	–7.2	50.0
	DFT	9.9	0.375	0.041	53.7	96.7	–10.8	46.5
**2A** ^**iPr**^	exp.		0.382	0.027	59.2	100.5	–6.7	51.0
	DFT	13.2	0.404	0.038	55.3	95.1	–11.5	46.3
**2A** ^**Br**^	exp.		0.384	0.021				
	DFT	12.5	0.381	0.028	55.3	94.7	–12.1	46.0
**2A** ^**Cl**^	exp.		0.390	0.023				
	DFT	12.4	0.361	0.111	54.9	94.6	–12.2	45.8
**2B**	exp.		0.399	0.020				
	DFT	14.1	0.385	0.013	56.1	94.3	–10.9	46.5
**2C**	exp.		0.366	0.011				
	DFT	11.5	0.351	0.036	50.8	96.3	–10.3	45.6
**2D**	exp.		0.393	0.019				
	DFT	18.4	0.394	0.018	55.4	92.8	–12.9	45.1
**2E**	exp.		0.386	0.020	58.5	100.3	–7.4	50.5
	DFT	13.9	0.410	0.031	53.4	95.1	–12.7	45.2
**2F**	exp.		0.387	0.012				
	DFT	12.1	0.381	0.013	51.6	94.2	–13.3	44.2
**DPC**	exp.		0.412	0.048	196	216.5	119.5	177.3
	DFT	12.5	0.353	0.030	180.1	202.3	104.2	162.2

a The uncertainties in the simulated values are ±0.5 MHz for the hf tensor components, ±0.003 cm^–1^ for *D*, and ±0.003 for |*E*|/*D*. The simulations were performed assuming *g*_iso_ = 2.0023; the ZFS and ^13^C hf tensors were assumed collinear.

b Vertical singlet–triplet gaps were computed with the mPW2PLYP^35^ functional, EPR parameters with TPSSh (see the SI for extensive computational details).

To confirm the triplet ground state, we conducted temperature-dependent X-band measurements for **2A** ^**iPr**^, which is representative of the 1,2,3-triazolium systems, over a range of 8 to 50 K (Figure 1, bottom). A Curie–Weiss plot of the EPR intensity vs the reciprocal of temperature (see inset) shows a linear dependence, in agreement with a well-isolated triplet ground state. This result confirms that the five-membered-ring vinylidenes containing a 1,2,3-triazolium substituent are ground-state triplets. For the imidazolyl-based vinylidenes, a triplet ground state could be inferred from the EPR stability measurements (see below).

DFT calculations support the assignment of spin triplet ground states for all vinylidenes. Analysis of frontier orbitals and spin densities (Figures 2 and S3.1) reveals that the ground states share the same essential features in all systems examined in the present study. Even the spin populations remain relatively constant across the different heterocycles. The unpaired electrons occupy a π-type orbital that is delocalized across the ring, as well as an in-plane p-type orbital localized on the terminal carbon, resulting in a net spin population of approximately 1.5 electrons on the terminal carbon in all cases. The triplet states are predicted to be well-separated from excited singlet states with the vertical energy differences (at the mPW2PLYP/def2-TZVP level) listed in Table 1. These closed-shell singlet states computed by DFT correspond to double occupation of the delocalized π-type orbital, while the in-plane p-type orbital becomes the LUMO. As will be discussed below, adiabatic S/T gaps cannot be reported for some of the compounds, because some of the vinylidenes do not possess a distinct singlet-state minimum at the geometries discussed here, but rather undergo spontaneous C–H activation. For this reason, we focus here on the vertical S/T gaps. Within the 1,2,3-triazolium substituent subgroup, the vertical S/T gap calculated with the double-hybrid mPW2PLYP functional is between 9.9 and 14.1 kcal/mol (**I**, **2A–2B**), 11.5 kcal/mol for the 4-imidazolium system (**2C**), 18.4 and 13.9 kcal/mol for the 2-imidazolium systems (**2D** and **2E**), and 12.1 kcal/mol for the benzimidazole system (**2F**). Clearly, the S/T gaps vary more strongly than the experimentally obtained ZFS parameters.

**Figure 2:**
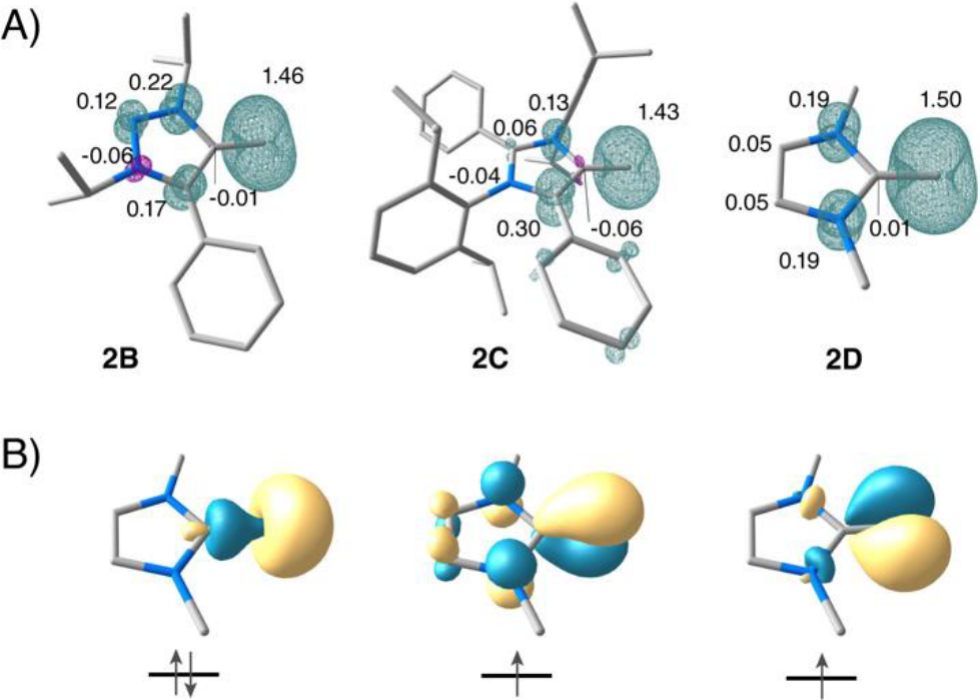
(A) Spin density distributions and Mulliken spin populations (from TPSSh calculations) for models **2B**, **2C**, and **2D** representing three heterocycle classes; (B) quasi-restricted orbitals describing the valence electronic structure of the triplet ground states exemplified for **2D**.

It should be taken into account that DFT estimations of S/T gaps are sensitive to the choice of functional (see Table S3.1), albeit without an obvious correlation with a particular component such as the percentage of exact exchange. The mPW2PLYP functional used here as reference, along with the more affordable BLYP^36,37^ functional, which shows good agreement with mPW2PLYP, were top performers in a recent benchmark study on S/T gaps in aryl carbenes.^38,39^ We note that DFT calculations cannot address the presence and relative energy of “open-shell” singlet states, or of the alternative closed-shell singlet state that would result from occupation of the in-plane C-centered p-type orbital. To gain insight into the magnitude of energy differences between the possible singlet states, we used the three parent unsubstituted heterocyclic systems, to conduct complete active space self-consistent field (CASSCF) calculations. The active space was composed of 10 electrons in 8 orbitals. Orbital averaging was performed among the triplet ground state and the three relevant singlet states, with final energies corrected for dynamic correlation by the N-electron valence state perturbation theory (NEVPT2) (Figure S3.3 and Table S3.2). The results confirm a good separation of the ground-state triplets (>10 kcal/mol) in all cases and indicate that the closed-shell singlet with occupation of the in-plane orbital is considerably disfavored, while the open-shell singlet state is slightly higher in energy than the lowest closed-shell singlet in the case of the 1,2,3-triazolium, lower in the case of the 4-imidazolium, and slightly higher in the case of the 2-imidazolium, within 1.5 kcal/mol at the NEVPT2 level.

### ^13^C ENDOR

Recently, we demonstrated that the vinylidene **I** possesses a significantly lower ^13^C isotropic hyperfine (hf) than triplet carbenes, highlighting the differences in the orbital configuration between the two types of carbon-centered diradicals as predicted by theory.^11^ Here, we investigated whether the low *A*_iso_(^13^C) ≈ 50 MHz is representative of the entire class of triplet vinylidenes based on five-membered heterocycles, and to what extent it can be varied by the ring and/or the substituent groups. Thus, we labeled the terminal carbon of vinylidenes **2A** ^**iPr**^ and **2E** with ^13^C (nuclear spin *I* = 1/2; for the synthesis see SI) and performed ENDOR experiments to determine the ^13^C hf tensors. For comparison with a typical triplet carbene, the same procedure was also performed on **DPC**.^34^

As with **I**, no additional splitting was observed in EPR spectra upon ^13^C labeling of vinylidenes, suggesting a relatively weak ^13^C hf coupling (Figure S2.4). In contrast, for **DPC**, the hf interaction was large enough for the splitting to be observed in a field-swept spectrum (Figure S2.4). Orientation-selective ^13^C ENDOR spectra were isolated by subtracting the ^1^H and ^14^N ENDOR features obtained for natural-abundance samples (see Figures S2.5–S2.7). The resulting ^13^C ENDOR signals at five canonical field positions are shown in Figure 3 with the corresponding simulations. The experimentally fitted ^13^C hf tensors agree very well with the computational TPSSh results (Table 1).

**Figure 3:**
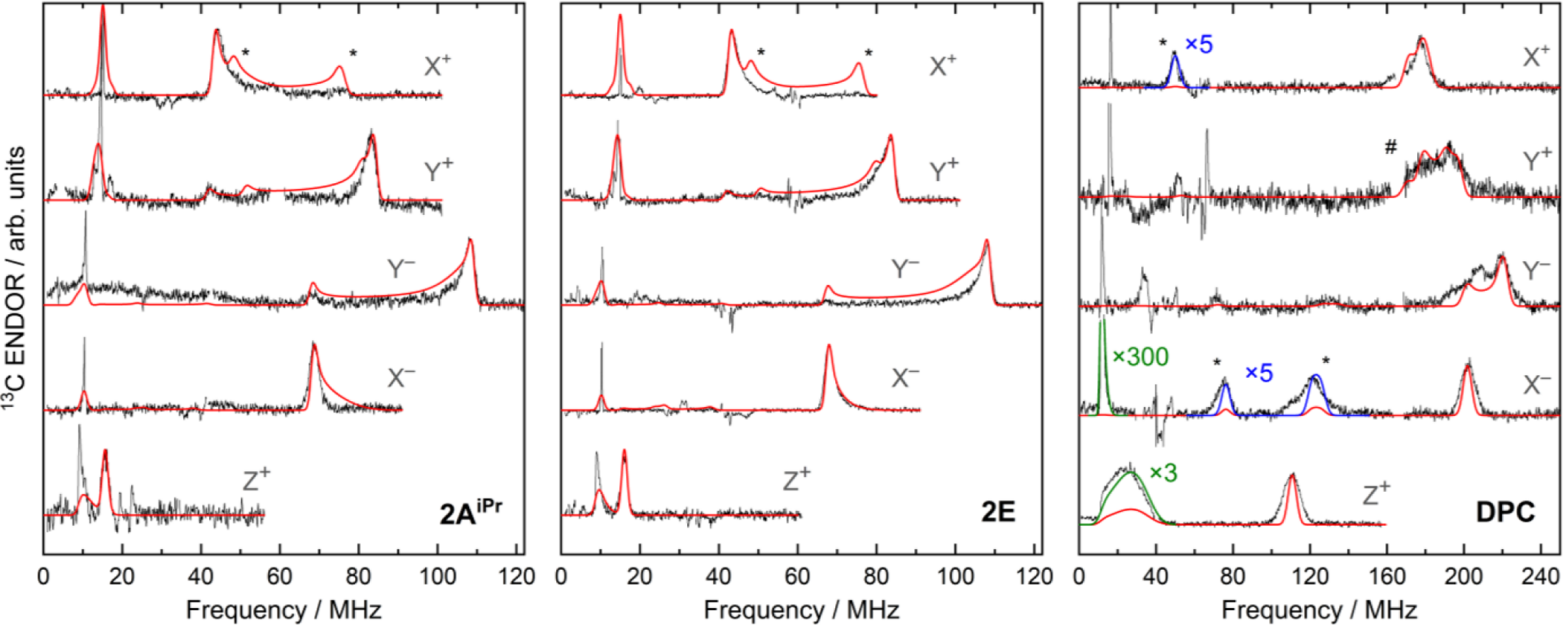
Background-corrected ^13^C Davies ENDOR spectra of **2A** ^**iPr**^ (left), **2E** (middle) and **DPC** (right) acquired at 6K at the canonical field positions (black) overlaid with simulations (red). Note the different frequency scales for the vinylidenes and carbene. Asterisks mark simulated ridges originating from noncanonical orientations (suppressed for the vinylidenes, see Figure S2.8); # marks a field-independent artifact removed from each trace for clarity.

^13^C ENDOR signals of **2A** ^**iPr**^ and **2E** closely resemble those of **I**,^11^ as evidenced by the nearly identical *A*_iso_(^13^C) values of about 50 MHz (see Table 1). These findings suggest that the electronic structure remains consistent across different structural variants within the vinylidene series. In contrast, *A*_iso_ for the **DPC** was found to be 3.5 times higher, in agreement with previously reported values.^40^ Consequently, we conclude that such a low *A*_iso_ is a characteristic parameter for the class of triplet vinylidenes. Two-dimensional simulated ^13^C ENDOR patterns obtained across the entire EPR absorption envelope for a vinylidene and carbene highlight the spectroscopic difference between the two classes (see Figure S2.8).

We note that the vinylidene electronic structure could be further experimentally constrained by ^14^N ENDOR. Specifically, analysis of the ENDOR features from ring nitrogens in **2A** ^**iPr**^ and **2E** provided information on the spin density delocalization into their respective heterocycles (see SI 2.6). Experimentally fitted ^14^N hf and quadrupole interaction (QI) tensors (Figure S2.9) closely match the computational predictions (Table S2.1). In **2E** a subtle effect of the C–C–N_2_ bond angle (prior to the elimination of dinitrogen) on the electron spin density distribution was detected (SI 2.6).

### Stability Measurements

Diradicals have found a series of interesting applications due to their magnetic and spin-related properties;^41^ however, low stability can limit applications of the vinylidenes. All EPR/ENDOR measurements discussed so far were carried out at relatively low temperatures where no decay of the triplet vinylidenes was observed. To evaluate stabilities of the different vinylidenes, we generated the triplet species at 94 K and subsequently monitored their EPR intensities at increasing temperatures (Figures 4, top and S2.12). The signal intensity, *I*, was monitored via the X^+^ feature at X-band (see Figure S2.11) and subsequently multiplied by the corresponding sample temperature, *T*, to account for Curie dependence. Temperature range, within which a triplet species is stable, corresponds to a constant value of the (*I* × *T*) product. Differences in vinylidene stability were further visualized by comparing EPR intensities measured at 94 K immediately after UV illumination and following a 30 min anneal at 116 K (Figure 4, bottom).

**Figure 4:**
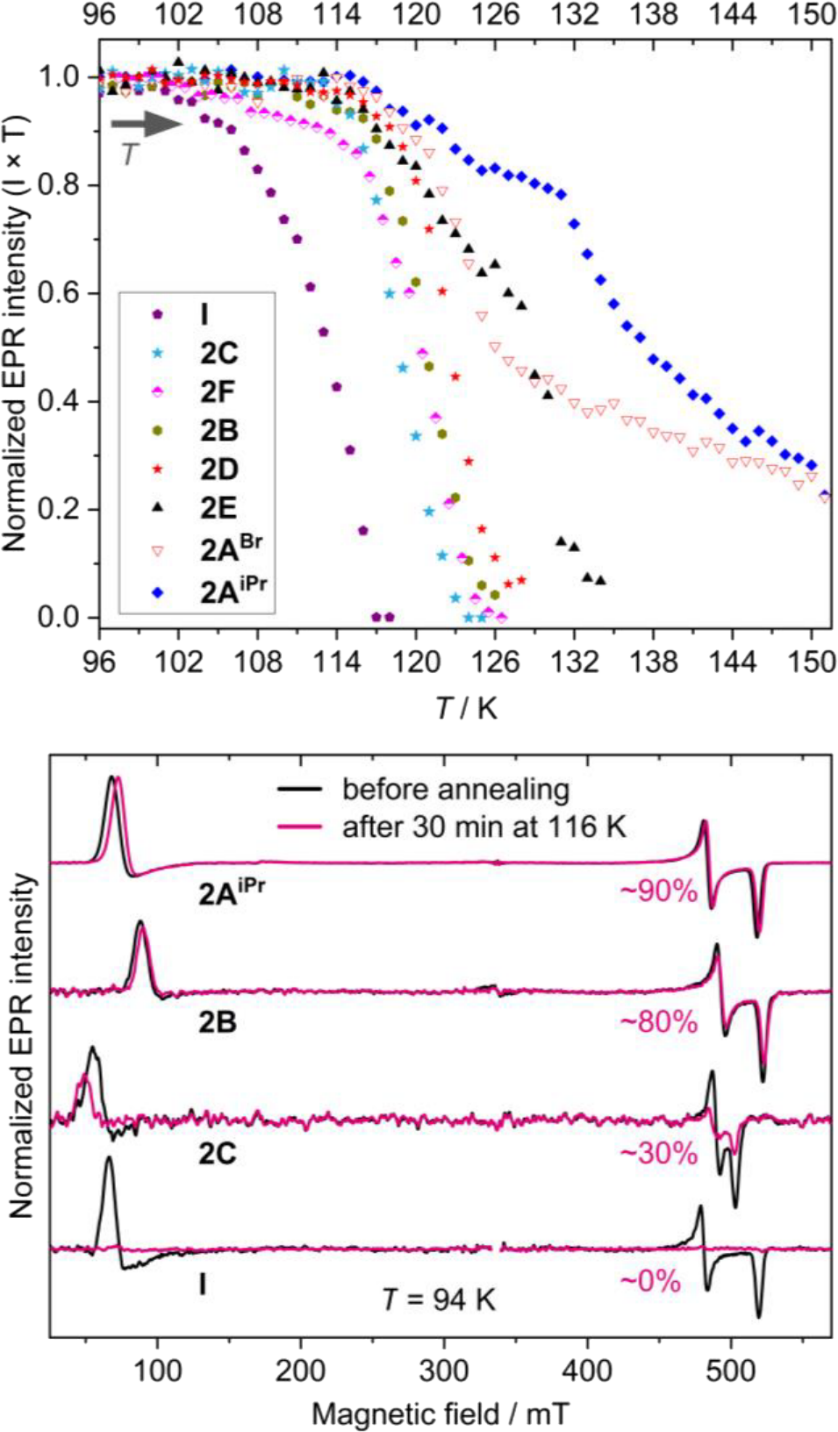
Top: normalized EPR intensity of triplet vinylidenes measured at increasing temperatures following UV illumination at 94 K (approximately 3 min per temperature point). **2A** ^**Cl**^ is omitted for clarity as its behavior is similar to that of **2A** ^**Br**^ (Figure S2.12). Bottom: background-corrected CW X-band EPR spectra of chosen vinylidene species collected at *T* = 94 K in toluene. Black traces: immediately upon irradiation with UV light (94 K); purple traces: following a 30 min anneal at 116 K. Percentage of the retained EPR intensity is shown for each trace. The sample of **I** was annealed at 110 K, leading to a full decay.

**2A** ^**iPr**^, **2A** ^**Br**^, and **2A** ^**Cl**^ exhibited minimal to no decay (within the sensitivity limits of the spectrometer) up to *T* ≈ 115 K, whereas the EPR signal of the previously described **I** decayed completely within minutes around that temperature. Because of a nearly identical behavior of the two halogenated systems, **2A** ^**Cl**^ and **2A** ^**Br**^, only the latter is shown in Figure 4 for clarity (see Figure S2.12 for a comparison). Systems **2B** and **2C**, which, like species **I**, also possess a phenyl group close to the monosubstituted carbon, showed a noticeable improvement in stability compared to **I**. While the onset of their decay was at ∼115 K, the decay kinetics of **2B** and **2C** were faster than those of **2A** ^**iPr**^/**2A** ^**Br**^/**2A** ^**Cl**^. Among the two 2-imidazole systems, **2D** decayed at marginally higher temperatures than **2B** and **2C**, whereas **2E** showed a slower decay trend between 115 and 128 K, with an abrupt increase in the decay rates at 129 K and higher. For the benzimidazole system **2F**, bearing strong resemblance to **2D**, two distinct decay phases were clearly observed: (i) a slow decay between ∼104 and 116 K and (ii) a steep decay above 116 K.

The decay of the three most stable species, **2A** ^**Cl**^/**2A** ^**Br**^/**2A** ^**iPr**^, can be divided into two to three phases: from 115 to 130 K and from 130 K onward, with an additional change in the decay rate observed uniquely for **2A** ^**iPr**^ at ∼120 K. This suggests that different decay pathways may dominate the different temperature ranges, potentially unlocking one after another as the temperature increases. Alternatively, the observed decay phases may originate from distinct conformations of the vinylidene molecules in a frozen toluene glass. Interestingly, slower decay kinetics are observed at higher temperatures, which can also be explained by conformational inequivalence. Despite the differences in the decay kinetics of **2A** ^**iPr**^ and **2A** ^**Cl**^/**2A** ^**Br**^, all three of them retain ∼20% of their original signal intensity at temperatures as high as 155 K.

Generally, various mechanisms can be responsible for the decay of triplet species, such as H-atom abstraction from solvent molecules, which is commonly observed in triplet carbenes.^42^ To test if it contributes to the vinylidene decay, we repeated the thermal decay measurements in toluene-d_8_ (Figures 5, top and S2.13). Solvent deuteration had no effect on the decay rates of **2A** ^**iPr**^ and **2B**, and only marginally increased the stability of **2E**. This demonstrates that H-atom abstraction does not play a significant, if any, role in vinylidene decay, including the more stable species in our series. This is in stark contrast to the stabilization of **DPC** in toluene-d_8_ (Figure 5, bottom).

**Figure 5:**
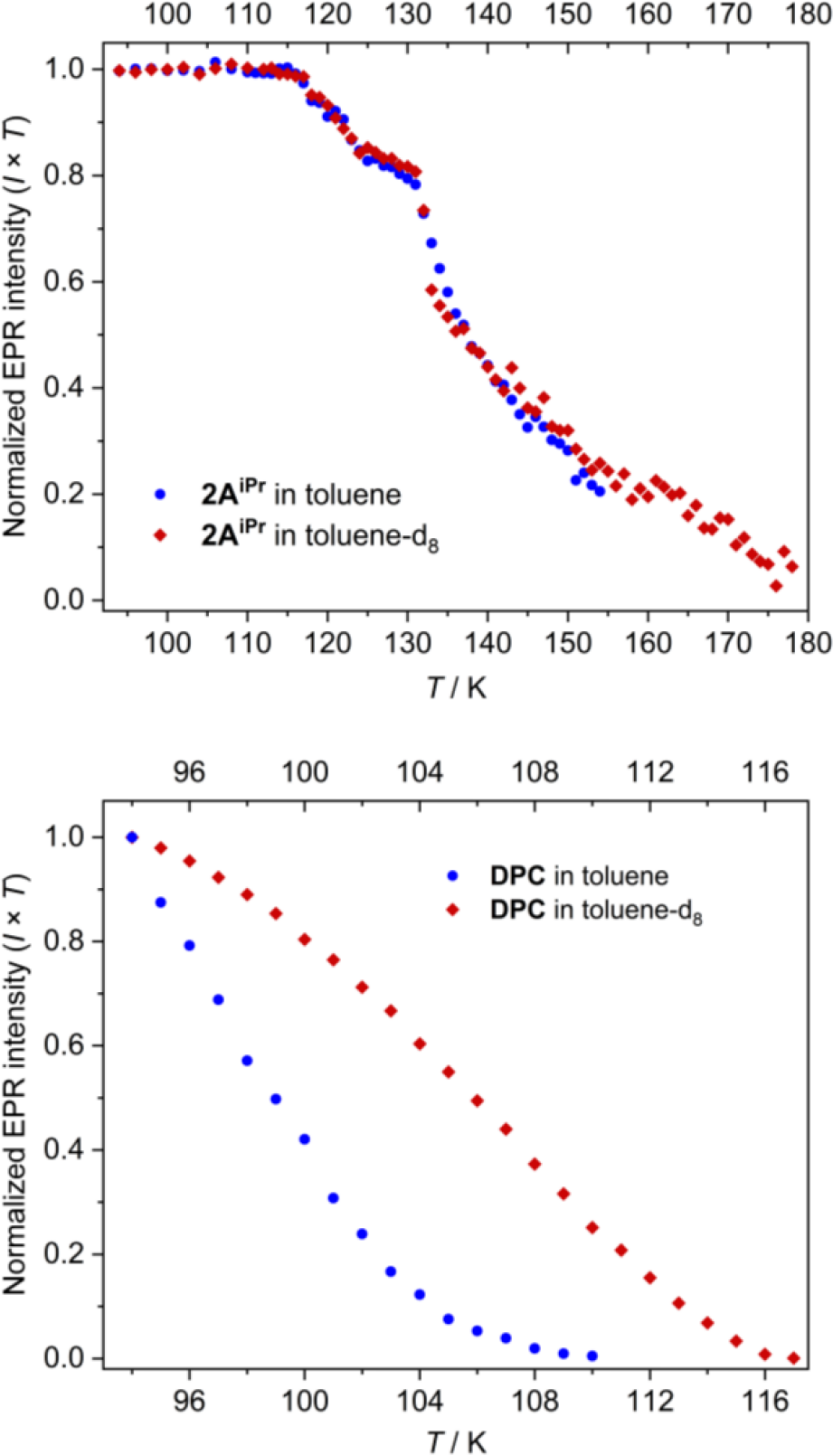
Thermal decay of EPR intensity of vinylidene **2A** ^**iPr**^ (top) and carbene **DPC** (bottom) in nondeuterated toluene (blue data points) and toluene-d_8_ (red points) measured at an increasing temperature following UV illumination at 94 K.

The decay onset temperature of ∼115 K, common for most of the studied vinylidenes, is likely connected to the toluene glass transition temperature, *T*_g_ = 117 K, where the motion of the guest molecules becomes increasingly less restricted.^43^ This is also reflected in the behavior of **2F**, where the decay process accelerates significantly as the sample temperature passes through *T*_g_. This implies that the decay process is likely driven by changes in the geometrical conformation (see mechanistic studies below).

Determination of the activation energies for vinylidenes from Arrhenius plots is hindered by nonexponential decay curves and the presence of multiple decay phases at various temperatures. For the isothermal decay curves of **2B** in the range of 118–122 K satisfactory fits could be obtained with a monoexponential function *I* = *I*_0_·e^–*t*·*k*^ (Figure S2.14). The resulting Arrhenius plot yielded *E*_a_ = 11.3 ± 1.7 kcal/mol. However, since the EPR signal of **2B** decays around *T*_g_ of toluene, this value is most likely affected by the glass transition of the solvent. The same is likely true for **2C**, **2D**, and **2F**. On the other hand, **I** decays below *T*_g_, but its traces are significantly nonexponential, typical for a process with a distribution of the exponential rate constant. Therefore, we limited the fits only to the initial 15% intensity loss (Figure S2.15), which can be interpreted as corresponding to a single conformation of the vinylidene with the highest rate constant within the distribution. This is in line with the literature on carbenes,^42^ where similar reasoning was applied to the decay involving H-atom abstraction. Although the resulting fit quality for **I** was still suboptimal, this procedure yielded an estimated activation energy of *E*_a_ ∼ 4 kcal/mol. Such complications in the experimental characterization of the decay processes unfortunately do not allow direct comparison of these values with computational results.

### Product Isolation Studies

Next, the decomposition products of the vinylidenes were analyzed by product isolation studies. We previously showed that in the case of **I**, intramolecular C-H activation of the phenyl group occurs to form product **3I** (Scheme 3A). Irradiation of **1B** with a 390 nm LED at room temperature in toluene affords cleanly the analogous C–H insertion product **3B**. Interestingly, upon irradiation of **1A** ^**iPr**^ and **1A** ^**Cl**^/**1A** ^**Br**^ in solution at room temperature, the formation of the six-membered C–H insertion product **4** arising from insertion into the Dipp-group was isolated cleanly in quantitative yields. Importantly, the *ortho*-halogen atoms (Br, Cl) as well as the ^i^Pr groups were not activated, shutting down the adjacent aryl activation pathway. Due to the orthogonal fixation of the aryl group in **1A** ^**iPr**^ and **1A** ^**Cl**^/**1A** ^**Br**^ (Scheme 2B), the C–H insertion is highly disfavored, dramatically increasing the lifetime observed by the temperature-dependent EPR studies (see above). Next, we investigated the reactivity of **2F** which appears unlikely to undergo intramolecular C–H activation into the N-Me group since this would result in a strained 4-membered ring. Interestingly, the reactivity of the small imidazole system **2D** could previously not be identified.^12b^ Irradiation of **1F** in THF or *d*_8_-THF afforded the THF C–H activation product **5** or the deuterated analog **5-d**_**8**_ (Scheme 3B). These products represent the first intramolecular trapping products of vinylidenes derived from stable diazoalkenes.

**Scheme 3:**
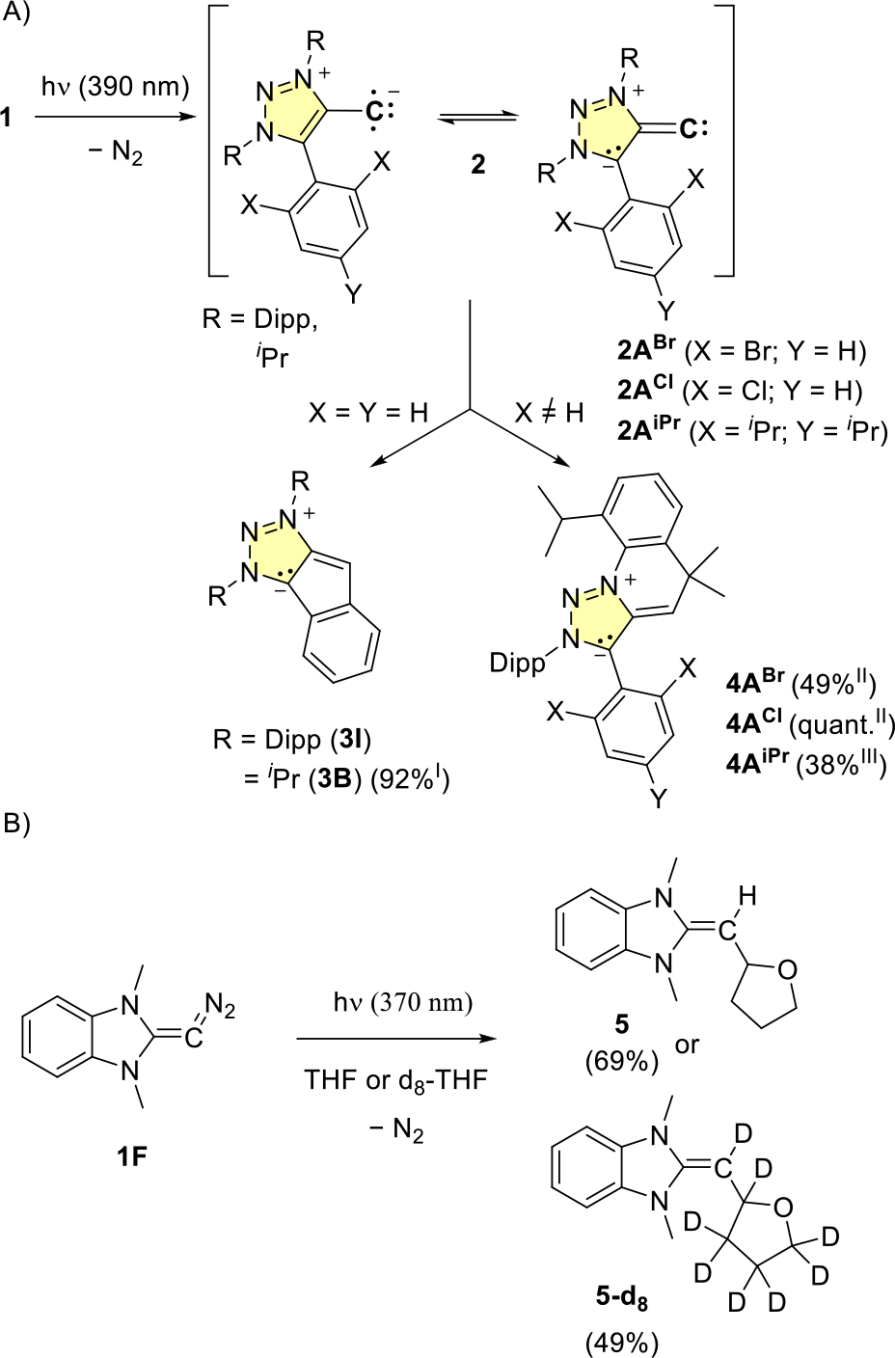
(A) Contrasting Intramolecular Five- vs Six-Membered C–H Activation Pathways at Room Temperature; (B) Intermolecular C–H Activation; Conditions I: Toluene, rt; II: C_6_D_6_, rt; III: Toluene, 0 °C

### Mechanistic Studies

Quantum chemical studies were employed to understand the mechanism of intramolecular C–H insertion that leads to the transformation of the vinylidenes toward the cyclized products. All vinylidenes in this work have triplet ground states, whereas their decay products have singlet ground states. Optimizations of the cyclized products in both singlet and triplet states confirm that the singlet-state products are at least 20 kcal mol^–1^ (see below) lower in energy than the triplets, consistent with the loss of triplet EPR signals upon C–H activation. In terms of the mechanism, this means that the reaction must involve a change in total spin. Therefore, to determine possible reaction pathways, we need to examine both the triplet and singlet potential energy surfaces, and also identify intermediate geometries in which the singlet and triplet states become (quasi-)­degenerate so that intersystem crossing from the triplet to the singlet surface can occur. In the following, we discuss in detail the transformations of ^**3**^ **I** and ^**3**^ **2A** ^**Cl**^ as representative cases of C–H activation of the phenyl and isopropyl groups, leading respectively to formation of five- and six-membered ring insertion products.

The computed reaction profiles in the triplet and singlet potential energy surfaces for ^**3**^ **I** are shown in Figure 6 (BLYP-D4 ^44^/def2-TZVP structures and relative energies). Along the triplet potential energy surface the C–H insertion is stepwise. The first step is hydrogen abstraction from the phenyl by the terminal carbon of the vinylidene via transition state ^**3**^ **TS1** at 16.5 kcal mol^–1^ above ^**3**^ **I** to yield the diradical intermediate ^**3**^ **Int** at 14.5 kcal mol^–1^. In the ^**3**^ **Int** diradical, the unpaired spin density is shared between the two carbon atoms that will be subsequently coupled (spin populations 0.91 and 0.66 for the terminal and phenyl carbon, respectively, see also Figure S3.4). Rebound of the phenyl radical of ^**3**^ **Int** to form the five-membered C–H insertion product ^**3**^ **3I** can take place through the high-energy transition state ^**3**^ **TS2** at 25.9 kcal mol^–1^. The spin density in ^**3**^ **3I** is delocalized across the π system of the fused rings (Figure S3.4). The ^**3**^ **3I** product is significantly stabilized compared to the reactant ^**3**^ **I** by −50.6 kcal mol^–1^. Nevertheless, the singlet state of the product, ^**1**^ **3I**, is considerably lower, at −86.1 kcal mol^–1^ compared to ^**3**^ **I**.

**Figure 6:**
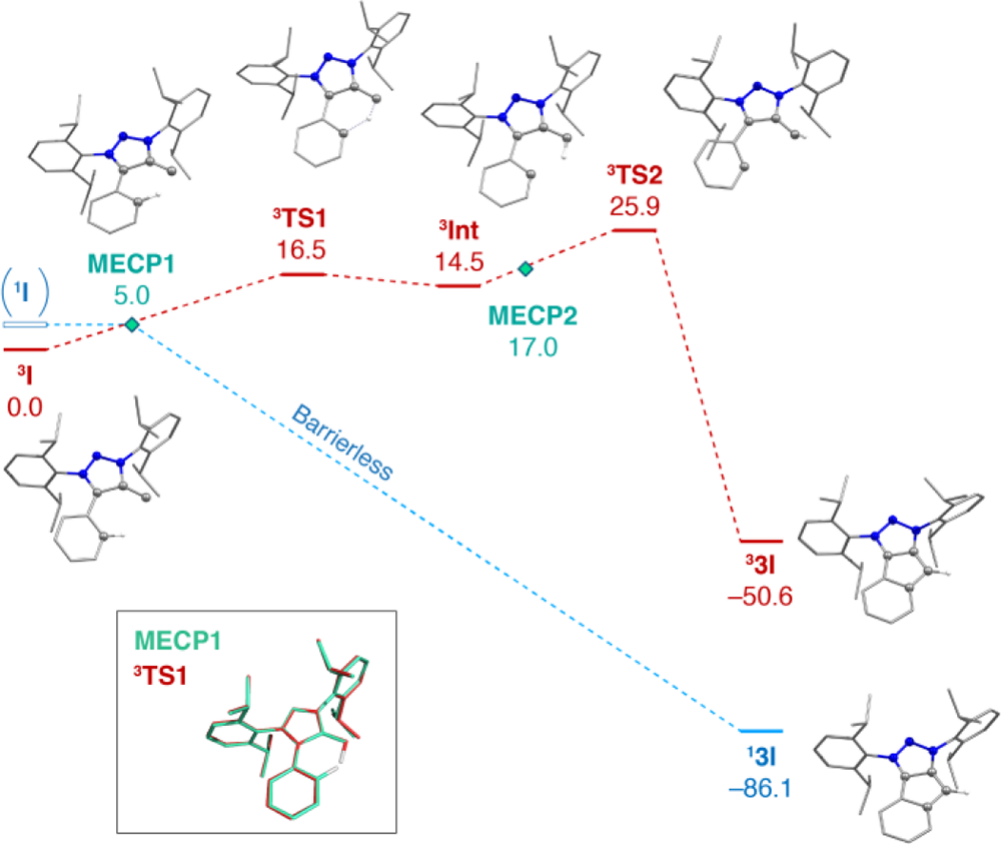
Computed (BLYP-D4/def2-TZVP) singlet (blue) and triplet (red) reaction pathway of the intramolecular C–H insertion for compound **I** to form **3I**. Electronic energies are shown in kcal mol^–1^. Hydrogen atoms not involved in the reaction are omitted for clarity. In the inset figure, the overlay of **MECP1** (cyan) with ^**3**^ **TS1** (red) is shown; the structures are almost identical, apart from the hydrogen position between the two carbon atoms.

The singlet surface has a drastically different topology compared to the triplet. At the reactant (^**3**^ **I**) geometry the vertical singlet–triplet gap is 9.8 kcal mol^–1^ (see also Table S3.3, note that here the BLYP value almost coincides with the reference double-hybrid mPW2PLYP estimate reported in Table 1). A local minimum geometry for the singlet vinylidene ^**1**^ **I** cannot be located (see Table S3.3 for more details), therefore an adiabatic singlet–triplet gap is undefined. Crucially, this is the case for all studied systems that involve phenyl C–H activation (**I**, **2B**, and **2C**). Instead, geometry optimization in the singlet state starting from the ^3^ **I** geometry leads directly to the five-membered C–H insertion product ^**1**^ **3I**, which is 86.1 kcal mol^–1^ lower than ^**3**^ **I**. This result suggests that the topology of the singlet surface is characterized by a single minimum, corresponding to the ^**1**^ **3I** product, and that spin crossing of ^**3**^ **I** from the triplet to the singlet state would be followed by barrierless product formation in a single step. Such crossing most easily occurs at the lowest-energy geometry on the seam of intersection between the two potential energy surfaces, which is known as the minimum energy crossing point (MECP).

Following the approach described by Harvey et al.^45^ as implemented in ORCA, we located an MECP between the triplet and singlet surfaces (denoted **MECP1** in Figure 6) at 5.0 kcal mol^–1^ higher than ^**3**^ **I**. This MECP involves rotation of the phenyl ring by ∼20° so that the vinylidene carbon, the triazole ring, and the phenyl ring become coplanar. Our calculations have not identified any other higher-energy intermediate between **MECP1** and ^**1**^ **3I**. This confirms that if spin crossover occurs at **MECP1**, C–H insertion into the phenyl ring proceeds in a concerted and barrierless step.

We note that the geometry of ^**3**^ **TS1** is similar to that of **MECP1**, with the triazole ring and the phenyl ring being coplanar (see overlay in the inset of Figure 6). Their main structural difference is the position of the hydrogen atom between the phenyl and vinylidene carbon atoms. The distance between the phenyl carbon and the hydrogen atom is 1.12 Å in **MECP1** and 1.54 Å in ^**3**^ **TS1**. Therefore, even though a similar ∼20° rotation of the phenyl ring in ^**3**^ **I** is needed to bring the structure close to both ^**3**^ **TS1** and **MECP1**, the hydrogen abstraction from the phenyl carbon in ^**3**^ **TS1** requires significantly higher energy. Notably, a second crossing point, **MECP2**, with a geometry similar to ^**3**^ **Int**, is located 2.5 kcal mol^–1^ higher than ^**3**^ **Int**. Therefore, spin crossing to the singlet state is also possible in principle after hydrogen abstraction on the triplet surface and before the high-barrier ^**3**^ **TS2** transition state.

The presence of an energetically accessible MECP that can effectively avoid the transition state on the potential energy surface of the reactant(s) is central to the concept of “two-state reactivity”,^46,47^ where spin crossing is implicated in the rate-determining step, as opposed to a barrier arising from a conventional transition state. In the present case, given the relative energies of **MECP1** and ^**3**^ **TS1**, the closer structural proximity of **MECP1** to ^**3**^ **I**, and the absence of further barriers on the singlet surface leading to the ^**1**^ **3I** minimum, it can be expected that spin crossing at the **MECP1** may be rate-determining and, hence, that the efficiency of the crossing to the singlet state may control the decay kinetics of ^**3**^ **I** at low temperatures.

Transitions between electronic states of different spin multiplicity are formally forbidden under quantum mechanical spin selection rules, however they become feasible under the effect of spin–orbit coupling (SOC). The probability of hopping^48,49^ from one spin surface to the other depends on the magnitude of the SOC matrix element between the wave functions of the specific states that cross at the MECP geometry, i.e., ⟨ψ_T_|*Ĥ*_SOC_|ψ_S_⟩. A SOC matrix element of zero would preclude spin crossing, while stronger SOC progressively increases the probability of transition between the two surfaces. Here we computed the SOC matrix elements using the BLYP functional with the time-dependent DFT based approach described by de Souza et al.^50^ The resulting SOC matrix element between the lowest triplet and singlet states at the **MECP1** geometry of **I** is 11.1 cm^–1^, indicating that spin crossing is allowed. We note that since the spin–orbit coupling is a one-electron property, it is not expected to be very sensitive to the level of theory, as has been shown in computational studies of intersystem crossing rates of light organic molecules.^50,51^ Consistent with this, calculations using the hybrid TPSSh and B3LYP functionals indeed provide almost identical estimates of the SOC matrix element between the lowest triplet and singlet states at the **MECP1** of **I**, 11.1 and 11.6 cm^–1^, respectively.

Activation of the isopropyl C–H bond can similarly proceed through either the stepwise triplet or the concerted singlet pathways (Figure 7). In the triplet surface, the reaction begins with hydrogen transfer from the isopropyl to the vinylidene carbon to generate the triplet diradical ^**3**^ **Int**, in which the spin density is distributed between the terminal and isopropyl carbon, with spin populations 0.77 and 0.63 respectively. ^**3**^ **Int** is more stable than the reactant ^**3**^ **2A** ^**Cl**^ by 3.4 kcal mol^–1^. Subsequently, the C–C bond can be formed via radical coupling through a low-energy transition state ^**3**^ **TS2** at 2.6 kcal mol^–1^ above ^**3**^ **Int1**, leading to the ^**3**^ **4A** ^**Cl**^ product. Notably, the relative energies of the triplet intermediates are lower for **2A** ^**Cl**^ compared to **I**, presumably because of the increased stability of the isopropyl compared to the phenyl radical.

**Figure 7:**
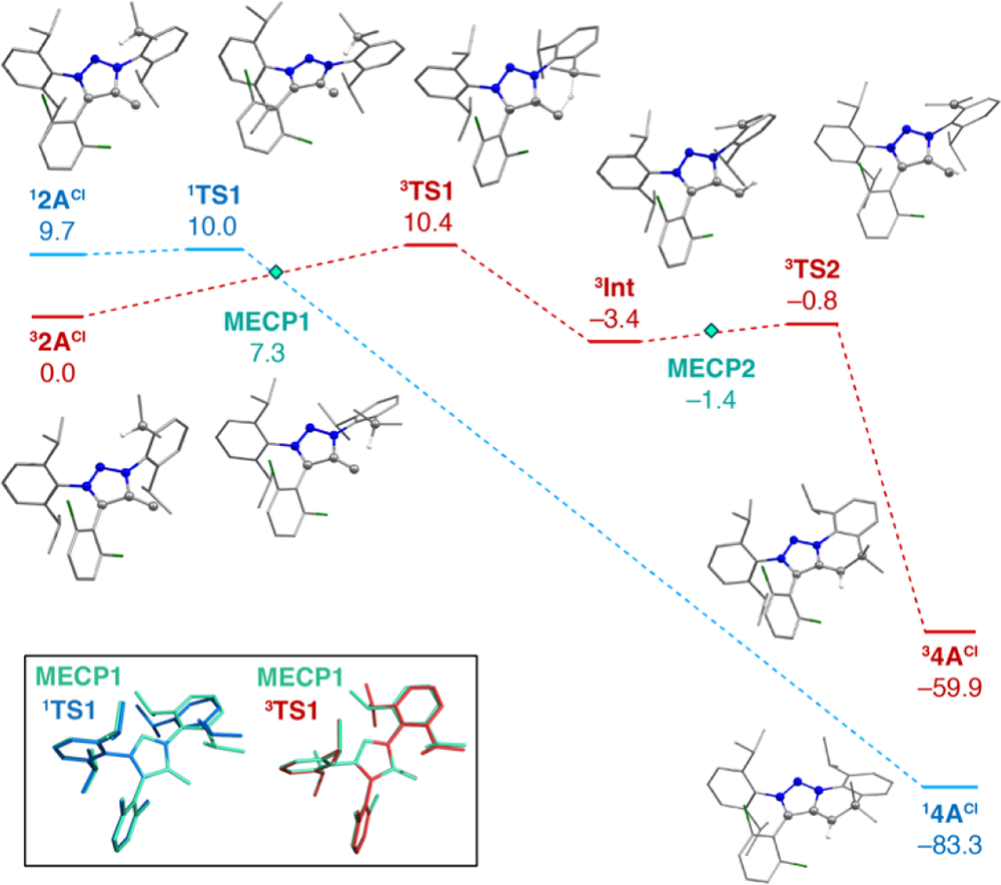
Computed (BLYP-D4/def2-TZVP) singlet (blue) and triplet (red) reaction pathways of the intramolecular C–H insertion for compound **2A** ^**Cl**^ to form the product **4A** ^**Cl**^. Electronic energies are shown in kcal mol^–1^. Hydrogen atoms not involved in the reaction are omitted for clarity. In the inset figure, the overlays of **MECP1** (cyan) with the transition states ^**1**^ **TS1** (blue) and ^**3**^ **TS1** (red) are shown; here, the hydrogen atom is omitted for clarity.

In contrast to **I**, a distinct ^**1**^ **2A** ^**Cl**^ minimum exists on the singlet potential energy surface at 9.7 kcal mol^–1^ above its triplet ground state ^**3**^ **2A** ^**Cl**^ (diabatic S/T gap = 11.7 kcal mol^–1^). The singlet pathway involves a concerted step with a transition state ^**1**^ **TS1** lying only 0.3 kcal mol^–1^ above ^**1**^ **2A** ^**Cl**^. Starting from ^**1**^ **2A** ^**Cl**^, where the triazole and Dipp phenyl ring planes are almost vertical, the Dipp ring needs to rotate by ∼25° to form ^**1**^ **TS1**, bringing the vinylidene and isopropyl carbon atoms at a distance of 3.2 Å. Formation of the six-membered ring is a concerted downhill process leading to the product ^**1**^ **4A** ^**Cl**^, which is more stable than the ^**3**^ **2A** ^**Cl**^ reactant by 83.3 kcal mol^–1^. As in the case of **I**, a crucial feature in the reactivity profile of **2A** ^**Cl**^ is the existence of **MECP1**, located 7.3 kcal mol^–1^ higher than ^**3**^ **2A** ^**Cl**^. In **MECP1** the Dipp substituent is rotated by ∼50° and the distance between the reacting carbon atoms is 2.6 Å. Besides, the distance between the propyl carbon and the hydrogen atom is similar in ^**1**^ **TS1** and **MECP1** (1.11 and 1.15 Å, respectively). Therefore, in the reaction coordinate of the C–H insertion reaction, **MECP1** can be considered to be situated “after” ^**1**^ **TS1** (see overlay of the two structures in Figure 7). Nudged elastic band calculations on the path connecting **MECP1** and the ^**1**^ **4A** ^**Cl**^ confirm that the energy of the singlet intermediate structures decreases monotonically until the ^**1**^ **4A** ^**Cl**^ product. The above results suggest that if spin crossing occurs at **MECP1**, then formation of the singlet product ^**1**^ **4A** ^**Cl**^ is a concerted, barrierless process. The computed SOC matrix element in this case is 10.4 cm^–1^, confirming that spin crossing at **MECP1** is possible. It is noted that **MECP1** is geometrically very similar to ^**3**^ **TS1** in terms of the rotation of the Dipp group, the main structural difference being that in ^**3**^ **TS1** hydrogen transfer to the vinylidene carbon is taking place.

The reaction pathways computed for compounds **2B** and **2C** are similar to those of **I**, while those of **2A** ^**Br**^ and **2E** are similar to those of **2A** ^**Cl**^, both in terms of the nature of critical points and in their energetics (see Table 2). Among **I**, **2B**, and **2C**, which involve activation of the phenyl C–H bond, **I** has the lowest-lying **MECP1** and the strongest SOC at the crossing point. This qualitatively agrees with **I** exhibiting the lowest stability in the series (Figure 4). The **2A** ^**Cl**^, **2A** ^**Br**^, and **2E**, which involve activation of the isopropyl C–H bond, have uniformly higher-lying **MECP1** points compared to **I**, **2B**, and **2C** but also lower triplet surface intermediates and transition states. Nevertheless, the **MECP1** is lower in energy than ^**3**^ **TS1** in all cases, and the SOC has similar magnitude for all compounds. Interestingly, despite the presence of Br in **2A** ^**Br**^, the SOC at **MECP1** is identical to that of **2A** ^**Cl**^, which implies the absence of a heavy atom effect. This can be rationalized by the fact that the halide does not participate in the relevant molecular orbitals involved in the triplet to singlet transition (Figure S3.4). Finally, we note that the energetic stabilization of the singlet products compared to the triplet vinylidenes is remarkably similar in all cases.

**Table 2:** Relative Energies (in kcal mol^–1^) of the First Minimum Energy Crossing Points (MECP1) Associated with Triplet-to-Singlet Crossing, and of Selected Points of the Potential Energy Surfaces^a^

	MECP1	^3^TS1	^3^Int1	^3^TS2	^3^P	^1^P
**I**	5.0 (11.1 cm^–1^)	16.5	14.5	25.9	–50.6	–86.1
**2B**	6.4 (10.2 cm^–1^)	16.6	14.6	24.5	–50.3	–86.8
**2C**	5.5 (9.4 cm^–1^)	17.2	14.4	23.7	–55.1	–85.0
**2A** ^**Cl**^	7.3 (10.4 cm^–1^)	10.4	–3.4	–0.8	–59.9	–83.3
**2A** ^**Br**^	7.3 (10.1 cm^–1^)	10.6	–3.2	–1.2	–59.7	–83.1
**2E**	9.8 (8.3 cm^–1^)	12.7	–4.2	–1.5	–30.1	–81.3

a ^3^P and ^1^P indicate the triplet and singlet cyclic products. Computed spin–orbit coupling matrix elements (in cm^–1^) are reported in parentheses for the MECP1 geometries.

Notably, a reaction profile similar to that of vinylidene insertion into the isopropyl C–H bond was predicted for intramolecular carbene insertion into an alkyl C–H bond in triplet metallocarbenes.^52^ It also involves two competing pathways that include a radical rebound pathway on the triplet potential energy surface and a concerted pathway on the singlet surface. A crucial difference is that in the case of metallocarbenes an early crossing to the singlet potential energy surface was predicted to enable access to a closed-shell singlet transition state with higher energy than the MECP,^52^ whereas in the present vinylidenes intramolecular C–H insertion is predicted to be barrierless after crossing to the singlet state.

The results of the computational studies described above lead to the following general conclusions:

1. A stepwise triplet pathway and a concerted singlet pathway are conceivable for all vinylidenes in our series.
2. In all cases there is a low-energy point (**MECP1**) where a crossing from the triplet to the singlet surface can occur.
3. This point is always lower in energy than the first transition state that would be encountered on the triplet surface (^**3**^ **TS1**) and that would lead to hydrogen abstraction as a first step of a radical rebound mechanism.
4. A smaller geometric distortion is required to reach **MECP1** than ^**3**^ **TS1** from the starting triplet vinylidene.
5. The spin crossing at **MECP1** is enabled by spin–orbit coupling that is of similar magnitude for all studied compounds.
6. The **MECP1-** ^**3**^ **TS1** energy difference is smaller and the triplet (stepwise) pathway overall is more accessible energetically in the case of aliphatic versus aromatic C–H activation, but the products are always in the singlet state and have similar stabilization.
7. The passage from the **MECP1** to the singlet product is barrierless.

These points suggest that the rate of decay of the triplet vinylidenes is ultimately determined by a combination of factors that include the relative energies of **MECP1** and ^**3**^ **TS1**, and the efficiency of spin crossing at **MECP1**. We note that in light of this description, the singlet–triplet gaps at the equilibrium geometry of the triplet vinylidenes appear less relevant to the reactivity discussion. It is stressed that the computational studies are conducted in vacuo, whereas the experimental studies are performed in the condensed phase. The temperature-dependent behavior of the toluene glass matrix (see stability measurements) may affect the observed decay behavior non-negligibly (for example, strongly differentiating the ability of phenyl and Dipp groups to rotate in order to access the **MECP1** region), thus introducing an additional complicating factor. Moreover, the present analysis does not consider intermolecular pathways that may lead to alternative decomposition products. Nevertheless, the computational studies presented in this work clarify the major intrinsic mechanistic possibilities and highlight the central role of early spin crossing from the triplet to the singlet surface.

## Conclusions

In summary we report the synthesis and characterization of eight new triplet vinylidenes, which feature four different *N*-heterocyclic substituents (1,2,3-triazole, 4-imidazole, 2-imidazole, and benzimidazole). Since prior to this study only a single example was described, this significantly widens the concept of triplet vinylidenes and shows that they form a much broader class of compounds. Considering the recent strong interest in pnictonide diradicals, this study gives a fundamental and systematic insight into the electronics and spectroscopy of a new emerging carbon diradical class. While the S/T gaps are diverse and largely tunable based on the heterocycle and substitution (9.9–18.4 kcal/mol), the axial ZFS parameter (*D* = +0.366 to +0.399 cm^–1^) and ^13^C hf tensor (*A*_iso_ ∼ 50 MHz) are in a narrow, characteristic range for the substance class. Interestingly, upon tuning of the steric environment of the vinylidene, the 5-membered C–H insertion could be shut down resulting in significantly more stable derivatives. Compounds **2A** ^**iPr**^ and the halogenated derivatives **2A** ^**Br**^ **/2A** ^**Cl**^ even show EPR signals at −123 °C, opening up pathways for magnetic/spin applications. Quantum chemical studies suggest that intramolecular functionalization of the phenyl and isopropyl C–H bonds to form the cyclized singlet decay products can proceed either via a hydrogen abstraction–radical rebound pathway in the triplet potential energy surface or via a concerted singlet pathway featuring an early spin crossing as the rate-determining step, followed by barrierless product formation. The first transition state in the triplet pathway is higher in energy and requires a larger geometric distortion compared to the minimum energy crossing point from the triplet to the singlet surface. Our results suggest that in the studied vinylidenes, intramolecular phenyl C–H insertion follows the more energetically accessible concerted singlet pathway, facilitated by spin–orbit coupling, whereas in the case of the propyl C–H insertion both the concerted and stepwise pathways are in principle accessible. Hence, the stability of the vinylidenes against intramolecular C–H functionalization depends on the efficiency of spin crossing and the accessibility of a low-energy crossing point, as well as on the energy of the triplet transition state. Considering the recent findings of room temperature stable pnictinidenes, this work constitutes the basis for raising the bar toward persistent and maybe even room temperature stable triplet vinylidenes.

## Methods

### General

C_6_D_6_ and *d*_8_-THF were distilled over sodium and stored under argon. Other solvents were taken from a PureSolv MD 7 from Inert Systems, stored over molecular sieves and degassed with argon. Reactions were carried out either under N_2_ or Ar atmosphere. Solids were handled and NMR samples were prepared in a nitrogen filled glovebox.

High resolution MS (EI): Finnigan MAT 8200 (70 eV), ESIMS: Finnigan MAT 95, accurate mass determinations: Bruker APEX III FT-MS (7 T magnet) and LTQ-Orbitrap-XL (Thermo Scientific) equipped with a heated electrospray ionization source (HESI). NMR: NMR spectra were measured on the spectrometers Bruker AV 500 Avance NEO, Bruker AV 400 Avance III HD NanoBay, AV 600 Avance III HD and AV 700 Avance III HD and chemical shifts (δ) are referenced for ^1^H and ^13^C NMR spectra to their solvent signals [C_6_D_6_, 7.16 (^1^H NMR), 128.06 (^13^C NMR); CD_3_CN, 1.94 (^1^H NMR), 118.26 (^13^C NMR), CDCl_3_ 7.26 (^1^H NMR), 77.16 (^13^C NMR), CD_2_Cl_2_, 5.32 (^1^H NMR), 54.00 (^13^C NMR), *d*_8_-THF, 3.58 (^1^H NMR), 67.57 (^13^C NMR)], ^15^N NMR are referenced against liq. NH_3_. Coupling constants (*J*) are given in Hz. All NMR spectra were recorded in 5 mm NMR tubes at the temperatures indicated. The solvent signals were used as references and the chemical shifts converted to the TMS scale. UV–vis spectra were recorded on an Agilent Cary60. Flash chromatography was performed with Merck 60 silica gel (40–63 μm). Thin-layer chromatography (TLC) analysis was performed using Merck silica gel 60 F254 TLC plates and visualized by UV irradiation and/or ceric ammonium molybdate, KMnO_4_ or *p*-anisaldehyde. All commercially available compounds (Acros, ABCR, Alfa Aesar, Sigma-Aldrich, Fluorochem) were used as received. IR-ATR measurements (diamond) were performed in reflection mode on a Bruker Alpha II inside a glovebox, wavenumbers in cm^–1^. Melting points were measured with a Büchi M-560 apparatus.

Irradiation experiments were carried out with LED lamps from Kessil of the respective wavelengths at full intensity (∼50 W) placed in ca. 20 cm distance to the solution of a diazoalkene.

1,3-Bis­(2,6-diisopropylphenyl)­triaz-1-ene (Dipp-triazene) was synthesized as described in the literature.^53^ The diazoalkenes **1C**,^12a^ **I,** ^54^ **1B,** ^27^ **1D**,^12b^ **1E**,^55^ **1F** ^29^ and the mNHO precursor for **1A** ^**iPr**^ ^56^ were synthesized as described in the literature.

### EPR Experimental Details

All samples were prepared in a nitrogen-filled glovebox. For Q-band measurements, solutions of the diazo precursors **1A–1F** and **DPC** in thoroughly degassed dry toluene were transferred into 1.6 mm quartz tubes (∼1 cm filling height), cooled to −40 °C for 15 min, sealed with BRAND sealing compound (∼5 mm length) and immediately flash-frozen in liquid nitrogen outside the glovebox. For X-band measurements, toluene solutions of the diazo precursors were transferred into 4.8 mm quartz tubes (∼2 cm filling height), sealed with the BRAND sealing compound (∼1 cm length) and immediately flash-frozen in liquid nitrogen outside the glovebox.

Q-Band pulse EPR measurements were carried out at 6 K using a Bruker Elexsys E580 spectrometer equipped with a 150 W TWT amplifier, Bruker EN 5107D2 resonator, Oxford Instruments CF935 continuous-flow helium cryostat and Oxford Instruments MercuryiTC temperature controller. Field-swept EPR spectra were detected via the free induction decay (FID) signal to avoid strong nuclear modulation artifacts found in the electron spin echo detected spectra. The microwave (MW) π/2 pulse was 500 ns. The triplet species were generated by irradiating the diazo precursors in frozen toluene solution at 10 K using a Hg arc lamp (LOT LSB610U) inside the resonator until the triplet species intensity reached a plateau (approximately 1 to 2 h). The FID-detected spectra were pseudomodulated^57^ using the fieldmod function of the EasySpin package^58^ with a modulation amplitude of 5 mT.

Orientation-selective Davies^59^ ENDOR spectra were collected with stochastic detection^60^ at 6 K using an AR 600 W radiofrequency (RF) amplifier (AR 600A225A). The following microwave pulse sequence was used: π–*T*–π/2−τ–π–τ–echo. The RF pulse was applied during the time interval *T* and had a length of 30 μs; the MW inversion π pulse was 28–30 ns; the π/2 and π detection pulses were 14 and 28 ns, respectively; the interpulse delay τ was 340 ns. Imperfections in the ^1^H and ^14^N ENDOR signal subtractions (Figure 3) are due to the variations in the MW frequency and output power of the RF amplifier, as well as EPR line shape changes upon ^13^C labeling.

Temperature-dependent X-band continuous wave (CW) EPR measurements in the temperature range of 8–50 K were carried out using a Bruker Elexsys E500 spectrometer equipped with a Bruker ER 4119 HS resonator, Oxford Instruments ESR 900 cryostat and Oxford Instruments MercuryiTC temperature controller. The spectra were recorded under nonsaturating conditions, with a modulation amplitude of 17 G. The triplet species was generated by irradiating the diazo precursor in frozen toluene solution at 10 K using a 395 nm fiber-coupled LED (Thorlabs M395FP1) inside the resonator until the triplet species intensity reached a plateau (∼40 min).

X-Band CW EPR stability measurements in the temperature range of 94–170 K were carried out using a benchtop Magnettech ESR5000 spectrometer. The spectra were recorded under nonsaturating conditions (1 mW); the modulation amplitude was 9.5G. The triplet species were generated by irradiating the diazo precursors (4 mM for **1C**, 20 mM for others) in frozen toluene solution at 94 K inside the resonator until the triplet species intensity reached a plateau (∼30 min). A Xe lamp was used for **I**, **1A** ^**iPr**^ and **1B**, and a 395 nm LED (Thorlabs M395FP1) for **1A** ^**Cl**^, **1A** ^**Br**^, **1C**, **1E**, **1F**.

EPR and ENDOR simulations were performed using the EasySpin package.^58^ The intensities of the simulated Q-band half-field (*M*_S_ = −1 ↔ 1) EPR signals at ∼550 mT were manually reduced to account for a difference in transition probabilities (Figures 1, S2.1, and S2.2). To minimize the number of simulation parameters, we used an isotropic *g*-factor *g*_iso_ = 2.0023 and only the anisotropic EPR line width parameter *Sys.HStrain* (taken into account by EasySpin for ENDOR simulations). See SI for further details.

### Computational Details

Quantum chemical calculations were performed with Orca 5.^33^ For calculations of spectroscopic properties, geometries were optimized in the triplet ground state with the hybrid TPSSh functional^34^ using the def2-TZVP basis sets.^61^ The resolution of identity approximation was used for Coulomb fitting with the def2/J basis set.^62^ The chain-of-spheres approximation (COSX)^63^ to exact exchange was used for hybrid functionals. Tight convergence criteria and increased integration grids were used in all calculations. Zero-field splitting parameters and hyperfine coupling constants were calculated with the TPSSh functional^34^ and a combination of appropriate basis sets^61,64,65^ detailed in the Supporting Information. Complete active space self-consistent field (CASSCF) calculations with the def2-TZVP basis set on simplified vinylidene models were performed using an active space of 10 electrons in 8 orbitals. State-averaged orbital optimizations were performed over different spin states, followed by N-electron valence state perturbation theory (NEVPT2)^66,67^ calculations to include dynamic electron correlation. For the description of C–H insertion mechanisms associated with the decay of triplet vinylidenes, initial geometry optimizations of intermediates and transition states were carried out using the r^2^SCAN-3c method,^68^ and the structures were subsequently refined using the BLYP^36,37^ functional combined with D4 dispersion corrections,^44^ to obtain more accurate relative energies between singlet and triplet intermediates and for identifying minimum energy crossing points (MECPs). Frequency calculations were used to confirm the nature of each stationary point. The spin–orbit coupling between the triplet and singlet states at the MECP geometries was calculated with quasi-degenerate perturbation theory^69^ using time-dependent DFT^50^ with the Tamm–Dancoff approximation.^70^

## Supplementary Material







## Notes

Synthesis and characterization; Additional experimental procedures, characterization data, NMR spectra, IR spectra, UV–vis spectra, EPR data, and computational details; and references (PDF)
Cartesian coordinates of optimized structures (TXT)
Crystal data of **1A** ^**Br**^ (CIF)
