# Supplementary material for: Triplet Vinylidenes Based on (Benz)imidazole and 1,2,3-Triazole N‑Heterocycles
Source: JACS Au. 2025 Jun 5;5(6):2884–97. doi: 10.1021/jacsau.5c00491 (PMC12188390; doi:10.1021/jacsau.5c00491)
Supplement: Supplementary file 1 [file au5c00491_si_001.pdf]

Supplementary Materials for

Triplet Vinylidenes Based on (Benz)imidazole and 1,2,3-Triazole *N*-Heterocycles

Yury Kutin,[a] Justus Reitz,[a] Maria Drosou,[b] Patrick W. Antoni,[a] Yijie He,[a] Victor R. Selve,[a] Sergius Boschmann,[a] Anton Savitsky,[c] Dimitrios A. Pantazis,[*,b] Müge Kasanmascheff,[*,a] Max M. Hansmann*[,a]

a) Department of Chemistry and Chemical Biology, Technische Universität Dortmund, Otto-Hahn-Str. 6, 44227 Dortmund, Germany;

b) Max-Planck-Institut für Kohlenforschung, Kaiser-Wilhelm-Platz 1, 45470 Mülheim an der Ruhr, Germany;

c) Department of Physics, Technische Universität Dortmund, Otto-Hahn-Str. 6, 44227 Dortmund, Germany.

Correspondence to: dimitrios.pantazis@kofo.mpg.de; muege.kasanmascheff@tu-dortmund.de; max.hansmann@tu-dortmund.de

Table of Contents

1. Synthesis and characterization 2
   1.1. Materials and methods 2
   1.2. Experimental 3
   1.3. Synthesis route for ^{13}C-labeled diazoalkenes 15
   1.4. Irradiation Experiments 19
   1.5. NMR 30
   1.6. IR-spectra 79
   1.7. SDT 86
   1.8. UV-vis spectra 87
   1.9. X-ray crystallography 92
2. EPR data 95
   2.1. EPR sample preparation 95
   2.2. EPR experimental details 95
   2.3. EPR spectra and energy level diagrams 97
   2.4. Subtraction of ^{1}H and ^{14}N ENDOR signals 101
   2.5. ^{13}C ENDOR simulations 102
   2.6. Analysis of the ^{14}N hyperfine and quadrupole interaction tensors 103
   2.7. Thermal stability studies 105
3. Quantum chemical calculations 108
   3.1. Computational details 108
   3.2. DFT description of triplet states 110
   3.3. Singlet–triplet gaps from DFT 113
   3.4. Multireference calculations on unsubstituted rings 114
   3.5. Electronic Structure of Decomposition Intermediates 117
4. References 120

1. Synthesis and characterization

1.1. Materials and methods

C_6D_6 and d_8-THF were distilled over sodium and stored under argon. Other solvents were taken from a PureSolv MD 7 from Inert Systems, stored over molecular sieves and degassed with argon. Reactions were carried out either under N_2 or Ar atmosphere. Solids were handled and NMR samples were prepared in a nitrogen filled glovebox.

High resolution MS (EI): Finnigan MAT 8200 (70 eV), ESIMS: Finnigan MAT 95, accurate mass determinations: Bruker APEX III FT-MS (7 T magnet) and LTQ-Orbitrap-XL (Thermo Scientific) equipped with a heated electrospray ionization source (HESI). NMR: NMR spectra were measured on the spectrometers Bruker AV 500 Avance NEO, Bruker AV 400 Avance III HD NanoBay, AV 600 Avance III HD and AV 700 Avance III HD and chemical shifts (δ) are referenced for ^{1}H and ^{13}C NMR spectra to their solvent signals [C_6D_6, 7.16 (^{1}H-NMR), 128.06 (^{13}C-NMR); CD_3CN, 1.94 (^{1}H-NMR), 118.26 (^{13}C-NMR), $CDCl_3$ 7.26 (^{1}H-NMR), 77.16 (^{13}C-NMR), CD_2Cl_2, 5.32 (^{1}H-NMR), 54.00 (^{13}C-NMR), d_8-THF, 3.58 (^{1}H-NMR), 67.57 (^{13}C-NMR)], ^{15}N NMR are referenced against liq. NH_3. Coupling constants (*J*) are given in Hz. All spectra were recorded in 5 mm NMR tubes at the temperatures indicated. The solvent signals were used as references and the chemical shifts converted to the TMS scale. UV-Vis spectra were recorded on an Agilent Cary60. Flash chromatography was performed with Merck 60 silica gel (40-63 µm). Thin-layer chromatography (TLC) analysis was performed using Merck silica gel 60 F254 TLC plates and visualized by UV irradiation and/or ceric ammonium molybdate, $KMnO_4$ or p-anisaldehyde. All commercially available compounds (Acros, ABCR, Alfa Aesar, Sigma Aldrich, Fluorochem) were used as received. IR-ATR measurements (diamond) were performed in reflection mode on a Bruker Alpha II inside a glovebox, wavenumbers in cm^{-1}. Melting points were measured with a Büchi M-560 apparatus.

Irradiation experiments were carried out with LED lamps from Kessil of the respective wavelengths at full intensity (~50 W) placed in ca. 20 cm distance to the solution of a diazoalkene.

1,3-bis(2,6-diisopropylphenyl)triaz-1-ene (Dipp-triazene) was synthesized as described in the literature.[1] The diazoalkenes **1C**,[2] **I**,[3] **1B**,[4] **1D**,[5] **1E**[6], **1F**[7] and the mNHO precursor for **1A^{iPr}**,[8] were synthesized as described in the literature.

1.2. Experimental

Synthesis of **1,3-dibromo-2-iodobenzene**

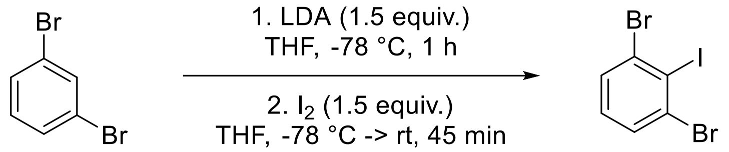

Lithium diisopropylamide (LDA) was synthesized *in situ* according to a literature procedure[9] and used immediately. Under an atmosphere of argon diisopropylamine (1.3 mL, 9.22 mmol, 1.5 equiv.) was dissolved in THF (20 mL) and cooled to -78 °C. ^{n}BuLi (1.6 M in hexane, 9.22 mmol, 5.8 mL, 1.5 equiv.) was added slowly and the solution was stirred for one hour at that temperature.

1,3-Dibromobenzene (0.74 mL, 6.15 mmol, 1.0 equiv.) was added dropwise to the solution of LDA and stirred for 2 h at that temperature. I_2 (3.35 g, 13.2 mmol, 1.04 eq.) was dissolved in THF (20 mL), cooled to –78 °C and added slowly to the lithiated bromobenzene. The reaction mixture was stirred for 15 min before warming up to room temperature for 30 min. The reaction was quenched with sat. $Na_2S_2O_3$ (30 mL), the phases were separated and the aqueous phase extracted with Et_2O (4x30 mL). The organic phases were dried over Na_2SO_4 and the solvent removed under reduced pressure. Purification by column chromatography (pentane) and drying under reduced pressure afforded the desired product (2.17 g, 6.0 mmol, 98 %) as a white solid.

^{1}H NMR (500 MHz, $CDCl_3$, 298 K): δ [ppm] = 7.56 (d, *J* = 8.0 Hz, 2 H), 7.07 (t, *J* = 8.0 Hz, 1 H); **^{13}C NMR** (126 MHz, $CDCl_3$, 298 K): δ [ppm] = 131.4, 131.2, 130.4, 109.5.

The spectroscopic data are in good agreement with the literature.[10]

Synthesis of **1,3-dichloro-2-propynylbenzene**

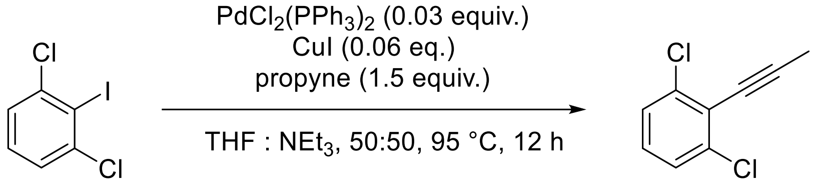

NEt_3 (25 mL) was degassed by bubbling with argon for 10 min and THF (25 mL) added. 1,3-dichloro-2-iodobenzene (1.0 g, 3.7 mmol, 1.0 equiv.) was dissolved in the solvent mixture. $PdCl_2(PPh_3)_2$ (0.21 g, 0.19 mmol, 0.05 equiv.), CuI (0.07 g, 0.7 mmol, 0.1 equiv.) and propyne (1 M in THF, 5.5 mL, 5.5 mmol, 1.5 equiv.) were added. The reaction mixture was heated to 80 °C for 12 h in a closed pressure Schlenk. The reaction mixture was cooled down, silica added to the flask and the solvent evaporated. Purification by column chromatography

(pentane) afforded **1,3-dichloro-2-propynylbenzene** (0.49 g, 2.7 mmol, 73 %) as a light-yellow solid.

^{1}H NMR (500 MHz, $CDCl_3$, 298 K): δ [ppm] = 7.29 (d, *J* = 8.1 Hz, 2 H), 7.10 (dd, *J* = 8.5, 7.7 Hz, 1 H), 2.19 (s, 3 H) ppm; **^{13}C NMR** (126 MHz, $CDCl_3$, 298 K): δ [ppm] =137.3, 128.4, 127.5, 123.9, 97.7, 74.2, 5.0 ppm.

The spectroscopic data are in good agreement with the literature.[11]

Synthesis of **1,3-dibromo-2-(prop1-yn1-yl)benzene**

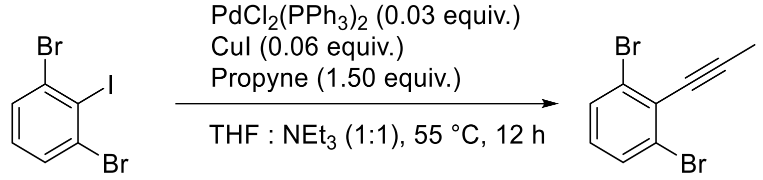

NEt_3 (40 mL) was degassed by bubbling with argon for 10 min and THF (40 mL) was added. **1,3-dibromo-2-iodobenzene** (4.2 g, 11.6 mmol, 1.0 equiv.), $PdCl_2(PPh_3)_2$ (0.24 g, 0.35 mmol, 0.03 equiv.), CuI (0.13 g, 0.7 mmol, 0.06 equiv.) and propyne (1 M in THF, 17.4 mL, 17.4 mmol, 1.50 eq.) were dissolved in the solvent mixture. The solution was heated to 55 °C and stirred for 12 h in a closed pressure Schlenk flask. After cooling to room temperature, silica was added, and the solvent removed under reduced pressure. Purification by column chromatography (pentane) afforded the desired alkyne (1.95 g, 7.12 mmol, 61 %) as a light-yellow solid.

m.p. 56 °C; **^{1}H NMR** (500 MHz, $CDCl_3$, 298 K): δ [ppm] = 7.51 (d, *J* = 8.1 Hz, 2 H, Ar-H), 6.95 (t, *J* = 8.0 Hz, 1 H, Ar-H), 2.19 (s, 3 H, CH_3), **^{13}C NMR** (126 MHz, $CDCl_3$, 298K): δ [ppm] = 131.2 (Ph-CH), 129.1 (Ph-CH), 127.7 (Ph-C_q), 126.4 (Ph-C-Br), 96.5 (alkyne-C), 78.1 (alkyne-C), 4.9 (CH_3); **IR** [cm^{-1}]: 2250, 1542, 1437, 1419, 1190, 1144, 1062, 971, 772, 716, 557. HR-APCI, HESI and ESI-MS were not successful due to decomposition during ionization. **GC-MS** calc. $C_9H_6Br_2$ $[M]^+$ calc. 271.9; found 271.9, C_9H_6Br $[M-Br]^+$ calc. 193.0; found 193.0, C_9H_6 $[M-2\ Br]^+$ calc. 114.0; found 114.1.

Synthesis of **dichloro-triazolium salt**

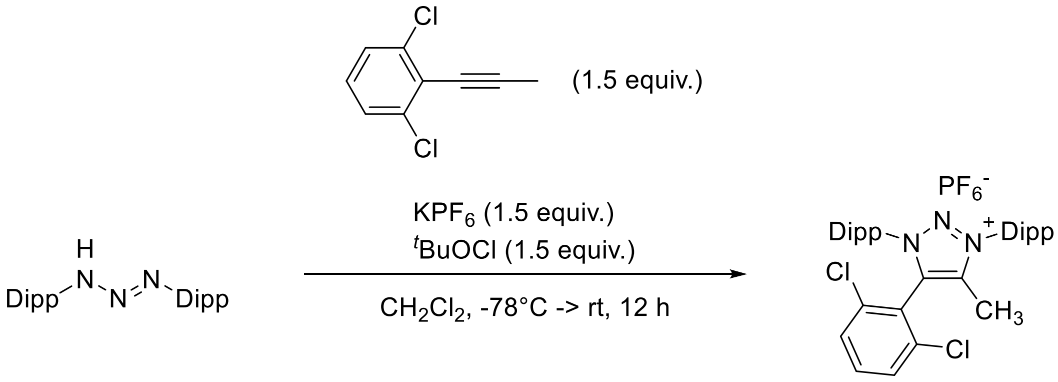

Under an atmosphere of argon, Dipp-triazene (1.21 g, 3.3 mmol, 1.0 equiv.) and KPF_6 (0.91 g, 4.9 mmol, 1.50 equiv.) were dissolved in CH_2Cl_2 (30 mL) at –78 °C. The flask was covered with aluminum foil and ^{t}BuOCl (0.55 mL, 4.9 mmol, 1.5 equiv.) was added while stirring. After stirring at that temperature for 30 min, the corresponding alkyne (0.9 g, 4.9 mmol, 1.5 equiv.) was added. The reaction was stirred for 16 h while slowly warming up in an acetone-dry ice bath and then filtered and the precipitate rinsed with CH_2Cl_2 (3 mL). The solvent of the filtrate was removed under reduced pressure and the residue dissolved in CH_2Cl_2 (10 mL). Under vigorous stirring, Et_2O (200 mL) was added. The precipitate was filtered, washed with Et_2O (20 mL) and dried under reduced pressure to afford the desired triazolium salt (1.25 g, 1.80 mmol, 54 %) as a light-beige powder.

m.p. 282°C; **^{1}H NMR** (500 MHz, $CDCl_3$, 298 K): δ [ppm] = 7.77 (t, *J* = 7.9 Hz, 1 H, Dipp-CH), 7.64 – 7.58 (m, 2 H, Dipp-CH and Ar^{Cl}-H), 7.57 (s, 1 H, Ar^{Cl}-H), 7.55 (d, *J* = 2.0 Hz, 1 H, Ar^{Cl}-H), 7.51 (d, *J* = 7.9 Hz, 2 H, Dipp-CH), 7.34 (d, *J* = 7.9 Hz, 2 H, Dipp-CH), 2.49 (hept, *J* = 6.8 Hz, 2 H, C*H*(CH_3)$_2$), 2.26 (hept, *J* = 6.9 Hz, 2 H, C*H*(CH_3)$_2$), 1.34 (d, *J* = 6.8 Hz, 6 H, CH(CH_3)$_2$), 1.24 (d, *J* = 6.8 Hz, 6 H, CH(CH_3)$_2$), 1.14 (d, *J* = 2.4 Hz, 6 H, CH(CH_3)$_2$), 1.12 (d, *J* = 2.6 Hz, 6 H, CH(CH_3)$_2$), **^{13}C NMR** (126 MHz, $CDCl_3$, 298 K): δ [ppm] = 145.7 (Dipp-C_q), 145.4 (Dipp-C_q), 144.4 (triaz-C_q), 138.8 (triaz-C_q), 136.4 (Ar^{Cl}-C_q), 135.3 (Ar^{Cl}-CH), 134.1 (Dipp-CH), 133.6 (Dipp-CH), 130.1 (Ar^{Cl}-CH), 129.3 (Dipp-C_q), 128.0 (Dipp-C_q), 125.7 (Dipp-CH), 125.3 (Dipp-CH), 119.6 (Ar^{Cl}-C_q), 29.7 (*C*H(CH_3)$_2$), 29.5 (*C*H(CH_3)$_2$), 26.8 (CH(*C*H_3)$_2$), 24.8 (CH(*C*H_3)$_2$), 23.4 (CH(*C*H_3)$_2$), 21.9 (CH(*C*H_3)$_2$), 10.6 (CH_3), **IR** [cm^{-1}]: 2965, 1468, 1432, 1389, 1368, 1333, 1229, 1195, 1060, 874, 835, 782, 758, 556, 433; **HR-MS-ESI(+)** calc. $C_{33}H_{40}Cl_2N_3^+$ [M-PF_6^-]$^+$ 548.2594, found 548.2592.

Synthesis of **dibromo-triazolium salt**

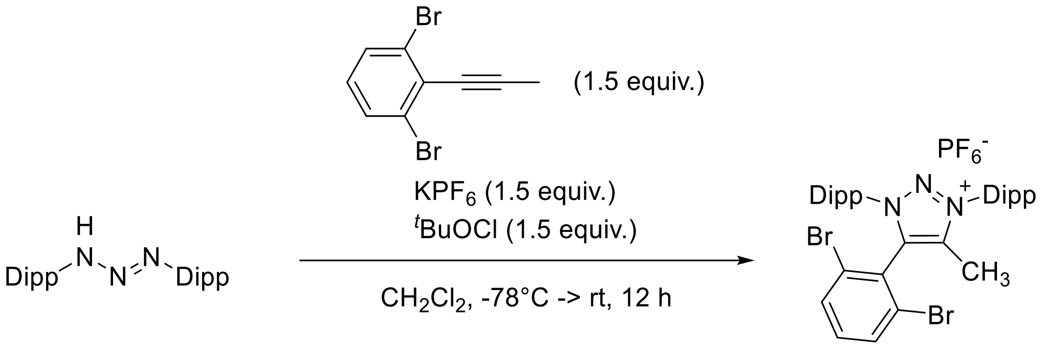

Under an atmosphere of argon, Dipp-triazene (1.1 g, 3.0 mmol, 1.0 equiv.) and KPF_6 (0.81 g, 4.4 mmol, 1.5 equiv.) were dissolved in CH_2Cl_2 (30 mL) at –78 °C. The flask was covered with aluminum foil and ^{t}BuOCl (0.50 mL, 4.4 mmol, 1.5 equiv.) was added while stirring. After stirring at that temperature for 30 min, the corresponding alkyne (0.9 g, 4.9 mmol, 1.5 equiv.) was added. The reaction was stirred for 16 h while slowly warming up in an acetone-dry ice bath and then filtered and the precipitate rinsed with CH_2Cl_2 (3 mL). The solvent of the filtrate was removed under reduced pressure and the residue dissolved in CH_2Cl_2 (10 mL). Under vigorous stirring, Et_2O (200 mL) was added. The precipitate was filtered, washed with Et_2O (20 mL) and dried under reduced pressure to afford the desired triazolium salt (1.99 g, 2.53 mmol, 84 %) as a light-beige powder.

m.p. 283 °C; **^{1}H NMR** (600 MHz, $CDCl_3$, 298 K): δ [ppm] = 7.83 – 7.72 (m, 3 H, Dipp-CH and Ar^{Br}-H), 7.62 (t, *J* = 7.8 Hz, 1 H, Dipp-CH), 7.52 (d, *J* = 7.9 Hz, 2 H, Dipp-CH), 7.45 (t, *J* = 8.2 Hz, 1 H, Ar^{Br}-H), 7.34 (d, *J* = 7.8 Hz, 2 H, Dipp-CH), 2.61 (hept, *J* = 6.8 Hz, 1 H, $C\mathit{H}(CH_3)_2$), 2.42 (s, 3 H, CH_3), 2.29 (hept, *J* = 6.8 Hz, 1H, $C\mathit{H}(CH_3)_2$), 1.34 (d, *J* = 6.8 Hz, 6 H, $CH(C\mathit{H}_3)_2$), 1.25 (d, *J* = 6.8 Hz, 6 H, $CH(C\mathit{H}_3)_2$), 1.15 (d, *J* = 6.6 Hz, 6 H, $CH(C\mathit{H}_3)_2$), 1.12 (d, *J* = 6.8 Hz, 6 H, $CH(C\mathit{H}_3)_2$), **^{13}C NMR** (151 MHz, $CDCl_3$, 298 K): δ [ppm] = 145.7 (Dipp-C_q), 145.4 (Dipp-C_q), 143.7 (triaz-C_q), 141.6 (triaz-C_q), 135.8 (Ar^{Br}-CH), 134.2 (Dipp-CH), 134.0 (Ar^{Br}-CH), 133.7 (Dipp-CH), 129.4 (Dipp-C_q), 127.9 (Dipp-C_q), 126.0 (Ar^{Br}-C_q), 125.8 (Dipp-CH), 125.4 (Dipp-CH), 123.0 (Ar^{Br}-C_q), 30.0 ($\mathit{C}H(CH_3)_2$), 29.6 ($\mathit{C}H(CH_3)_2$), 26.9 ($CH(\mathit{C}H_3)_2$), 24.8 ($CH(\mathit{C}H_3)_2$), 23.4 ($CH(\mathit{C}H_3)_2$), 22.0 ($CH(\mathit{C}H_3)_2$), 10.9 (CH_3), **IR** [cm^{-1}]: 2964, 1553, 1425, 1388, 1331, 1227, 1186, 1060, 833, 779, 758, 737, 555; **HR-MS-ESI(+)** calc. $C_{33}H_{40}Br_2N_3^+$ $[M-PF_6^-]^+$ 636.1583, found 636.1574.

Synthesis of **dichloro-mNHO**

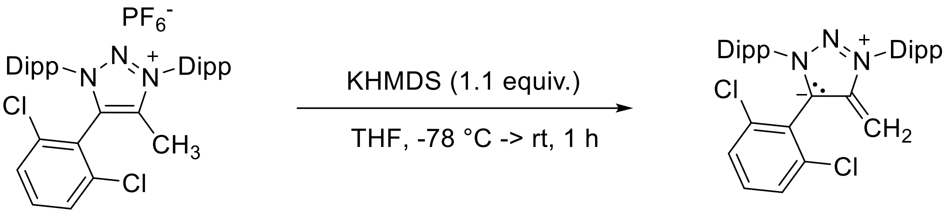

Under an atmosphere of argon, the corresponding triazolium salt (1.10 g, 1.60 mmol, 1.0 equiv.) and KHMDS (0.35 g, 1.74 mmol, 1.1 equiv.) were mixed, cooled to –78°C and then dissolved in THF (30 mL). After 15 min of stirring at that temperature the solution was warmed up to room temperature for 1 h. The solvent was evaporated under reduced pressure and the residue extracted with pentane (3 x 30 mL). The solvent was evaporated under reduced pressure to afford the mesoionic NHO (0.85 g, 1.55 mmol, 96 %) as a dark purple solid.

m.p. 169-180 °C; ^{1}**H NMR** (500 MHz, C_6D_6, 298 K): δ [ppm] = 7.29 (dd, *J* = 8.6, 6.6 Hz, 1 H, Dipp-CH), 7.25 – 7.19 (m, 2 H, Dipp-CH), 7.06 (dd, *J* = 8.4, 7.0 Hz, 1 H, Dipp-CH), 7.00 – 6.93 (m, 2 H, Dipp-CH), 6.78 (d, *J* = 8.1 Hz, 2 H, Ar^{Cl}-H), 6.28 (t, *J* = 8.1 Hz, 1 H, Ar^{Cl}-H), 3.64 (hept, *J* = 6.8 Hz, 2H, C*H*(CH_3)$_2$), 3.53 (hept, *J* = 6.7 Hz, 2H, C*H*(CH_3)$_2$), 2.81 (s, 2 H, CH_2), 1.47 (d, *J* = 6.9 Hz, 6H, CH(C*H*$_3$)$_2$), 1.32 (d, *J* = 6.9 Hz, 6 H, CH(C*H*$_3$)$_2$), 1.25 (d, *J* = 6.8 Hz, 6 H, CH(C*H*$_3$)$_2$), 1.14 (d, *J* = 6.8 Hz, 6 H, CH(C*H*$_3$)$_2$); ^{1}**H NMR** (400 MHz, *d*$_8$-THF, 298 K): δ [ppm] = 7.48 – 7.28 (m, 6 H, Ar^{Cl}-H and Dipp-CH), 7.21 (d, *J* = 7.6 Hz, 3 H, Ar^{Cl}-H and Dipp-CH), 3.38 – 3.21 (m, 4 H, C*H*(CH_3)$_2$), 2.13 (br.s, 2 H, CH_2), 1.34 (d, *J* = 6.8 Hz, 6 H, CH(C*H*$_3$)$_2$), 1.23 (d, *J* = 7.0 Hz, 6 H, CH(C*H*$_3$)$_2$), 1.14 (d, *J* = 6.8 Hz, 6 H, CH(C*H*$_3$)$_2$), 1.10 (d, *J* = 6.7 Hz, 6 H, CH(C*H*$_3$)$_2$); ^{13}**C NMR** (126 MHz, C_6D_6, 298 K): δ [ppm] = 148.8 (Dipp-C_q), 147.9 (triaz-C_q), 146.6 (Dipp-C_q), 137.7 (Ar^{Cl}-C_q), 133.1 (Dipp-C_q), 132.7 (Dipp-C_q), 131.0 (Dipp-CH), 130.4 (Dipp-CH), 129.8 (Ar^{Cl}-CH), 128.9 (Ar^{Cl}-H), 127.3 (Ar^{Cl}-CH), 124.7 (Dipp-CH), 124.2 (Dipp-CH), 116.1 (triaz-C_q), 45.9 (CH_2), 29.3 (*C*H(CH_3)$_2$), 28.9 (*C*H(CH_3)$_2$), 27.3 (CH(*C*H$_3$)$_2$), 24.4 (CH(*C*H$_3$)$_2$), 23.9 (CH(*C*H$_3$)$_2$), 22.3 (CH(*C*H$_3$)$_2$); ^{13}**C NMR** (100 MHz, *d*$_8$-THF, 298 K): δ [ppm] = 149.4 (Dipp-C_q), 148.3 (triaz-C_q), 147.2 (Dipp-C_q), 138.5 (Ar^{Cl}-C_q), 133.7 (Dipp-C_q), 133.3 (Dipp-C_q), 131.7 (Dipp-CH), 131.2 (Ar^{Cl}-CH), 130.9 (Dipp-CH), 123.0 (Ar^{Cl}-CH), 127.8 (Ar^{Cl}-C_q), 125.2 (Dipp-CH), 125.0 (Dipp-CH), 116.7 (triaz-C_q), 45.5 (CH_2), 30.0 (*C*H(CH_3)$_2$), 29.5 (*C*H(CH_3)$_2$), 27.7 (CH(*C*H$_3$)$_2$), 24.7 (CH(*C*H$_3$)$_2$), 24.1 (CH(*C*H$_3$)$_2$), 22.5 (CH(*C*H$_3$)$_2$); **IR** [cm^{-1}]: 2906, 2853, 1619, 1508, 1452, 1345, 1314, 1232, 1104, 1036, 981, 938, 915, 882, 857, 816, 744, 649, 586, 509; **HR-MS-ESI(+)** calc. $C_{33}H_{40}Cl_2N_3^+$ $[M+H]^+$ 548.2595, found 548.2606.

Synthesis of **dibromo-mNHO**

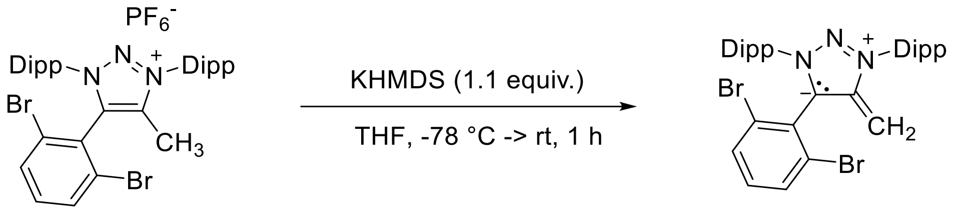

Under an atmosphere of argon, the corresponding triazolium salt (0.90 g, 1.10 mmol, 1.0 equiv.) and KHMDS (0.24 g, 1.20 mmol, 1.1 equiv.) were mixed, cooled to –78°C and then dissolved in THF (30 mL). After 15 min of stirring at that temperature the solution was warmed up to room temperature for 1 h. The solvent was evaporated under reduced pressure and the residue extracted with pentane (3 x 20 mL). The solvent was evaporated under reduced pressure to afford the mesoionic NHO (0.64 g, 1.00 mmol, 91 %) as a dark purple solid.

m.p. 165 °C; **^{1}H NMR** (600 MHz, C_6D_6, 298 K): δ [ppm] = 7.29 (dd, *J* = 8.5, 6.7 Hz, 1 H, Dipp-CH), 7.23 (d, *J* = 7.2 Hz, 2 H, Dipp-CH), 7.09 – 7.02 (m, 3 H, Dipp-CH and Ar^{Br}-H), 6.98 (d, *J* = 7.7 Hz, 2 H, Ar^{Br}-H), 6.12 (t, *J* = 8.0 Hz, 1 H, Ar^{Br}-H), 3.65 (hept, *J* = 6.9 Hz, 2 H, C*H*(CH_3)$_2$), 3.58 (hept, *J* = 6.6 Hz, 2 H, C*H*(CH_3)$_2$), 2.78 (d, *J* = 1.9 Hz, 1 H, CH_2), 2.77 (d, *J* = 1.9 Hz, 1 H, CH_2), 1.46 (d, *J* = 6.9 Hz, 6 H, CH(CH_3)$_2$), 1.33 (d, *J* = 6.9 Hz, 6 H, CH(CH_3)$_2$), 1.24 (d, *J* = 6.7 Hz, 6 H, CH(CH_3)$_2$), 1.14 (d, *J* = 6.7 Hz, 6 H, CH(CH_3)$_2$), **^{13}C NMR** (151 MHz, C_6D_6, 298 K): δ [ppm] = 148.8 (Dipp-C_q), 147.3 (triaz-C_q), 146.6 (Dipp-C_q), 133.1 (Ar^{Br}-CH), 133.1 (Dipp-C_q), 132.9 (Dipp-C_q), 131.0 (Dipp-CH), 130.7 (Ar^{Br}-C_q), 130.4 (Ar^{Br}-CH), 128.0 (Ar^{Br}-C_q), 124.7 (Dipp-CH), 124.3 (Dipp-CH), 119.5 (triaz-C_q), 46.0 (CH_2), 29.6 (*C*H(CH_3)$_2$), 28.9 (*C*H(CH_3)$_2$), 27.4 (CH(*C*H_3)$_2$), 24.5(CH(*C*H_3)$_2$), 23.9 (CH(*C*H_3)$_2$), 22.3 (CH(*C*H_3)$_2$), **IR** [cm^{-1}]: 2963, 1594, 1542, 1460, 1418, 1382, 1361, 1334, 1280, 1151, 1110, 945, 800, 773, 756, 715, 617, 561, 433; **HR-MS-ESI(+)** calc. $C_{33}H_{40}Br_2N_3^+$ $[M+H]^+$ 636.1583; found 636.1575.

Synthesis of **$1A^{Cl}$**

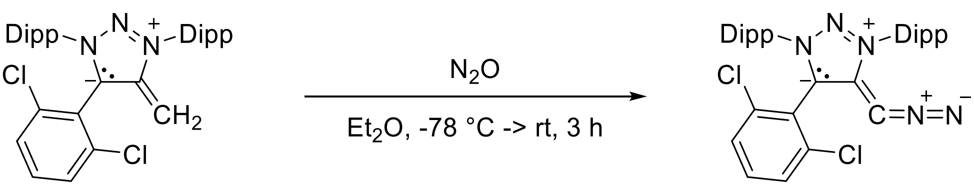

Under an atmosphere of nitrogen, **dichloro-mNHO** (550 mg, 1.00 mmol, 1.0 equiv.) was dissolved in Et_2O (15 mL). The solution was cooled to –78 °C and the inert gas was exchanged for 1 atm. N_2O, by evacuating the Schlenk flask until gas evolution from the solution ceased and repressurizing it with N_2O (quality 5.0 from Messner). The flask was closed and warmed up to room temperature. Stirring the solution for 3 h led to a change of color from dark violet solution to a brown inhomogeneous mixture. The solution was filtered off and the remaining

residue was washed with pentane (10+5 mL) and dried under reduced pressure to afford **1A^{Cl}** (272 mg, 0.47 mmol, 48%) as an orange solid.

m.p. 223 °C; **^{1}H NMR** (600 MHz, C_6D_6, 298 K): δ [ppm] = 7.27 (t, *J* = 7.7 Hz, 1 H, Dipp-CH), 7.14 (d, *J* = 7.8 Hz, 2 H, Dipp-CH), 7.03 (t, *J* = 7.8 Hz, 1 H, Dipp-CH), 6.90 (d, *J* = 7.8 Hz, 2 H, Dipp-CH), 6.78 (d, *J* = 8.1 Hz, 2 H, Ar^{Cl}-H), 6.38 (t, *J* = 8.1 Hz, 1 H, Ar^{Cl}-H), 3.13 (dhept, *J* = 10.7, 6.7 Hz, 4 H, C*H*(CH_3)$_2$), 1.46 (d, *J* = 6.9 Hz, 6 H, CH(C*H*$_3$)$_2$), 1.20 (d, *J* = 6.9 Hz, 6 H, CH(C*H*$_3$)$_2$), 1.11 (d, *J* = 6.8 Hz, 6 H, CH(C*H*$_3$)$_2$), 1.04 (d, *J* = 6.7 Hz, 6 H, CH(C*H*$_3$)$_2$); **^{13}C NMR** (151 MHz, C_6D_6, 298 K) δ [ppm] = 153.8 (triaz-C$_q$), 147.1 (Dipp-C$_q$), 146.4 (Dipp-C$_q$), 138.0 (Ar^{Cl}-C$_q$), 131.9 (Dipp-CH), 131.9 (Dipp-C$_q$), 131.9 (Ar^{Cl}-C$_q$), 131.6 (Dipp-C$_q$), 131.5 (Dipp-CH), 130.0 (triaz-C$_q$), 128.6 (Ar^{Cl}-CH), 124.5 (Dipp-CH), 124.3 (Dipp-CH), 35.5 (CN_2), 29.5 (*C*H(CH_3)$_2$), 29.4 (*C*H(CH_3)$_2$), 26.9 (CH(*C*H$_3$)$_2$), 24.0 (CH(*C*H$_3$)$_2$), 22.0 (CH(*C*H$_3$)$_2$); **IR** [cm^{-1}]: 2961, 2865, 1956 (CN_2), 1544, 1487, 1467, 1426, 1383, 1363, 1341, 1302, 1277, 1250, 1233, 1171, 1150, 1127, 1058, 1011, 982, 936, 824, 801, 787, 774, 754, 727, 703, 632, 570, 542, 433, 411; **HR-MS-ESI(+)** calc. $C_{33}H_{38}Cl_2N_5^+$ [M+H]$^+$ 574.2499, found 574.2491.

Synthesis of **1A^{Br}**

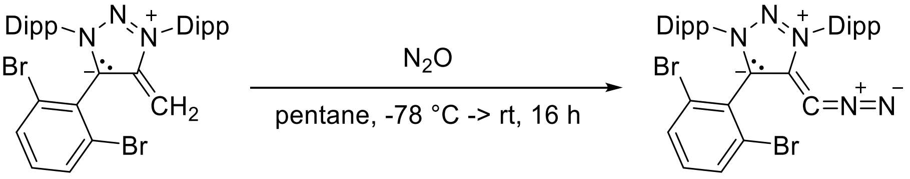

Under an atmosphere of nitrogen, **dibromo-mNHO** (235 mg, 0.37 mmol, 1.0 equiv.) was dissolved in pentane (10 mL) and 100 mg thoroughly activated 4 Å molecular sieves were added. The solution was cooled to –78 °C and the inert gas was exchanged for 1 atm. N_2O, by evacuating the Schlenk flask until gas evolution from the solution ceased and repressurizing it with N_2O (quality 5.0 from Messner). The flask was closed and warmed up to room temperature. Stirring the solution for 16 h led to a change of color from dark violet to brown. The solution was filtered off and the remaining residue was dried. The residue was extracted with toluene (20 mL) and filtered over celite. The solvent was removed under reduced pressure, dissolved in toluene (2 mL) and the diazoalkene precipitated by addition of pentane (20 mL). The solid was filtered, washed with Et_2O (2 x 1 mL) and dried under reduced pressure to afford **1A^{Br}** (71.0 mg, 0.11 mmol, 29%) as an orange solid.

m.p. 193 °C; **^{1}H NMR** (700 MHz, C_6D_6, 298 K) δ [ppm] = 7.28 (t, *J* = 7.7 Hz, 1 H, Dipp-CH), 7.15 (d, *J* = 8.6 Hz, 2 H, Dipp-CH), 7.04 (dd, *J* = 7.9, 6.1 Hz, 3 H, Ar^{Br}-H and Dipp-CH), 6.92 (d, *J* = 7.8 Hz, 2 H, Dipp-CH), 6.24 (t, *J* = 8.0 Hz, 1 H, Ar^{Br}-H), 3.23 (hept, *J* = 6.7 Hz, 2 H, C*H*(CH_3)$_2$), 3.15 (hept, *J* = 6.8 Hz, 2 H, C*H*(CH_3)$_2$), 1.47 (d, *J* = 6.8 Hz, 6 H, CH(C*H*$_3$)$_2$), 1.21 (d, *J* = 6.9 Hz, 6 H, CH(C*H*$_3$)$_2$), 1.10 (d, *J* = 6.8 Hz, 6 H, CH(C*H*$_3$)$_2$), 1.04 (d, *J* = 6.7 Hz, 6 H,

CH(CH_3)$_2$), **^{13}C NMR** (176 MHz, C_6D_6, 298 K) δ 153.2 (triaz-C_q), 147.1 (Dipp-C_q), 146.4 (Dipp-C_q), 133.2 (triaz-C_q), 132.6 (Ar^{Br}-CH), 132.4 (Ar^{Br}-CH), 131.9 (Dipp-CH), 131.9 (Dipp-C_q), 131.8 (Dipp-C_q), 131.5 (Dipp-CH), 128.4 (Ar^{Br}-C_q), 127.8 (Ar^{Br}-C_q), 124.6 (Dipp-CH), 124.3 (Dipp-CH), 35.3 (CN_2), 29.7 (*C*H(CH_3)$_2$), 29.5 (*C*H(CH_3)$_2$), 26.9 (CH(*C*H_3)$_2$), 24.1 (CH(*C*H_3)$_2$), 24.1 (CH(*C*H_3)$_2$), 22.1 (CH(*C*H_3)$_2$), **IR** [cm^{-1}]: 2961, 2866, 1950 (CN_2), 1541, 1465, 1423, 1383, 1363, 1338, 1312, 1297, 1276, 1250, 1232, 1164, 1145, 1125, 1043, 1005, 978, 937, 822, 807, 800, 782, 754, 725, 634, 568, 537, 429; **HR-MS-ESI(+)** calc. $C_{33}H_{38}Br_2N_5^+$ $[M+H]^+$ 662.1488, found 662.1491.

Synthesis of **$1A^{iPr}$**

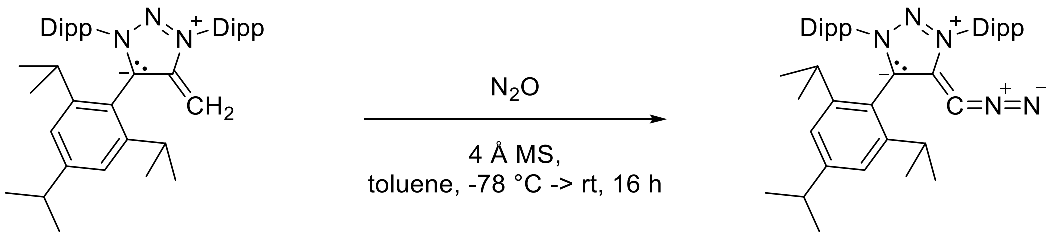

Under an atmosphere of argon, the respective mNHO (360 mg, 0.59 mmol, 1.0 equiv.) was dissolved in diethyl ether (40 mL) and 300 mg thoroughly activated 4 Å molecular sieves were added. The solution was cooled to –78 °C and the inert gas was exchanged for 1 atm. N_2O, by evacuating the Schlenk flask until gas evolution from the solution ceased and repressurizing it with N_2O (quality 5.0 from Messner). The flask was closed and warmed up to room temperature. Stirring the solution for 16 h led to a change of color from dark violet over brown to bright orange. The solution was filtered off and the remaining residue was washed four times with toluene (4 x 20 mL). The crude product was washed twice with pentane (2 x 5 mL). Drying under reduced pressure afforded the diazoalkene **$1A^{iPr}$** (190 mg, 0.30 mmol, 51 %) as a yellow solid.

m.p. 160 °C decomp.; **^{1}H NMR** (600 MHz, C_6D_6, 298 K): δ [ppm] = 7.31 (t, *J* = 7.8 Hz, 1 H, Ar-H), 7.18 – 7.16 (m, 2 H, Ar-H), 7.08 (s, 2 H, Ar-H), 7.04 (t, *J* = 7.8 Hz, 1 H, Ar-H), 6.90 (d, *J* = 7.8 Hz, 2 H, Ar-H), 3.06 (hept, *J* = 6.8 Hz, 4 H, C*H*(CH_3)$_2$), 2.72 (hept, *J* = 6.9 Hz, 3 H, C*H*(CH_3)$_2$), 1.55 (d, *J* = 6.7 Hz, 6 H, CH(C*H*$_3$)$_2$), 1.52 (d, *J* = 6.8 Hz, 6 H, CH(C*H*$_3$)$_2$), 1.24 (d, *J* = 6.9 Hz, 6 H, CH(C*H*$_3$)$_2$), 1.13 (d, *J* = 6.9 Hz, 6 H, CH(C*H*$_3$)$_2$), 1.08 (d, *J* = 6.8 Hz, 6 H, CH(C*H*$_3$)$_2$), 0.97 (d, *J* = 6.7 Hz, 6 H, CH(C*H*$_3$)$_2$), 0.87 (d, *J* = 6.7 Hz, 6 H, CH(C*H*$_3$)$_2$); **^{13}C NMR** (151 MHz, C_6D_6, 298 K): δ [ppm] = 154.0 (triaz-C_q), 151.4 (Ar-C_q), 150.1 (Ar- C_q), 147.3 (Ar-C_q), 145.7 (Ar- C_q), 132.8 (Ar- C_q), 132.5 (triaz-C_q), 132.1 (Ar-C_q), 131.6 (Ar-CH), 131.4 (Ar-CH), 125.0 (Ar-CH), 124.4 (Ar-CH), 122.4 (Ar-CH), 120.9 (Ar-C_q), 34.6 (*C*H(CH_3)$_2$), 34.3 (CN_2), 31.9(*C*H(CH_3)$_2$), 29.9 (*C*H(CH_3)$_2$), 29.8 (*C*H(CH_3)$_2$), 26.6 (CH(*C*H_3)$_2$), 26.2 (CH(*C*H_3)$_2$), 24.3 (CH(*C*H_3)$_2$), 24.1 (CH(*C*H_3)$_2$), 23.9 (CH(*C*H_3)$_2$), 23.1 (CH(*C*H_3)$_2$), 21.7 (CH(*C*H_3)$_2$); **^{15}N NMR** (61 MHz, C_6D_6, 298 K): δ [ppm] = 324.18, 285.41, 259.04, 243.47, 226.12; **IR** [cm^{-1}]: ṽ = 2961,

1959 (CN_2), 1540, 1459, 1383, 1161, 1130, 979, 804, 752; **HR-MS-ESI(+)** calc. $C_{84}H_{115}N_{10}^+$ $[2M+H]^+$ 1263.9301; found 1263.9422.

Temperature dependence of dichloro-mNHO

^{1}H NMR spectra of **dichloro-mNHO**, show a broadening of the CH_2 NMR signal, which was followed by T-dependent NMR (Figure S1.1). ^{1}H NMR spectra were recorded at varying temperatures and a coalescence temperature could be observed at 10 °C.

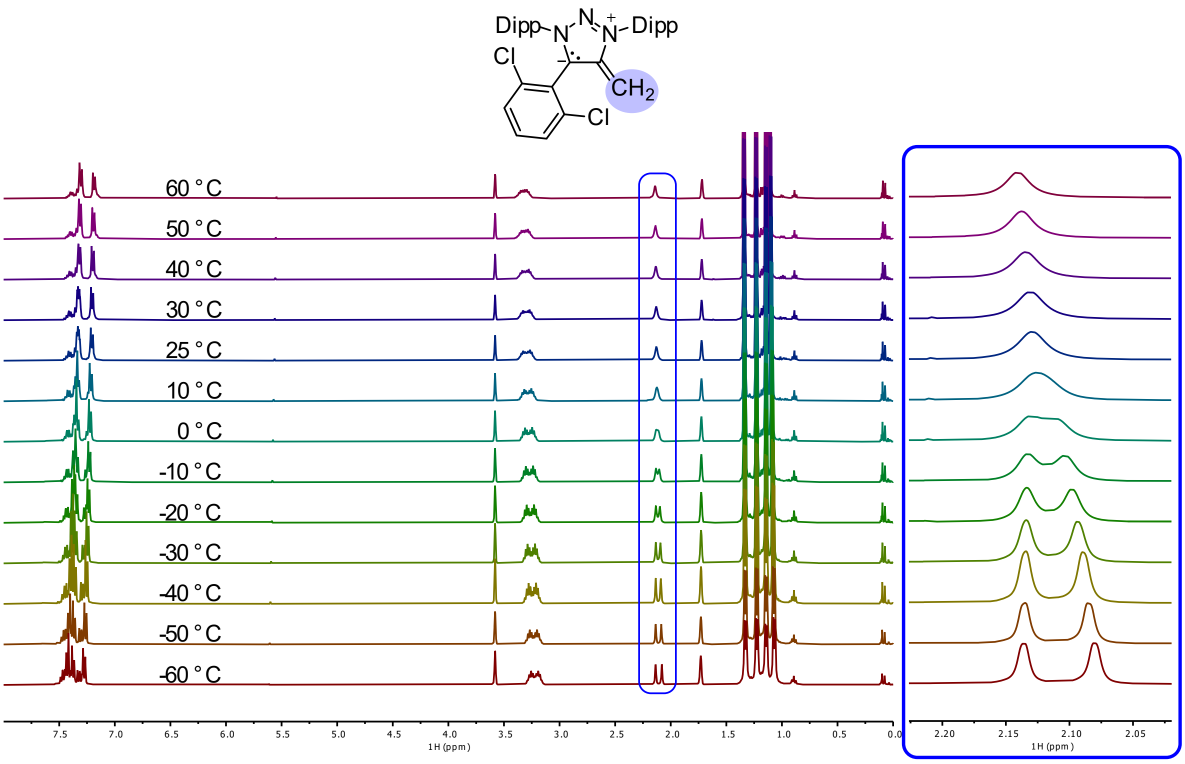

Figure S1.1. Stacked **^{1}H NMR** (400 MHz, d_8-THF) spectra of **dichloro-mNHO** from 60 °C to –60 °C. Cutout (blue frame) shows a zoom-in on the signal of the CH_2-group.

Synthesis Route for Ph_2CN_2

The synthesis of diphenyl diazomethane and ^{13}C-labeled diphenyl diazomethane were carried out according to literature procedures.[12]

Synthesis of **^{13}C-labeled benzophenone**

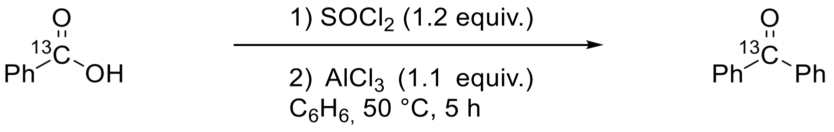

Under an atmosphere of argon, ^{13}C benzoic acid (185 mg, 1.50 mmol, 1.0 equiv.) were dissolved in $SOCl_2$ (0.13 mL, 1.80 mmol, 1.2 equiv.) and heated to reflux for 2 h. The reaction

solution was evaporated under reduced pressure and the solid residue was dissolved in benzene (5 mL) and $AlCl_3$ (220 mg, 1.65 mmol, 1.1 equiv.) added. The mixture was heated to 50 °C for 5 h, cooled to room temperature and aqueous HCl (1 M, 20 mL) added. The phases were separated and the aqueous phase was extracted with Et_2O (3 x 20 mL). The combined organic phases were dried over Na_2SO_4, the solvent evaporated and the crude product was purified by column chromatography (pentane:EtOAc, 8:1) to afford **^{13}C-labeled benzophenone** (76.1 mg, 0.45 mol, 28 %) as a colorless solid.

^{1}H NMR (501 MHz, $CDCl_3$, 298 K): δ [ppm] = 7.85 – 7.78 (m, 4 H), 7.63 – 7.56 (m, 2 H), 7.53 – 7.45 (m, 4 H).; **^{13}C NMR** (126 MHz, $CDCl_3$, 298 K): δ [ppm] = 196.9 (^{13}C), 137.8 (d, $^1J_{C\text{-}C}$ = 54.8 Hz), 132.6, 130.2 (d, $^3J_{C\text{-}C}$ = 2.8 Hz), 128.42 (d, $^2J_{C\text{-}C}$ = 3.9 Hz).

The spectroscopic data are in agreement with the literature.[13]

Synthesis of **benzophenone tosylhydrazone**

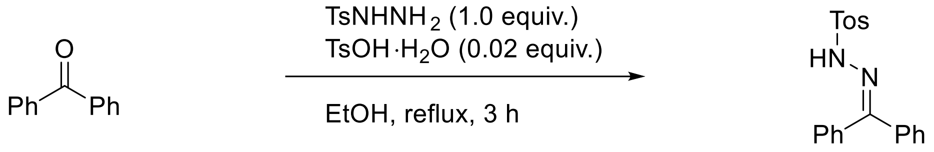

Under an atmosphere of argon, benzophenone (200 mg, 1.10 mmol, 1.0 eq.) was dissolved in ethanol (1.5 mL) and $TsNHNH_2$ (204 mg, 1.10 mmol, 1.0 eq.) and *p*-toluenesulfonic acid monohydrate (4.20 mg, 0.02 mmol, 0.02 equiv.) were added. The solution was stirred for 3 h at reflux, cooled down to room temperature and filtered. The residue was washed with ethanol (0.5 mL) and Et_2O (2 x 0.5 mL). Drying under reduced pressure afforded the corresponding **benzophenone tosylhydrazone** (314.5 mg, 0.90 mmol, 82 %) as a colorless powder.

^{1}H NMR (501 MHz, $CDCl_3$, 298 K): δ [ppm] = 7.89 – 7.83 (m, 2 H), 7.52 (qd, J = 4.3, 1.5 Hz, 4 H), 7.48 – 7.42 (m, 2 H), 7.37 – 7.32 (m, 3 H), 7.32 – 7.25 (m, 2 H), 7.17 – 7.09 (m, 2 H), 2.44 (s, 3 H); **^{13}C NMR** (126 MHz, $CDCl_3$, 298 K): δ [ppm] = 154.4, 144.3, 136.6, 135.7, 131.3, 130.3, 130.0, 129.9, 129.8, 128.4, 128.4, 128.1, 127.8, 21.8.

The spectroscopic data are in agreement with the literature.[14]

Synthesis of **benzophenone tosylhydrazone sodium salt**

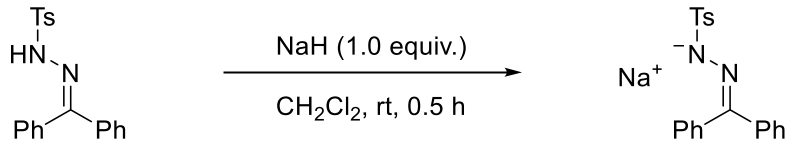

Under an atmosphere of nitrogen, tosylhydrazone (150 mg, 0.43 mmol, 1.0 equiv.) was dissolved in CH_2Cl_2 (4 mL) and NaH (10.3 mg, 0.43 mmol, 1.0 equiv.) was added. The mixture was stirred for 0.5 h until a clear solution formed. Et_2O (20 mL) was added and the solution stored for 3 h at –40 °C, upon which the product precipitated. The solid was filtered, washed with Et_2O (2 mL) and dried under reduced pressure to afford the sodium salt (124 mg, 0.33 mmol, 78 %) as a colorless powder.

^{1}H NMR (501 MHz, d_6-DMSO, 298 K): δ [ppm] = 7.60 (d, *J* = 7.8 Hz, 2 H), 7.35 (t, *J* = 7.5 Hz, 2 H), 7.24 (ddd, *J* = 12.6, 7.6, 4.2 Hz, 6 H), 7.20 – 7.14 (m, 4 H), 7.07 (t, *J* = 7.2 Hz, 1 H), 2.30 (s, 3 H); **^{13}C NMR** (126 MHz, d_6-DMSO, 298 K): δ [ppm] = 144.6, 141.7, 138.7, 138.1, 130.3, 128.6, 128.0, 127.1, 126.8, 126.0, 125.8, 21.3.

The spectroscopic data are in agreement with the literature.[15]

Synthesis of **diphenyl diazomethane**

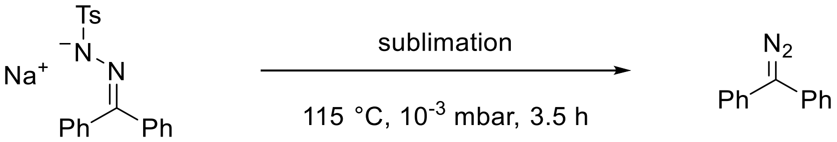

Under an atmosphere of nitrogen, tosylhydrazone sodium salt (200 mg, 0.54 mmol, 1.0 equiv.) was placed in a Schlenk flask equipped with a cooling finger that was loaded with a dry ice / acetone mixture. The Schlenk was placed in an oil bath, evacuated and then slowly heated to 115 °C. After 3.5 h the apparatus was disassembled and the solid collected to afford Ph_2CN_2 (87.5 mg, 0.45 mmol, 84 %) as a purple solid.

^{1}H NMR (501 MHz, $CDCl_3$, 298 K): δ [ppm] = 7.43 – 7.35 (m, 4 H), 7.34 – 7.27 (m, 4 H), 7.19 (tt, *J* = 7.4, 1.3 Hz, 2 H); **^{13}C NM R** (126 MHz, $CDCl_3$, 298 K): δ [ppm] = 129.7, 129.3, 125.8, 125.3.

The carbon ^{13}C-N2 is not visible as reported in the literature. The spectroscopic data are in agreement with the literature.[13]

The ^{13}C-labeled compound **^{13}C-diphenyl diazomethane** was prepared accordingly.

^{1}H NMR (501 MHz, $CDCl_3$, 298 K): δ [ppm] = 7.40 (tt, *J* = 7.3, 0.9 Hz, 4 H), 7.40 – 7.26 (m, 4 H), 7.20 (tq, *J* = 7.5, 1.1 Hz, 2 H); **^{13}C NMR** (126 MHz, $CDCl_3$, 298 K): δ [ppm] = 129.7 (d, $^1J_{C\text{-}C}$ = 67.9 Hz), 129.27 (d, $^2J_{C\text{-}C}$ = 4.8 Hz), 125.8 (d, $^4J_{C\text{-}C}$ = 1.0 Hz), 125.3 (d, $^3J_{C\text{-}C}$ = 2.9 Hz), 62.5 ($^{13}CN_2$).

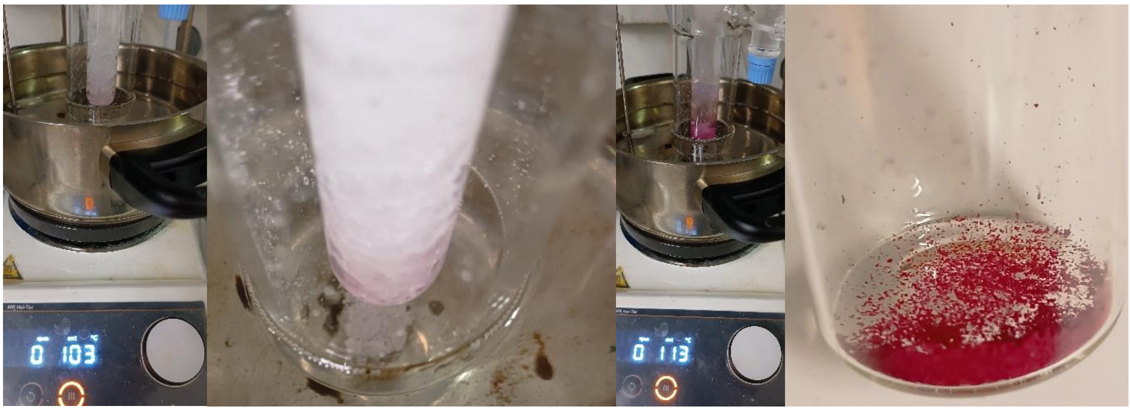

Figure S1.2. Process of sublimation of diphenyl diazomethane. From left to right: Heating the evacuated Schlenk flask with a cooling finger leads to initial sublimation of diphenyl diazomethane condensation. Heating for 3 h at 113 °C leads to the formation of a thick, violet crust on the cooling finger that can be collected as a red-purple powder.

1.3. Synthesis route for ^{13}C-labeled diazoalkenes

Synthesis of **^{13}C-1,3,5-triisopropyl-2-(prop-1-yn-1-yl)benzene**

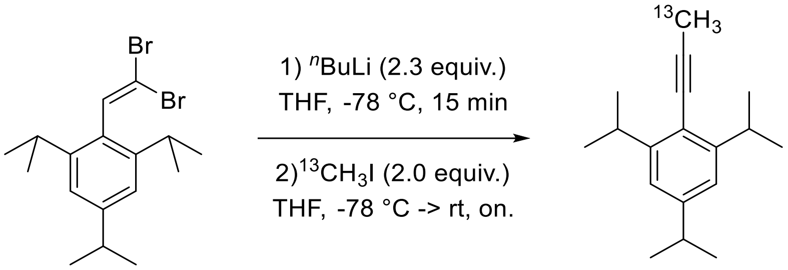

2-(2,2-dibromovinyl)-1,3,5-triisopropylbenzene was synthesized as previously described and the synthesis route followed the same pathway as for the unlabeled compound.[8]

Under an atmosphere of argon, 2-(2,2-dibromovinyl)-1,3,5-triisopropylbenzene (1.0 g, 2.58 mmol, 1.0 equiv.) was dissolved in THF (40 mL) and cooled to –78 °C. At this temperature ^{n}BuLi (2.37 mL, 2.5 M in hexane, 5.92 mmol, 2.30 eq.) was added dropwise over a period of 15 minutes. The reaction was stirred for 1 h at –78 °C followed by addition of ^{13}C labeled methyl iodide (320 µL, 5.15 mmol, 2.0 eq.). After allowing the solution to warm to ambient temperature overnight water was added (50 mL), and the organic phase was washed with water (100 mL) and brine (100 mL). The solvent of the organic phase was evaporated under reduced pressure and purified by column chromatography (pentane) furnishing ^{13}C-labeled alkyne (600 mg, 2.48 mmol, 96%) as a colorless oil.

^{1}H NMR (400 MHz, $CDCl_3$, 298 K): δ [ppm] = 6.95 (s, 2 H), 3.51 (hept, J = 6.9 Hz, 2 H), 2.88 (hept, J = 6.9 Hz, 1 H), 2.13 (d, $^{1}J_{H\text{-}C}$ = 131.0 Hz, 3 H, $^{13}CH_3$), 1.26 (d, J = 7.0 Hz, 12 H), 1.26 (d, J = 6.8 Hz, 6 H); **^{13}C NMR** (101 MHz, $CDCl_3$, 298 K): δ [ppm] = 150.5, 148.2, 120.3, 119.4 (d, $^{3}J_{C\text{-}C}$ = 1.6 Hz), 92.6 (d, $^{1}J_{C\text{-}C}$ = 68.7 Hz), 76.64 (d, $^{2}J_{C\text{-}C}$ = 12.0 Hz), 34.6, 31.8, 24.1, 23.5, 4.8 ($^{13}CH_3$).

Synthesis of **^{13}C-Tripp-triazolium salt**

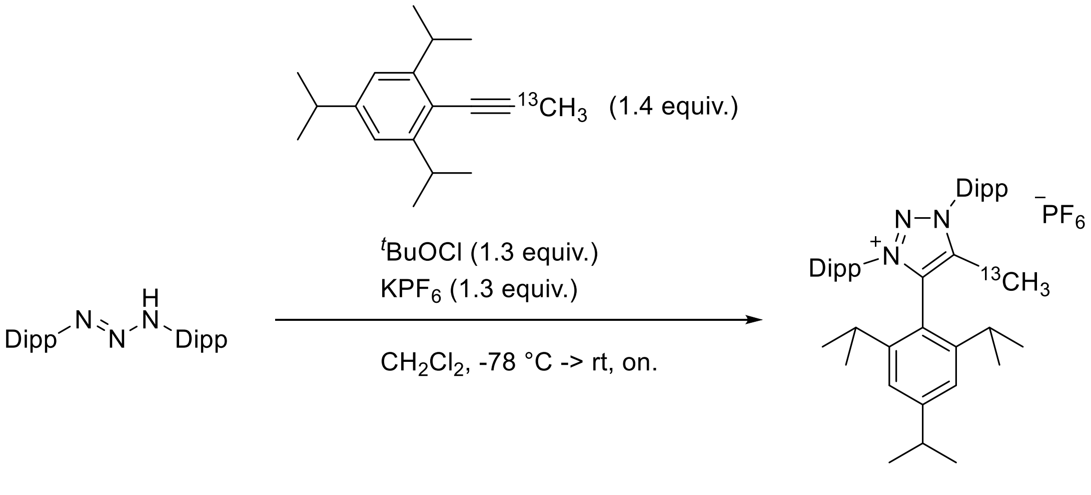

Under an atmosphere of argon, to a solution of Dipp-triazene (646 mg, 1.77 mmol, 1.0 equiv.) in CH_2Cl_2 (25 mL) was added ^{t}BuOCl (260 µL, 2.30 mmol, 1.3 equiv.) and KPF_6 (423 mg, 2.30 mmol, 1.30 equiv.) at –78 °C. After stirring the mixture for 30 minutes under exclusion of light, the corresponding alkyne (600 mg, 2.48 mmol, 1.40 eq.) was added in one portion. The solution was slowly warmed up to room temperature overnight, filtrated over a glass frit, the solvent removed under reduced pressure and the resulting reddish solid was stirred with Et_2O (100 mL) for 1 h. Filtration and drying under reduced pressure furnished the desired ^{13}C-labeled triazolium salt as colorless solid (635 mg, 0.84 mmol, 48 %).

^{1}H NMR (500 MHz, $CDCl_3$, 298 K): δ [ppm] = 7.78 (t, *J* = 7.9 Hz, 1 H), 7.61 (t, *J* = 7.8 Hz, 1 H), 7.53 (d, *J* = 7.9 Hz, 2 H), 7.35 (d, *J* = 7.9 Hz, 2 H), 7.13 (s, 2 H), 2.92 (hept., *J* = 6.9 Hz, 1 H), 2.38 (d, $^{1}J_{H-C}$ = 133.2 Hz, 3 H, $^{13}CH_3$), 2.30 (hept., *J* = 6.7 Hz, 2 H), 2.23 – 2.08 (m, 4 H), 1.38 (d, *J* = 6.9 Hz, 6 H), 1.28 (d, *J* = 6.8 Hz, 6 H), 1.23 (d, *J* = 7.0 Hz, 6 H), 1.18 (d, *J* = 6.8 Hz, 6 H), 1.13 (d, *J* = 6.8 Hz, 6 H), 0.93 (d, *J* = 6.8 Hz, 6 H), 0.92 (d, *J* = 6.8 Hz, 6 H); **^{13}C NMR** (126 MHz, $CDCl_3$, 298 K): δ [ppm] = 154.6, 149.1, 145.3, 145.0, 143.6 (d, $^{1}J_{C-C}$ = 51.4 Hz), 142.0 (d, $^{2}J_{C-C}$ = 5.1 Hz), 134.2, 133.3, 130.2, 128.1, 126.0, 125.9, 123.6, 115.1, 34.6, 31.7, 30.3, 29.9, 26.6, 26.5, 25.1, 23.7, 23.5, 22.7, 21.6, 11.4 ($^{13}CH_3$).

Synthesis of **^{13}C-Tripp-mNHO**

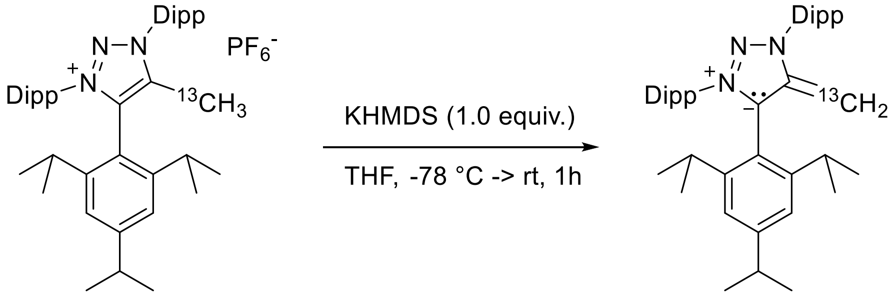

Under an atmosphere of argon, the corresponding ^{13}C labeled triazolium salt (475 mg, 632 µmol, 1.0 eq.) and KHMDS (138 mg, 695 µmol, 1.1 eq.) were mixed as solids and cooled to -78 °C. THF (40 mL) was added, leading to an instantaneous change of color to dark purple. The reaction mixture was stirred for 15 minutes at -78 °C and 1 h at ambient temperature. After removing the solvent under reduced pressure, the product was extracted with pentane (filter cannula, 3 x 20 mL). The solvent was removed under reduced pressure, furnishing the desired ^{13}C labeled mNHO (340 mg, 561 µmol, 89%) as a dark purple solid.

^{1}H NMR (600 MHz, C_6D_6, 298 K): δ [ppm] = 7.29 (dd, *J* = 8.5, 6.9 Hz, 1 H), 7.23 (d, *J* = 7.3 Hz, 2 H), 7.07 (s, 2 H), 7.05 (d, *J* = 7.7 Hz, 1 H), 6.96 (d, *J* = 7.6 Hz, 2 H), 3.52 (hept., *J* = 6.9 Hz, 2 H), 3.38 (hept., *J* = 6.9 Hz, 2 H), 3.16 (hept., *J* = 6.7 Hz, 2 H), 2.83 (d, $^{1}J_{H-C}$ = 86.9 Hz, 1 H, $^{13}CH_2$), 2.72 (hept., *J* = 6.9 Hz, 1 H), 2.56 (d, $^{1}J_{H-C}$ = 84.1 Hz, 1 H, $^{13}CH_2$), 1.45 (d, *J* = 6.9 Hz, 6 H), 1.42 (d, *J* = 6.7 Hz, 6 H), 1.34 (d, *J* = 6.9 Hz, 6 H), 1.23 (d, *J* = 6.8 Hz, 6 H), 1.15 (d, *J* =

6.9 Hz, 6 H), 1.04 (d, *J* = 6.8 Hz, 6 H), 0.96 (d, *J* = 6.8 Hz, 6 H); **^{13}C NMR** (151 MHz, C_6D_6, 298 K): δ [ppm] = 151.6 (d, $^{1}J_{C\text{-}C}$ = 82.1 Hz), 150.6, 149.7, 148.8, 145.9, 133.9, 133.6, 130.4, 130.2, 124.8, 124.7, 124.0, 122.6, 119.2 (d, $^{2}J_{C\text{-}C}$) = 5.5 Hz), 46.5 ($^{13}CH_2$), 34.6, 31.3, 29.8, 29.3, 27.3, 27.1, 24.8, 24.1, 23.9, 23.1, 21.9.

Synthesis of **^{13}C-1A^{iPr}**

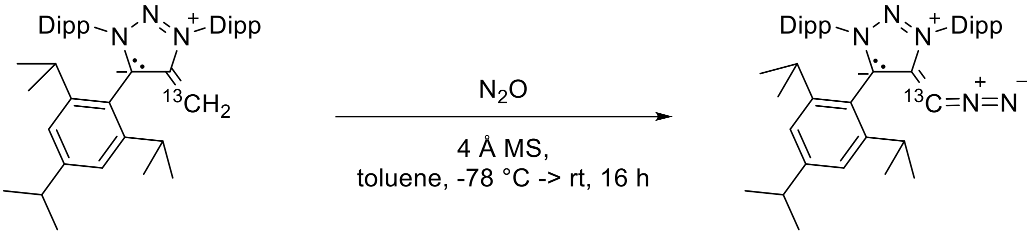

Under an atmosphere of argon, **^{13}C-Tripp-mNHO** (350 mg, 0.59 mmol, 1.0 eq) was dissolved in Et_2O (40 mL) and 300 mg thoroughly activated 4 Å molecular sieves were added. The solution was cooled to –78 °C and the inert gas was exchanged for 1 atm. N_2O, by evacuating the pressure Schlenk flask until gas evolution from the solution ceased from the solution at -78°C and repressurizing it with N_2O (1 bar). The flask was closed and warmed up to room temperature (Caution: the actual pressure in the flask at room temperature is higher approximately 2-3 bar; use pressure glassware). Stirring the solution for 16 h led to a change of color from dark violet over brown to bright orange. The solution was filtered off and the remaining residue was washed four times with toluene (4 x 20 mL). The solvent was removed under reduced pressure furnishing an orange solid. The crude product was washed twice with pentane (2 x 5 mL). Drying under reduced pressure afforded the desired diazoalkene (186 mg, 0.29 mmol, 49%) as a yellow solid.

^{1}H NMR (700 MHz, C_6D_6, 298 K): δ [ppm] = 7.30 (t, *J* = 7.8 Hz, 1 H), 7.08 (s, 2 H), 7.03 (t, *J* = 7.9 Hz, 1 H), 6.90 (d, *J* = 8.0 Hz, 2 H), 3.10 – 3.02 (m, 4 H), 2.77 – 2.68 (m, 3 H), 1.55 (d, *J* = 6.7 Hz, 6 H), 1.52 (d, *J* = 6.9 Hz, 6 H), 1.23 (d, *J* = 6.9 Hz, 6 H), 1.13 (d, *J* = 6.8 Hz, 6 H), 1.08 (d, *J* = 6.8 Hz, 6 H), 0.97 (d, *J* = 6.7 Hz, 6 H), 0.87 (d, *J* = 6.7 Hz, 6 H); **^{13}C NMR** (176 MHz, C_6D_6, 298 K): δ [ppm] = 153.7 (d, $^{1}J_{C\text{-}C}$ = 62 Hz), 151.4, 150.2, 147.3, 145.7, 132.8, 132.5 (d, $^{2}J_{C\text{-}C}$ = 6 Hz), 132.1, 131.5, 131.4, 125.0, 124.4, 122.4, 120.9, 34.6, 34.4 ($^{13}C{=}N_2$), 31.9, 29.9, 29.8, 26.6, 26.2, 24.3, 24.1, 23.9, 23.1, 21.7.

Synthesis of **^{13}C-IPr=CH2**

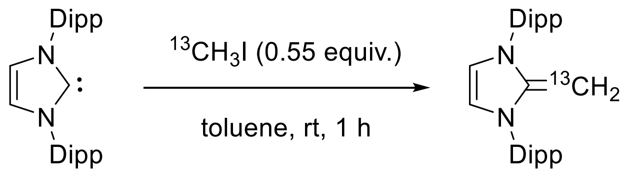

1,3-Bis(2,6-diisopropylphenyl)imidazol-2-ylidene (IPr) was synthesized following a literature procedure.[16] **^{13}C-IPr=CH$_2$** was prepared according to a modified literature procedure.[17]

Under an atmosphere of argon, IPr carbene (0.63 g, 1.62 mmol, 1.0 equiv.) was dissolved in toluene (15 mL) and $^{13}CH_3I$ (56.0 μmol, 0.89 mmol, 0.55 equiv.) added, which resulted in the formation of a cloudy suspension. The solution was stirred for 1 h at ambient temperature. The reaction mixture was filtered over celite and the solvent of the filtrate removed under reduced pressure to afford the corresponding NHO (254 mg, 0.63 mmol, 71%) as a colorless solid.

^{1}H NMR (500 MHz, C_6D_6, 298 K): δ [ppm] = 7.18 (m, 2 H), 7.10 (d, 4 H, J = 7.1 Hz), 5.80 (s, 2 H), 3.30 (septet, J = 6.9 Hz, 4 H), 2.36 (d, 2 H, $J_{H\text{-}C}$ = 161.6 Hz, $^{13}CH_2$), 1.31 (d, 12 H, J = 6.9 Hz), 1.17 (d, 1 2H, J = 6.9 Hz); **^{13}C NMR** (126 MHz, C_6D_6, 298 K): δ [ppm] = 152.5 ($^1J_{C\text{-}C}$ = 87.3 Hz), 149.0, 135.0, 129.3, 124.6, 114.7, 44.4 ($^{13}CH_2$), 28.8, 24.4, 23.9.

Synthesis of **^{13}C-1E**

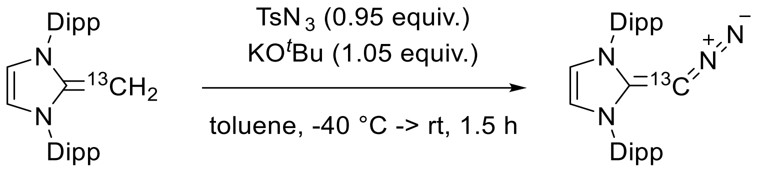

Under an atmosphere of argon, **^{13}C-IPr=CH$_2$** (250 mg, 0.62 mmol, 1.0 equiv.) and KO^{t}Bu (73.0 mg, 0.65 mmol, 1.05 equiv.) were dissolved in THF (15 mL) and the solution was cooled to –40 °C. *p*-tosylazide (0.38 mL, 34% in toluene, 0.59 mmol, 0.95 equiv.) was dissolved in a second Schlenk flask (10 mL) and cooled to -78 °C. The solution of the azide was added dropwise over 15 min to the solution of the NHO. The reaction mixture was stirred at that temperature for 30 min and then warmed up to room temperature under stirring for 1 h. The solvent was removed under reduced pressure and the residue extracted with a solvent mixture of THF:Et_2O (1:10, 2 x 20 mL) and filtered over celite. The solvent was removed under reduced pressure and the solid washed with pentane (5 mL) and dried under reduced pressure to afford **^{13}C-1E** (186 mg, 0.43 mmol, 70 %) as a light-brownish powder.

^{1}H NMR (500 MHz, C_6D_6, 298 K): δ [ppm] = 7.25 (t, J = 7.7 Hz, 1 H), 7.11 (d, J = 7.7 Hz, 2 H), 5.92 (d, J = 0.8 Hz, 1 H), 2.98 (hept, J = 6.9 Hz, 2 H), 1.42 (d, J = 6.9 Hz, 6 H), 1.15 (d, J = 6.9 Hz, 6 H), **^{13}C NMR** (126 MHz, C_6D_6, 298 K): δ [ppm] = 152.1 (d, $^1J_{C\text{-}C}$ = 66.9 Hz), 147.4, 133.2, 130.6, 124.2, 117.9, 117.9, 35.2 ($^{13}CN_2$), 29.2, 24.1, 23.8.

1.4. Irradiation Experiments

Overview of triplet vinylidene generation and their C–H activation products

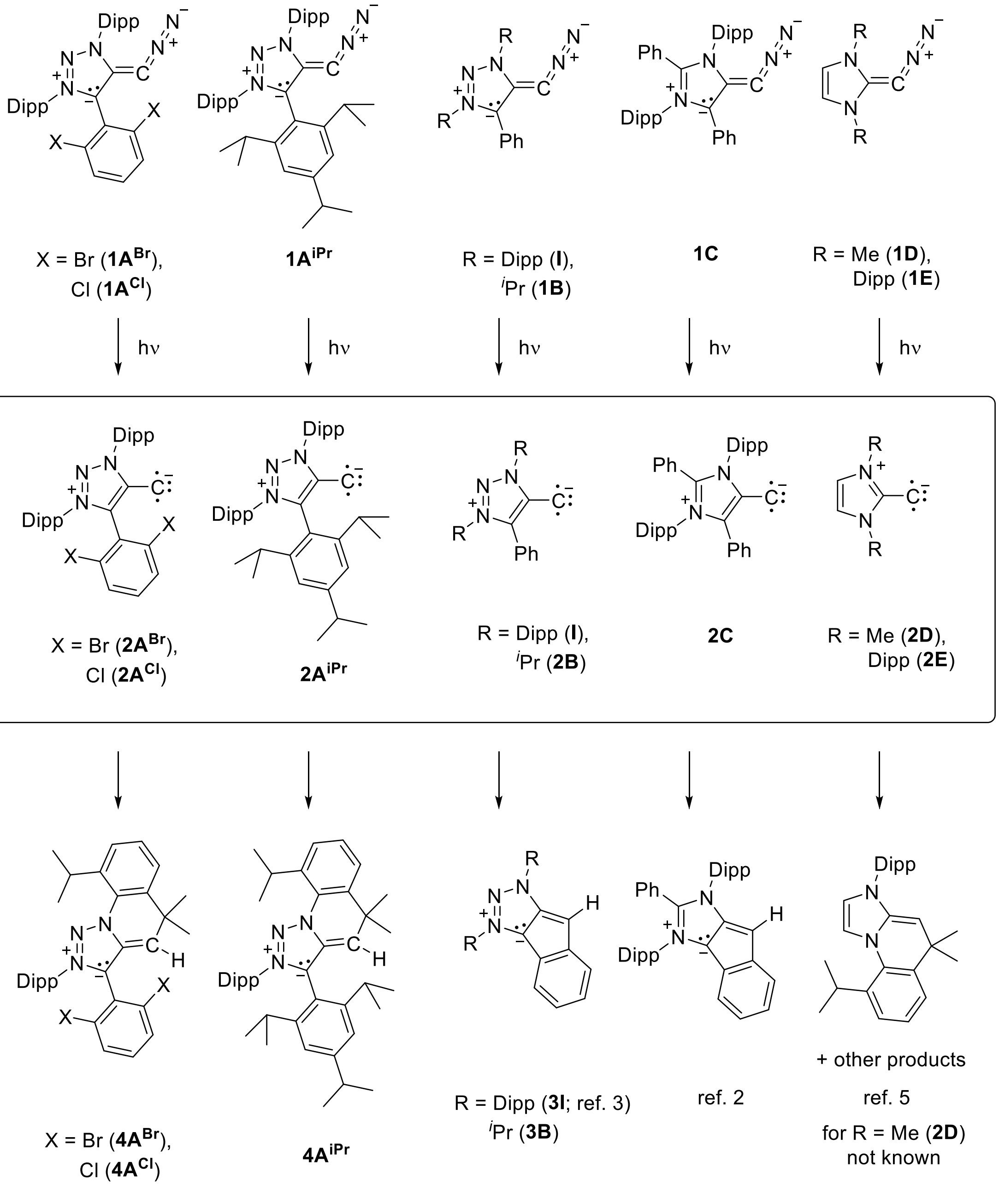

Figure S1.3 Synthesis of triplet vinylidenes and their C–H activation products.

For the *in situ* irradiation of halogenated diazoalkenes **1A^{Cl}** and **1A^{Br}** the respective compounds were dissolved in a J-Young NMR tube in C_6D_6 as described in the experimental section. While the chloro-substituted diazoalkene (**1A^{Cl}**) afforded upon irradiation C-H insertion product quantitatively (Figure S1.4), the bromo-substituted diazoalkene (**1A^{Br}**) produced minor side products (Figure S1.5).

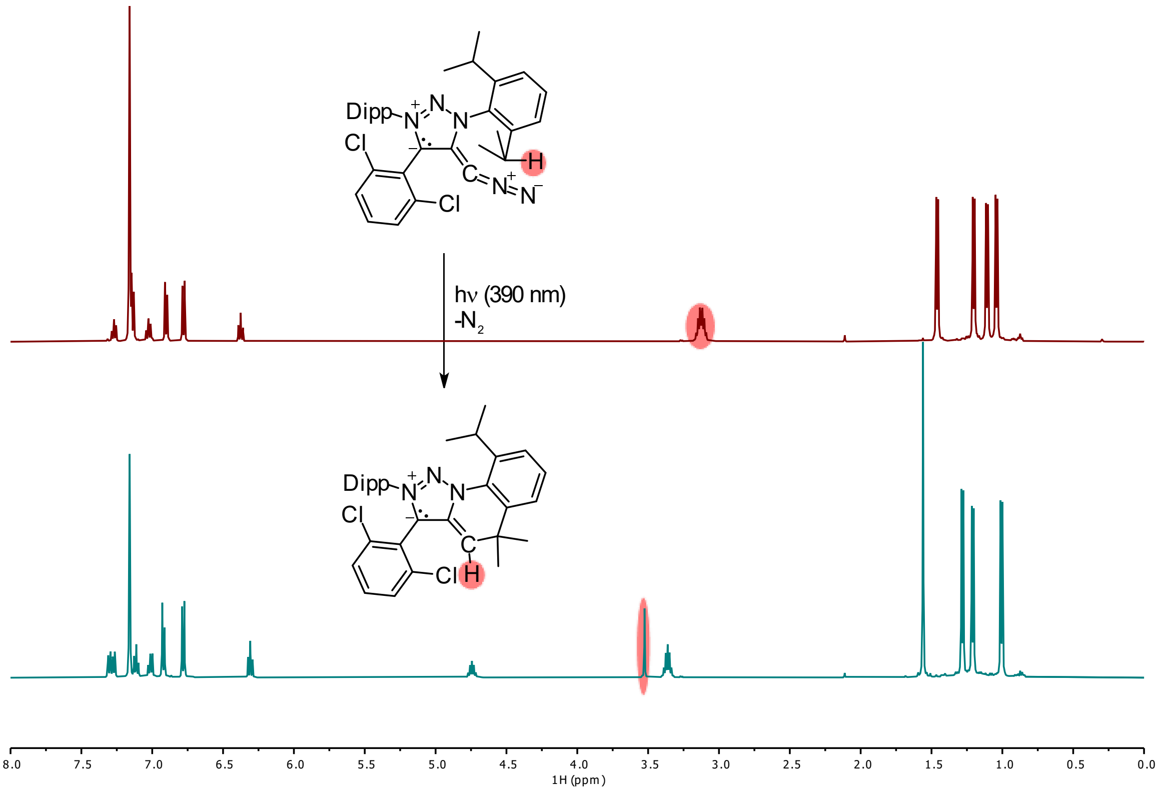

Figure S1.4. Stacked **^{1}H NMR** (501 MHz, C_6D_6, 298 K) spectra of chloro-diazoalkene **1A^{Cl}** before irradiation (top) and after irradiation for one hour at 390 nm (bottom).

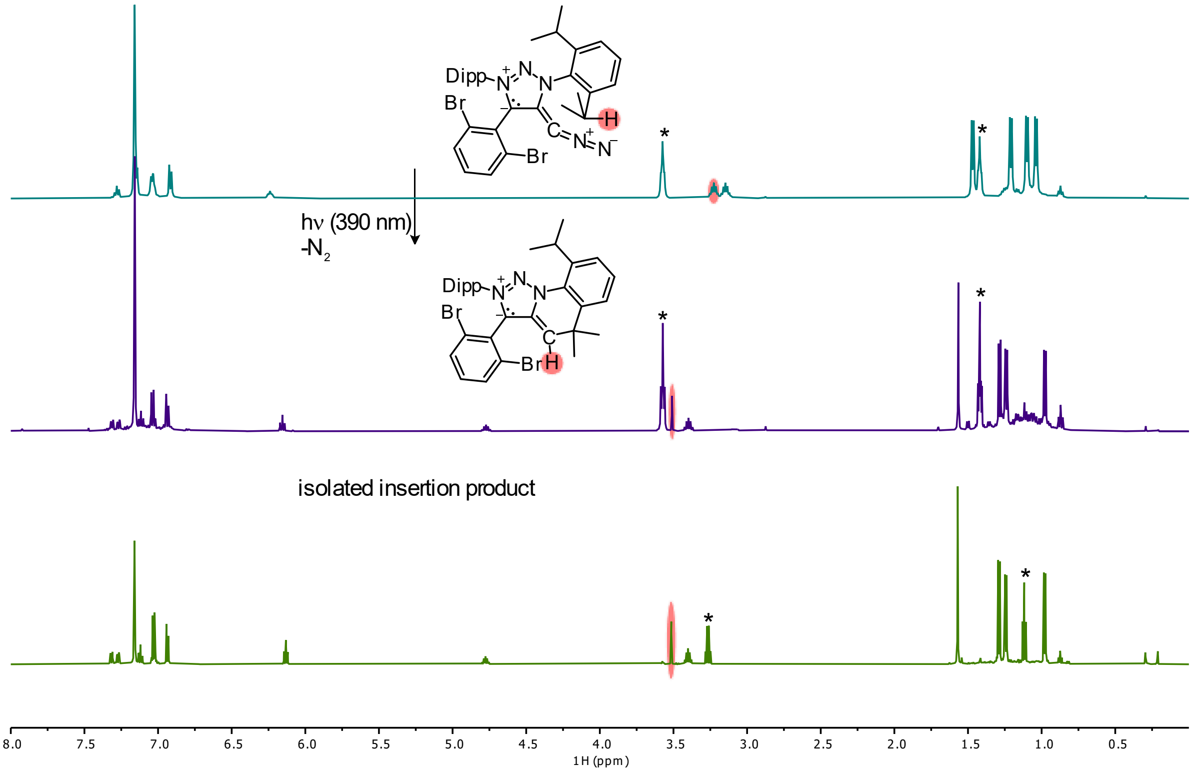

Figure S1.5**.** Stacked **^{1}H NMR** (501 MHz, C_6D_6, 298 K) spectra of bromo-diazoalkene **1A^{Br}** before irradiation (top) and after irradiation for 50 min (middle) and isolated product spectrum (bottom).

The *in situ* formation of insertion product **3B** was monitored by ^{1}H and ^{13}C NMR. Diazoalkene **1B** (~15 mg) was dissolved in 0.5 mL C_6D_6 in a J-Young NMR tube. The tube was placed with ~15 cm distance from a Kessil LED (390 nm) and irradiated for 40 min.

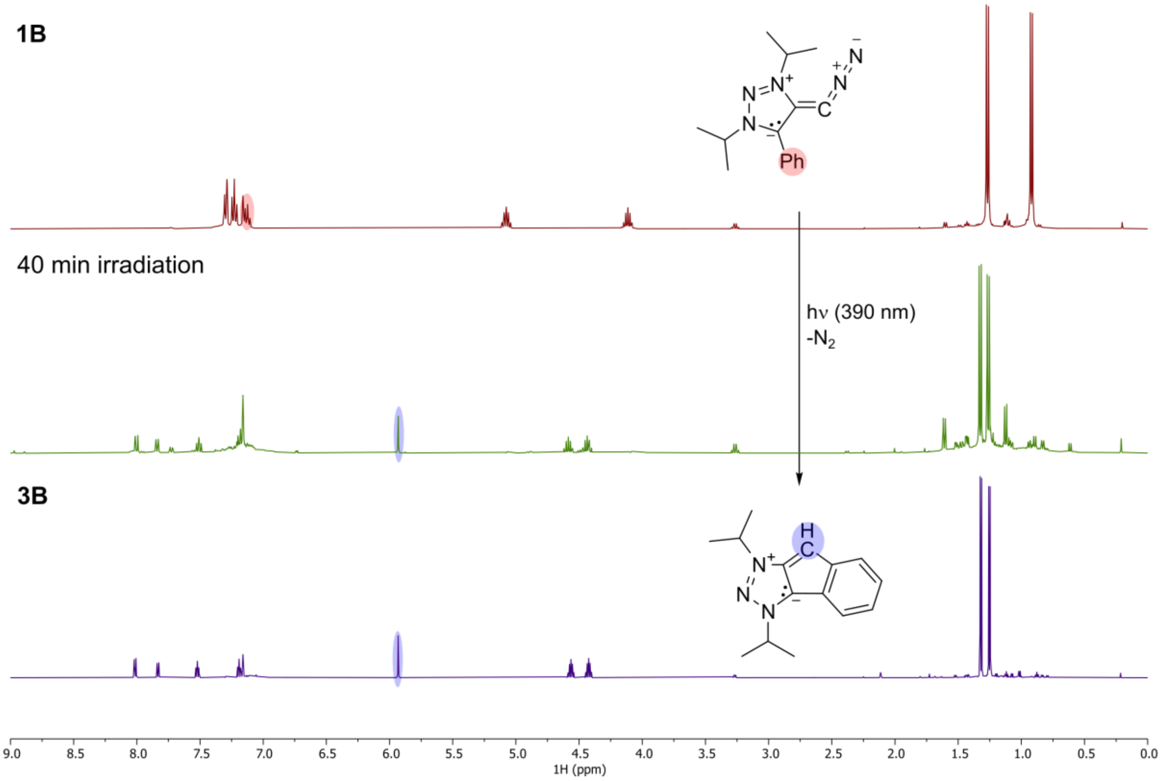

Figure S1.6. Stacked ^{1}**H NMR** spectra of the irradiation of diazoalkene **1B**. Top: ^{1}**H NMR** (400 MHz, C_6D_6, 298 K) of isolated **1B**. Middle: ^{1}**H NMR** (400 MHz, C_6D_6, 298 K) after 40 min of irradiation. Bottom: ^{1}**H NMR** (600 MHz, C_6D_6, 298 K) of isolated **3B**.

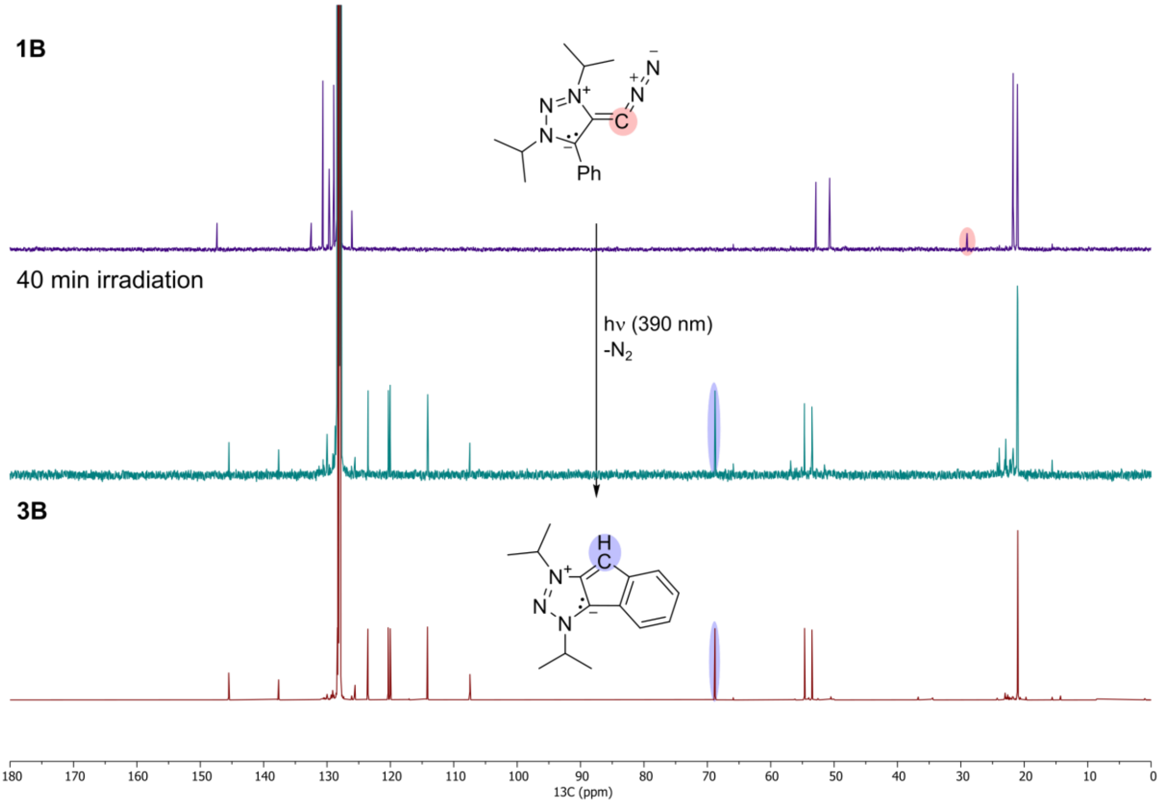

Figure S1.7. Stacked ^{13}C NMR spectra of the irradiation of diazoalkene **1B**. Top: ^{13}**C NMR** (400 MHz, C_6D_6, 298 K) of isolated **1B**. Middle: ^{13}**C NMR** (400 MHz, C_6D_6, 298 K) after 40 min of irradiation. Bottom: ^{13}**C NMR** (600 MHz, C_6D_6, 298 K) of isolated **3B**.

Synthesis of **4A^{Cl}**

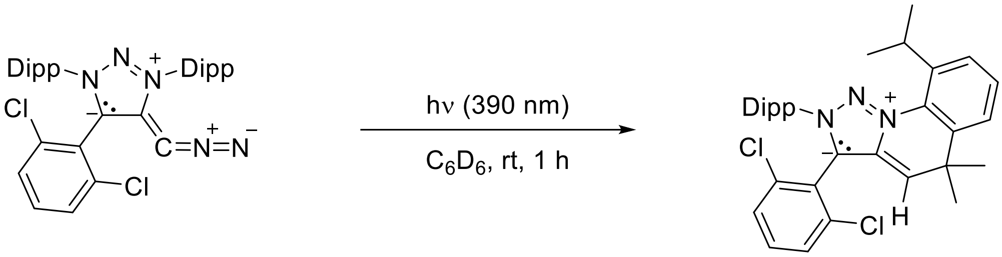

Under an atmosphere of nitrogen, diazoalkene **1A^{Cl}** (13.6 mg, 23.7 µmol, 1.0 eq.) was dissolved in C_6D_6 (1 mL) in a J Young NMR tube. The tube was placed in a water bath and the solution irradiated with a 390 nm Kessil lamp under which the solution turned from orange to dark purple. The solvent was evaporated to afford **4A^{Cl}** (12.8 mg, 23.4 µmol, quant.) as a dark purple solid.

m.p. 98 °C; ^{1}**H-NMR** (501 MHz, C_6D_6, 298 K): δ [ppm] = 7.30 (dd, *J* = 7.7, 1.6 Hz, 1 H, Ar-CH)7.27 (dd, *J* = 7.7, 1.6 Hz, 1 H, Ar-CH), 7.11 (t, *J* = 7.7 Hz, 1 H, Ar-CH), 7.01 (dd, *J* = 8.3, 7.1 Hz, 1 H, Dipp-CH), 6.92 (d, *J* = 7.8 Hz, 2 H, Dipp-CH), 6.78 (d, *J* = 8.1 Hz, 2 H, Ar^{Cl}-H),

6.31 (t, *J* = 8.1 Hz, 1 H, Ar^{Cl}-H), 4.74 (hept, *J* = 7.0 Hz, 1 H, C*H*(CH_3)$_2$), 3.53 (s, 1 H, olefin-CH), 3.36 (hept, *J* = 6.7 Hz, 2 H, C*H*(CH_3)$_2$), 1.56 (s, 6 H, C(C*H*$_3$)$_2$), 1.28 (d, *J* = 6.7 Hz, 6 H, CH(C*H*$_3$)$_2$), 1.21 (d, *J* = 6.7 Hz, 6 H, CH(C*H*$_3$)$_2$), 1.01 (d, *J* = 6.7 Hz, 6 H, CH(C*H*$_3$)$_2$); **^{13}C NMR** (126 MHz, C_6D_6, 298 K): *δ* [ppm] = 146.8 (Dipp-C_q), 137.9 (triaz-C_q), 137.7 (Ar^{Cl}-C_q), 135.9 (Ar-C_q), 132.8 (Ar-C_q), 132.6 (Ar-C_q), 132.6 (Dipp-C_q), 131.0 (Dipp-CH), 130.0 (Ar^{Cl}-CH), 128.9 (Ar^{Cl}-CH), 126.6 (Ar^{Cl}-C_q), 125. (Ar-CH), 125.1 (Ar-CH), 124.8 (Ar-CH), 124.2 (Dipp-CH), 115.3 (triaz-C_q), 75.1 (olefin-CH), 36.0 (*C*$_q$-(CH_3)$_2$), 35.6 (C_q-(*C*H$_3$)$_2$), 29.2 (*C*H(CH_3)$_2$), 29.0 (*C*H(CH_3)$_2$), 27.1 (CH(*C*H$_3$)$_2$), 24.5 (CH(*C*H$_3$)$_2$), 22.0 (CH(*C*H$_3$)$_2$); **IR** [cm^{-1}]: 2963, 2867, 1653, 1552, 1490, 1466, 1426, 1383, 1350, 1256, 1240, 1135, 1105, 1056, 1000, 978, 956, 935, 803, 790, 776, 762, 741, 683, 623, 547, 498; **HR-MS-ESI(+)** calc. $C_{33}H_{38}Cl_2N_3^+$ $[M+H]^+$ calc. 546.2437; found 546.2419.

Synthesis of **4A^{Br}**

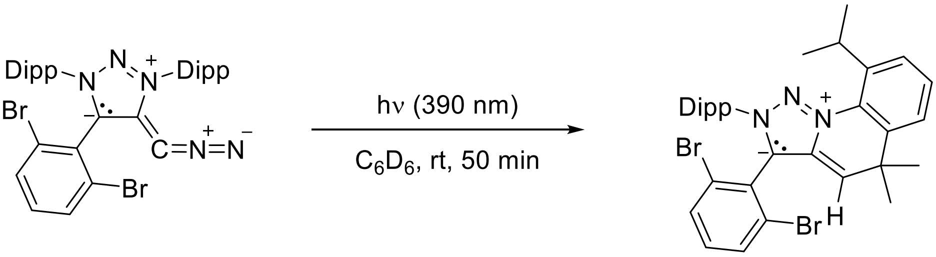

Under an atmosphere of nitrogen, diazoalkene **1A^{Br}** (11.5 mg, 17.3 μmol, 1.0 eq.) was dissolved in C_6D_6 (0.5 mL) in a J Young NMR tube. The tube was placed in a water bath and the solution irradiated with a 390 nm Kessil lamp under which the solution turned from orange to dark purple. The solvent was evaporated and the residue extracted with pentane (2 x 1 mL) and filtered over celite. The solution was stored at -40 °C overnight upon which a brown precipitate formed. The precipitate was filtered off. The solvent of the filtrate was removed under reduced pressure to afford **4A^{Br}** (5.30 mg, 8.34 mmol, 49%) as a dark blue-purple solid.

m.p. 88 °C; **^{1}H-NMR** (600 MHz, C_6D_6, 298 K): δ [ppm] = 7.32 (dd, *J* = 7.8, 1.6 Hz, 1 H, Ar-CH), 7.27 (dd, *J* = 7.8, 1.5 Hz, 1 H, Ar-CH), 7.12 (t, *J* = 7.7 Hz, 1 H, Ar-CH), 7.05 – 7.01 (m, 3 H, Dipp-CH and Ar^{Br}-H), 6.94 (d, *J* = 7.7 Hz, 2 H, Dipp-CH), 6.13 (t, *J* = 8.0 Hz, 1 H, Ar^{Br}-H), 4.78 (hept, *J* = 6.7 Hz, 1 H, C*H*(CH_3)$_2$), 3.51 (s, 1 H, olefin-CH), 3.40 (hept, *J* = 6.7 Hz, 2 H, C*H*(CH_3)$_2$), 1.57 (s, 6 H, C(C*H*$_3$)$_2$), 1.29 (d, *J* = 6.8 Hz, 6 H, CH(C*H*$_3$)$_2$), 1.24 (d, *J* = 6.7 Hz, 6 H, CH(C*H*$_3$)$_2$), 0.98 (d, *J* = 6.7 Hz, 6 H, CH(C*H*$_3$)$_2$); **^{13}C NMR** (151 MHz, C_6D_6, 298 K): *δ* [ppm] = 146.8 (Dipp-C_q), 137.7 (triaz-C_q), 135.9 (Ar-C_q), 133.0 (Ar^{Br}-CH), 132.9 (Ar-C_q), 132.8 (Dipp-C_q), 132.6 (Ar-C_q), 131.0 (Dipp-CH), 130.6 (Ar^{Br}-CH), 130.1 (Dipp-C_q), 128.0 (Ar^{Br}-C_q), 125.4 (Ar-CH), 125.0 (Ar-CH), 124.8 (Ar-CH), 124.4 (Dipp-CH), 118.6 (triaz-C_q), 75.4 (olefin-CH), 36.0 (*C*$_q$-(CH_3)$_2$), 35.4 (C_q-(*C*H$_3$)$_2$), 29.4 (*C*H(CH_3)$_2$), 28.9 (*C*H(CH_3)$_2$), 27.1 (CH(*C*H$_3$)$_2$), 24.4 (CH(*C*H$_3$)$_2$), 22.1 (CH(*C*H$_3$)$_2$); **IR** [cm^{-1}]: 2959, 2923, 2852, 1772, 1672, 1587, 1549, 1460,

1386, 1364, 1258, 1167, 1066, 800, 752, 729, 699, 658, 621; **HR-MS-ESI(+)** calc. $C_{33}H_{38}Br_2N_3^+$ $[M+H]^+$ calc. 634.1427; found 634.1394.

Synthesis of **4A^{iPr}**

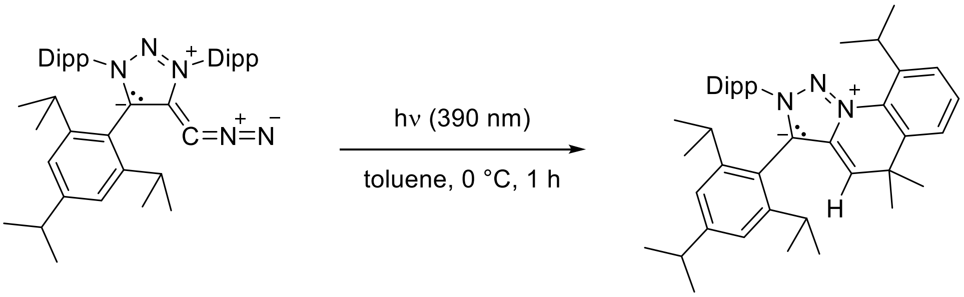

Irradiation of a solution of **1A^{iPr}** (100 mg, 0.16 mmol, 1.0 eq.) in toluene (10 mL) under argon atmosphere at 0 °C with a 390 nm Kessil lamp (10 cm distance) for 1 h led to a change of color from orange to dark purple. The solvent was removed under reduced pressure and the resulting solid was extracted with pentane (2 x 20 ml). The solvent was again removed under reduced pressure before the crude product was recrystallized twice from HMDSO (10 mL, -40 °C), furnishing **4A^{iPr}** (36 mg, 59 µmol, 38%) as a dark purple crystalline solid.

m.p. 102 °C; **^{1}H NMR** (500 MHz, C_6D_6, 298 K): δ [ppm] = 7.31 (dd, J = 7.7, 1.5 Hz, 1H, Ar-H), 7.24 (dd, J = 7.8, 1.5 Hz, 1H, Ar-H), 7.11 (t, J = 7.7 Hz, 1H, Ar-H), 7.05 (s, 2H, Tripp-Ar-H), 7.03 (t, J = 7.2 Hz, 1H, Ar-H), 6.92 (d, J = 0.7 Hz, 1H, Ar-H), 4.86 (hept, J = 6.8 Hz, 1H, $CH(C\mathit{H}_3)_2$), 3.53 (s, 1H, alkene-H), 3.14 (hept, J = 6.7 Hz, 2H, $C\mathit{H}(CH_3)_2$), 3.04 (hept, J = 6.7 Hz, 2H, $C\mathit{H}(CH_3)_2$), 2.71 (hept, J = 6.8 Hz, 1H, $C\mathit{H}(CH_3)_2$), 1.51 (s, 6H, $(C\mathit{H}_3)_2$), 1.36 (d, J = 6.7 Hz, 6H, $CH(C\mathit{H}_3)_2$), 1.29 (d, J = 6.7 Hz, 6H, $CH(C\mathit{H}_3)_2$), 1.26 (d, J = 6.8 Hz, 6H, $CH(C\mathit{H}_3)_2$), 1.15 (d, J = 6.9 Hz, 6H, $CH(C\mathit{H}_3)_2$), 0.90 (d, J = 6.8 Hz, 6H, $CH(C\mathit{H}_3)_2$), 0.82 (d, J = 6.7 Hz, 6H, $CH(C\mathit{H}_3)_2$); **^{13}C NMR** (126 MHz, C_6D_6, 298 K): δ [ppm] = 150.6 (Ar-C_q), 150.2 (Ar-C_q), 145.8 (Ar-C_q), 142.0 (triaz-C_q), 135.8 (Ar-C_q), 134.0 (Ar-C_q), 132.9 (Ar-C_q), 132.6 (triaz-C_q), 130.4 (Ar-CH), 125.1 (Ar-CH), 124.8 (Ar-CH), 124.8 (Ar-CH), 124.2 (Ar-CH), 122.9 (Ar-C_q), 122.5 (Ar-CH), 118.6 (Ar-C_q), 75.7 (Alkene-CH), 36.0 ($\mathit{C}(CH_3)_2$), 34.6 ($\mathit{C}H(CH_3)_2$), 34.2 ($C(\mathit{C}H_3)_2$), 31.3 ($\mathit{C}H(CH_3)_2$), 29.7 ($\mathit{C}H(CH_3)_2$), 28.7 ($\mathit{C}H(CH_3)_2$), 27.2 ($CH(\mathit{C}H_3)_2$), 26.7 ($CH(\mathit{C}H_3)_2$), 24.4 ($CH(\mathit{C}H_3)_2$), 24.1 ($CH(\mathit{C}H_3)_2$), 23.0 ($CH(\mathit{C}H_3)_2$), 21.8 ($CH(\mathit{C}H_3)_2$); **IR** [cm^{-1}]: ṽ = 2866, 1631, 1459, 1427, 1383, 1346, 1230, 1137, 1100, 1053, 998, 977, 935, 880, 842, 803, 785, 756, 738, 694, 647, 489; **HR-MS-ESI(+)** calc. $C_{42}H_{57}N_3^+$ $[M+H]^+$ 604.4625; found 604.4626.

Synthesis of 5

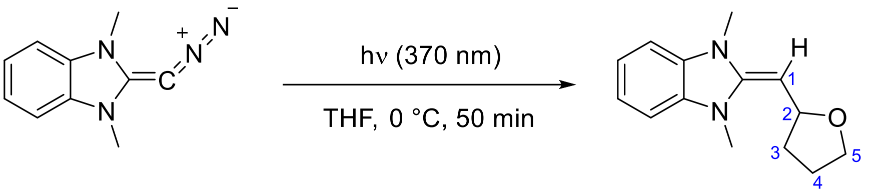

1F (20.0 mg, 107 µmol, 1.0 eq.) was dissolved in THF (0.5 mL) in a J-Young NMR tube and cooled to 0 °C. The solution was irradiated with a 370 nm Kessil lamp (50 W) for 50 minutes. The solvent was removed under reduced pressure. The residual brown mixture was extracted with pentane (3 x 1 mL) and collected with a glass pipette. The solvent was again removed under reduced pressure to afford **5** (17.1 mg, 74 µmol, 69%) as a light yellow oil.

^{1}H NMR (500 MHz, C_6D_6, 298 K): δ [ppm] = 6.87 – 6.79 (m, 2H, $Ar^{benzimi}$-CH), 6.37 – 6.31 (m, 1H, $Ar^{benzimi}$-CH), 6.30 – 6.26 (m, 1H, $Ar^{benzimi}$-CH), 4.86 – 4.77 (m, 1H, H2), 4.04 – 3.97 (m, 1H, H5), 3.77 – 3.69 (m, 1H, H5), 3.44 (d, *J* = 8.4 Hz, 1H, H1), 3.27 (s, 3H, N-CH_3), 2.51 (s, 3H, N-CH_3), 2.11 – 2.03 (m, 1H, H3), 1.87 – 1.77 (m, 1H, H4), 1.75 – 1.66 (m, 1H, H4), 1.66 – 1.57 (m, 1H, H3); **^{13}C NMR** (126 MHz, C_6D_6, 298 K): δ [ppm] = 150.0 (N-C-N), 136.8 ($Ar^{benzimi}$-C_q), 135.6 ($Ar^{benzimi}$-C_q), 119.9 ($Ar^{benzimi}$-CH), 119.2 ($Ar^{benzimi}$-CH), 104.9 ($Ar^{benzimi}$-CH), 104.0 ($Ar^{benzimi}$-CH), 75.9 (C2), 68.4 (C1), 66.8 (C5), 35.4 (C3), 32.2 (N-CH_3), 28.5 (N-CH_3), 26.9 (C4); **IR** [cm^{-1}]: ṽ = 3055, 2962, 2864, 1628, 1607, 1502, 1448, 1391, 1296, 1247, 1225, 1139, 1014, 909, 838, 722, 683, 618, 563, 528, 500, 430; **HR-MS-ESI(+)** calc. $C_{14}H_{19}N_2O^+$ $[M+H]^+$ calc. 231.1492; found 231.1495.

Synthesis of 5-d$_8$

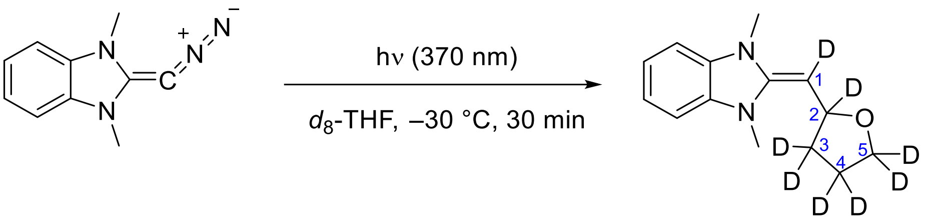

1F (15.5 mg, 83 µmol, 1.0 eq.) was dissolved in *d*$_8$-THF (0.4 mL) in a J. Young NMR tube and cooled to –30 °C. The solution was irradiated with a 370 nm Kessil lamp (50 W) for 30 minutes. The solvent was removed under reduced pressure. The residual brown mixture was extracted with pentane (3 x 1 mL) and collected with glass pipette. The solvent was again removed under reduced pressure to afford **5-d$_8$** (9.8 mg, 41 µmol, 49%) as an amber oil.

^{1}H NMR (500 MHz, C_6D_6, 298 K): δ [ppm] = 6.87 – 6.79 (m, 2H, $Ar^{benzimi}$-CH), 6.37 – 6.26 (m, 2H, $Ar^{benzimi}$-CH), 3.27 (s, 3H, N-CH_3), 2.51 (s, 3H, N-CH_3); **^{13}C NMR** (151 MHz, C_6D_6, 298 K): δ [ppm] = 150.0 (N-C-N), 136.8 ($Ar^{benzimi}$-C_q), 135.6 ($Ar^{benzimi}$-C_q), 119.9 ($Ar^{benzimi}$-CH), 119.2

(Ar^{benzimi}-CH), 104.9 (Ar^{benzimi}-CH), 104.0 (Ar^{benzimi}-CH), 75.3 (t, J = 21.7 Hz, C2), 68.0 (t, J = 23.8 Hz, C1), 65.9 (quintet, J = 21.8 Hz, C5), 34.3 (quintet, J = 19.7 Hz, C3), 32.2 (N-CH_3), 28.5 (N-CH_3), 25.8 (quintet, J = 19.8 Hz, C4); **^{2}H NMR** (500 MHz, toluene, 298 K): δ [ppm] = 4.69, 3.86, 3.59, 3.32, 1.95, 1.71, 1.60, 1.47; **IR** [cm^{-1}]: ṽ = 3056, 2929, 1709, 1603, 1501, 1444, 1388, 1324, 1215, 1157, 1135, 1015, 884, 810, 723, 558, 499, 430; **HR-MS-ESI(+)** calc. $C_{14}H_{11}D_8N_2O^+$ $[M+H]^+$ calc. 239.1994; found 239.1978.

***In situ* Irradiation of ^{13}C-1A^{iPr}**

^{13}C-labeled diazoalkene **^{13}C-1A^{iPr}** was irradiated in a J-Young NMR tube to confirm the connectivity of the insertion product **4A^{iPr}**. For this, **^{13}C-1A^{iPr}** (15 mg) was dissolved in C_6D_6 (0.5 mL) and the NMR tube placed in a cooling bath. The solution was irradiated for the respective times with a 390 nm Kessil LED and ^{13}C NMR spectra measured to observe the process (Figure S1.8).

The comparison of the NMR spectra of isolated insertion product **4A^{iPr}** with the *in situ* generated NMR spectra of **^{13}C-1A^{iPr}** after 1 h of irradiation show the clean formation of the C−H activation product (Figure S1.9 and Figure S1.10).

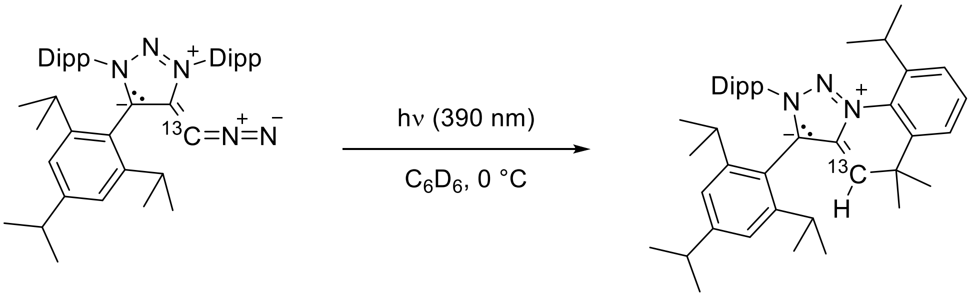

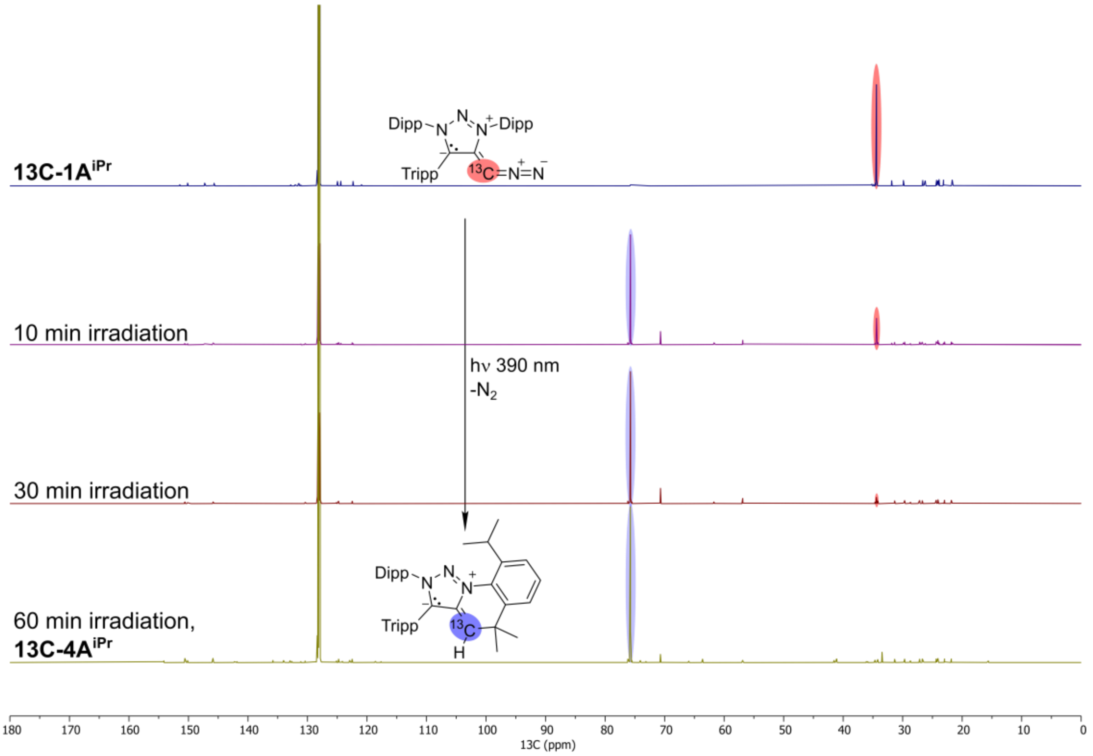

Figure S1.8. Stacked **^{13}C NMR** spectra of ^{13}C-labeled diazoalkene irradiation. 1st: (700 MHz, C_6D_6, 298 K) of isolated **^{13}C-1A^{iPr}**; 2nd: (500 MHz, C_6D_6, 298 K) after 10 min of irradiation; 3rd: (500 MHz, C_6D_6, 298 K) after 30 min of irradiation; 4th: (700 MHz, C_6D_6, 298 K) after 60 minutes of irradiation.

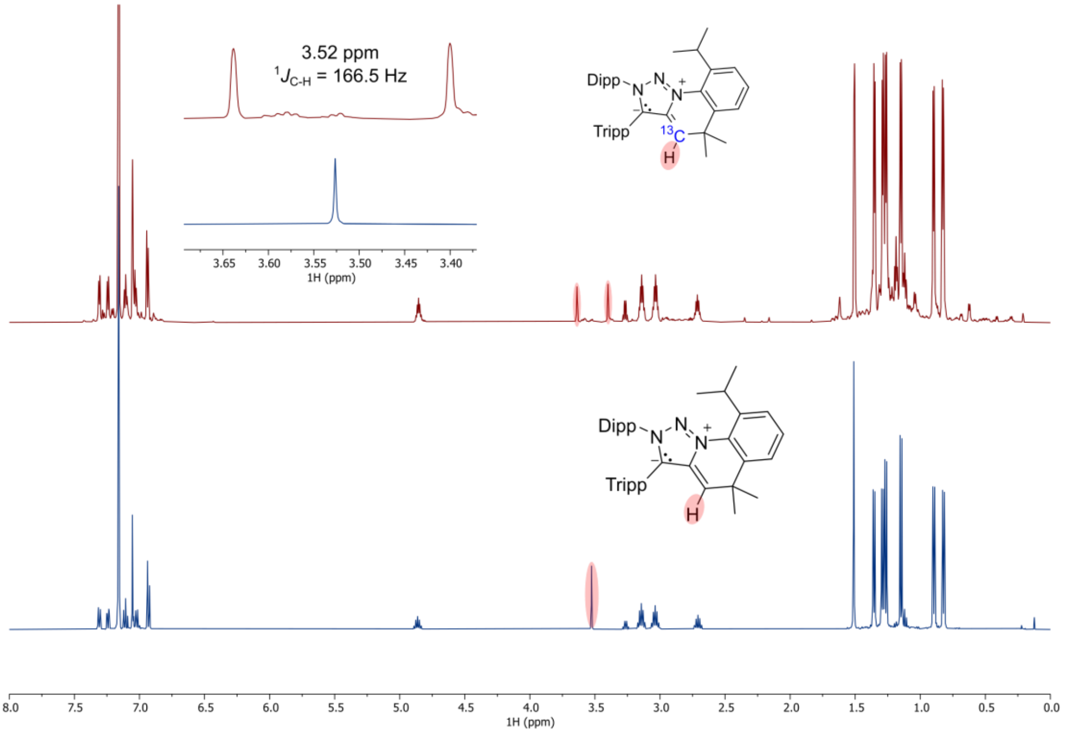

Figure S1.9. Stacked **^{1}H NMR** spectra of **^{13}C-4A^{iPr}** (top, 700 MHz, C_6D_6, 298 K) and **4A^{iPr}** (bottom, 500 MHz, C_6D_6, 298 K).

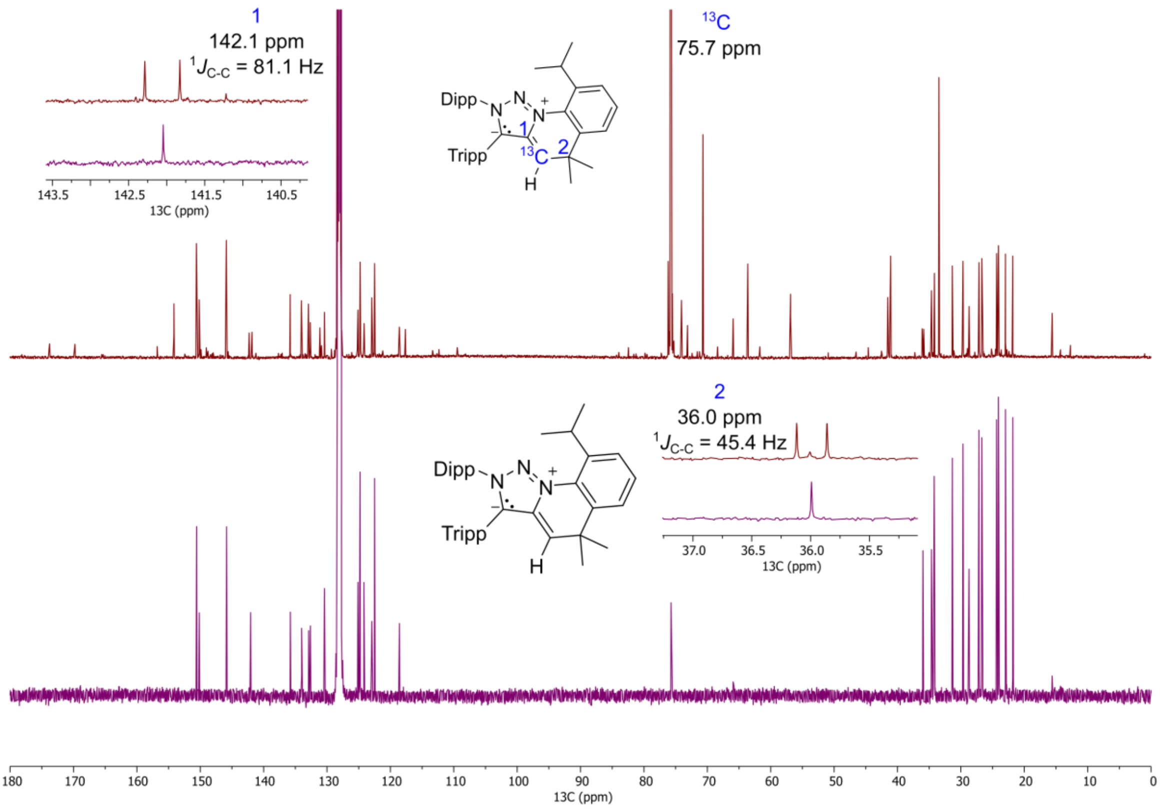

Figure S1.10. Stacked **^{13}C NMR** spectra of **^{13}C-4A^{iPr}** (top, 176 MHz, C_6D_6, 298 K) and **4A^{iPr}** (bottom, 126 MHz, C_6D_6, 298 K).

Synthesis of **3B**

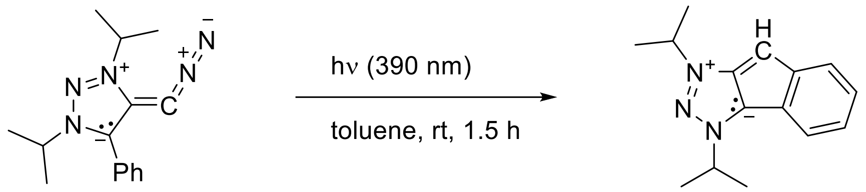

Under an atmosphere of argon, diazoalkene **1B** (50 mg, 0.19 mmol, 1.0 equiv.) was dissolved in toluene (5 mL) and the Schlenk tube was placed in a water bath at room temperature. The solution of the diazoalkene was irradiated with a 390 nm Kessil lamp in a distance of ca. 20 cm for 1.5 h, while the color changed from bright yellow-orange to dark purple. The solvent was removed under reduced pressure, the residue extracted with pentane (2 x 3 mL) and filtered over celite. The solvent was again removed under reduced pressure to afford insertion product **3B** (41 mg, 0.17 mmol, 92 %) as a bright red solid.

m.p. 165 °C; **^{1}H-NMR** (600 MHz, C_6D_6, 298 K): δ [ppm] = 8.01 (dt, J = 8.3, 1.0 Hz, 1 H, Ar-CH), 7.83 (dq, J = 8.0, 0.9 Hz, 1 H, Ar-CH), 7.52 (ddd, J = 8.1, 6.8, 1.1 Hz, 1 H, Ar-CH), 7.19 (ddd, J = 7.9, 6.8, 1.0 Hz, 1 H, Ar-CH), 5.93 (d, J = 0.7 Hz, 1 H, olefin-CH), 4.56 (hept, J = 6.8

Hz, 1 H, $CH(CH_3)_2$), 4.42 (hept, J = 6.7 Hz, 1 H, $CH(CH_3)_2$), 1.32 (d, J = 6.8 Hz, 6 H, $CH(CH_3)_2$), 1.25 (d, J = 6.8 Hz, 6 H, $CH(CH_3)_2$).; **^{13}C NMR** (151 MHz, C_6D_6, 298 K): δ [ppm] = 145.5 (Ar-C_q), 137.6 (triaz-C_q), 125.6 (triaz-C_q), 123.6 (Ar-CH), 120.4 (Ar-CH), 120.0 (Ar-CH), 114.1 (Ar-CH), 107.5 (Ar-C_q), 68.8 (olefin-CH), 54.7 ($CH(CH_3)_2$), 53.5 ($CH(CH_3)_2$), 21.1 ($CH(CH_3)_2$), 21.0 ($CH(CH_3)_2$); **IR** [cm^{-1}]: 3059, 2979, 2934, 2871, 1611, 1553, 1531, 1497, 1456, 1441, 1426, 1388, 1369, 1343, 1323, 1272, 1252, 1240, 1195, 1162, 1144, 1130, 1112, 1079, 1054, 1010, 998, 921, 885, 835, 813, 771, 733, 697, 680, 619, 562, 550, 501, 433; **HR-MS-ESI(+)** calc. $C_{15}H_{20}N_3^+$ $[M+H]^+$ calc. 242.1652; found 242.1645.

1.5. NMR

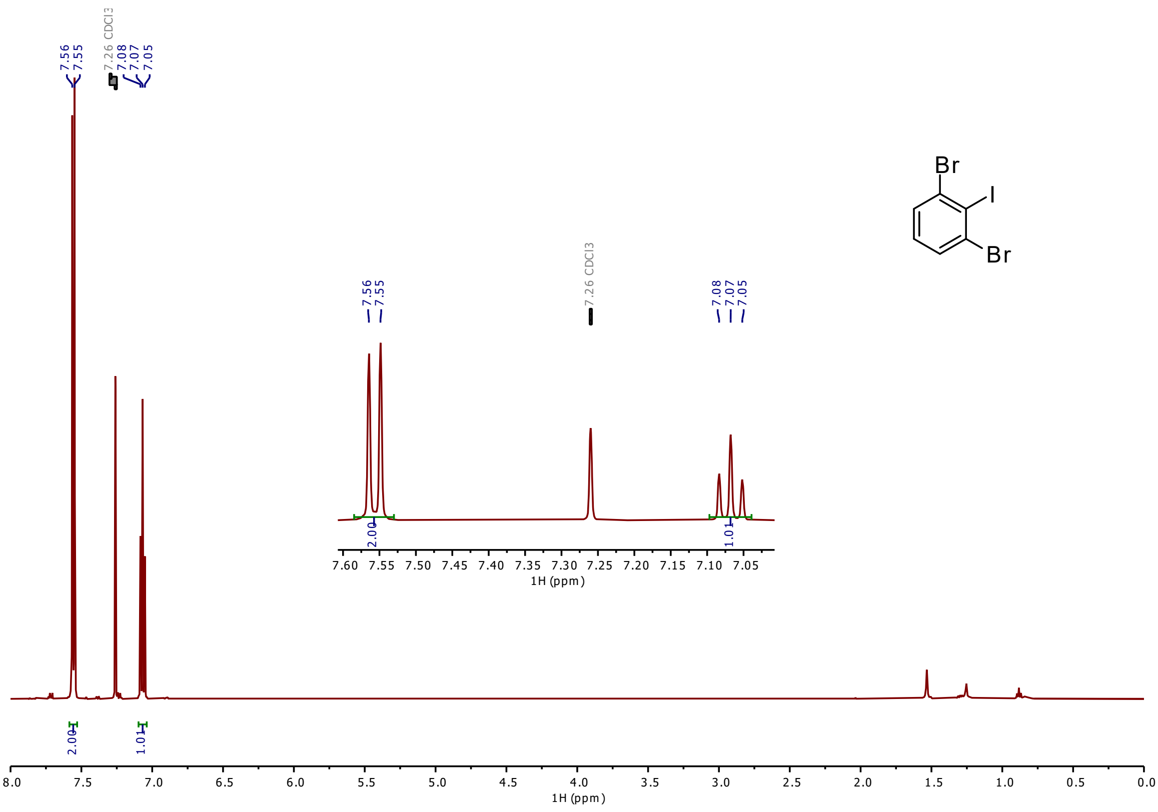

Figure S1.11. ^{1}**H NMR** (501 MHz, $CDCl_3$, 298 K) of **1,3-dibromo-2-iodobenzene**.

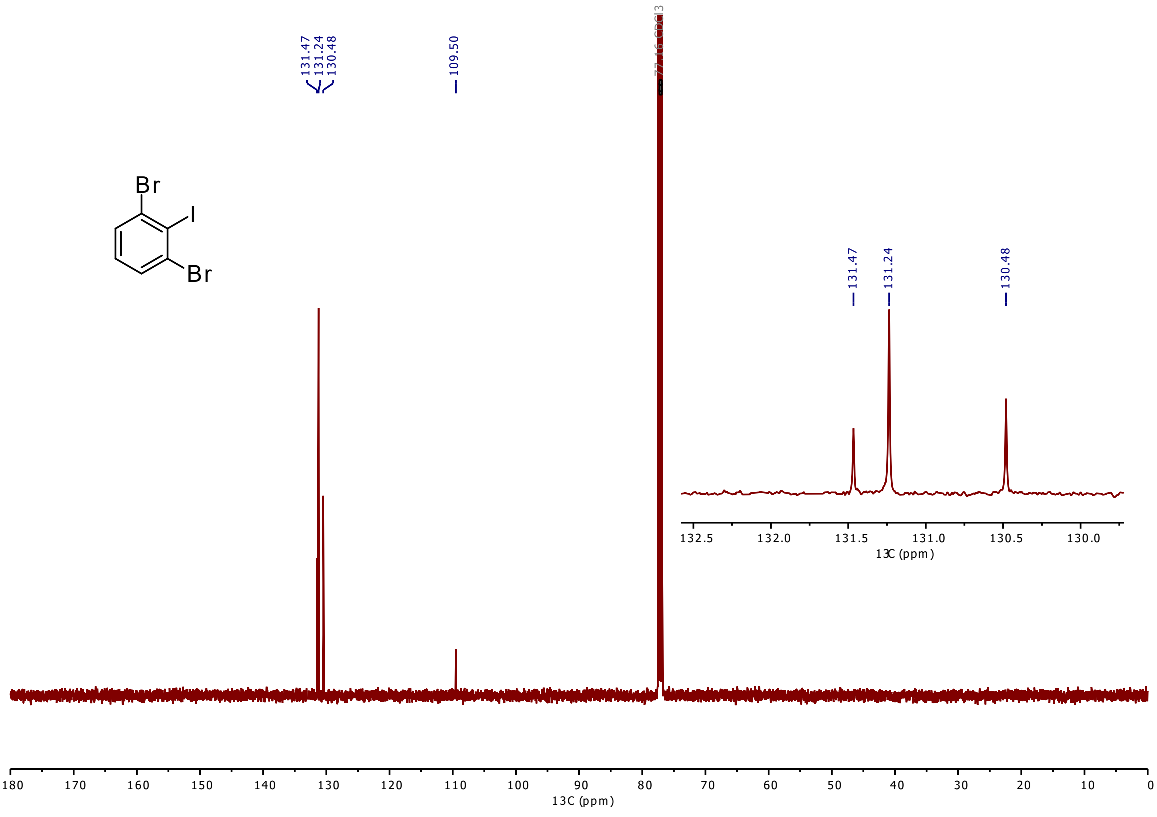

Figure S1.12. ^{13}**C NMR** (126 MHz, $CDCl_3$, 298 K) of **1,3-dibromo-2-iodobenzene.**

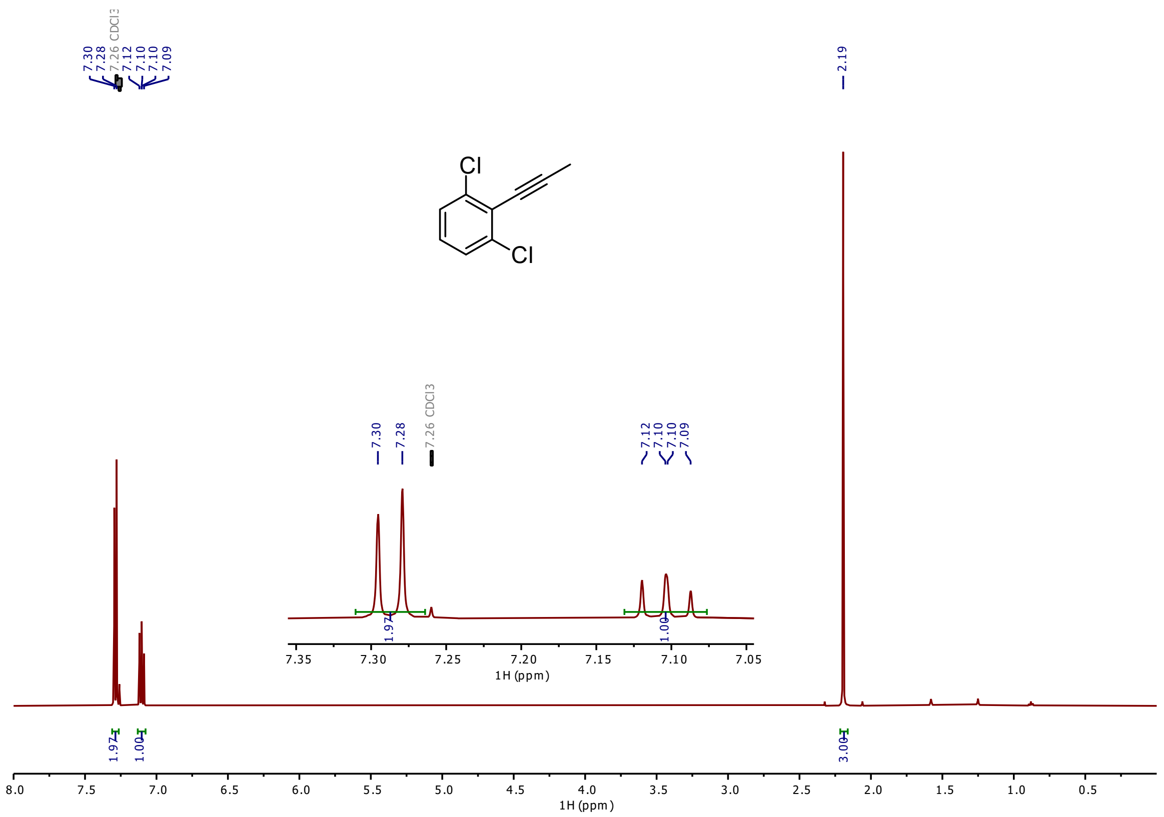

Figure S1.13. ^{1}**H NMR** (501 MHz, $CDCl_3$, 298 K) of **1,3-dichloro-2-propynylbenzene.**

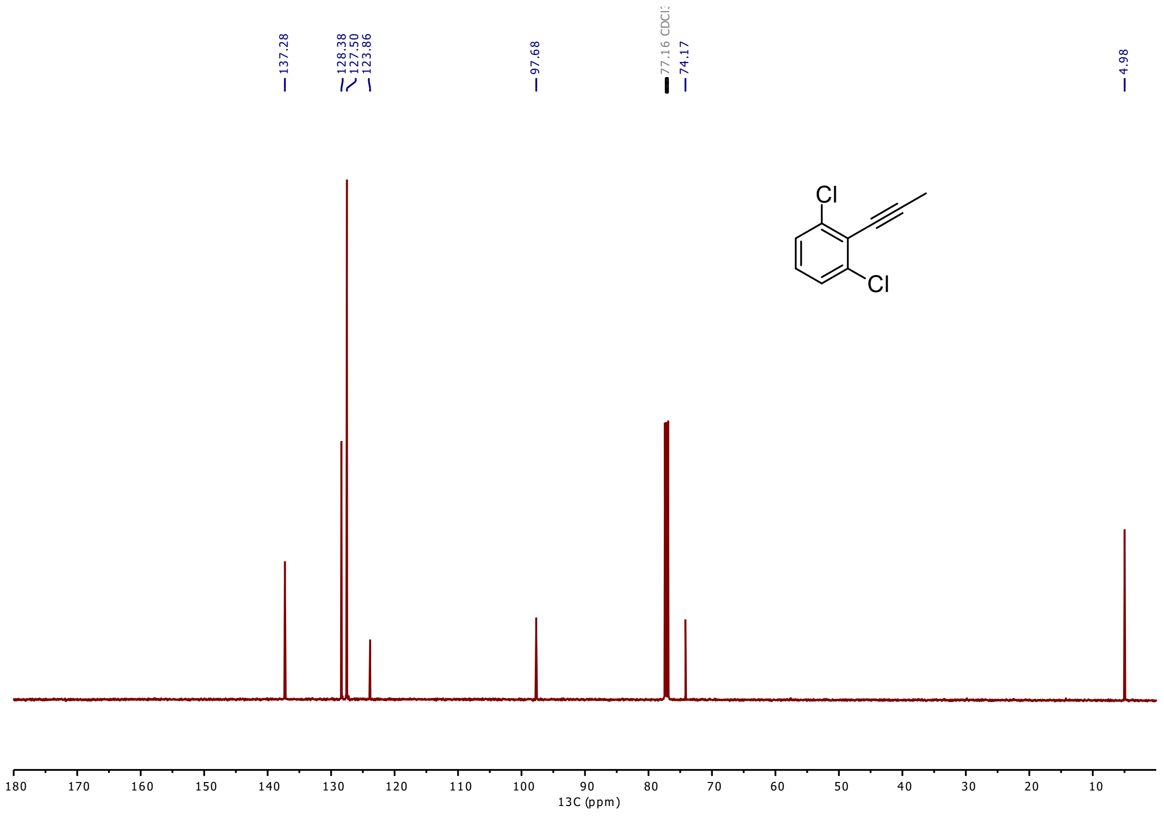

Figure S1.14. ^{13}**C NMR** (126 MHz, $CDCl_3$, 298 K) of **1,3-dichloro-2-propynylbenzene**.

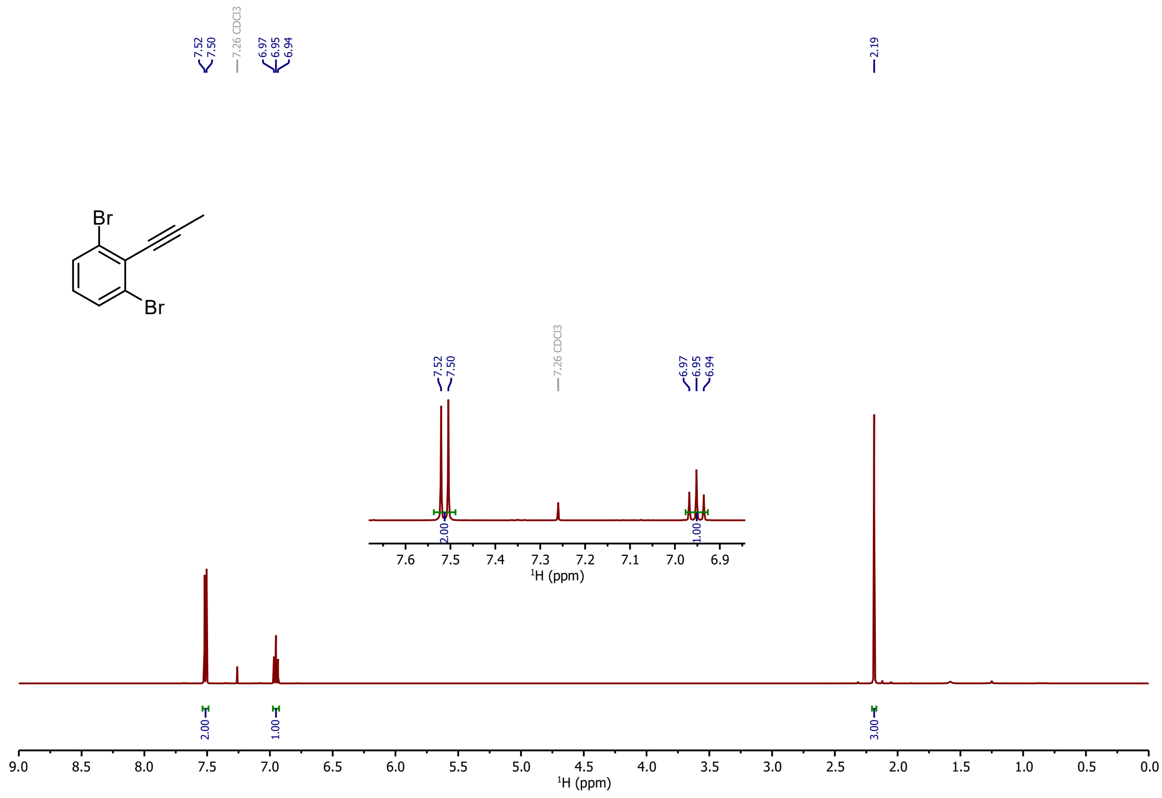

Figure S1.15. **^{1}H NMR** (501 MHz, $CDCl_3$, 298 K) of **1,3-dibromo-2-(prop1-yn1-yl)benzene**.

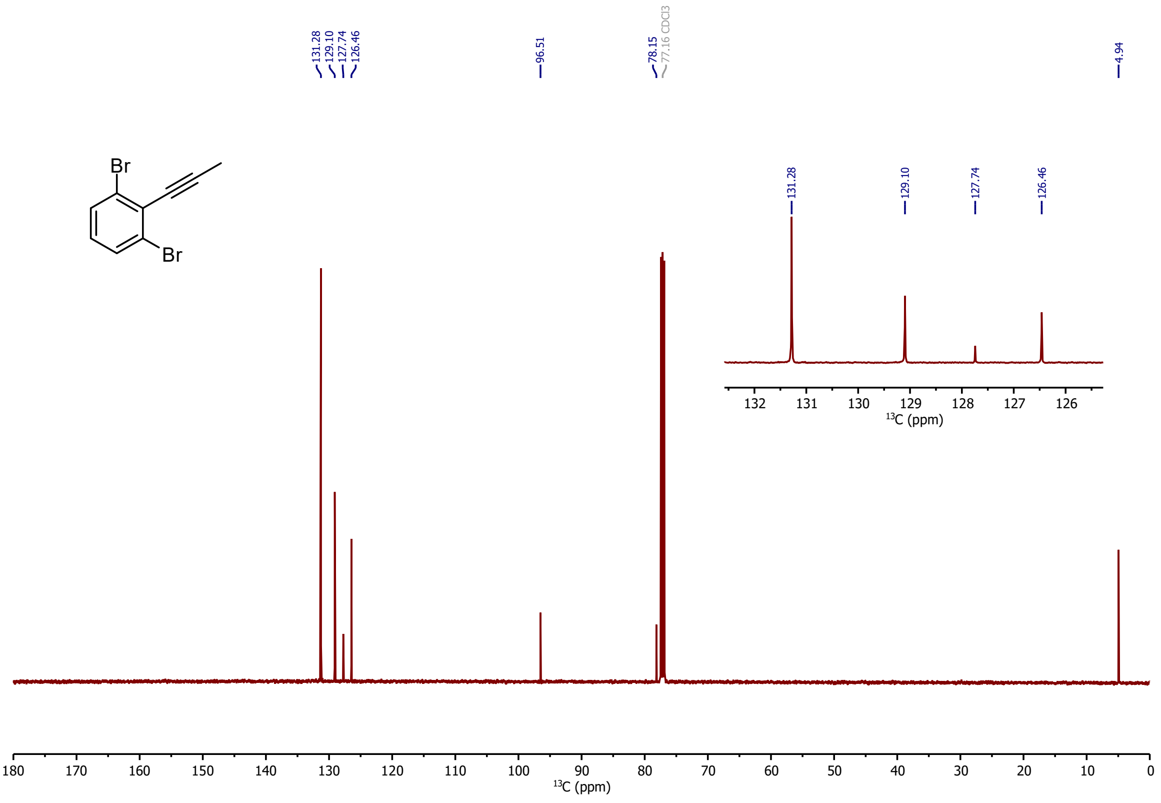

Figure S1.16. **^{13}C NMR** (126 MHz, $CDCl_3$, 298 K) of **1,3-dibromo-2-(prop1-yn1-yl)benzene**.

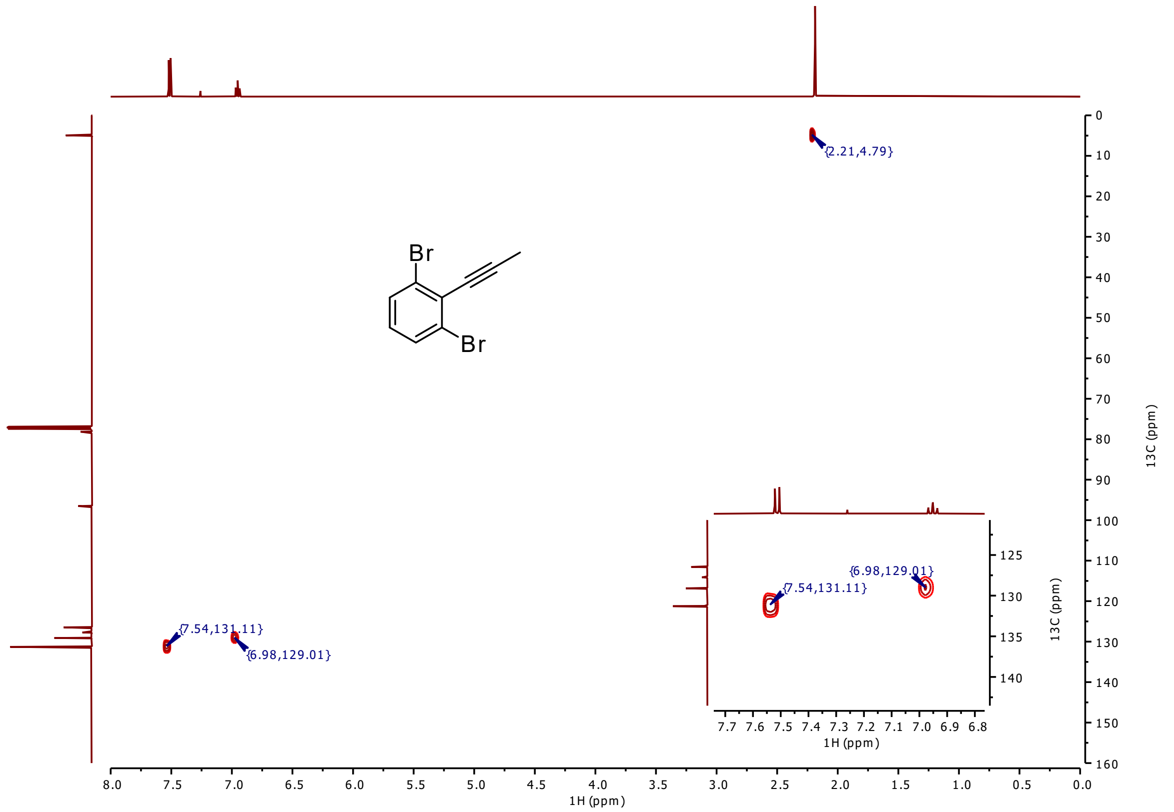

Figure S1.17. $^{1}H/^{13}C$ **HSQC** (500/126 MHz, $CDCl_3$, 298 K) of **1,3-dibromo-2-(prop1-yn1-yl)benzene**.

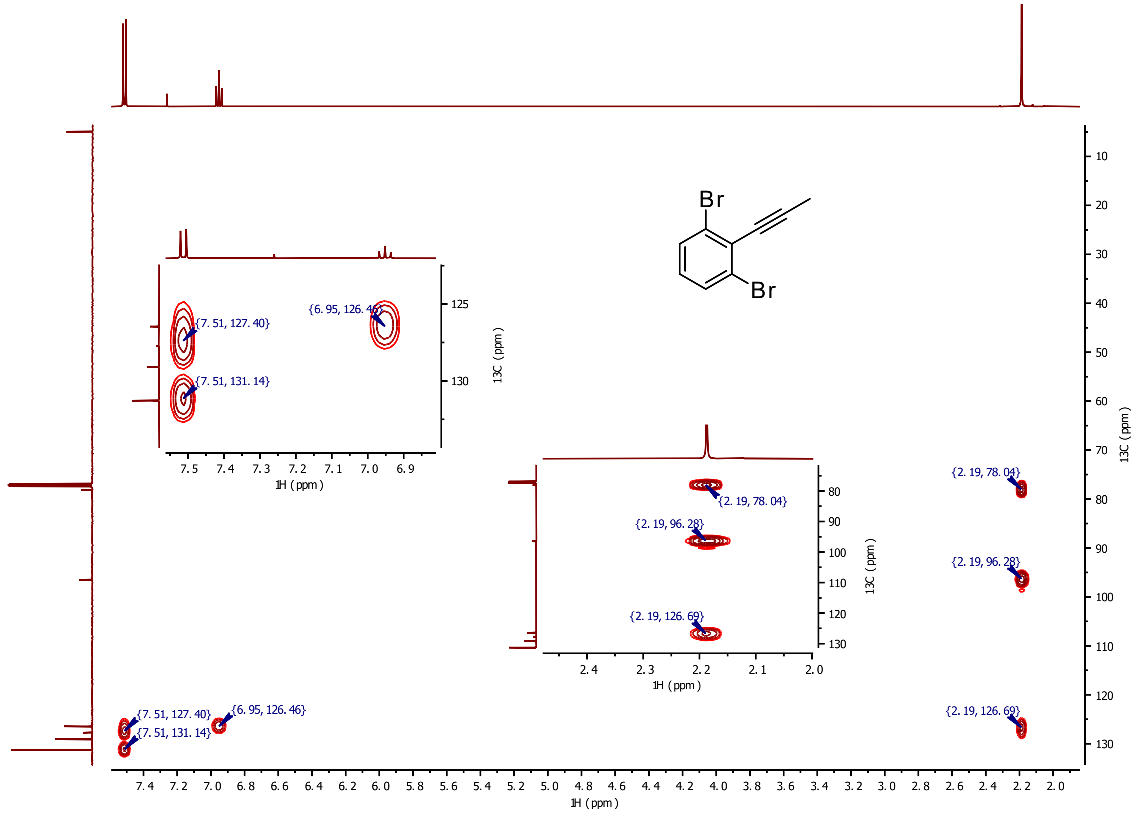

Figure S1.18. $^{1}H/^{13}C$ **HMBC** (500/126 MHz, $CDCl_3$, 298 K) of **1,3-dibromo-2-(prop1-yn1-yl)benzene**.

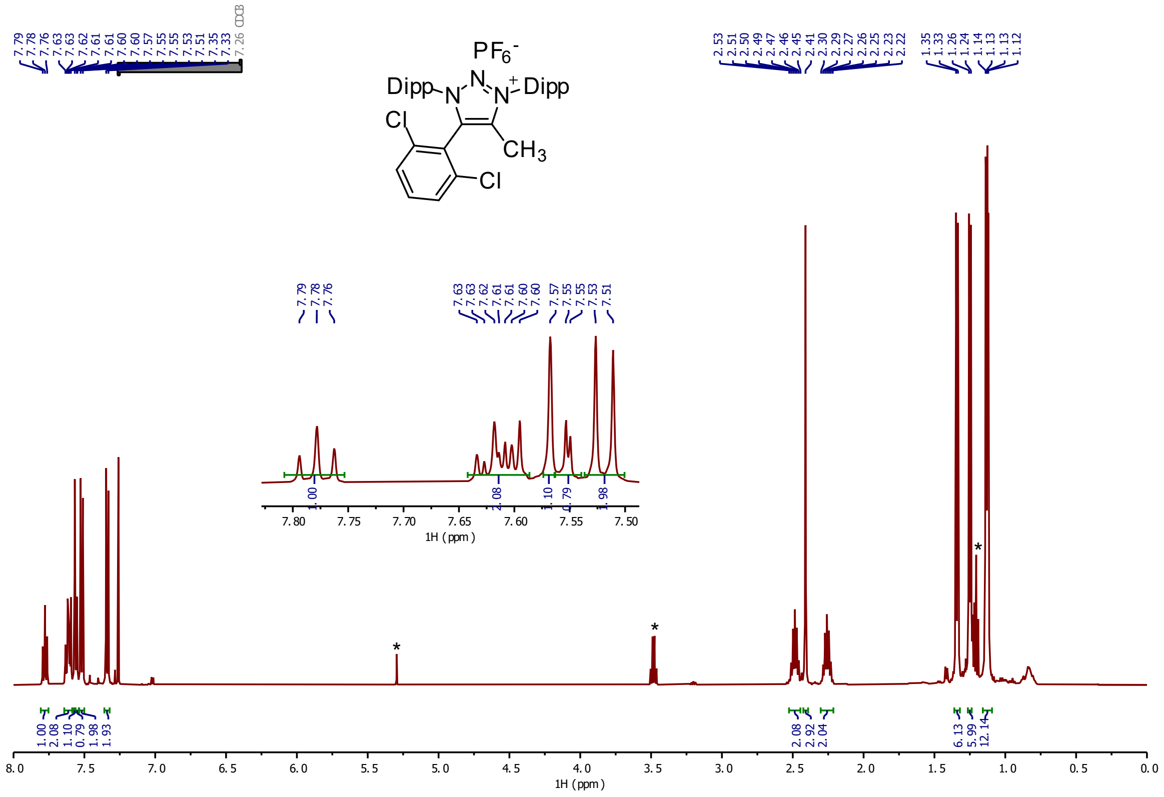

Figure S1.19. ^{1}**H NMR** (501 MHz, $CDCl_3$, 298 K) of **dichloro-triazolium salt** (asterisks mark CH_2Cl_2 and Et_2O).

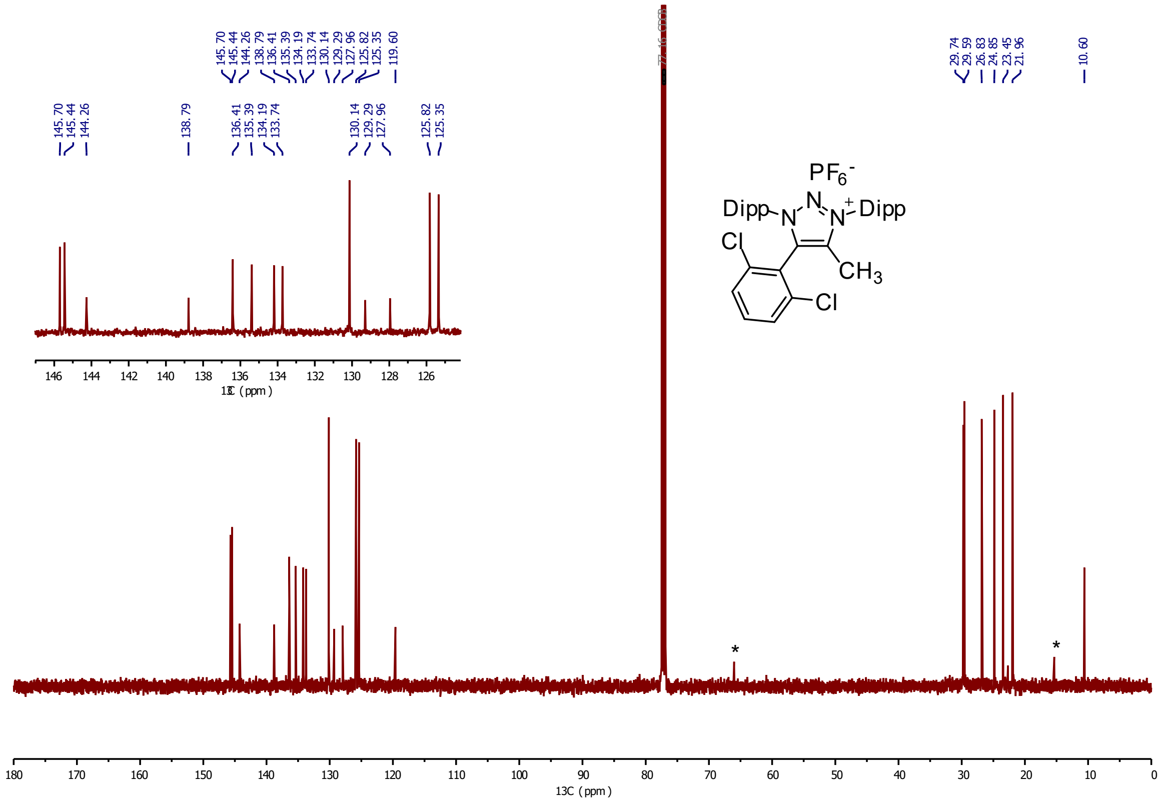

Figure S1.20. ^{13}**C NMR** (126 MHz, $CDCl_3$, 298 K) of **dichloro-triazolium salt** (asterisks mark Et_2O).

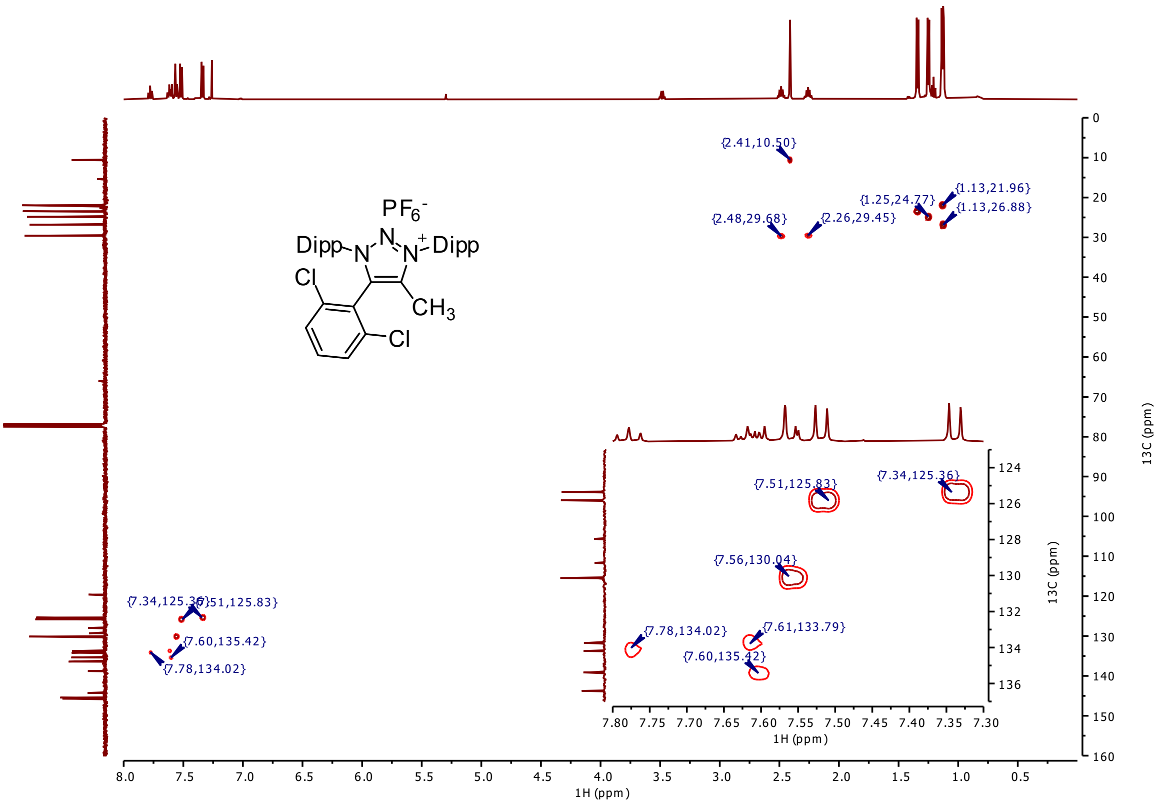

Figure S1.21. ^{1}H/^{13}C **HSQC** (500/126 MHz, $CDCl_3$, 298 K) of **dichloro-triazolium salt**.

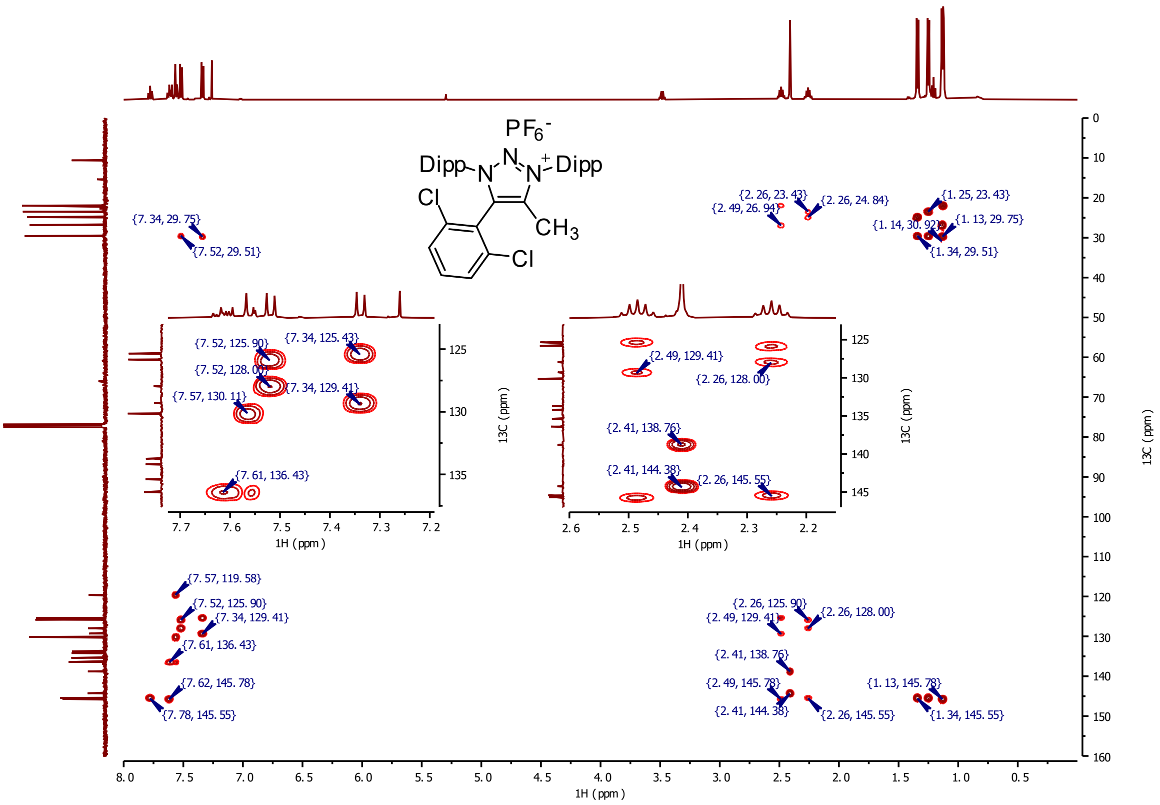

Figure S1.22. ^{1}H/^{13}C **HMBC** (500/126 MHz, $CDCl_3$, 298 K) of **dichloro-triazolium salt**.

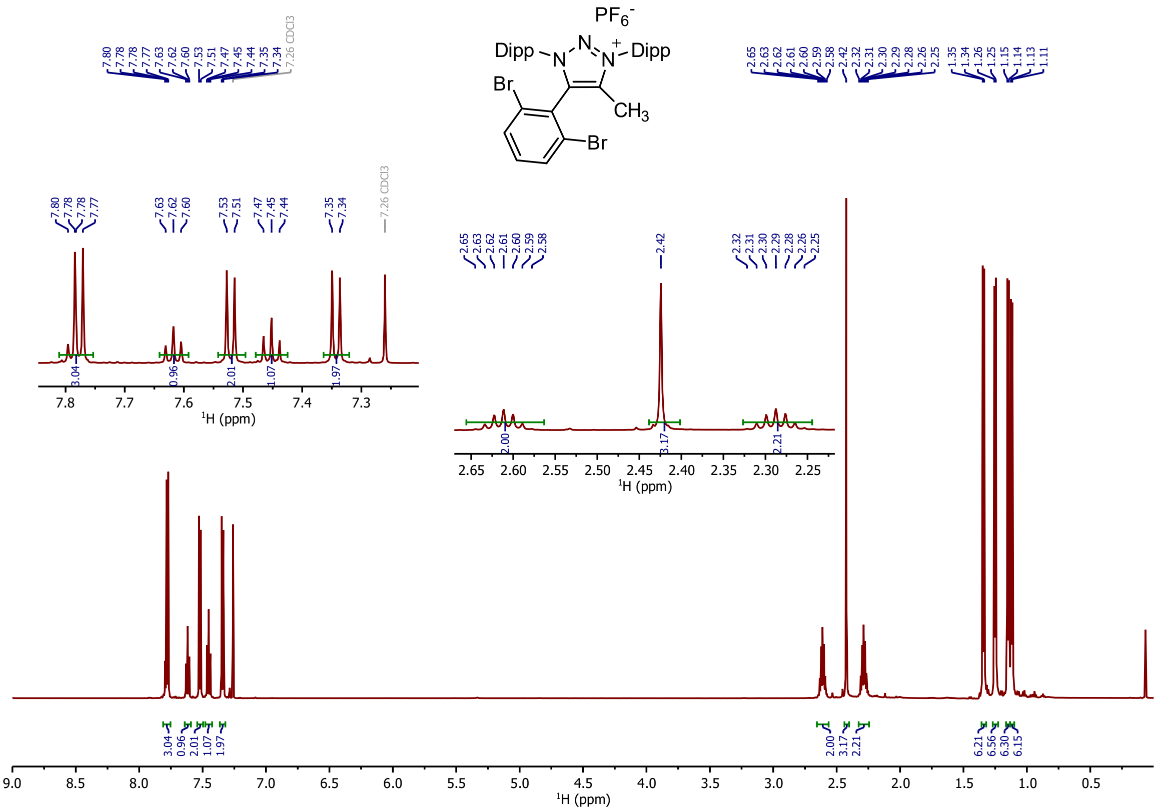

Figure S1.23. ^{1}**H NMR** (600 MHz, $CDCl_3$, 298 K) of **dibromo-triazolium salt**.

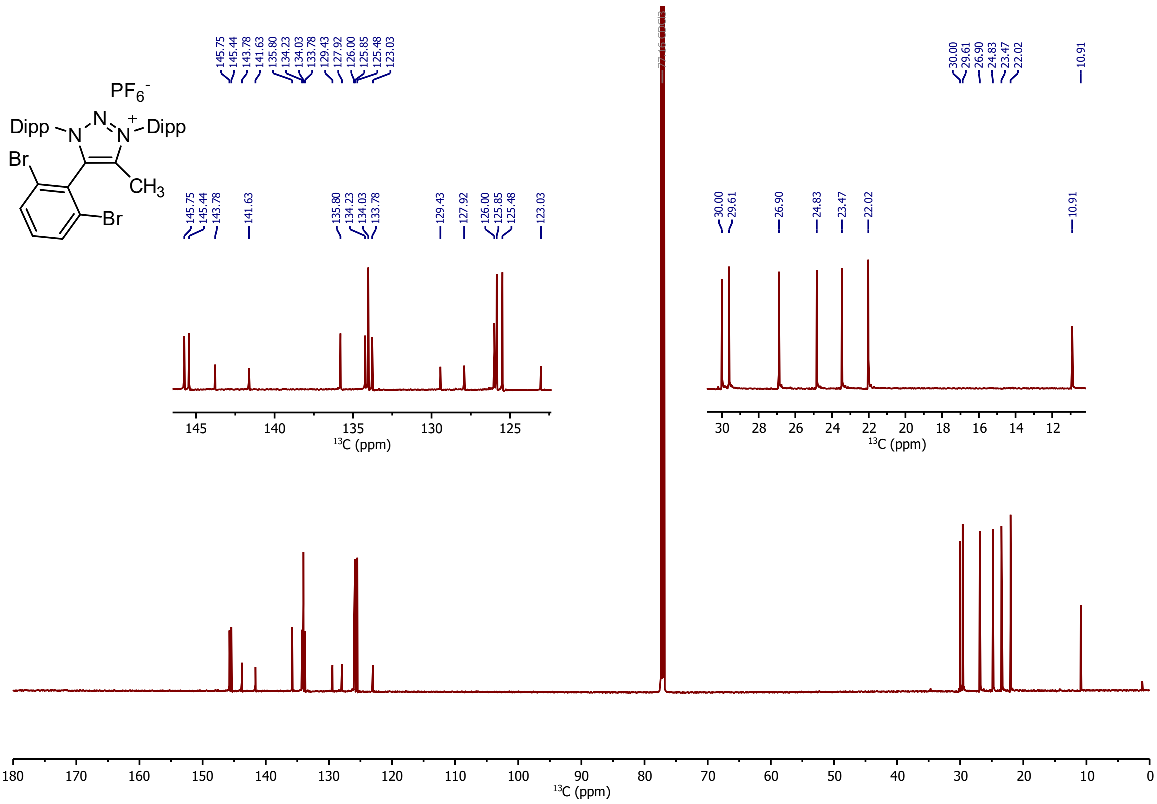

Figure S1.24. ^{13}**C NMR** (151 MHz, $CDCl_3$, 298 K) of **dibromo-triazolium salt**.

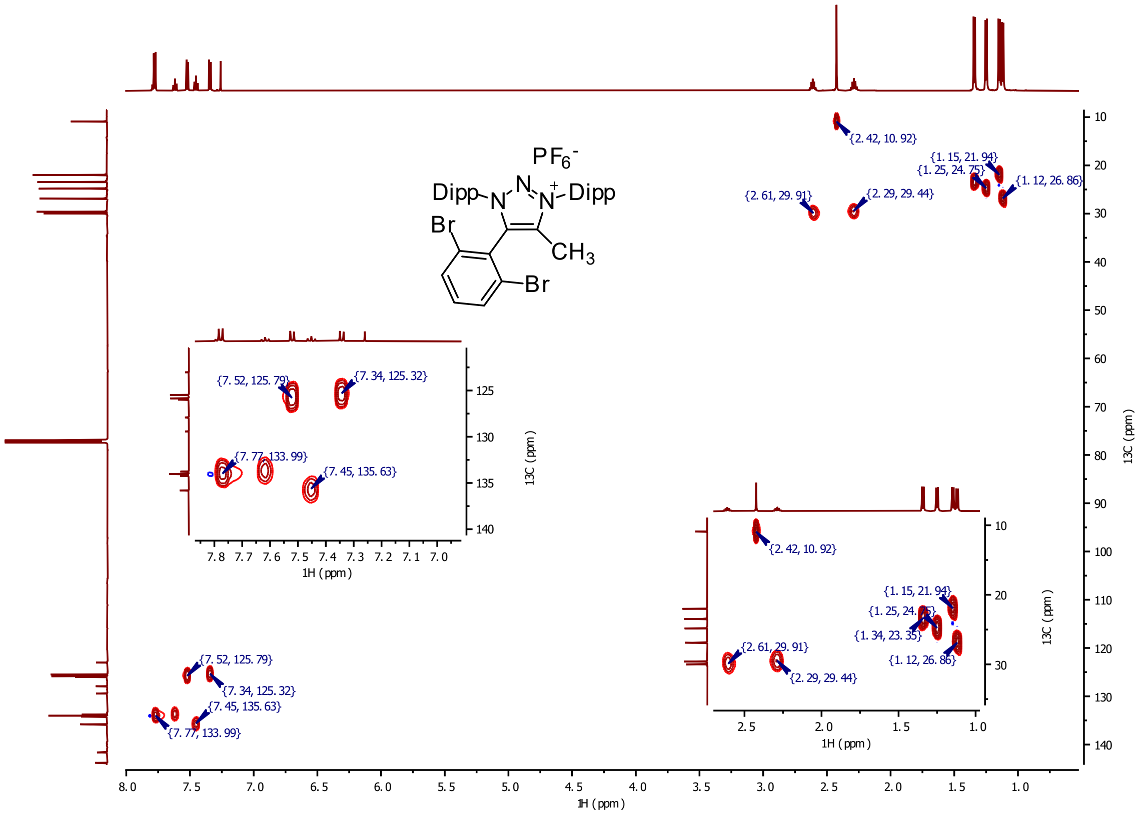

Figure S1.25. ^{1}H/^{13}C HSQC (600/151 MHz, $CDCl_3$, 298 K) of **dibromo-triazolium salt**.

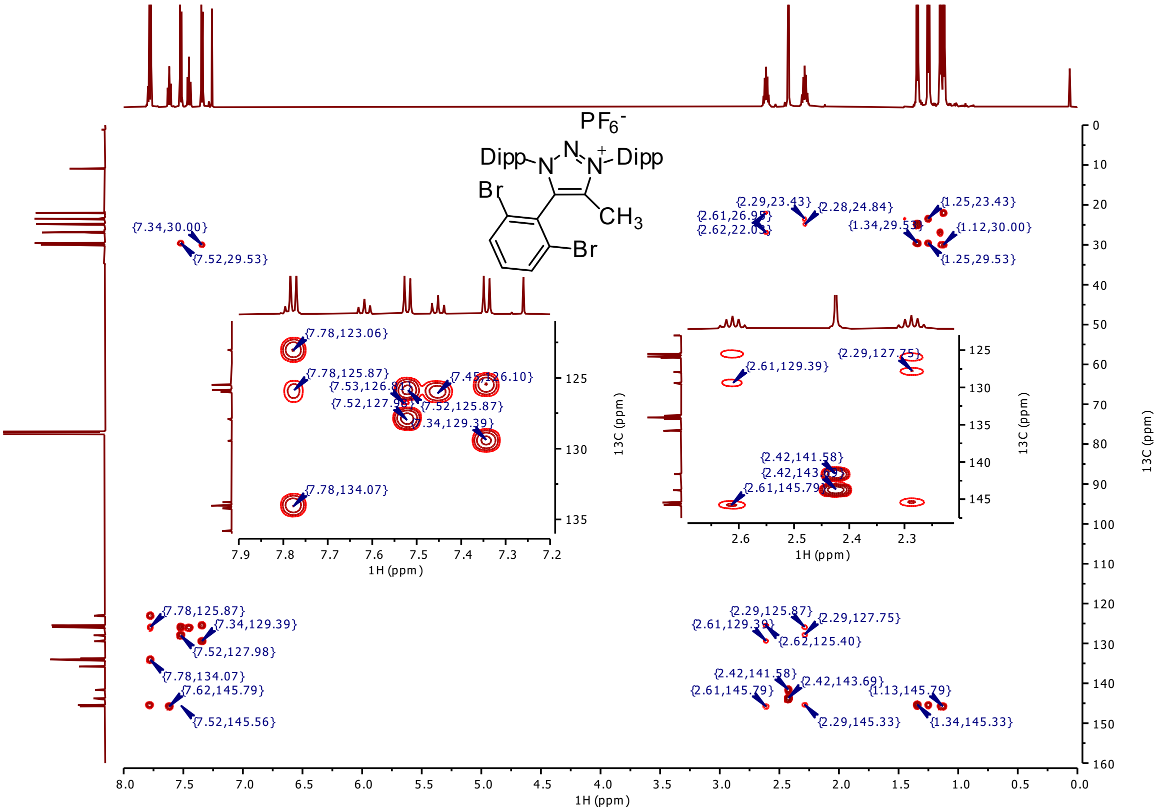

Figure S1.26. ^{1}H/^{13}C HMBC (600/151 MHz, $CDCl_3$, 298 K) of **dibromo-triazolium salt**.

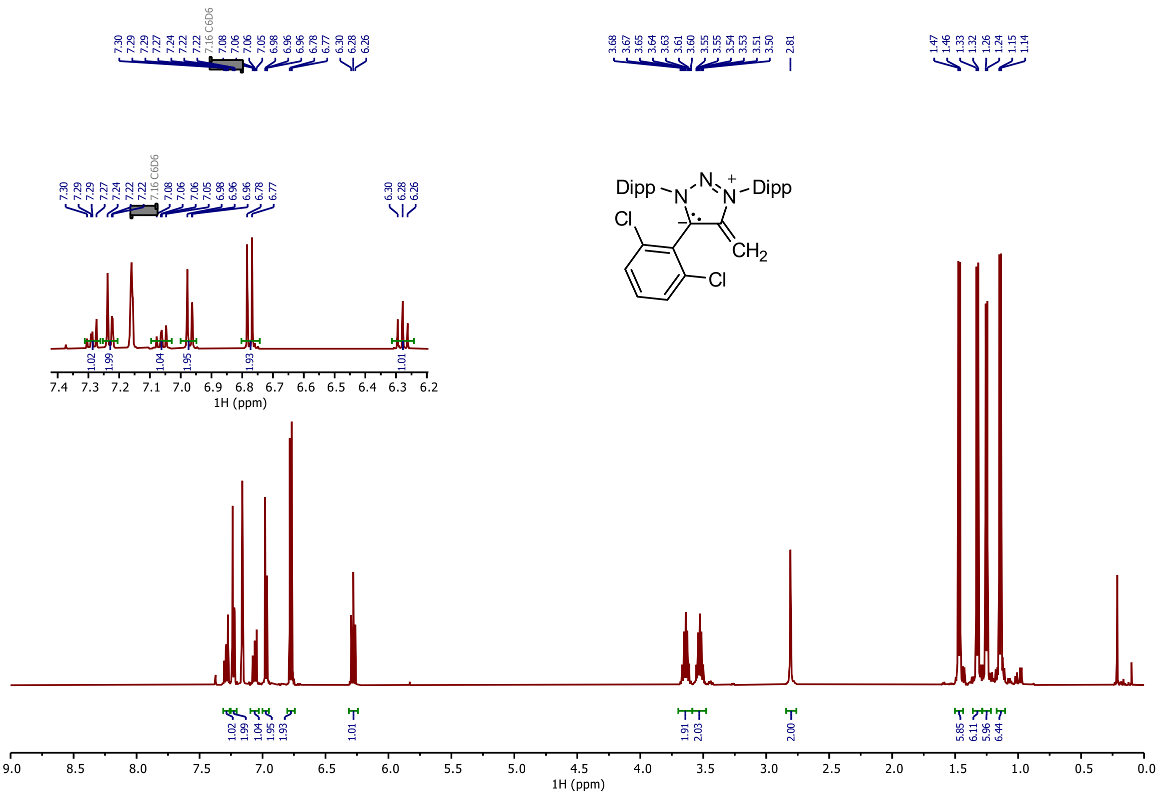

Figure S1.27. **^{1}H NMR** (501 MHz, C_6D_6, 298 K) of **dichloro-mNHO**.

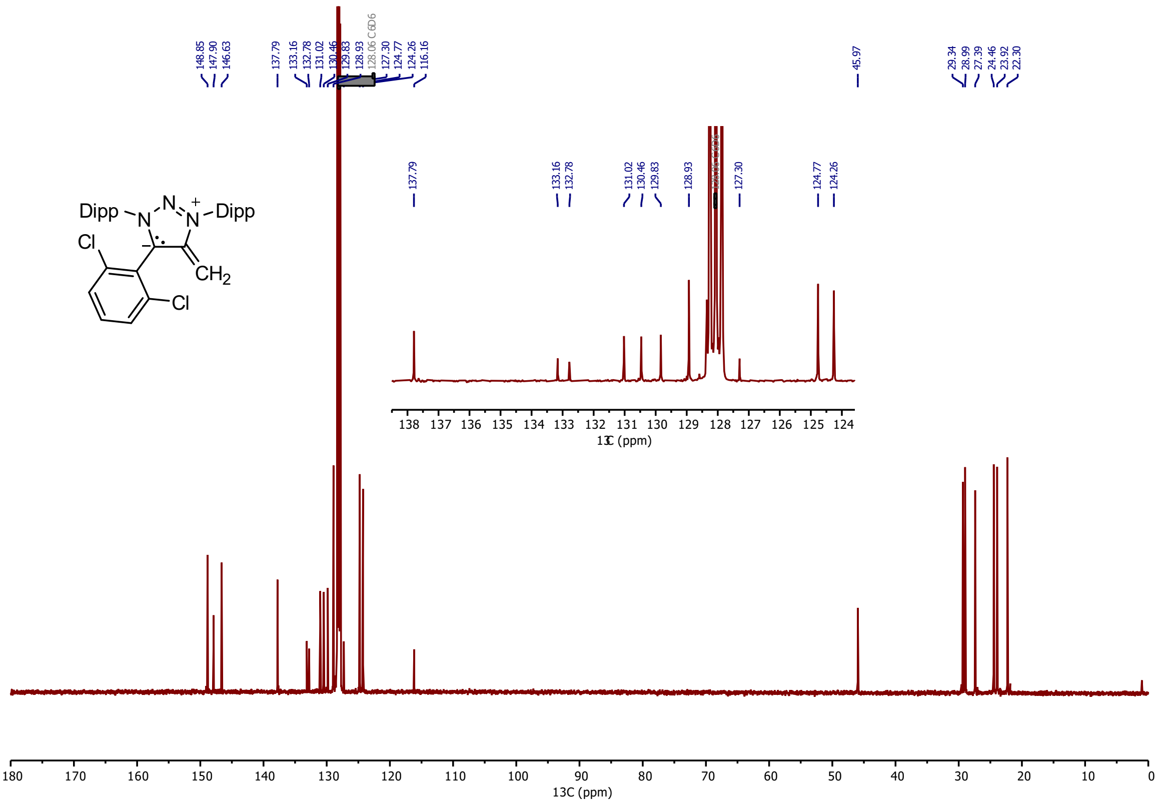

Figure S1.28. **^{13}C NMR** (126 MHz, C_6D_6, 298 K) of **dichloro-mNHO**.

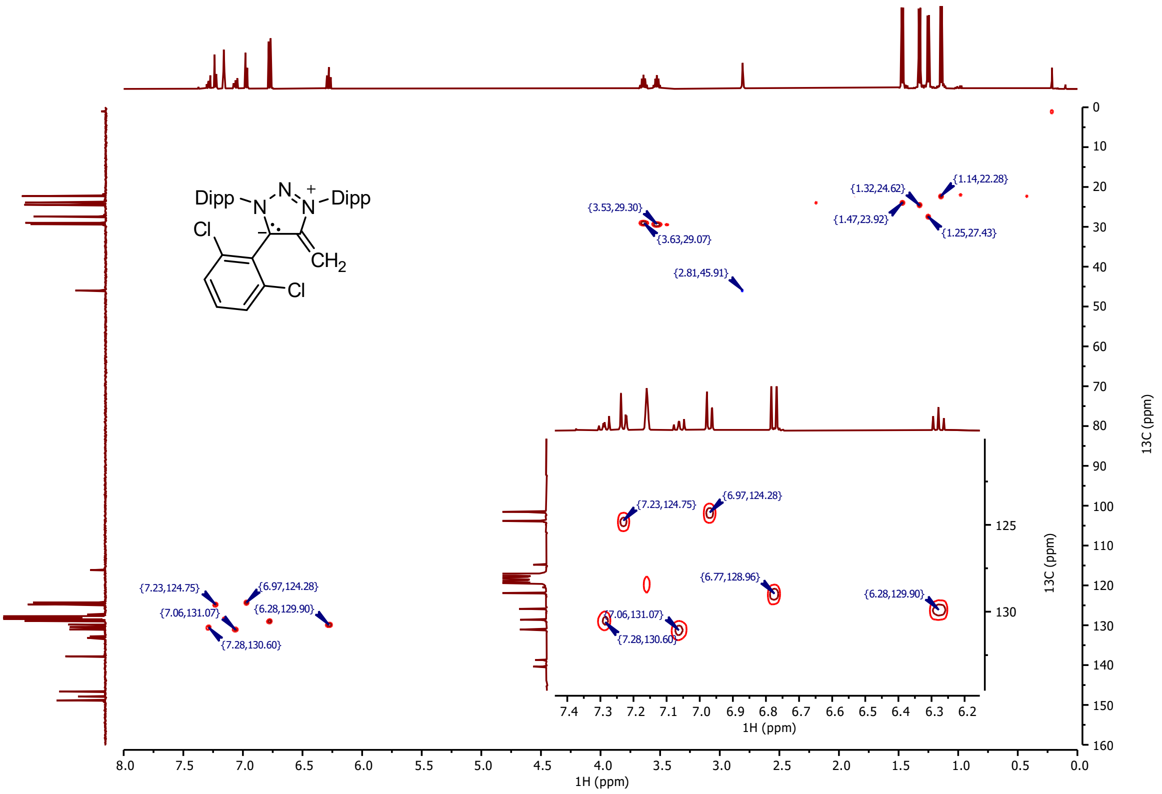

Figure S1.29. **^{1}H/^{13}C HSQC** (500/126 MHz, C_6D_6, 298 K) of **dichloro-mNHO**.

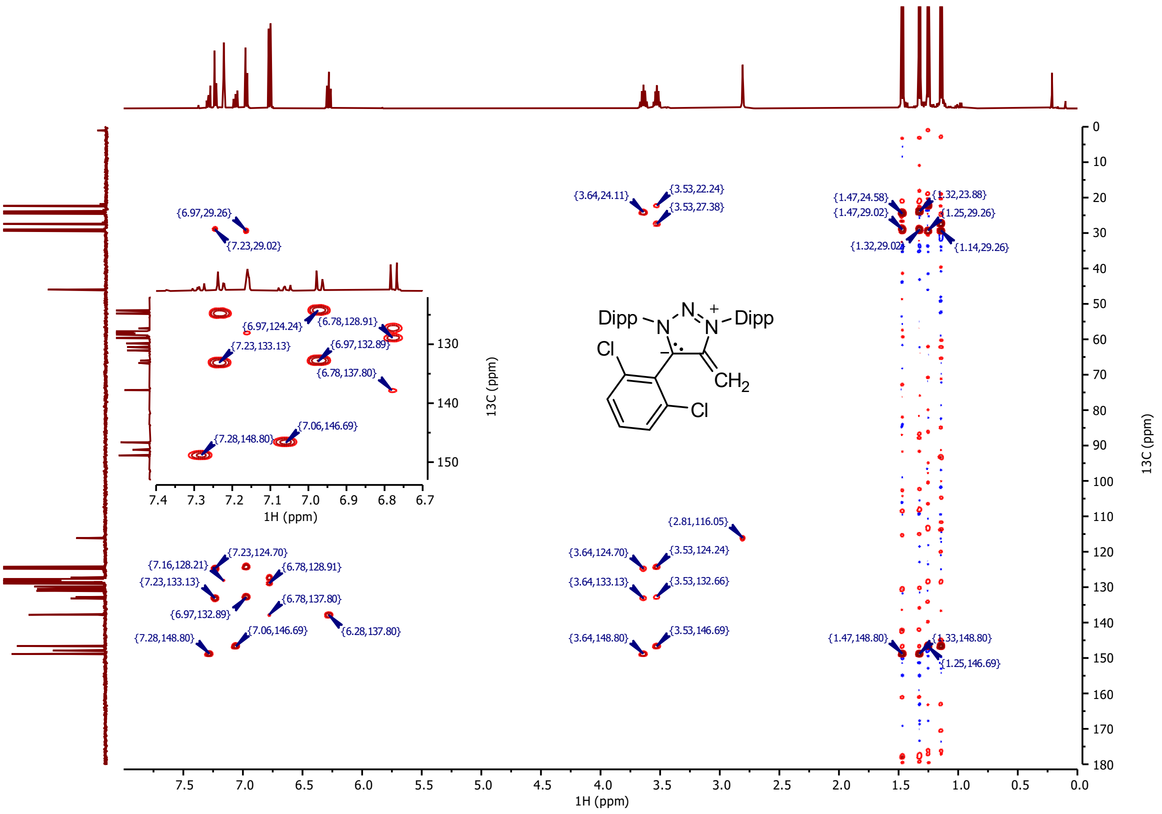

Figure S1.30. **^{1}H/^{13}C HMBC** (500/126 MHz, C_6D_6, 298 K) of **dichloro-mNHO**.

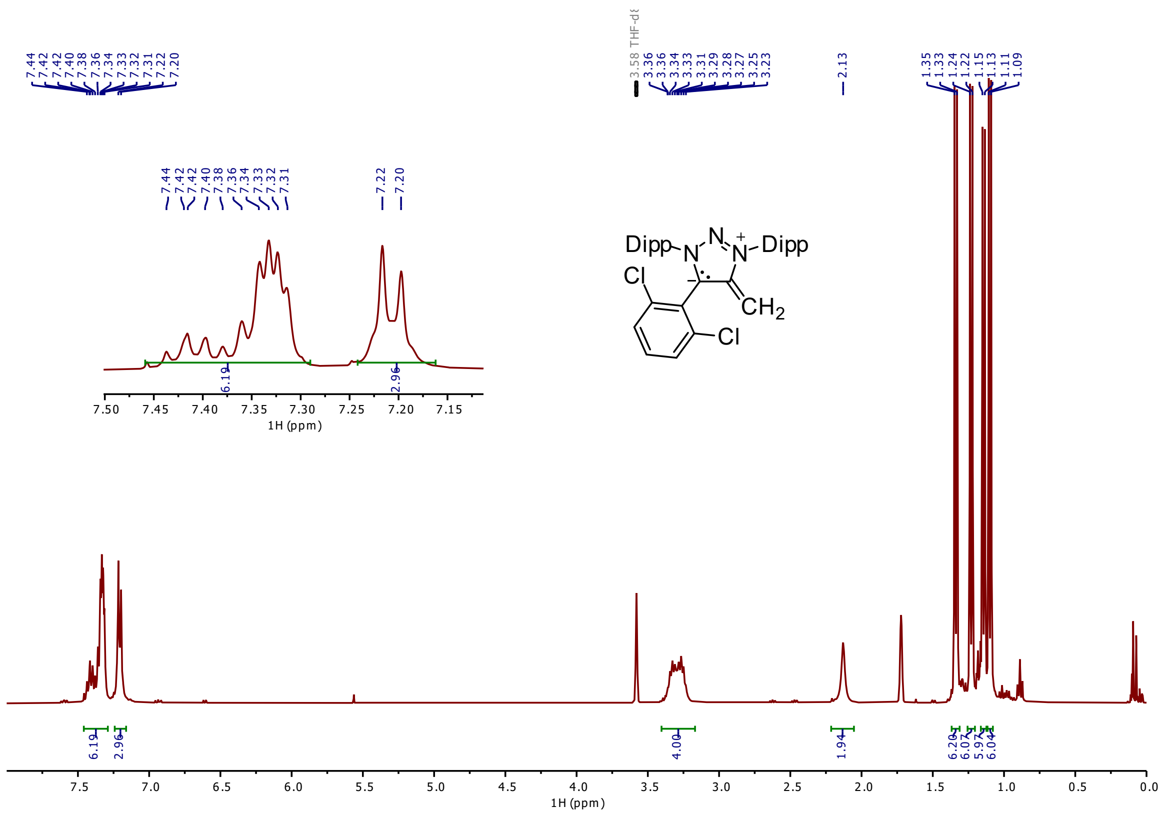

Figure S1.31. **^{1}H NMR** (400 MHz, d_8-THF, 298 K) of **dichloro-mNHO**.

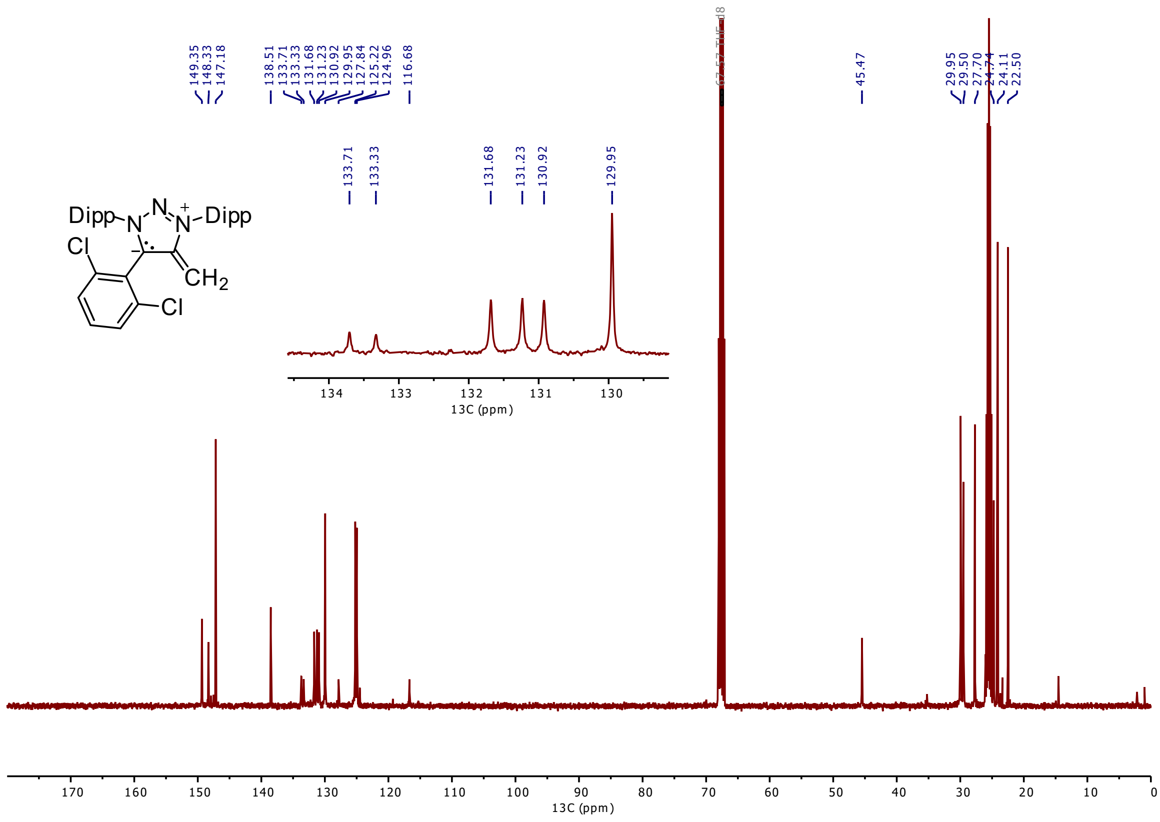

Figure S1.32. **^{13}C NMR** (101 MHz, d_8-THF, 298 K) of **dichloro-mNHO**.

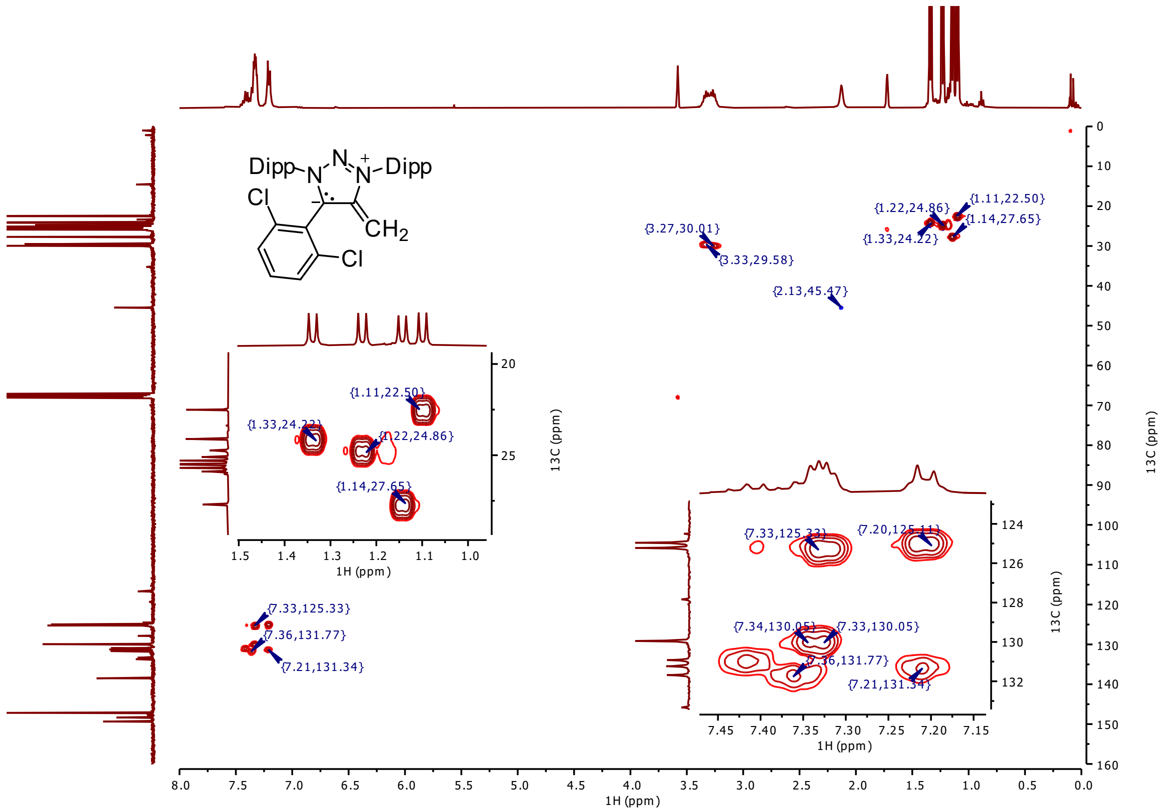

Figure S1.33. **^{1}H/^{13}C HSQC** (400/101 MHz, d_8-THF, 298 K) of **dichloro-mNHO**.

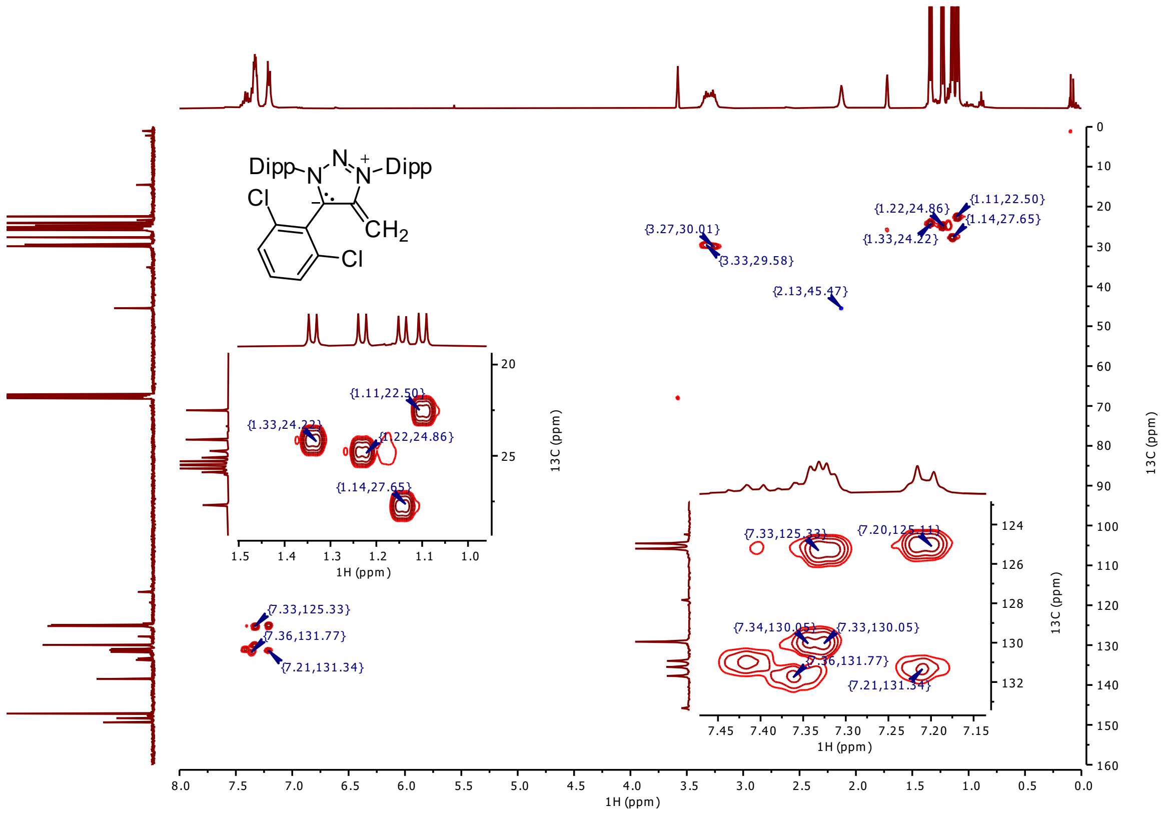

Figure S1.34. **^{1}H/^{13}C HMBC** (400/101 MHz, d_8-THF, 298 K) of **dichloro-mNHO**.

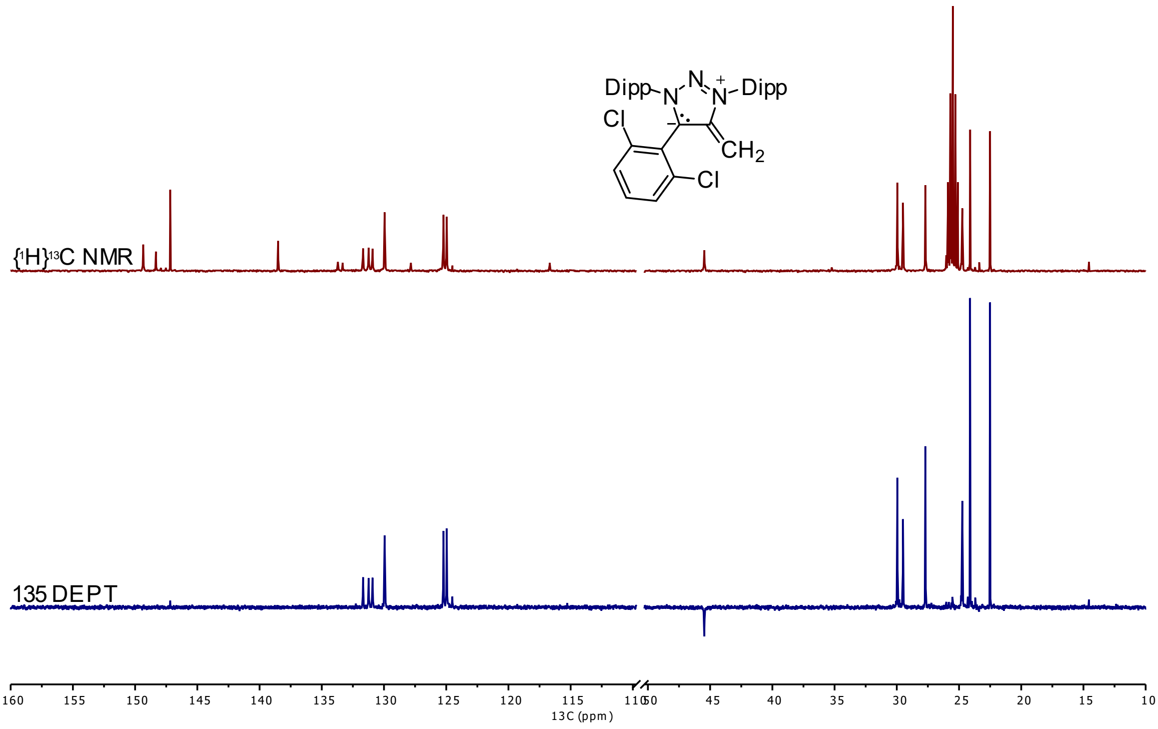

Figure S1.35. Stacked ^{13}**C NMR** (101 MHz, d_8-THF, 298 K, top) and **DEPT** (101 MHz, d_8-THF, 298 K, bottom) of **dichloro-mNHO**.

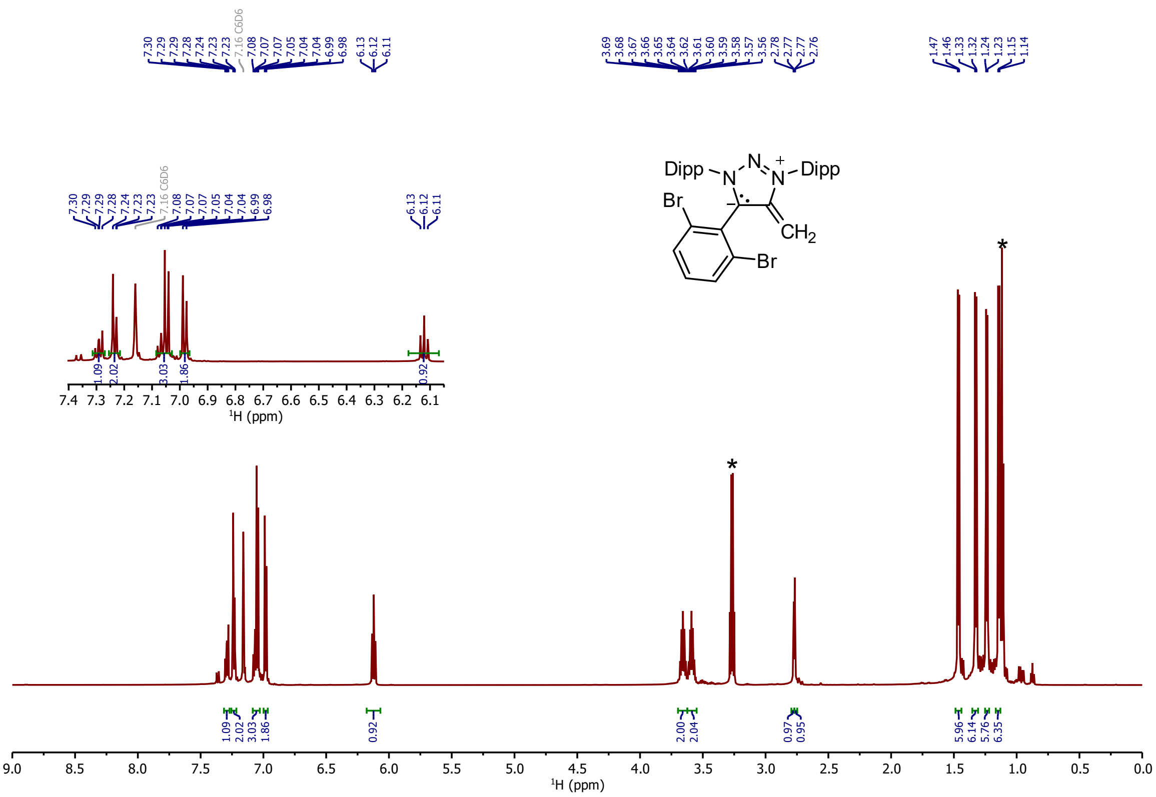

Figure S1.36. ^{1}**H NMR** (600 MHz, C_6D_6, 298 K) of **dibromo-mNHO**.

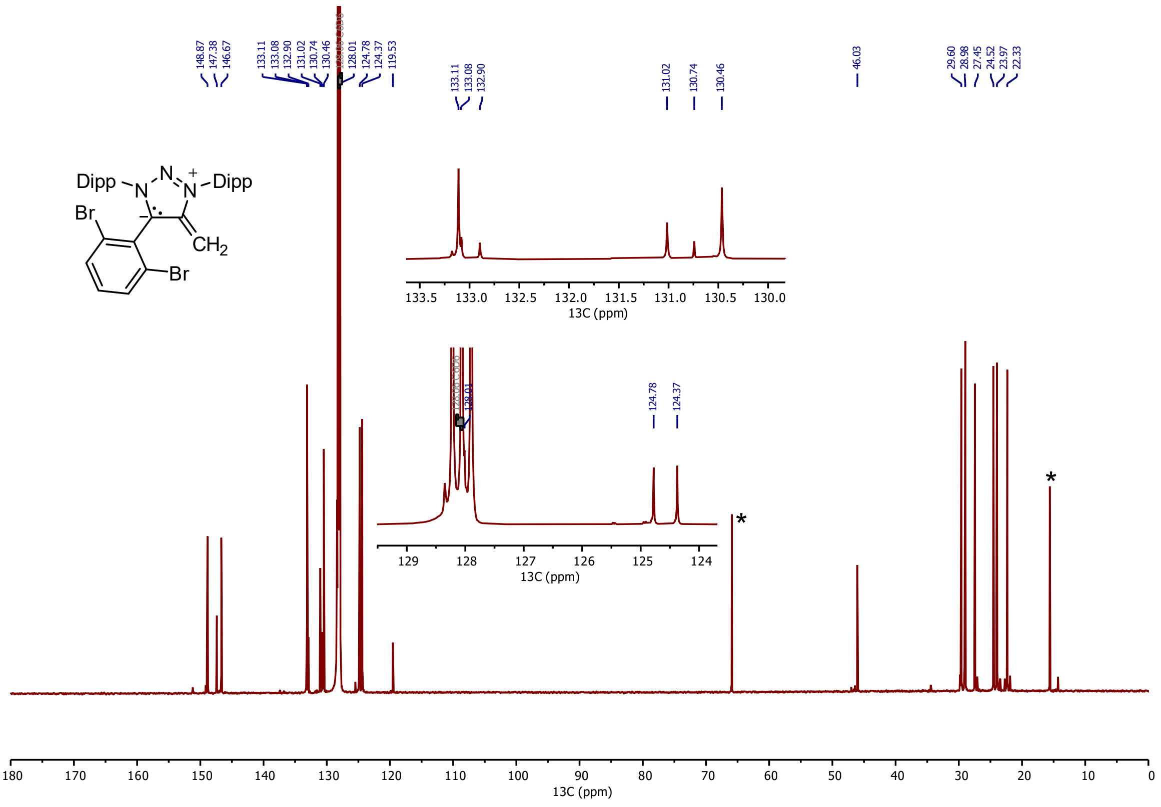

Figure S1.37. **^{13}C NMR** (151 MHz, C_6D_6, 298 K) of **dibromo-mNHO**.

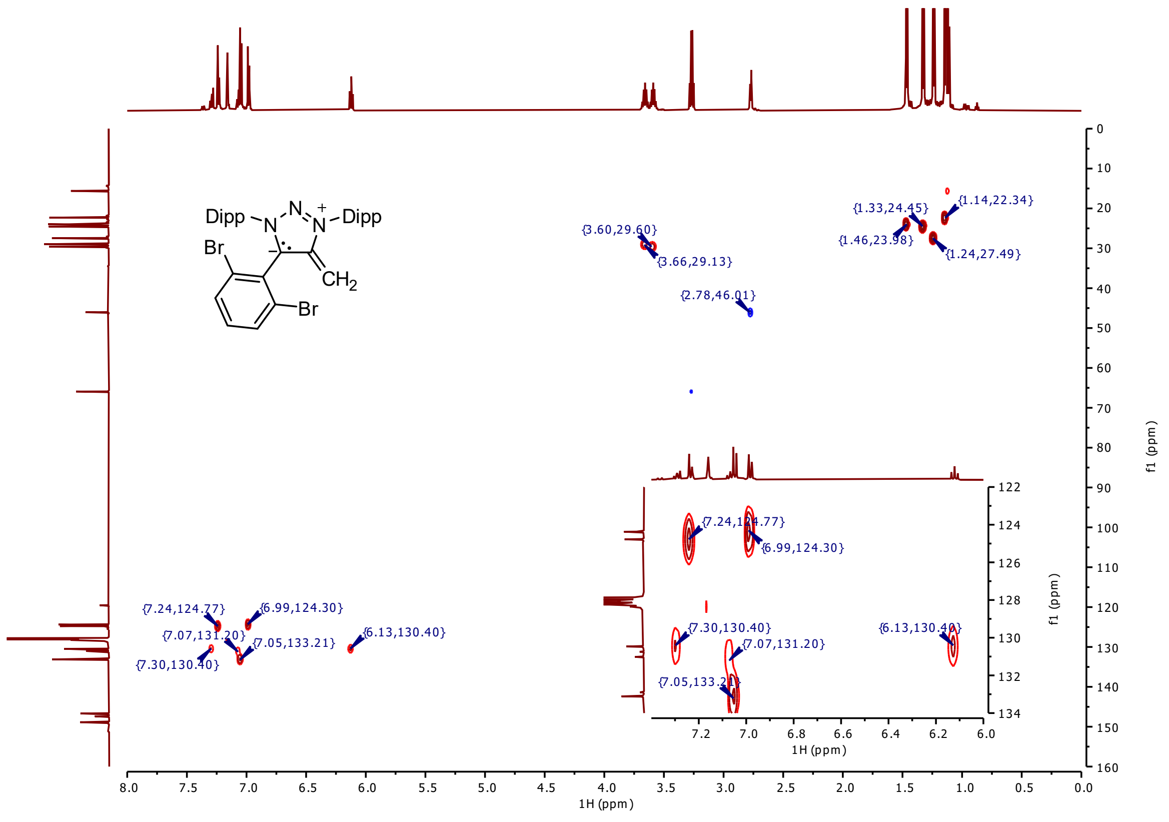

Figure S1.38. **^{1}H/^{13}C HSQC** (600/151 MHz, C_6D_6, 298 K) of **dibromo-mNHO**.

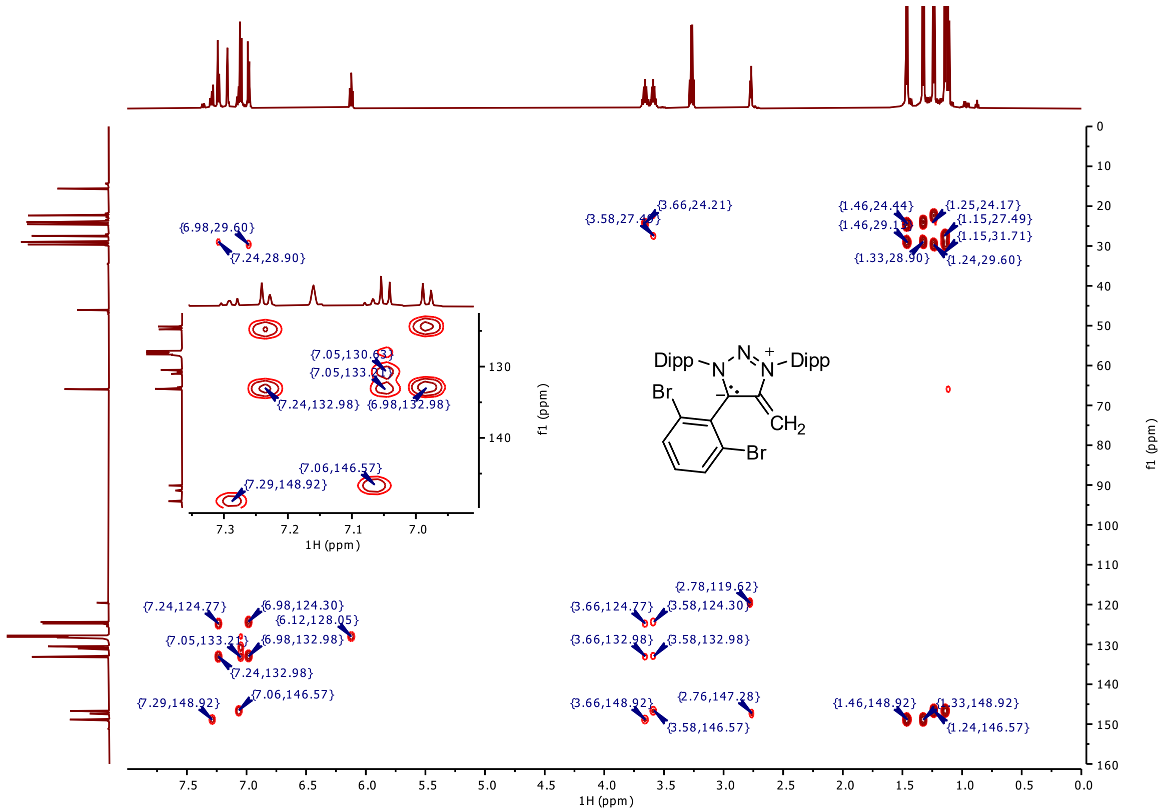

Figure S1.39. **^{1}H/^{13}C HMBC** (600/151 MHz, C_6D_6, 298 K) of **dibromo-mNHO**.

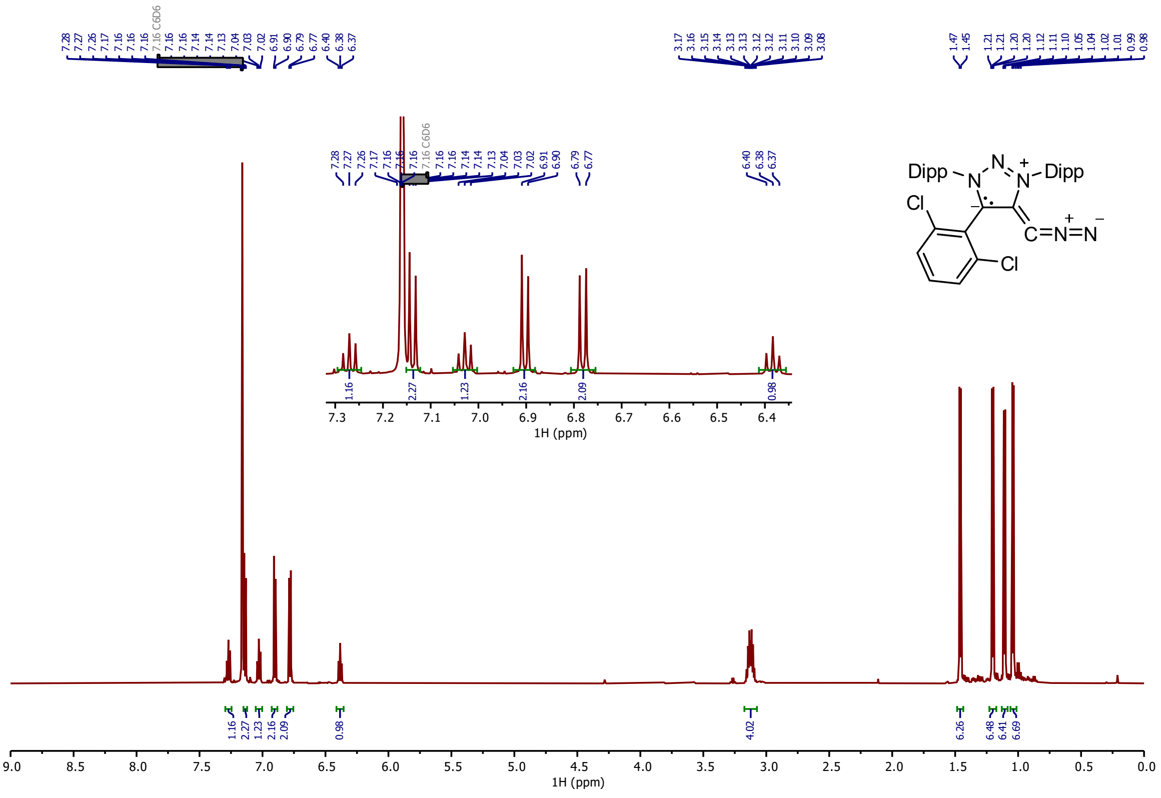

Figure S1.40. **^{1}H NMR** (501 MHz, C_6D_6, 298 K) of **1A^{Cl}**.

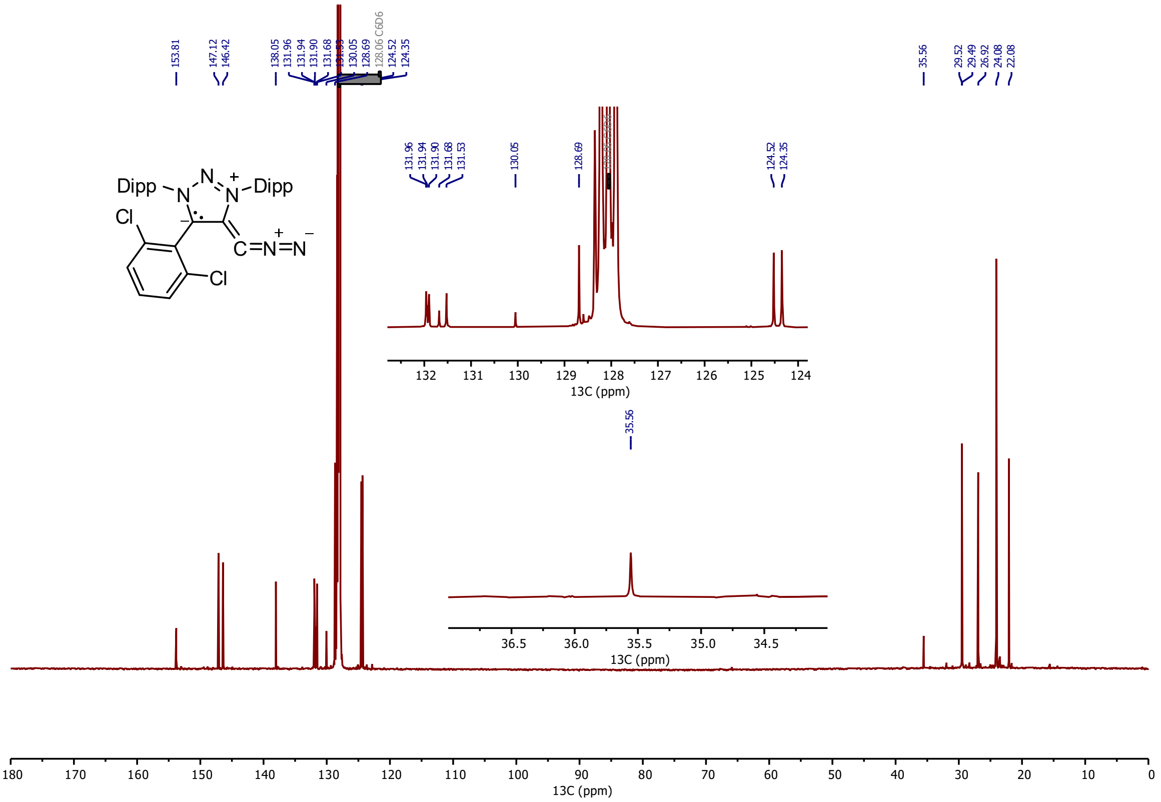

Figure S1.41. **^{13}C NMR** (126 MHz, C_6D_6, 298 K) of **1A^{Cl}**.

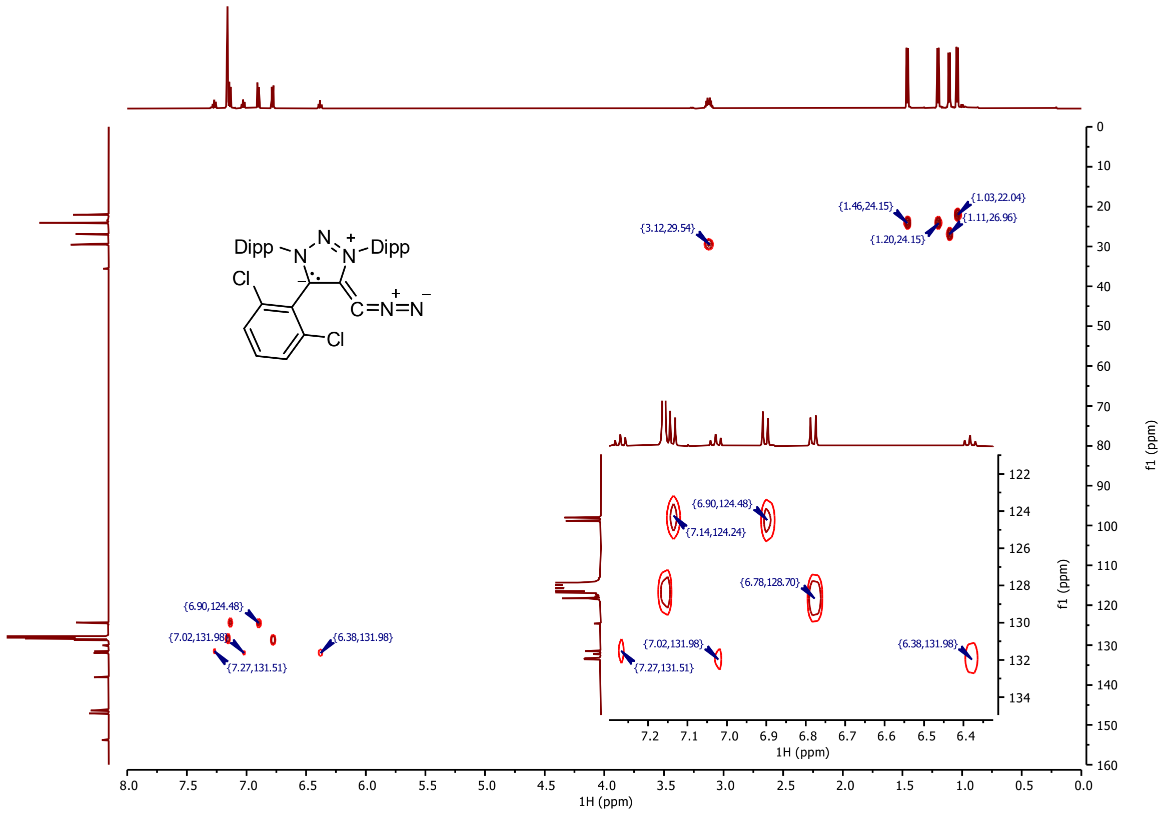

Figure S1.42. **^{1}H/^{13}C HSQC** (500/126 MHz, C_6D_6, 298 K) of **1A^{Cl}**.

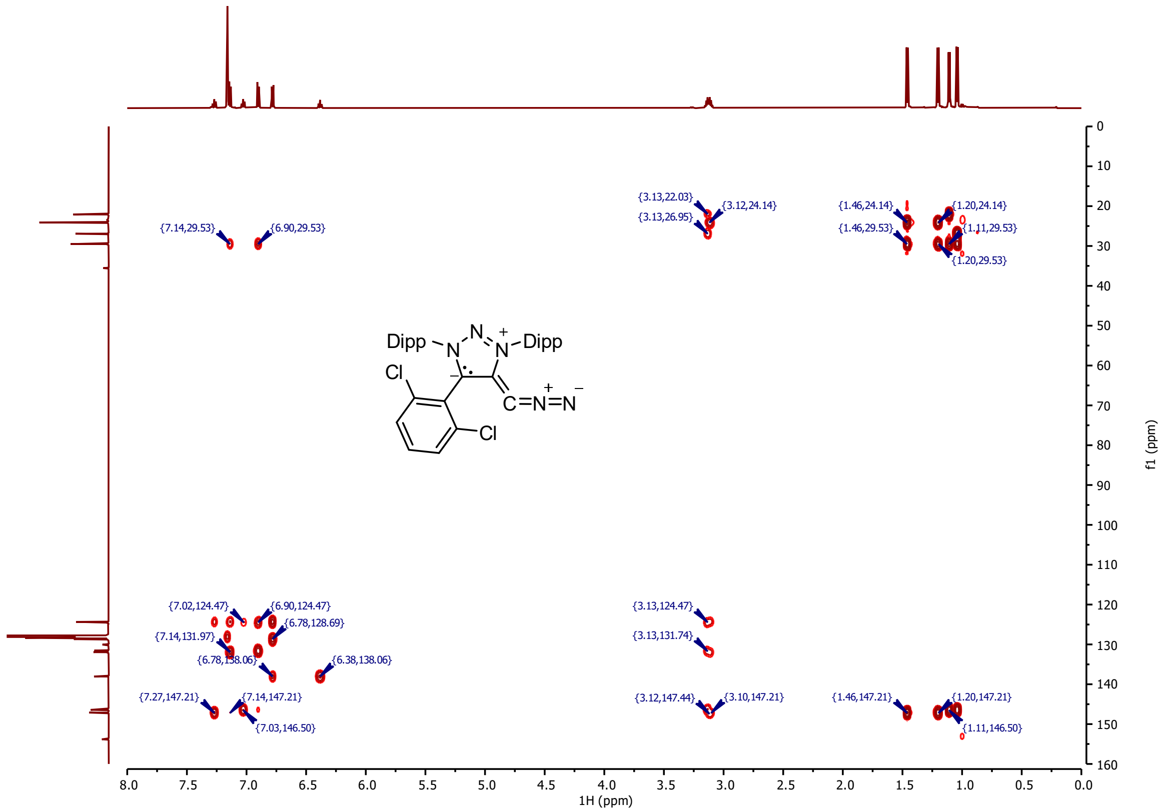

Figure S1.43. **^{1}H/^{13}C HMBC** (500/126 MHz, C_6D_6, 298 K) of **1A^{Cl}**.

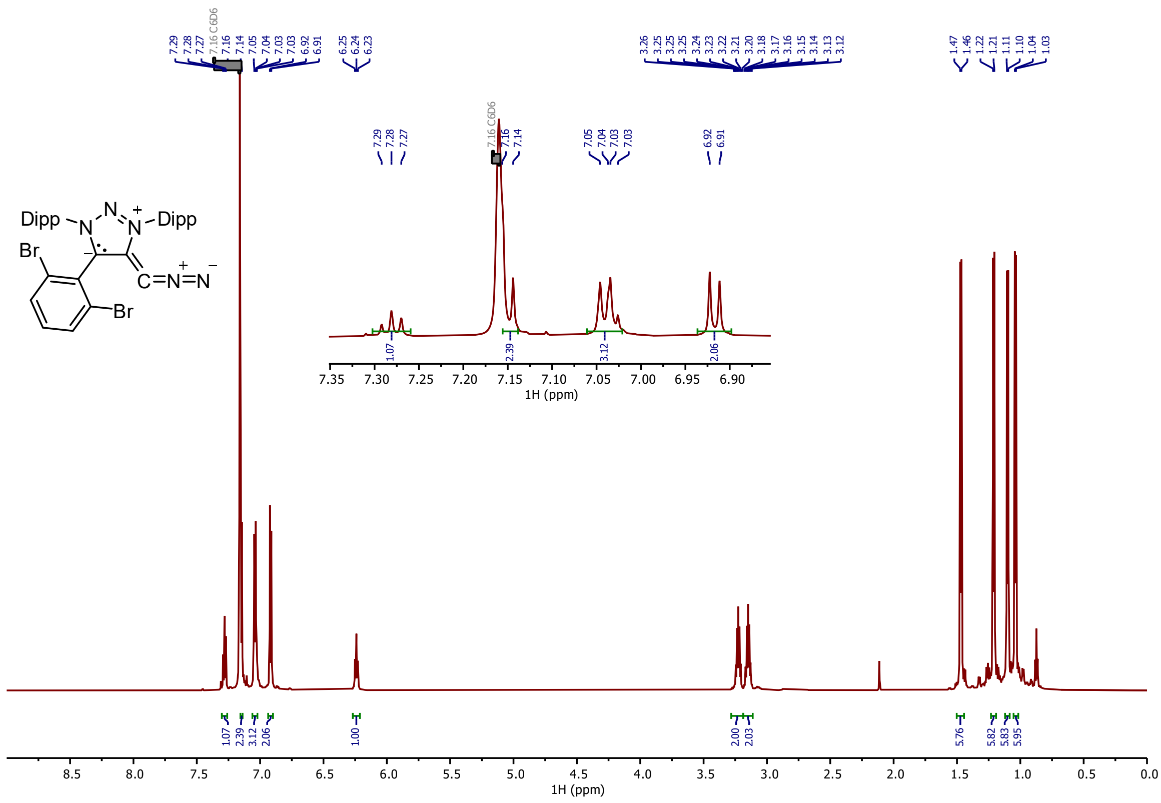

Figure S1.44. **^{1}H NMR** (700 MHz, C_6D_6, 298 K) of **1A^{Br}**.

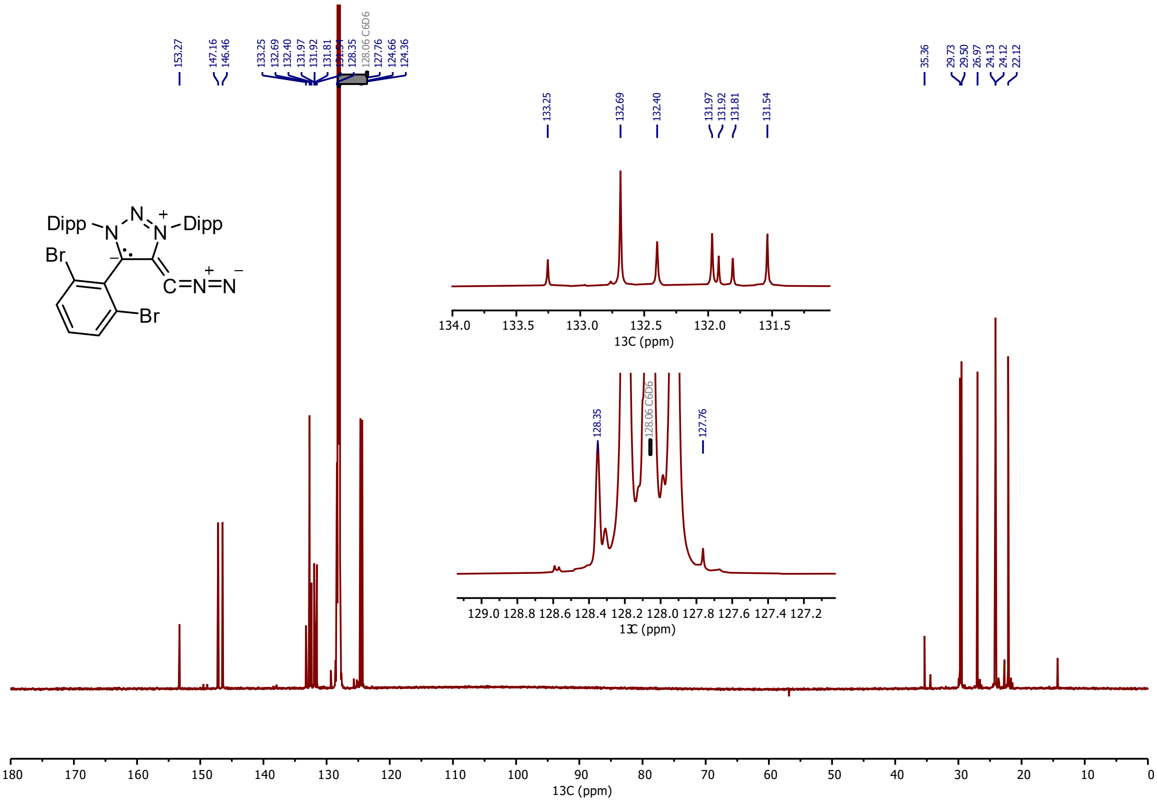

Figure S1.45. ^{13}C NMR (176 MHz, C_6D_6, 298 K) of $\mathbf{1A^{Br}}$.

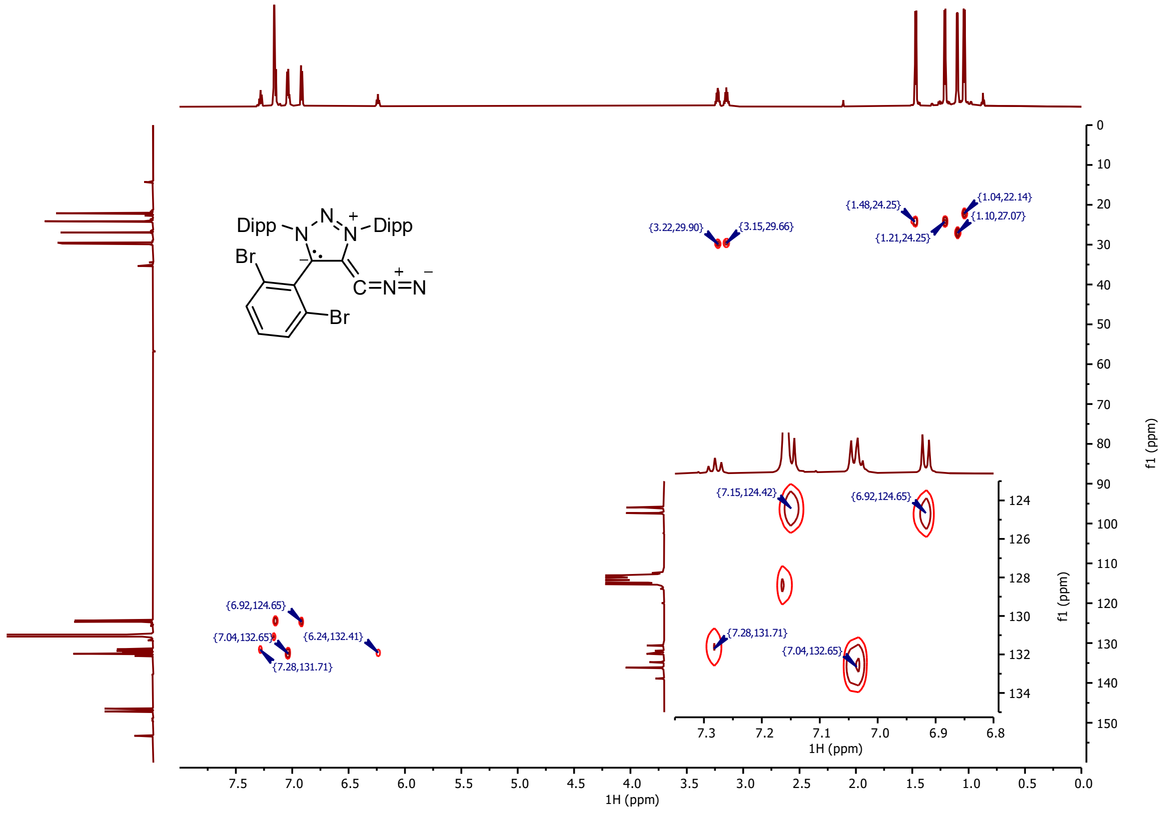

Figure S1.46. ^{1}H/^{13}C HSQC (700/176 MHz, C_6D_6, 298 K) of $\mathbf{1A^{Br}}$.

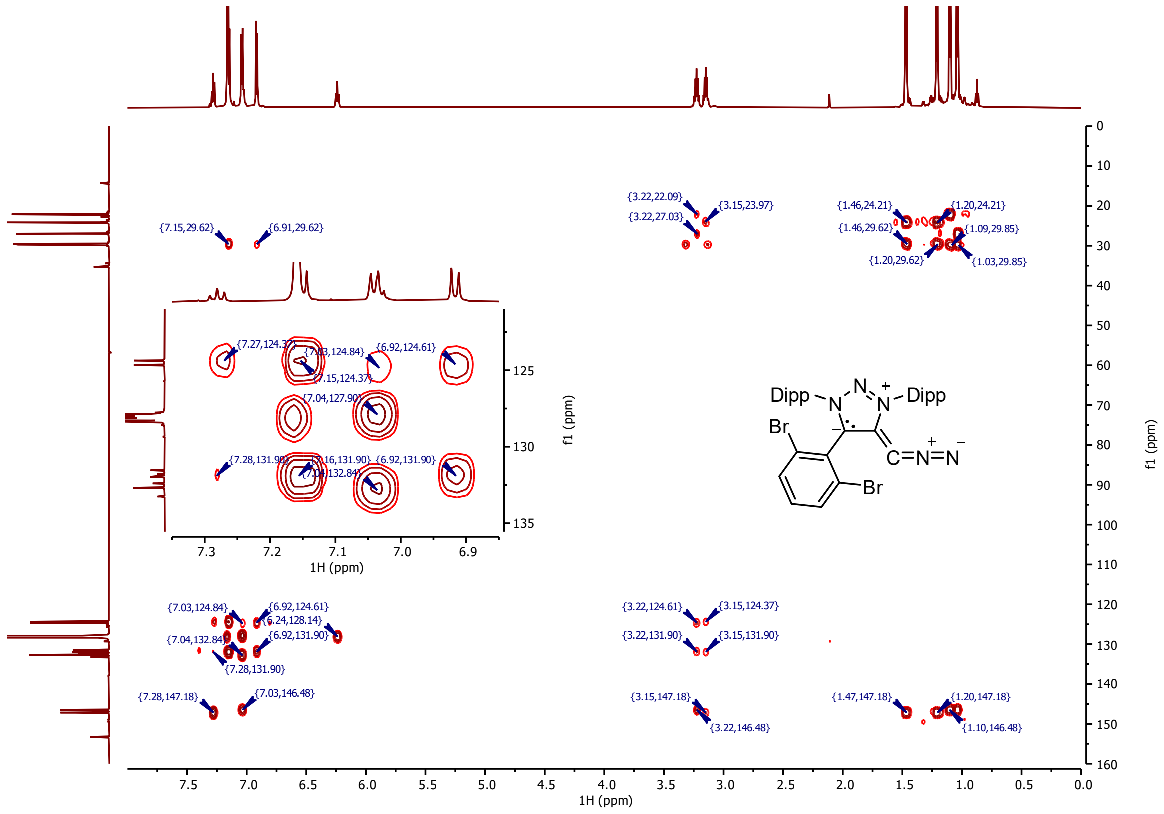

Figure S1.47. **^{1}H/^{13}C HSQC** (700/176 MHz, C_6D_6, 298 K) of **1A^{Br}**.

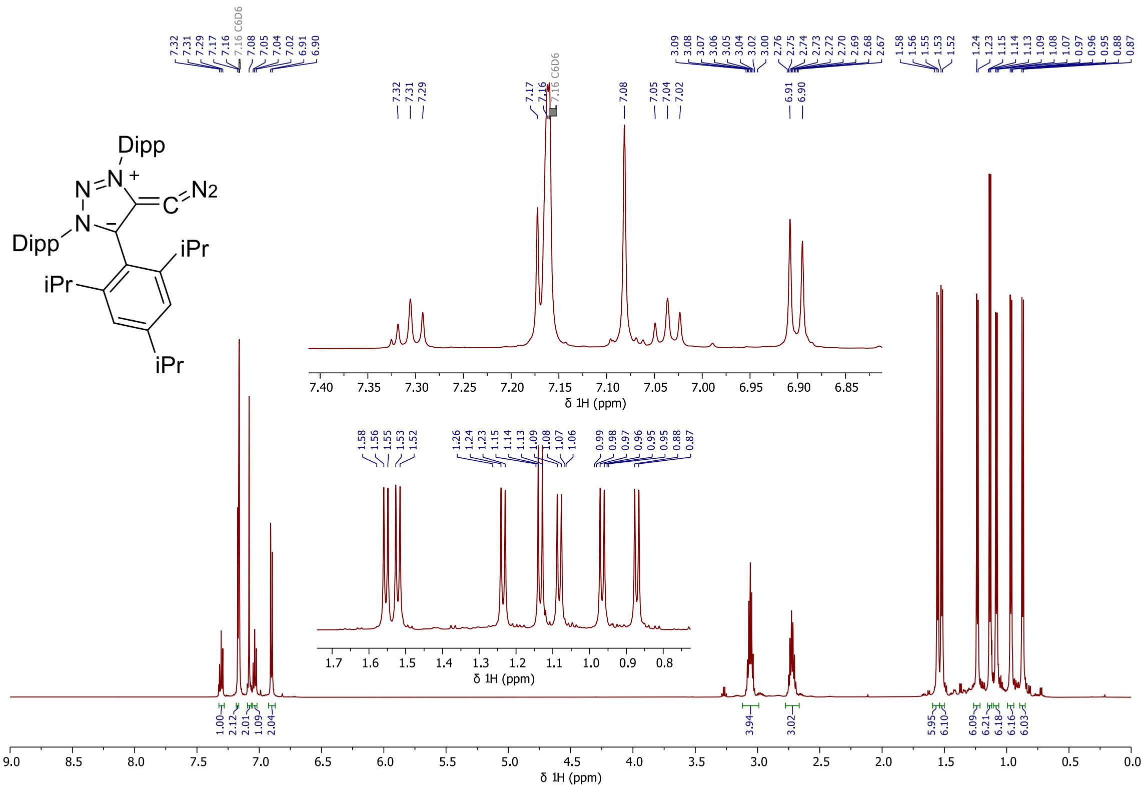

Figure S1.48. **^{1}H NMR** (600 MHz, C_6D_6, 298 K) of **1A^{iPr}**.

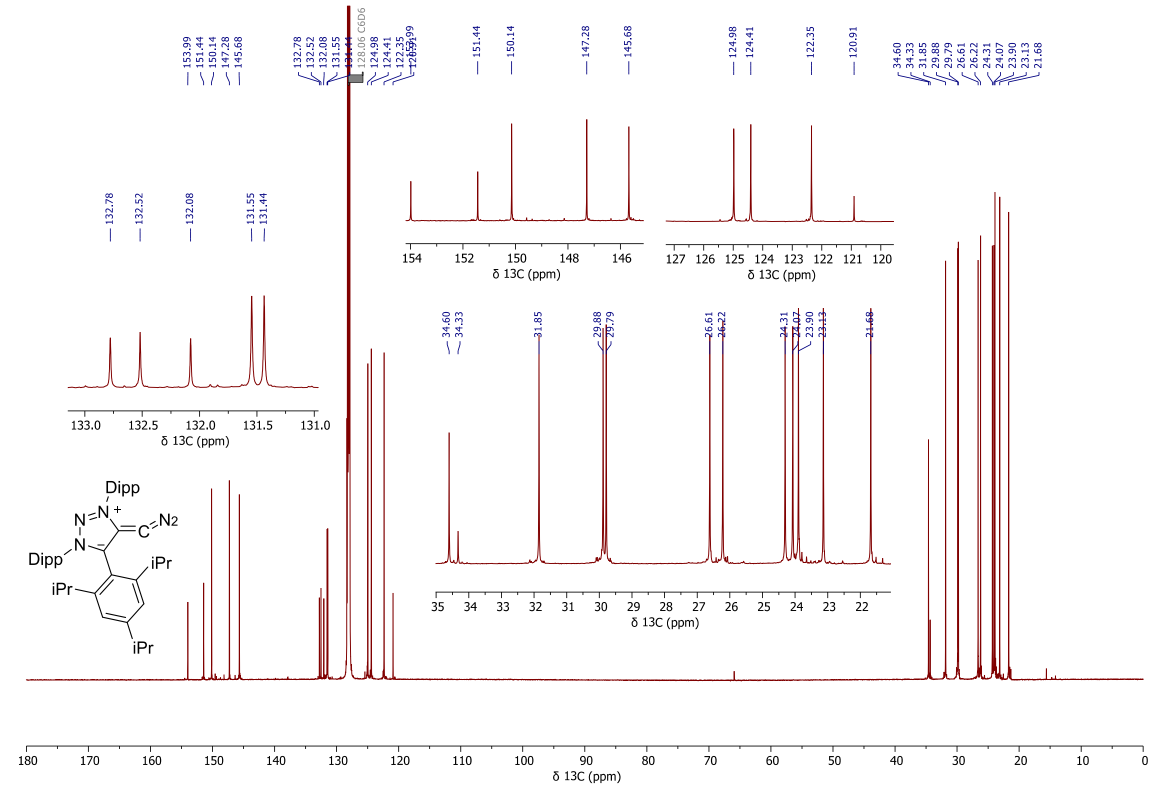

Figure S1.49. ^{13}**C NMR** (151 MHz, C_6D_6, 298 K) of **$1A^{iPr}$**.

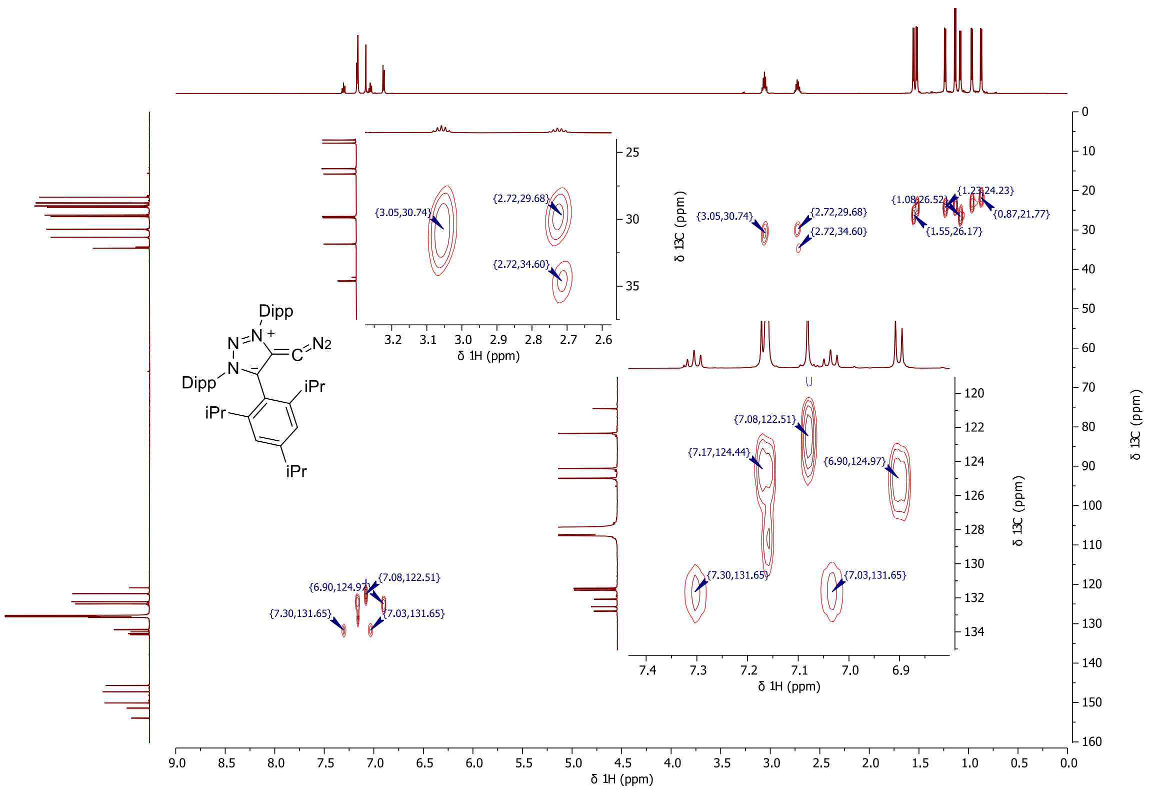

Figure S1.50. ^{1}**H/**^{13}**C HSQC** (500/126 MHz, C_6D_6, 298 K) of **$1A^{iPr}$**.

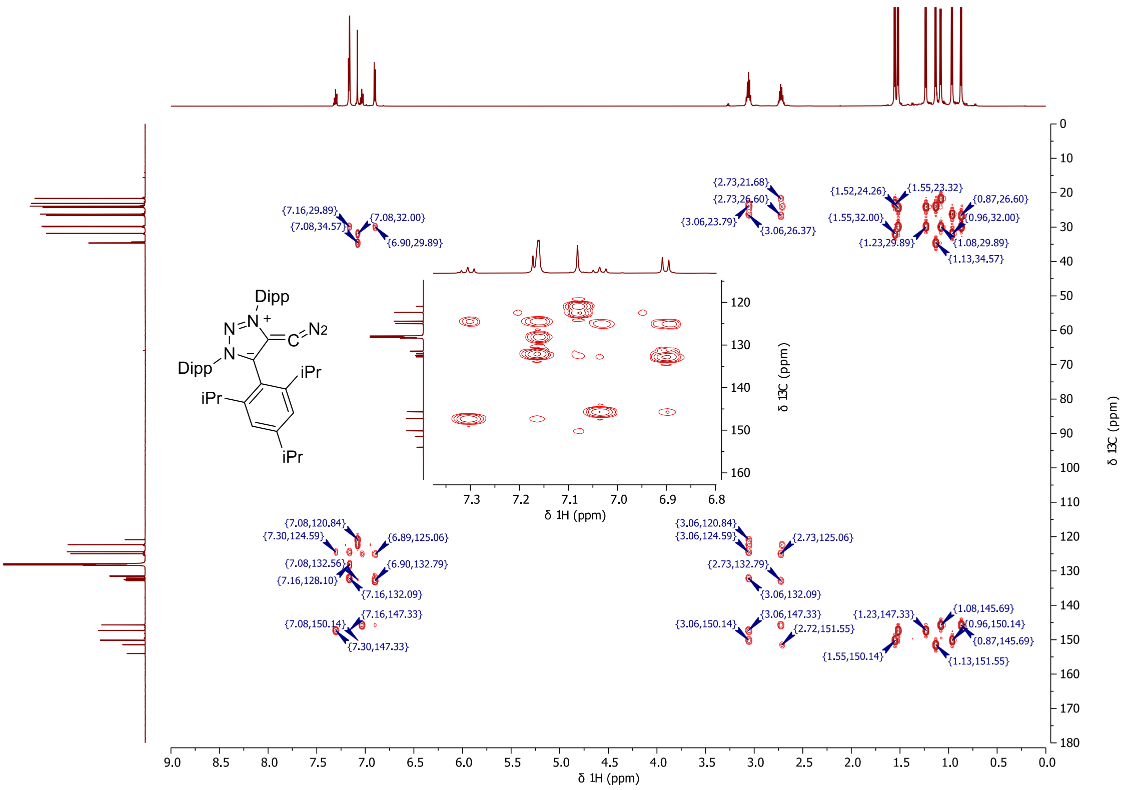

Figure S1.51. ^{1}**H/**^{13}**C HMBC** (500/126 MHz, C_6D_6, 298 K) of **1A^{iPr}**.

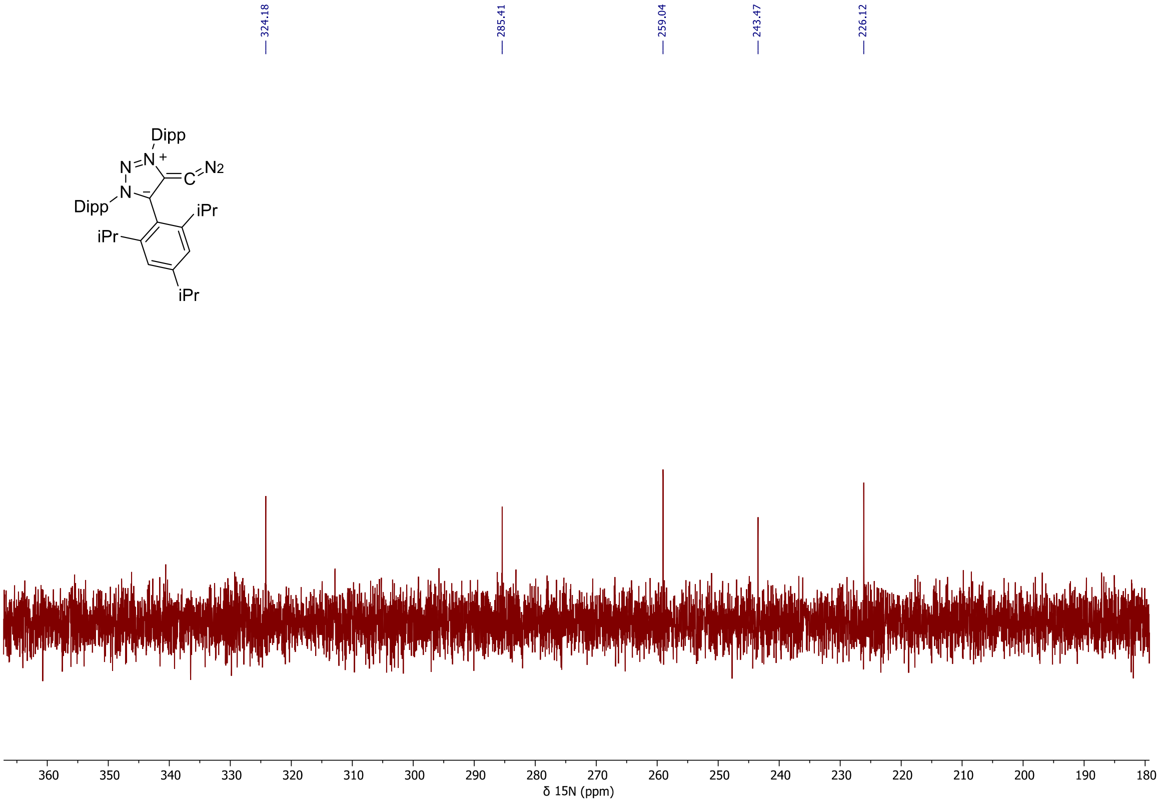

Figure S1.52. ^{15}**N NMR** (61 MHz, C_6D_6, 298 K) of **1A^{iPr}**.

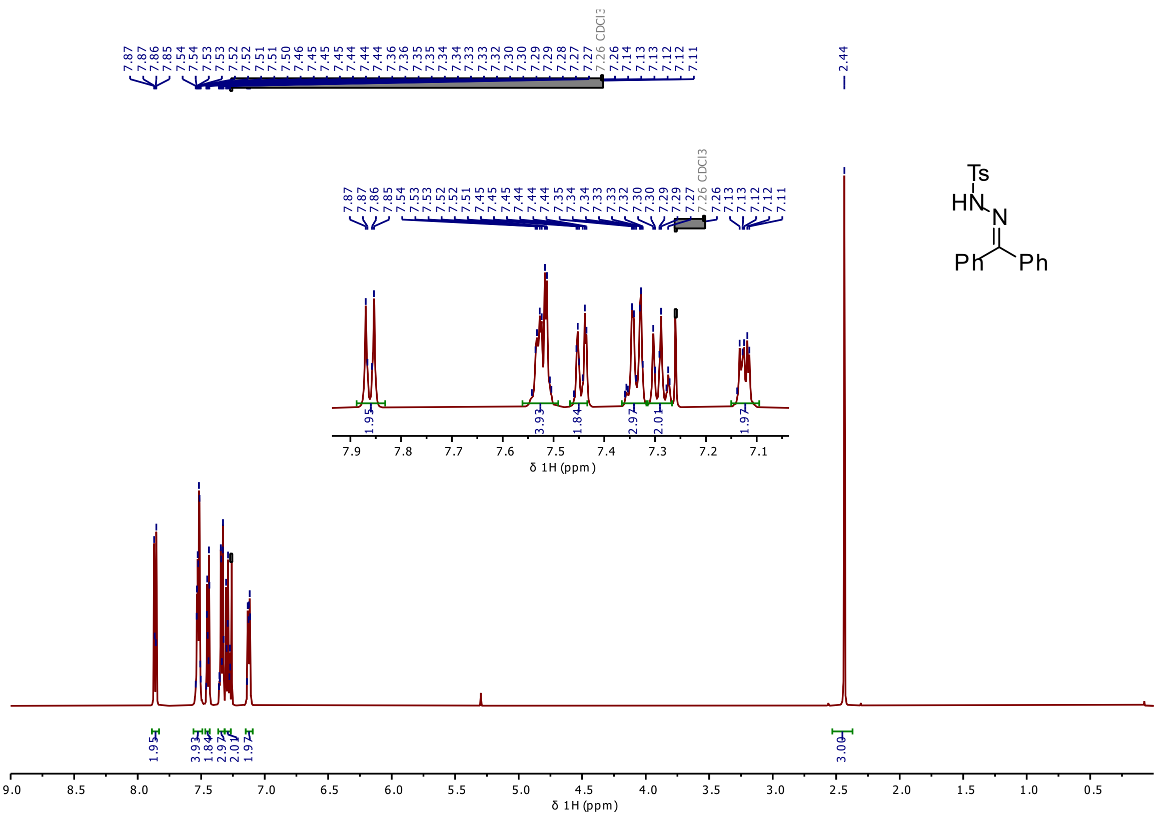

Figure S1.53. ^{1}**H NMR** (500 MHz, $CDCl_3$, 298 K) of **benzophenone tosylhydrazone**.

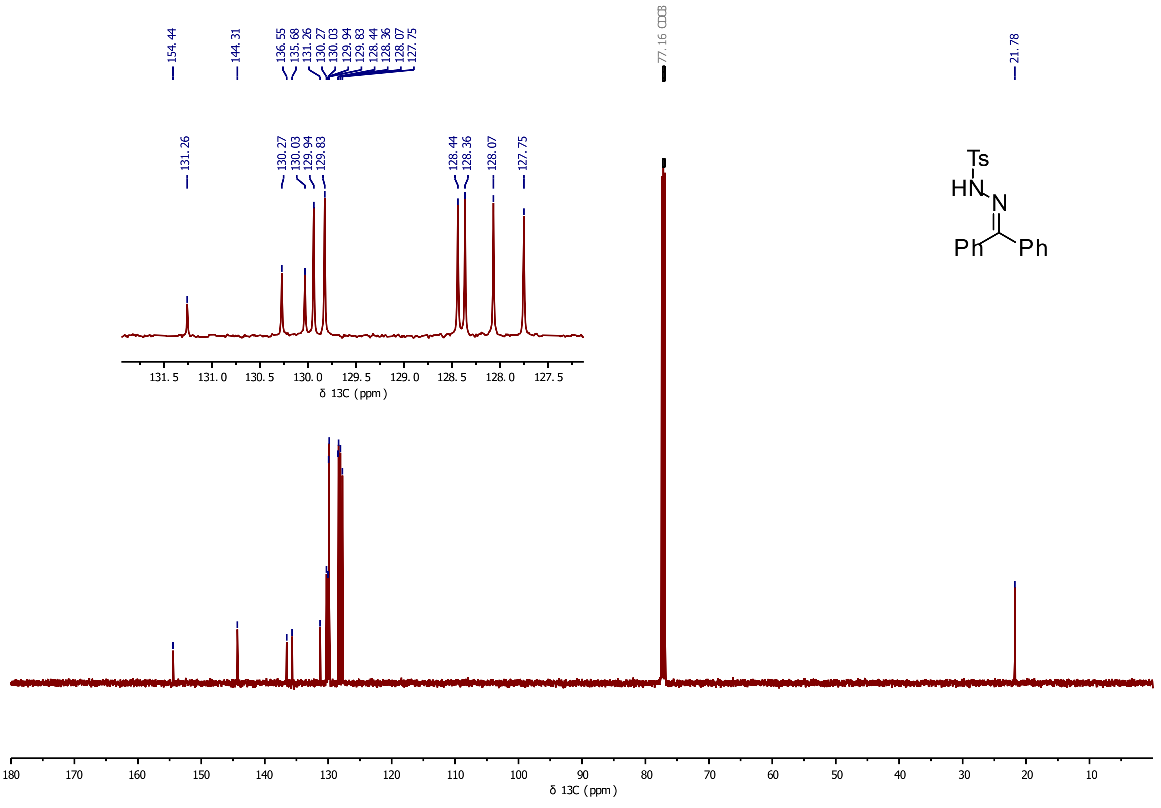

Figure S1.54. ^{13}**C NMR** (126 MHz, $CDCl_3$, 298 K) of **benzophenone tosylhydrazone**.

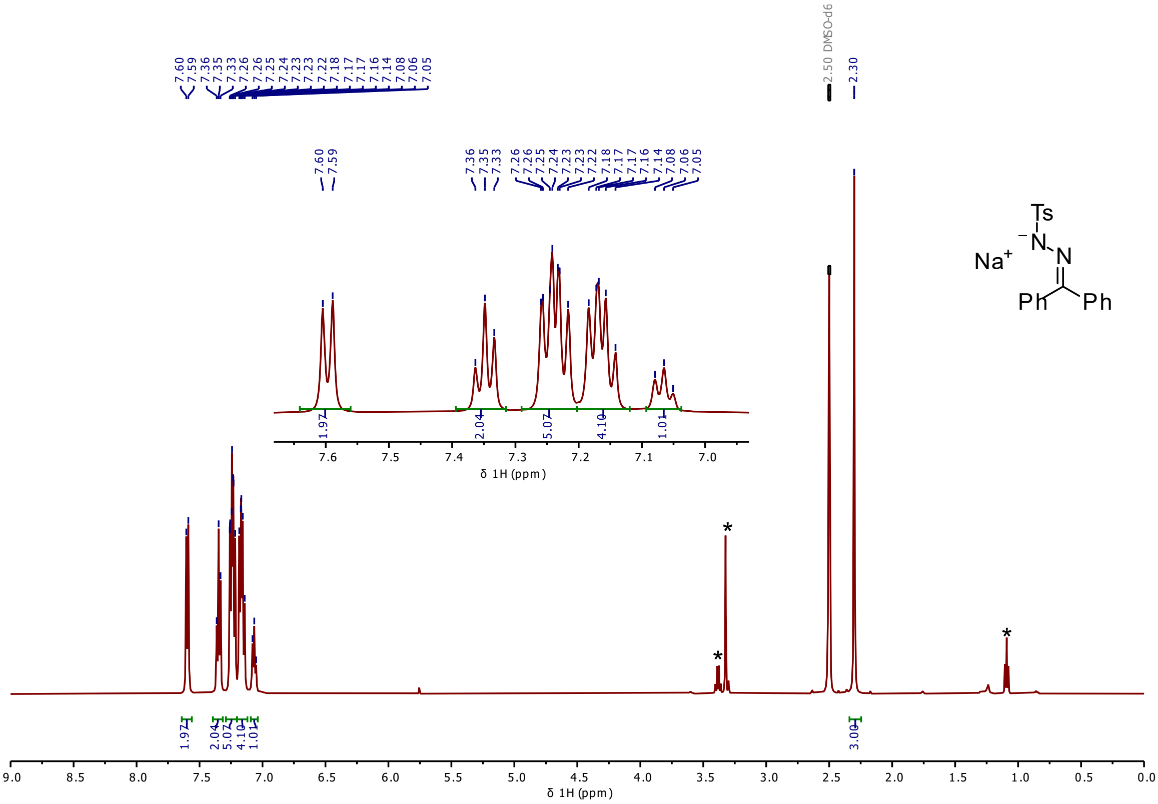

Figure S1.55. ^{1}**H NMR** (500 MHz, d_6-DMSO, 298 K) of **benzophenone tosylhydrazone sodium salt** (asterisks mark H_2O and Et_2O).

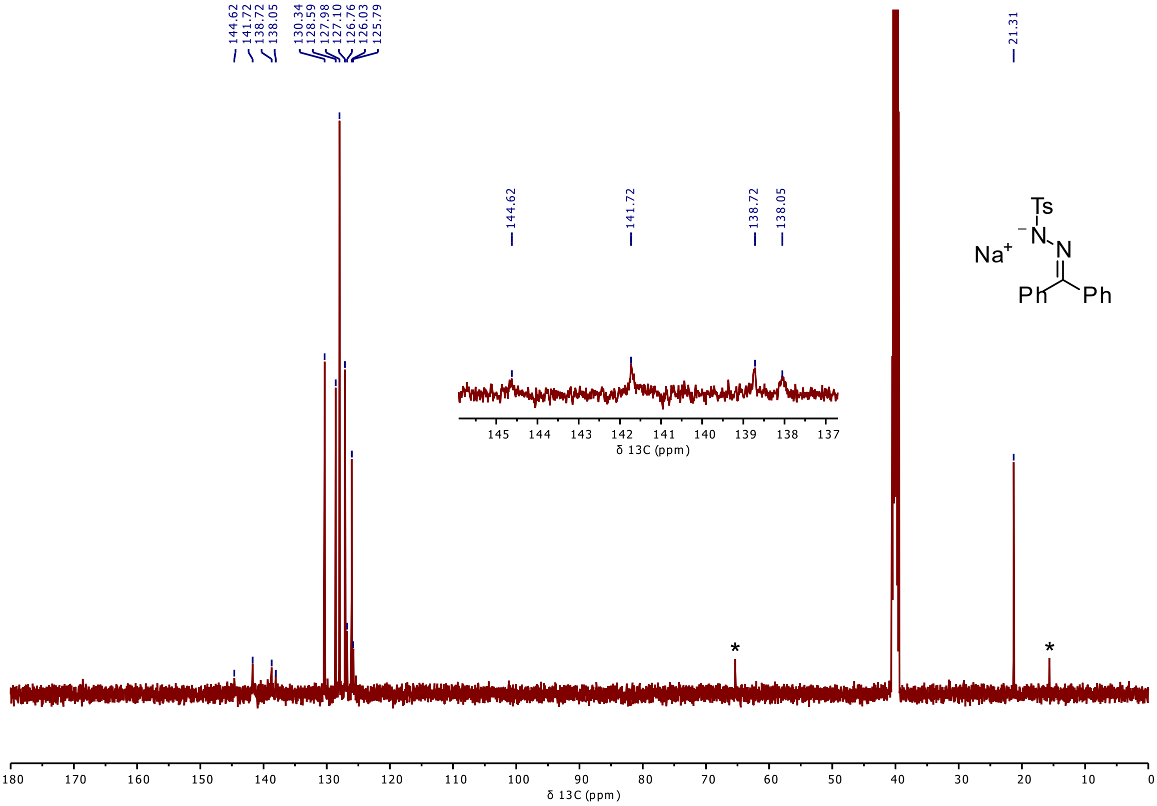

Figure S1.56. ^{13}**C NMR** (126 MHz, d_6-DMSO, 298 K) of **benzophenone tosylhydrazone sodium salt** (asterisks mark Et_2O).

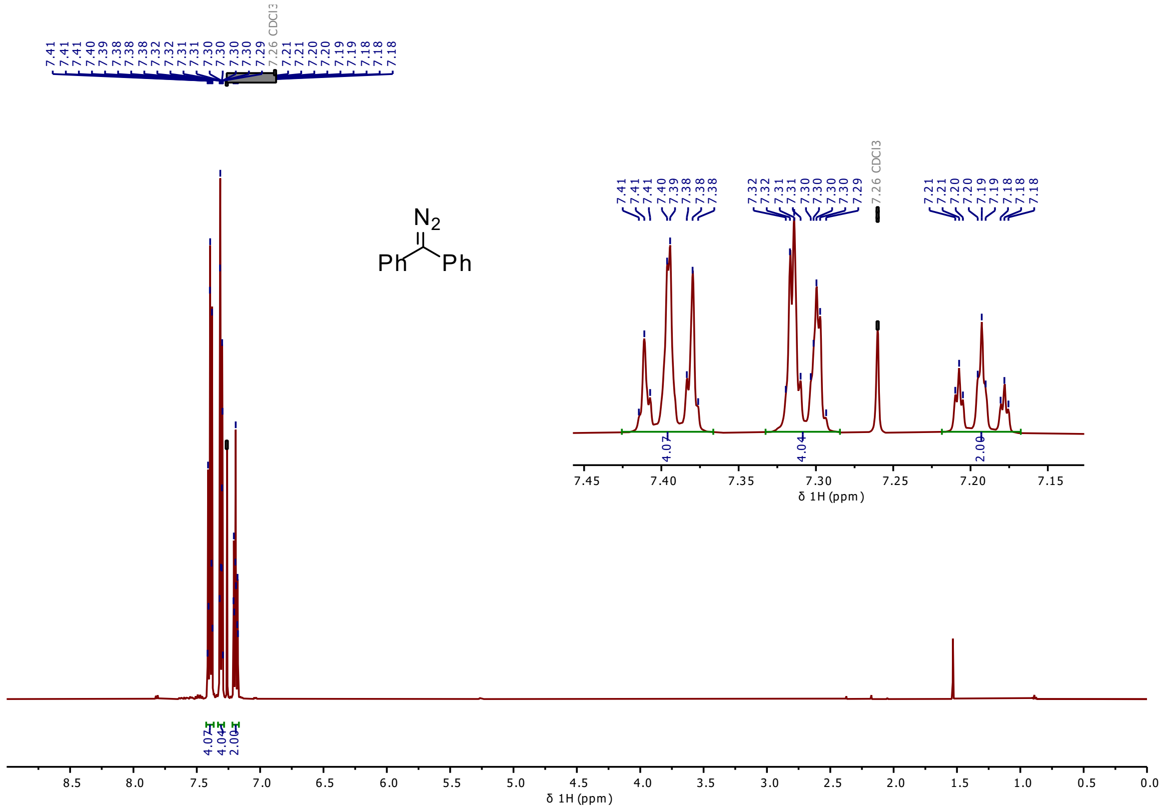

Figure S1.57. **¹H NMR** (500 MHz, $CDCl_3$, 298 K) of **diphenyl diazomethane**.

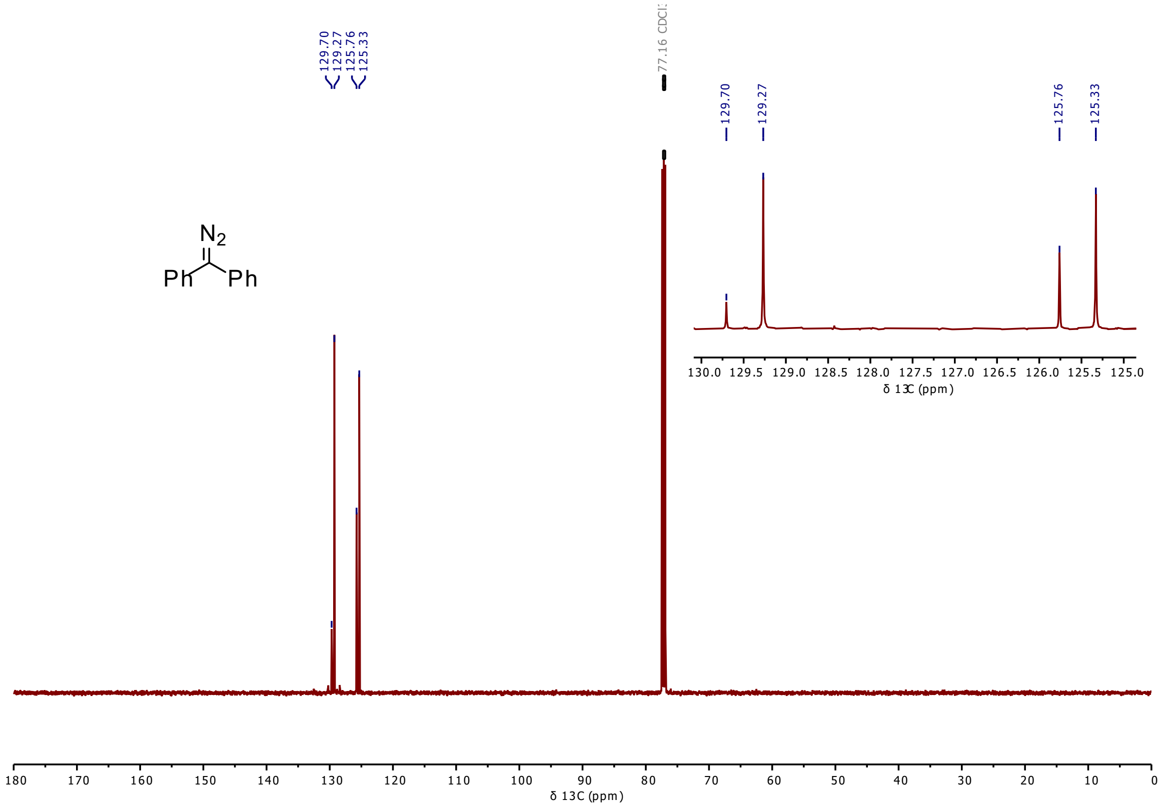

Figure S1.58. **¹³C NMR** (126 MHz, $CDCl_3$, 298 K) of **diphenyl diazomethane**.

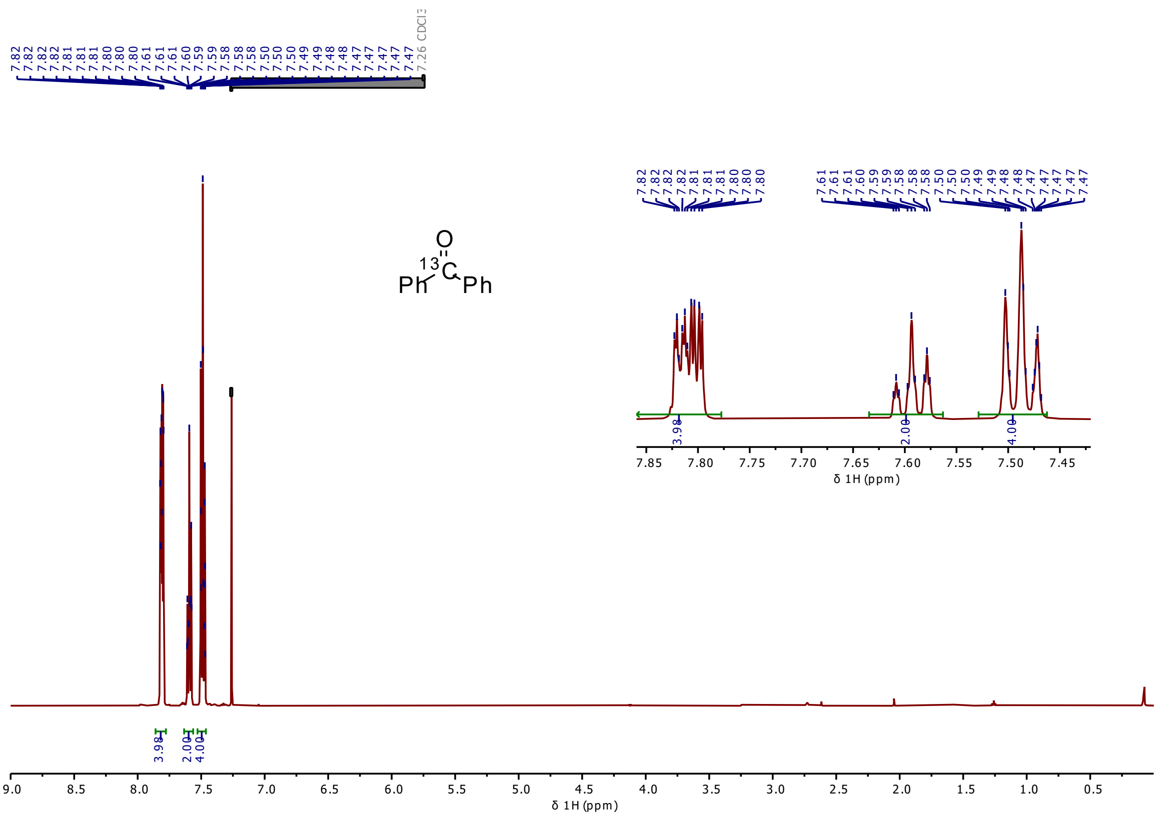

Figure S1.59. ^{1}**H NMR** (500 MHz, $CDCl_3$, 298 K) of ^{13}**C-labeled benzophenone**.

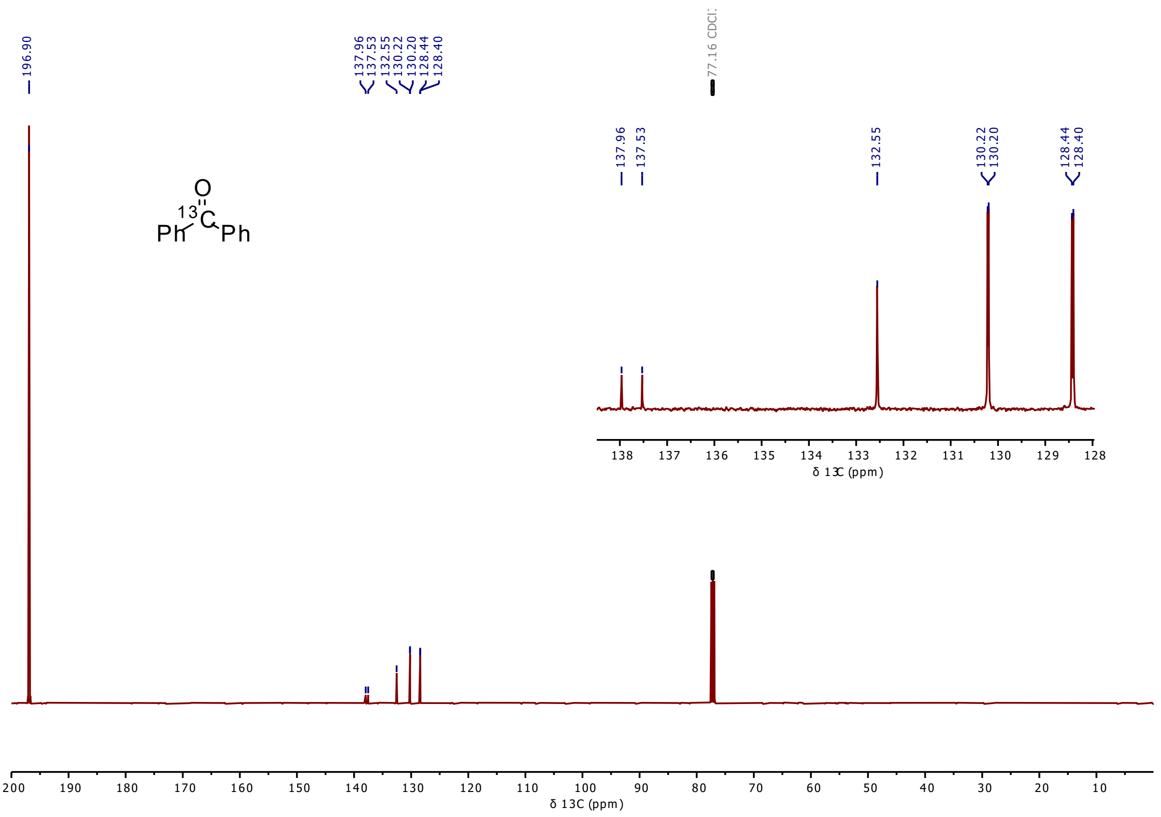

Figure S1.60. ^{13}**C NMR** (126 MHz, $CDCl_3$, 298 K) of ^{13}**C-labeled benzophenone**.

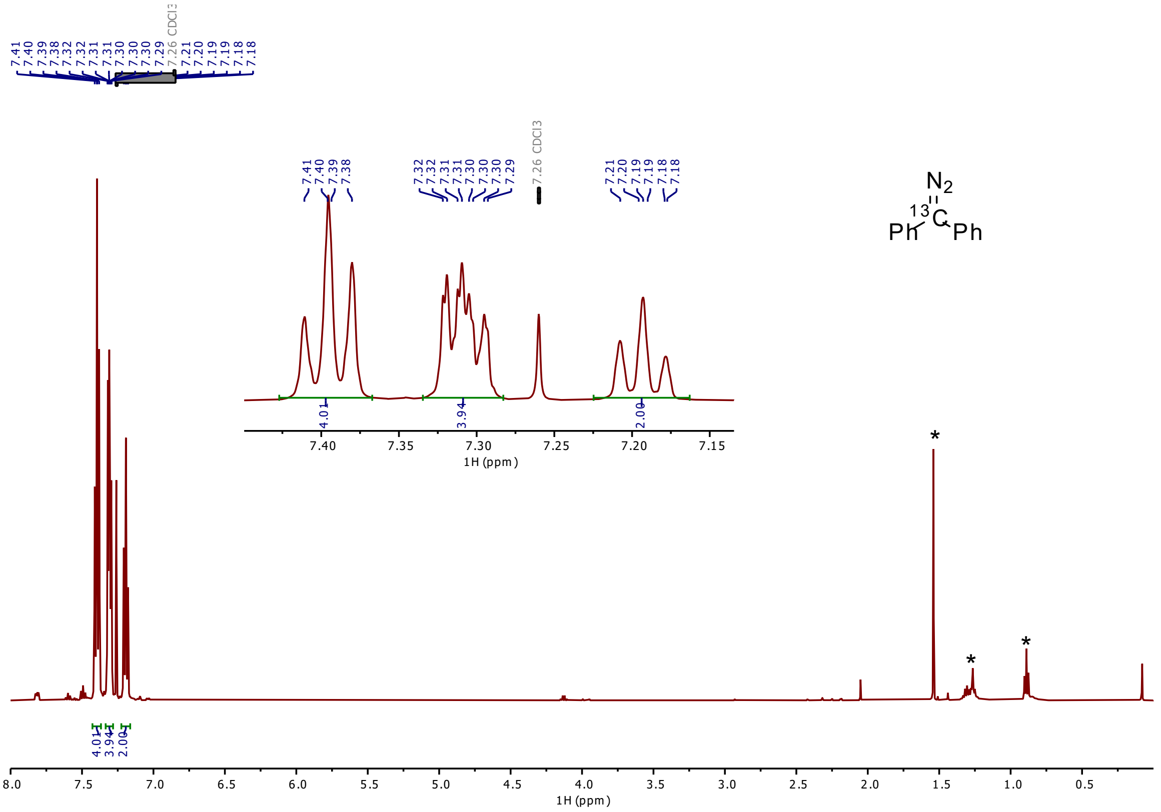

Figure S1.61. **^{1}H NMR** (501 MHz, $CDCl_3$, 298 K) of **^{13}C-diphenyl diazomethane** (asterisks mark pentane and H_2O).

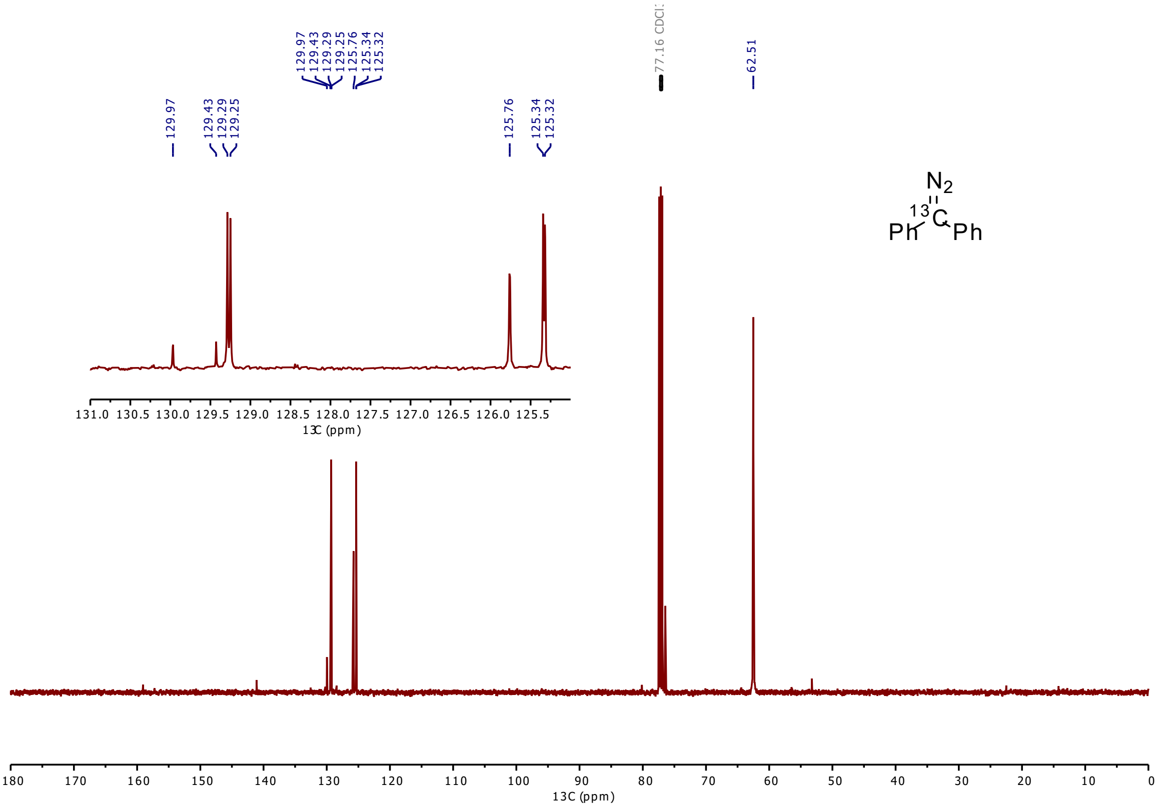

Figure S1.62. **^{13}C NMR** (126 MHz, $CDCl_3$, 298 K) of **^{13}C-diphenyl diazomethane**.

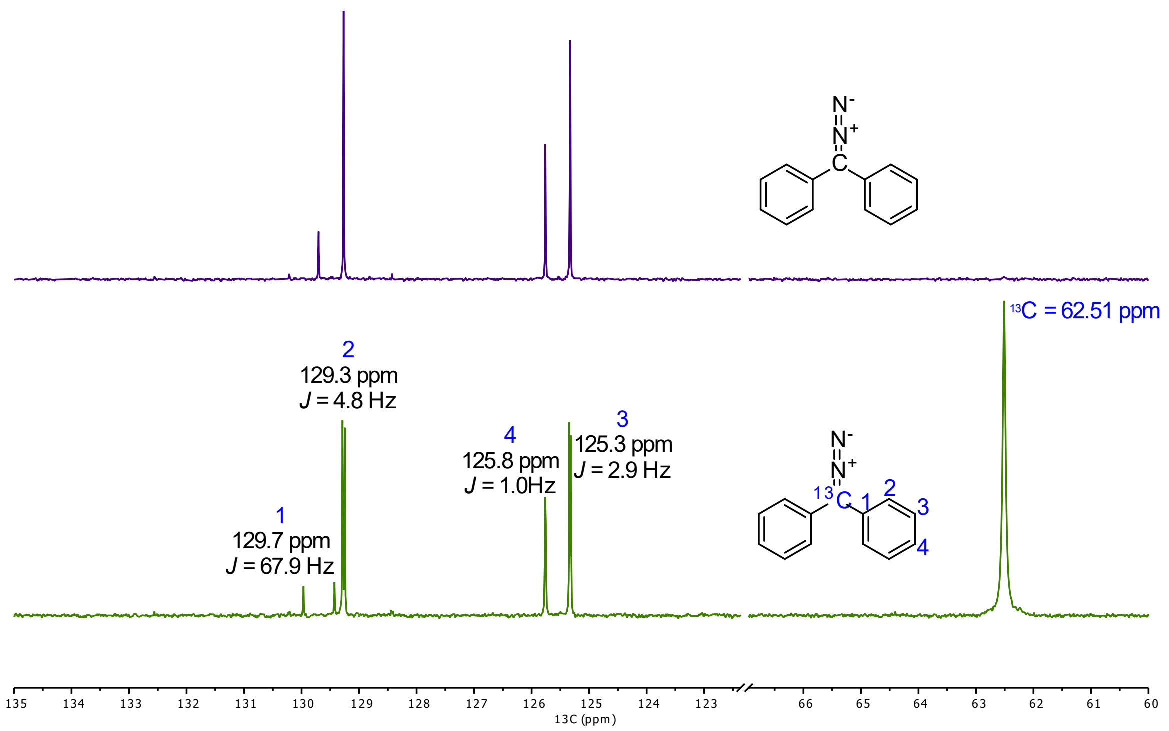

Figure S1.63. Stacked [13]**C NMR** (126 MHz, $CDCl_3$, 298 K) of **diphenyl diazomethane** (top) and [13]**C-diphenyl diazomethane** (bottom).

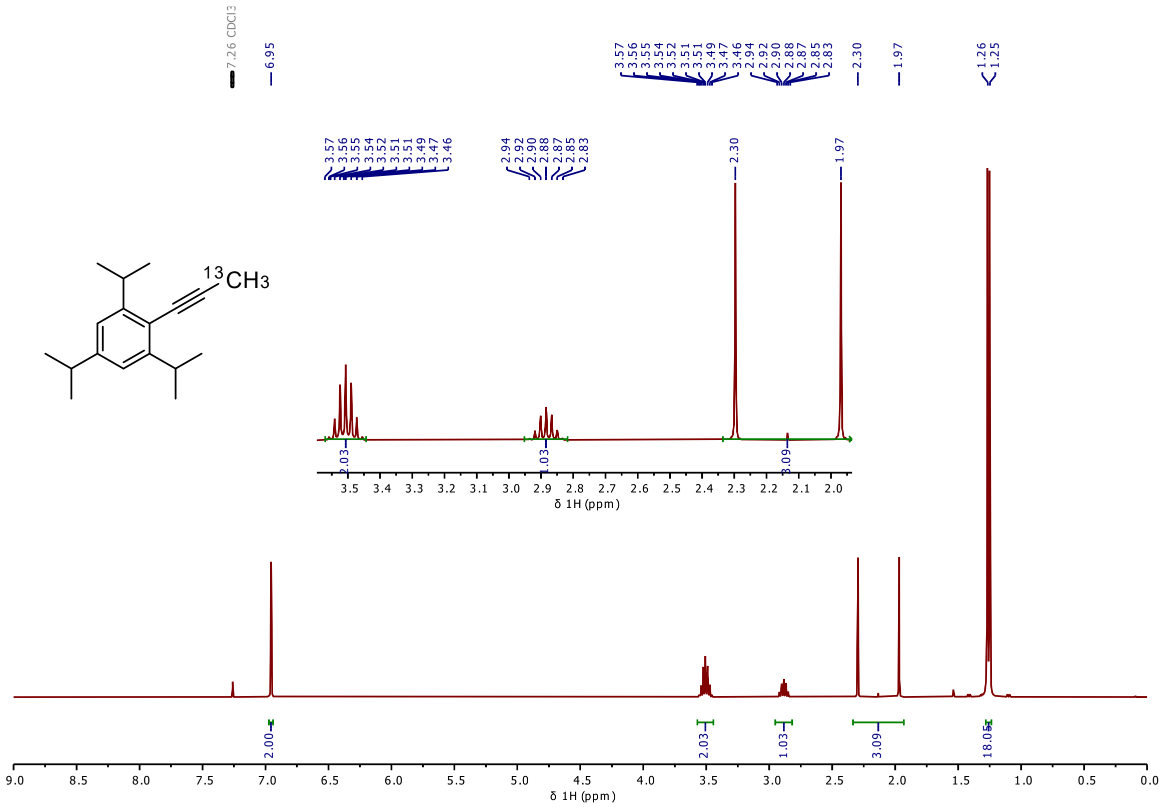

Figure S1.64. [1]**H NMR** (400 MHz, $CDCl_3$, 298 K) of [13]**C-1,3,5-triisopropyl-2-(prop-1-yn-1-yl)benzene**.

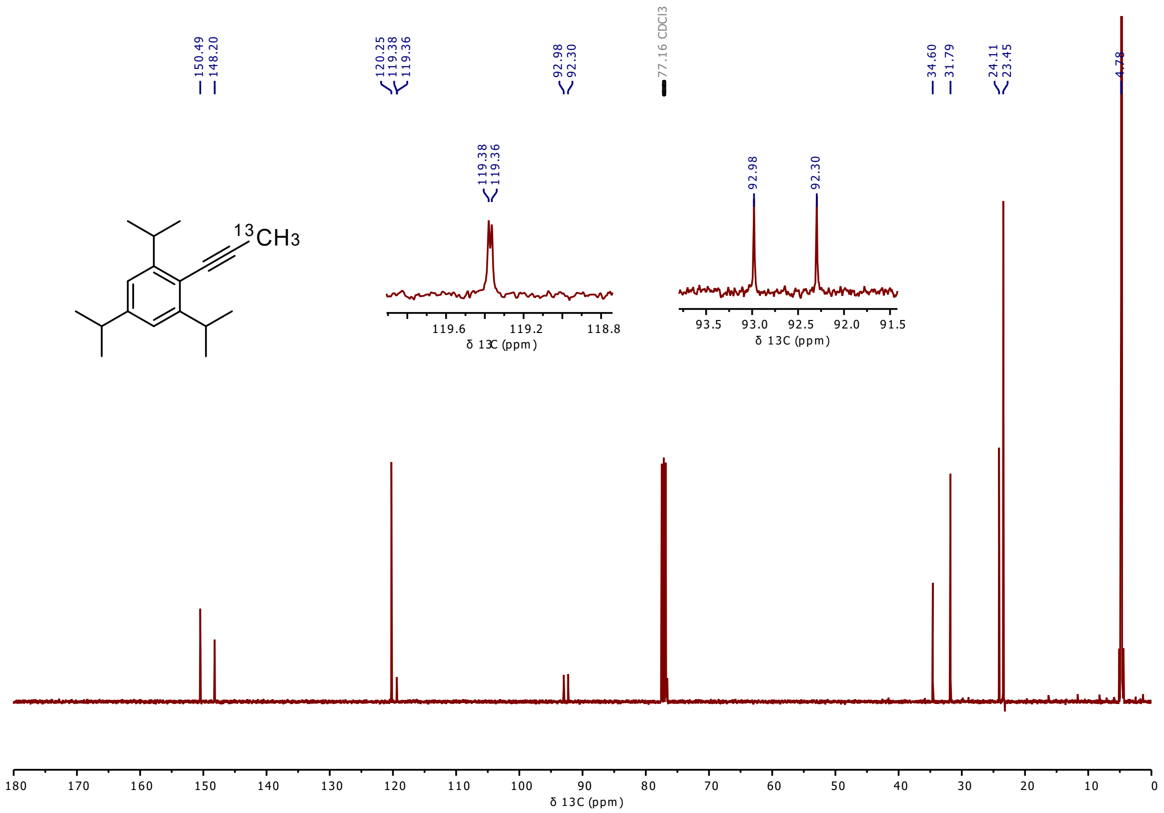

Figure S1.65. **^{13}C NMR** (100 MHz, $CDCl_3$, 298 K) of **^{13}C-1,3,5-triisopropyl-2-(prop-1-yn-1-yl)benzene**.

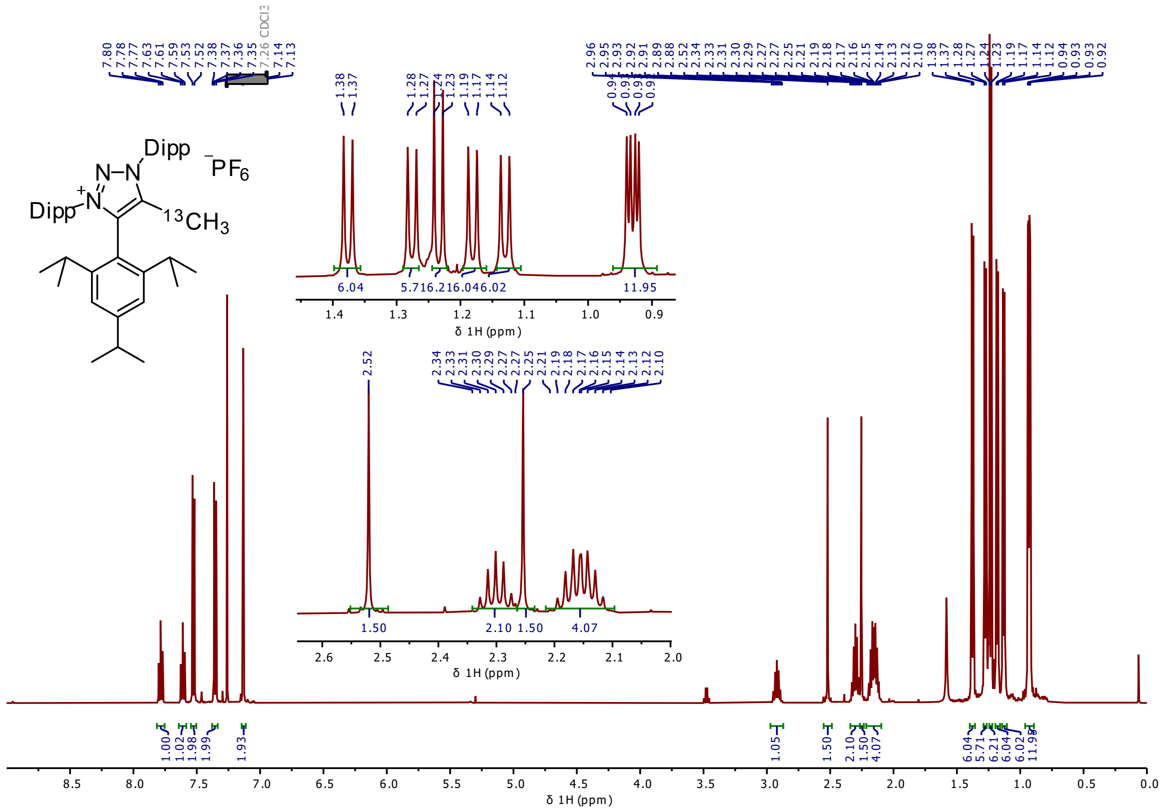

Figure S1.66. **^{1}H NMR** (500 MHz, $CDCl_3$, 298 K) of **^{13}C-Tripp-triazolium salt**.

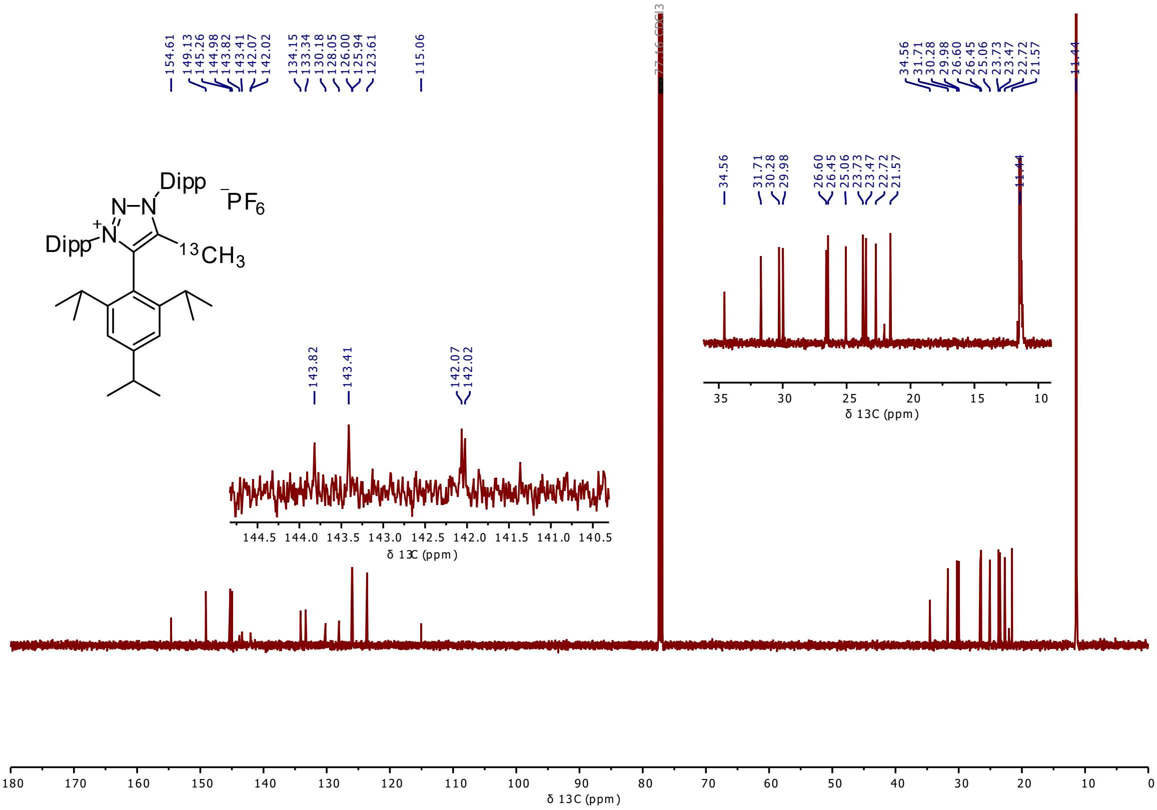

Figure S1.67. **^{13}C NMR** (125 MHz, $CDCl_3$, 298 K) of **^{13}C-Tripp-triazolium salt**.

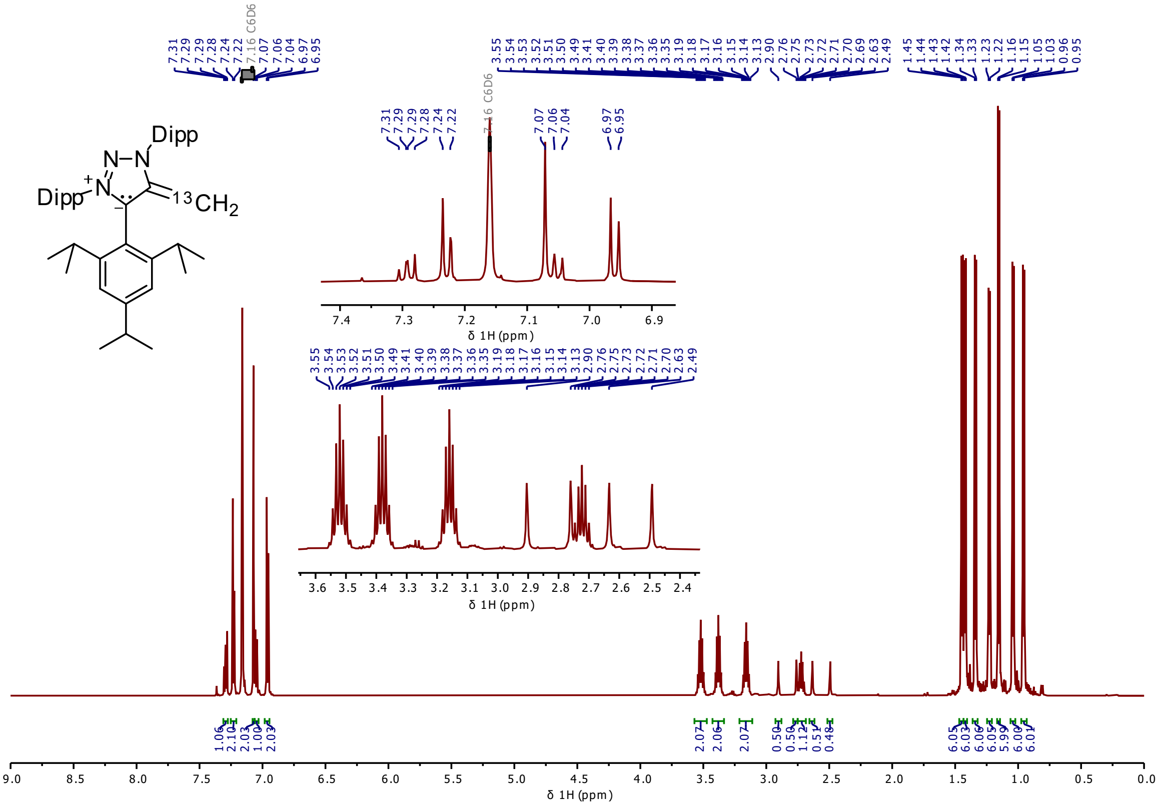

Figure S1.68. **^{1}H NMR** (600 MHz, C_6D_6, 298 K) of **^{13}C-Tripp-mNHO**.

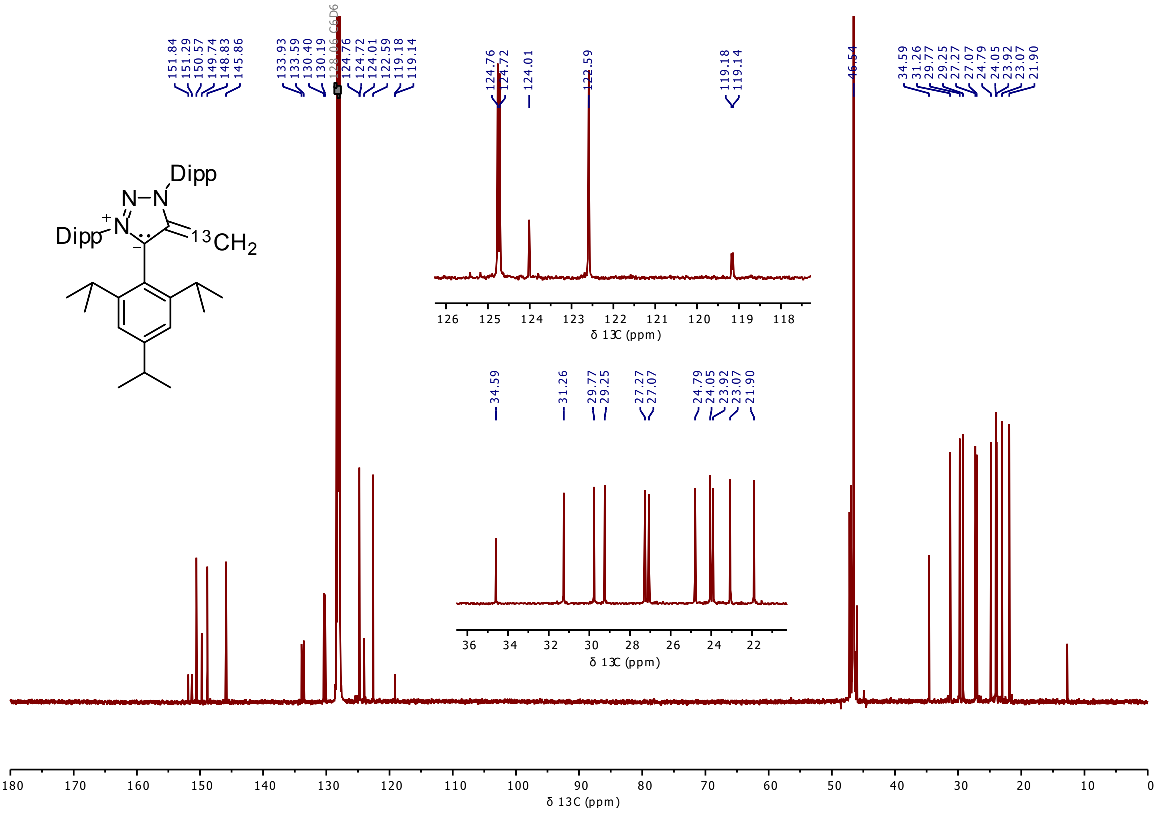

Figure S1.69. **^{13}C NMR** (151 MHz, C_6D_6, 298 K) of **^{13}C-Tripp-mNHO**.

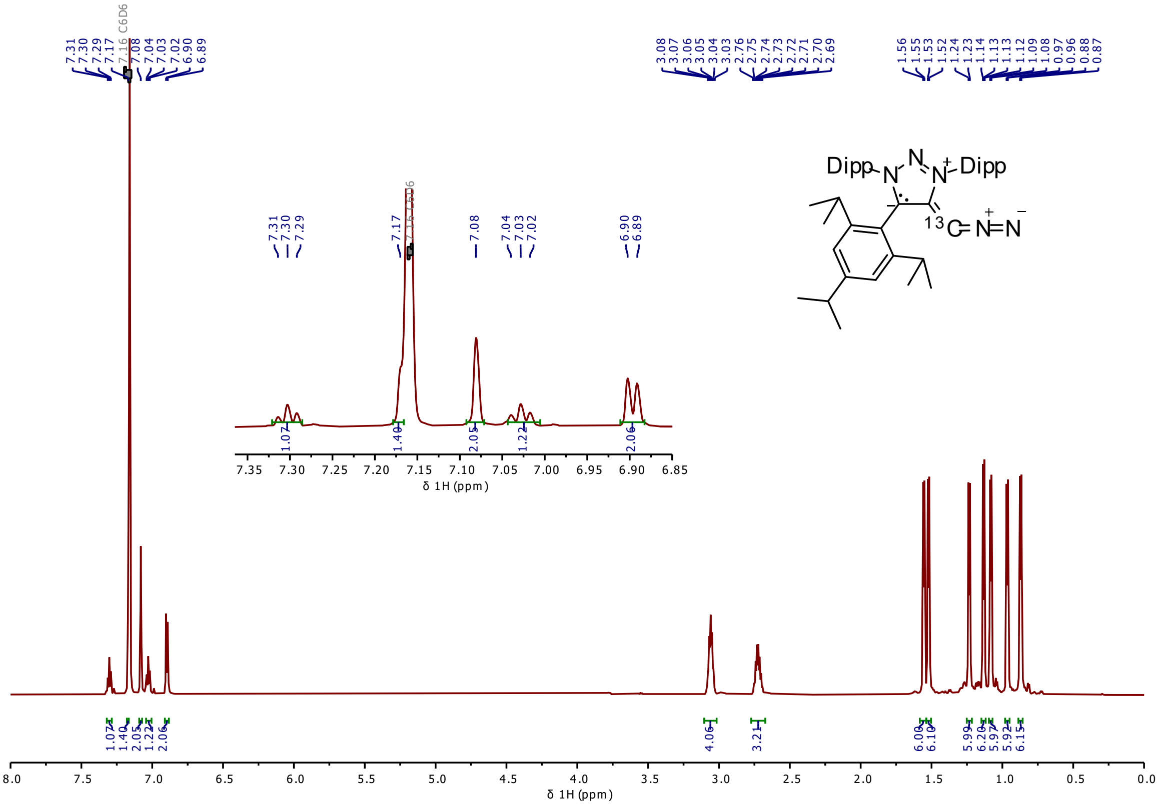

Figure S1.70. **^{1}H NMR** (700 MHz, C_6D_6, 298 K) of **^{13}C-1A^{iPr}**.

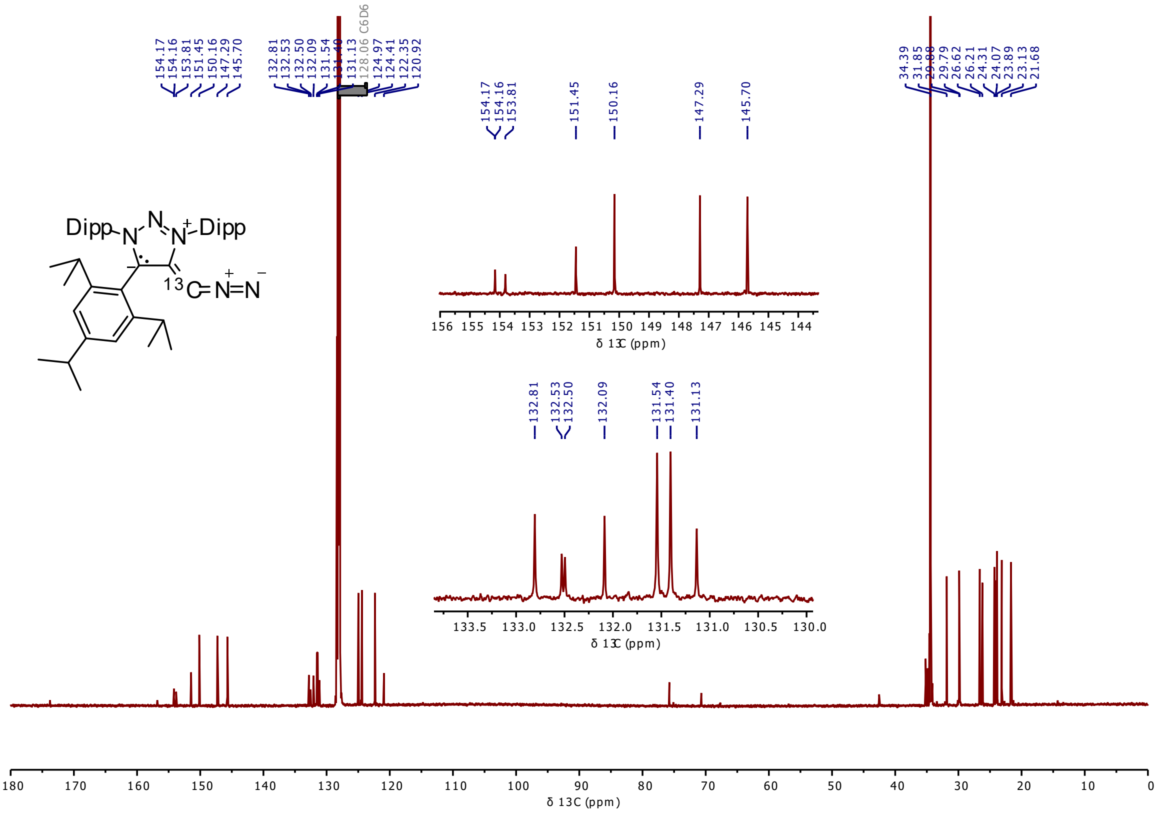

Figure S1.71. ^{13}**C NMR** (176 MHz, C_6D_6, 298 K) of ^{13}**C-1A^{iPr}**.

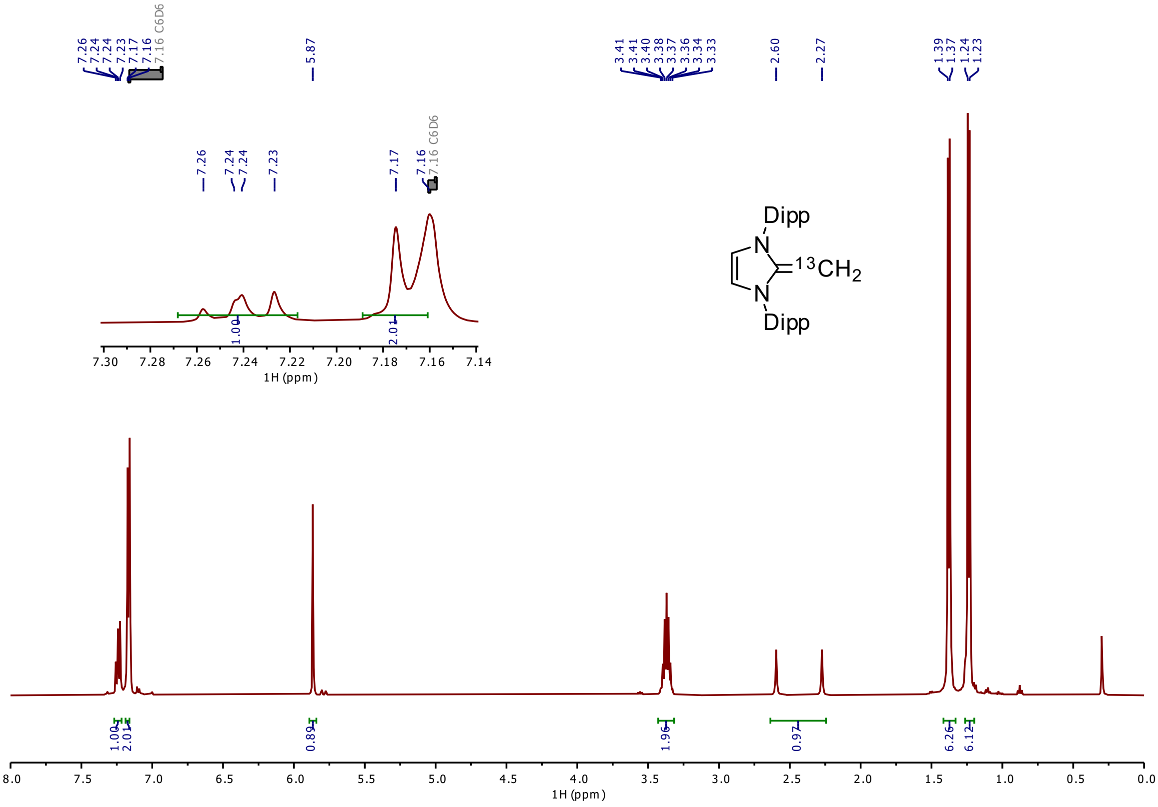

Figure S1.72. ^{1}**H NMR** (501 MHz, C_6D_6, 298 K) of ^{13}**C-IPr=CH$_2$**.

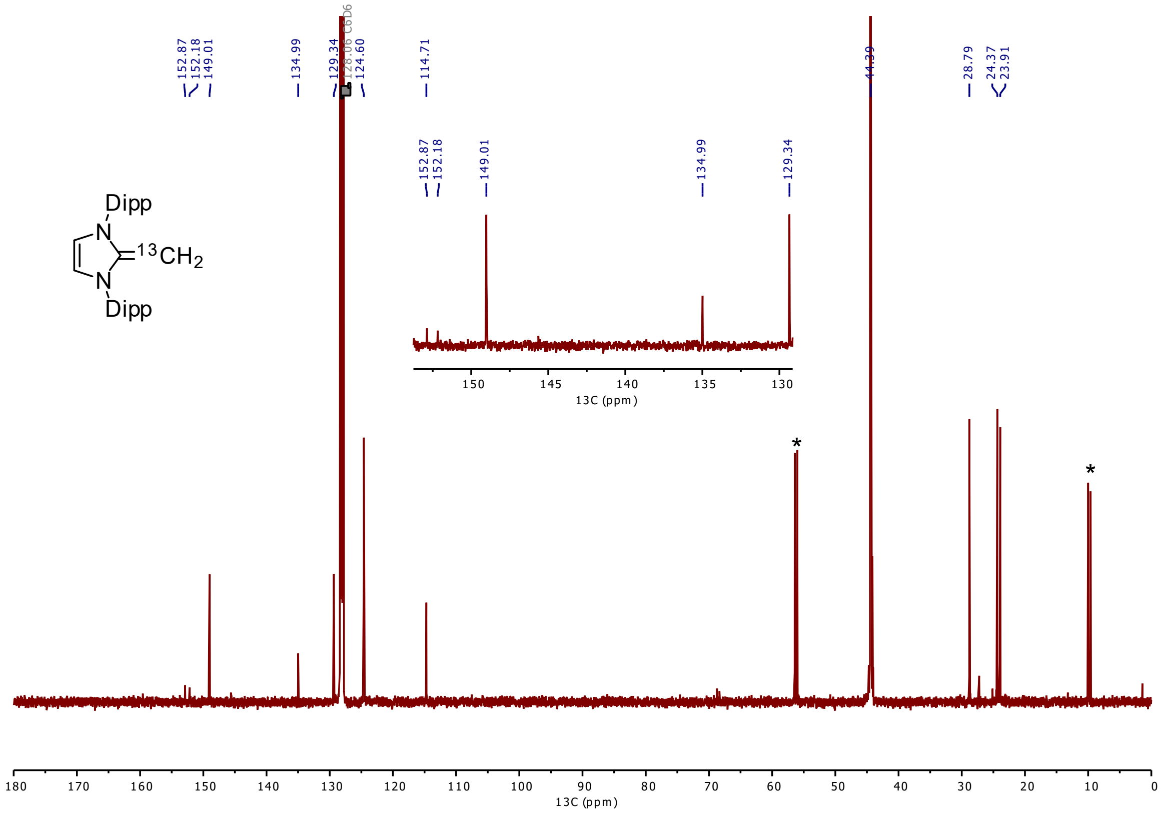

Figure S1.73. **^{13}C NMR** (126 MHz, C_6D_6, 298 K) of **^{13}C-IPr=CH$_2$** (asterisk mark minor amounts double^{13}C-labeled impurity, probably stemming from two times ^{13}C-methylated NHO, for comparison see ref [18]).

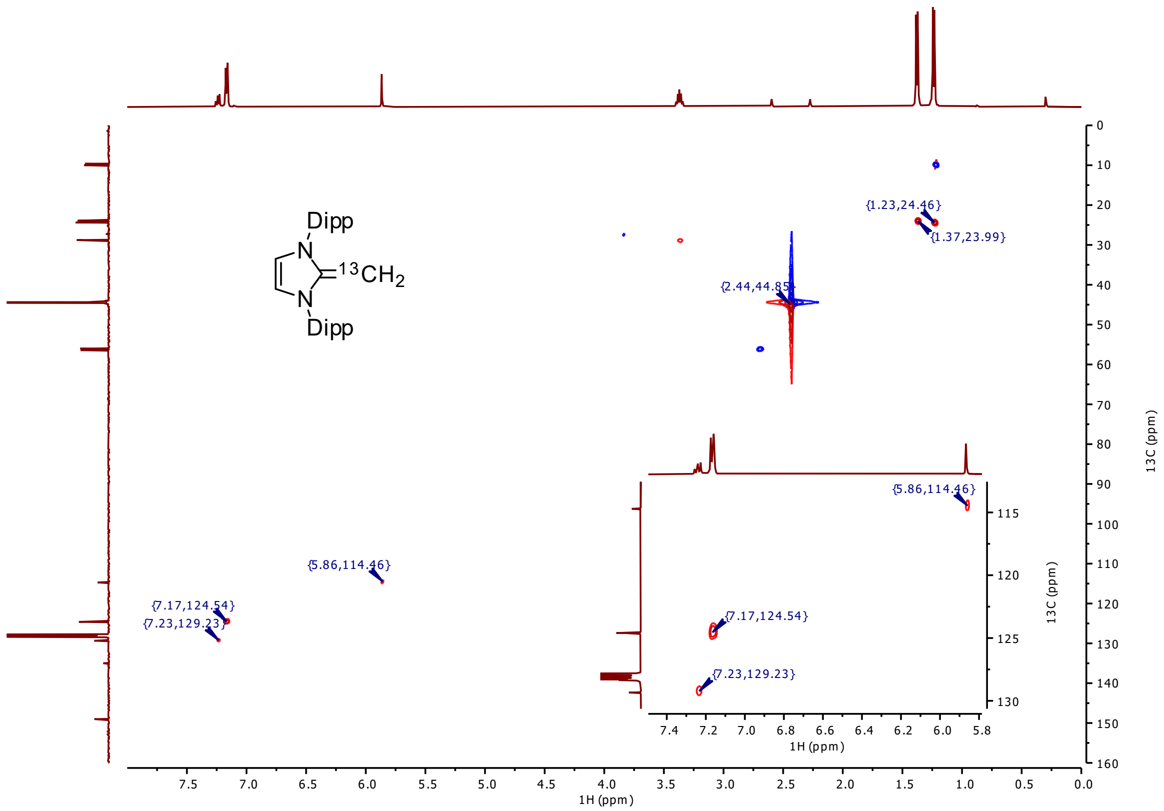

Figure S1.74. **^{1}H/^{13}C HSQC** (501/126 MHz, C_6D_6, 298 K) of **^{13}C-IPr=CH$_2$**.

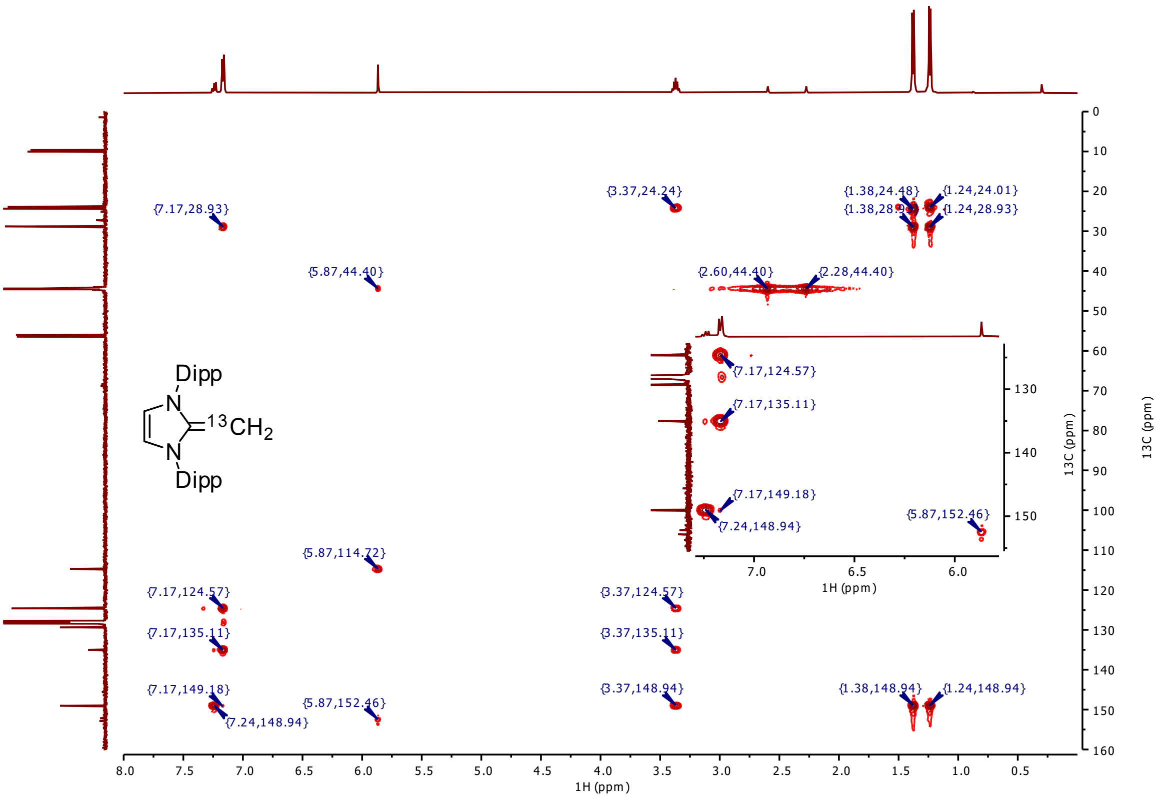

Figure S1.75. ^{1}**H/**^{13}**C HMBC** (501/126 MHz, C_6D_6, 298 K) of ^{13}**C-IPr=CH$_2$**.

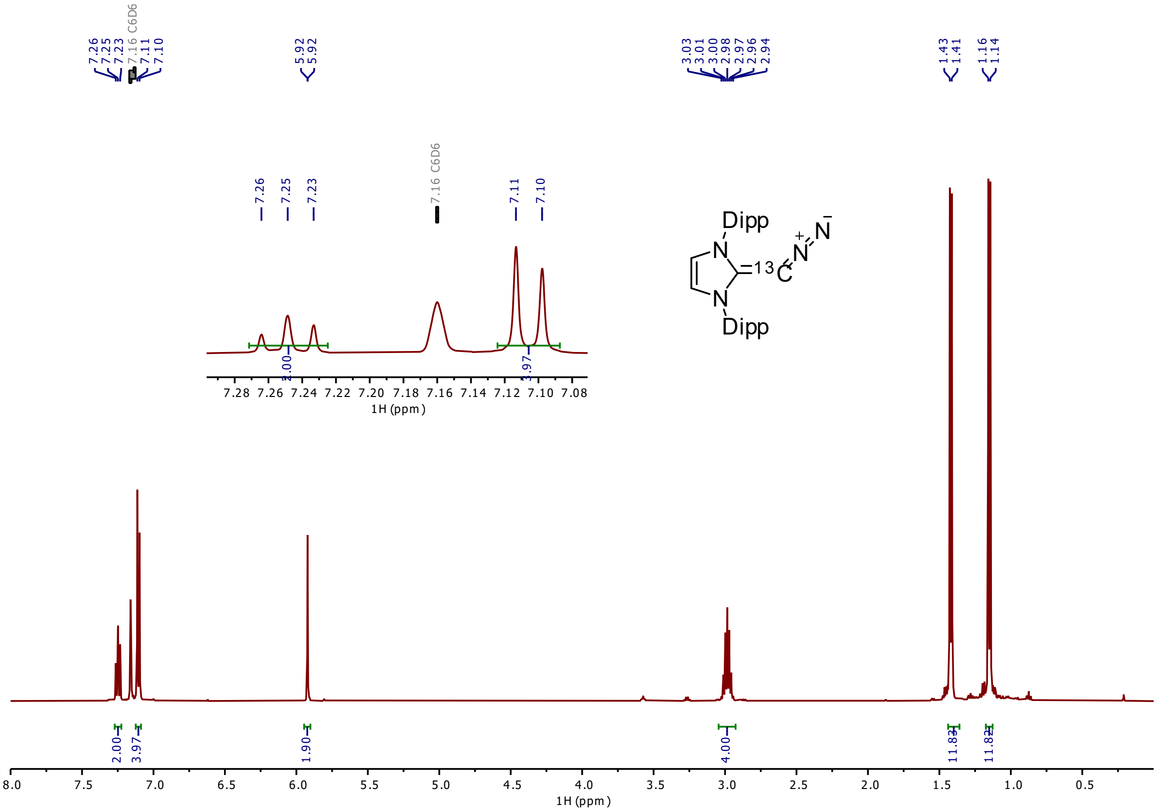

Figure S1.76. ^{1}**H NMR** (501 MHz, C_6D_6, 298 K) of ^{13}**C-1E**.

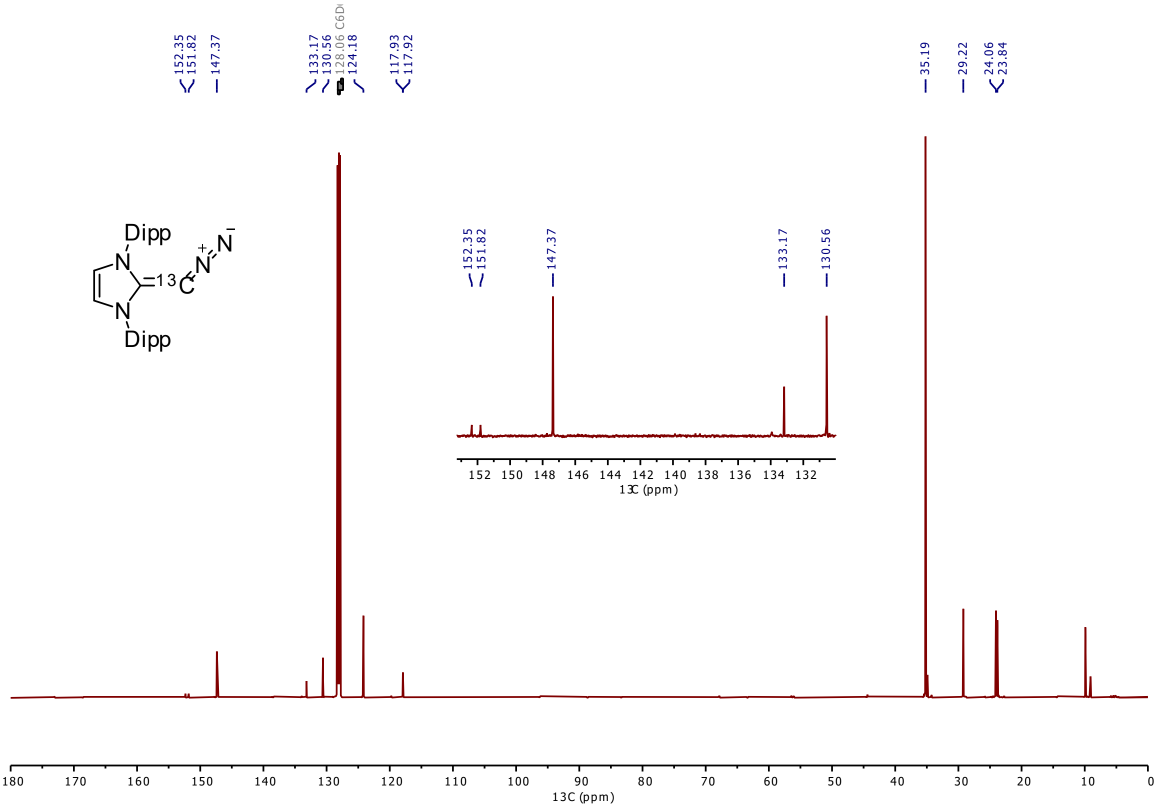

Figure S1.77. **^{13}C NMR** (126 MHz, C_6D_6, 298 K) of **^{13}C-1E**.

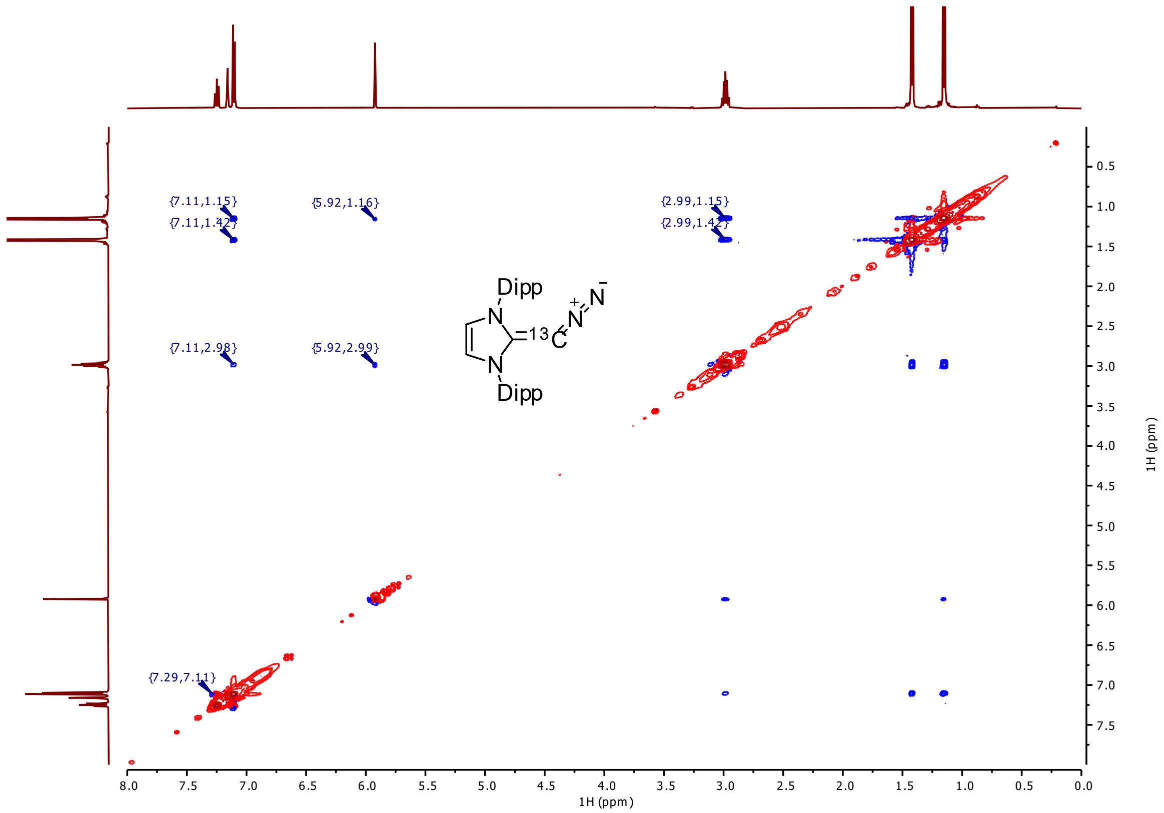

Figure S1.78. **^{1}H/^{1}H NOESY** (501/501 MHz, C_6D_6, 298 K) of **^{13}C-1E**.

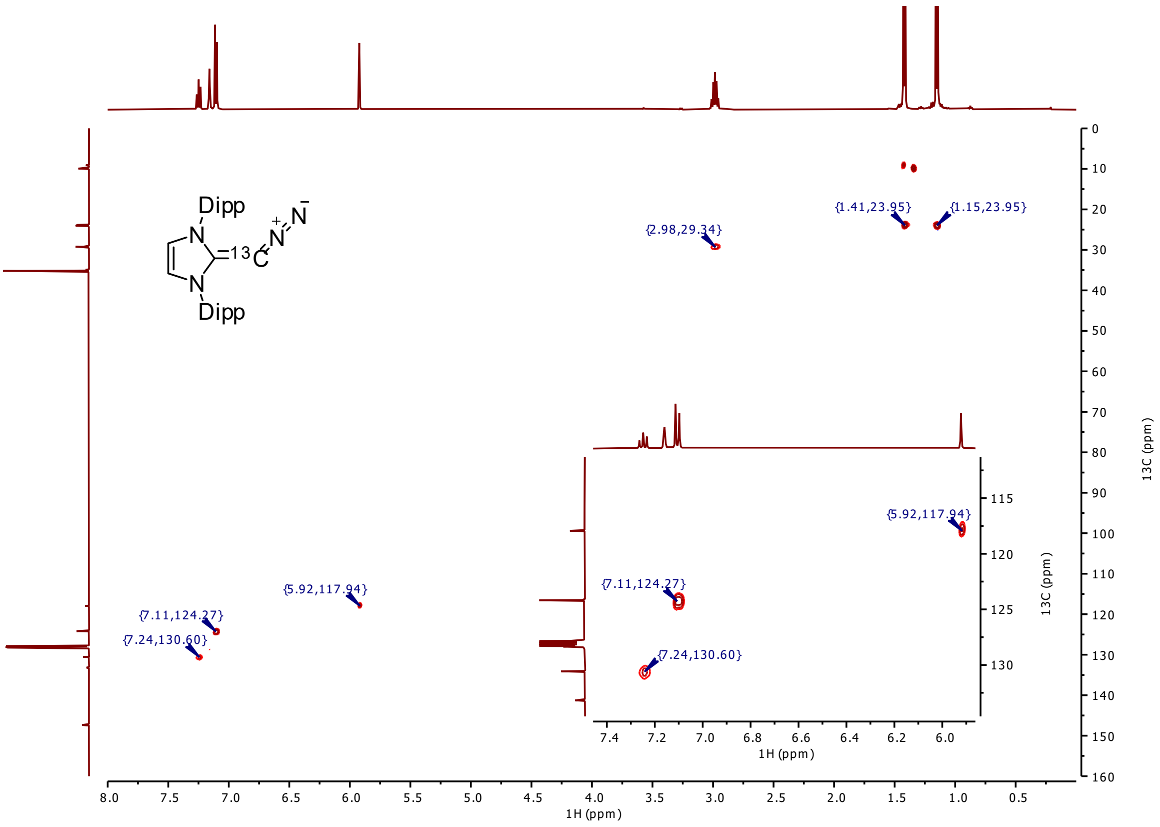

Figure S1.79. **^{1}H/^{13}C HSQC** (501/126 MHz, C_6D_6, 298 K) of **^{13}C-1E**.

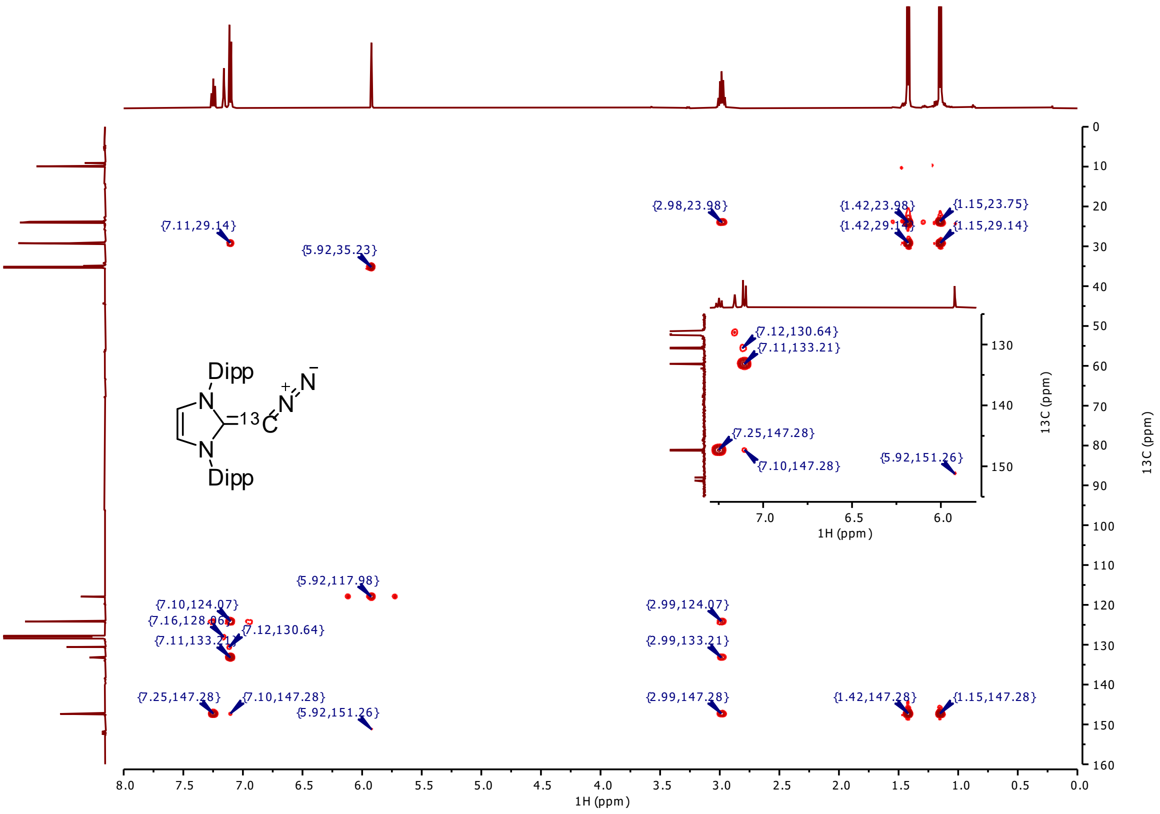

Figure S1.80. **^{1}H/^{13}C HMBC** (501/126 MHz, C_6D_6, 298 K) of **^{13}C-1E**.

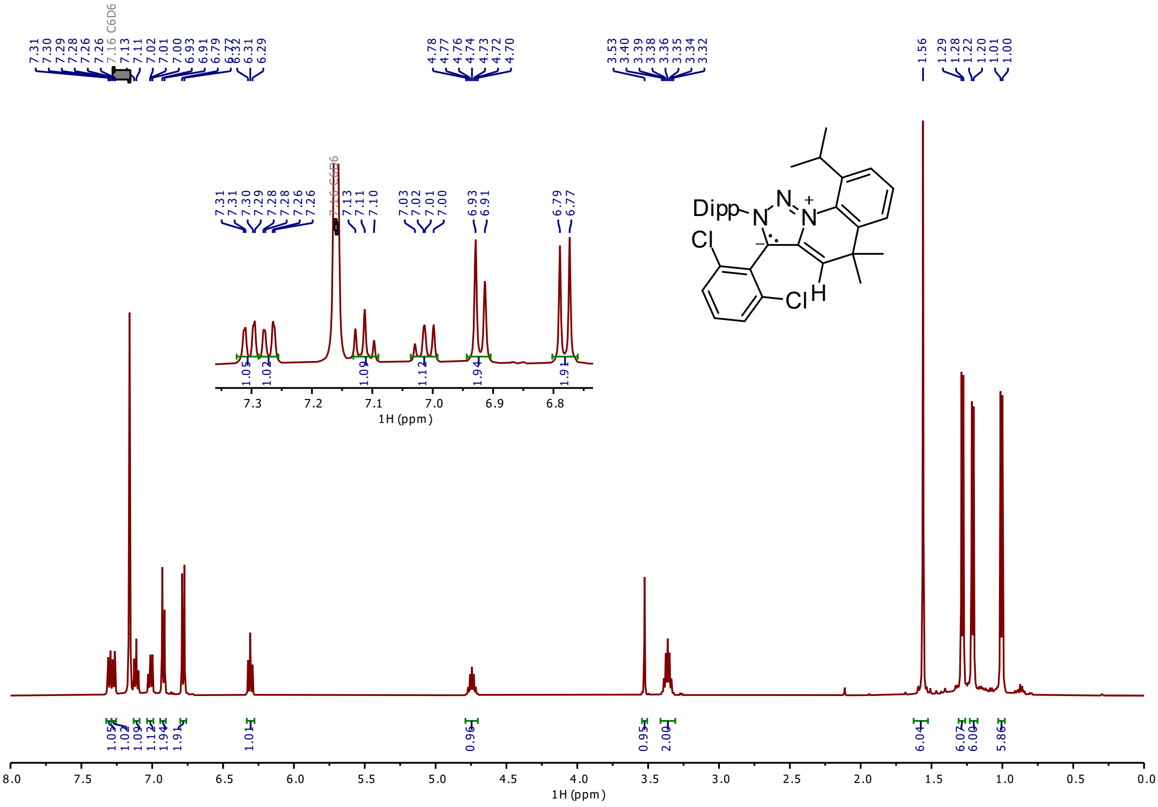

Figure S1.81. ^{1}**H NMR** (501 MHz, C_6D_6, 298 K) of **4A^{Cl}**.

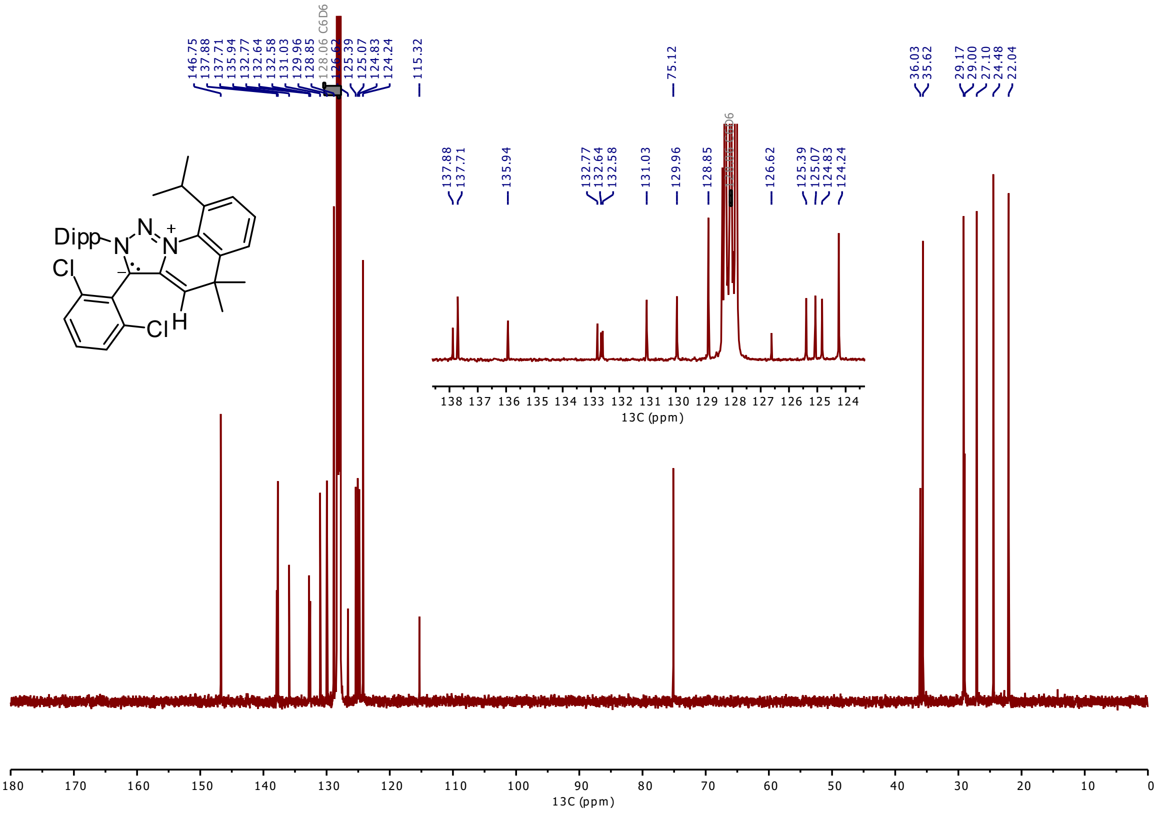

Figure S1.82. ^{13}**C NMR** (126 MHz, C_6D_6, 298 K) of **4A^{Cl}**.

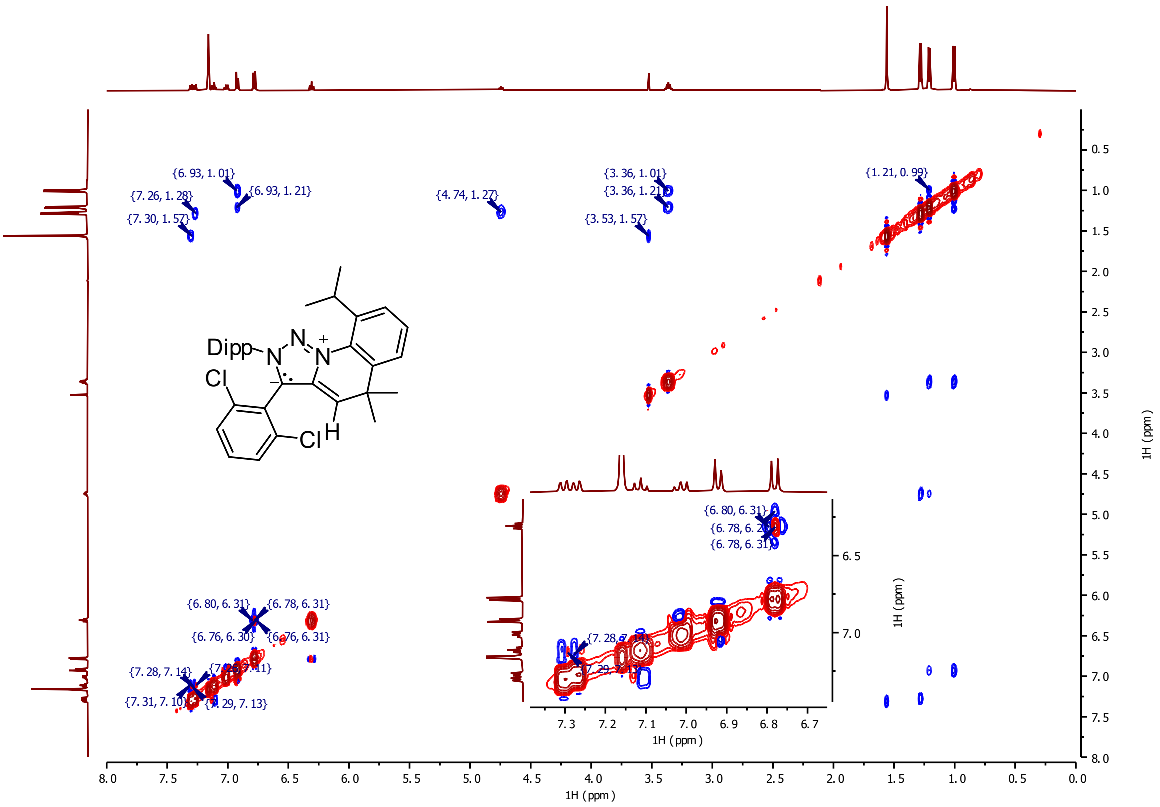

Figure S1.83. ^{1}H/^{1}H **NOESY** (501/501 MHz, C_6D_6, 298 K) of **4A^{Cl}**.

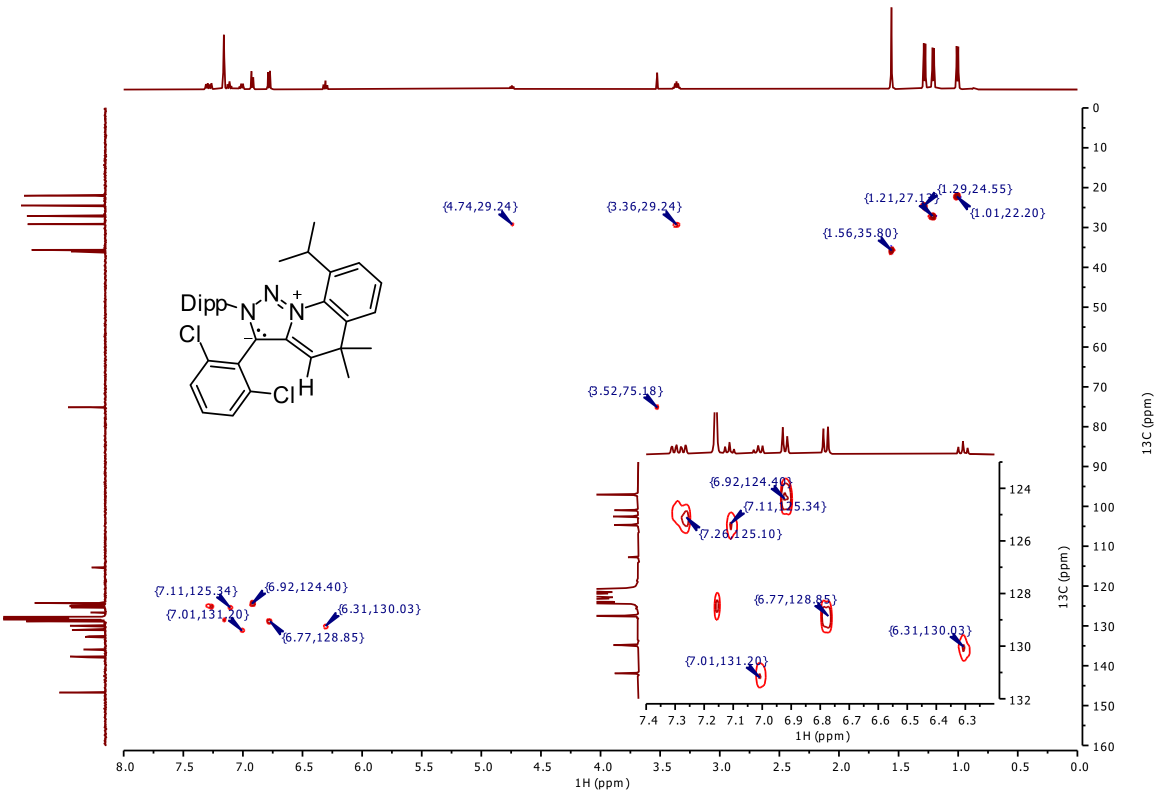

Figure S1.84. ^{1}H/^{13}C **HSQC** (501/126 MHz, C_6D_6, 298 K) of **4A^{Cl}**.

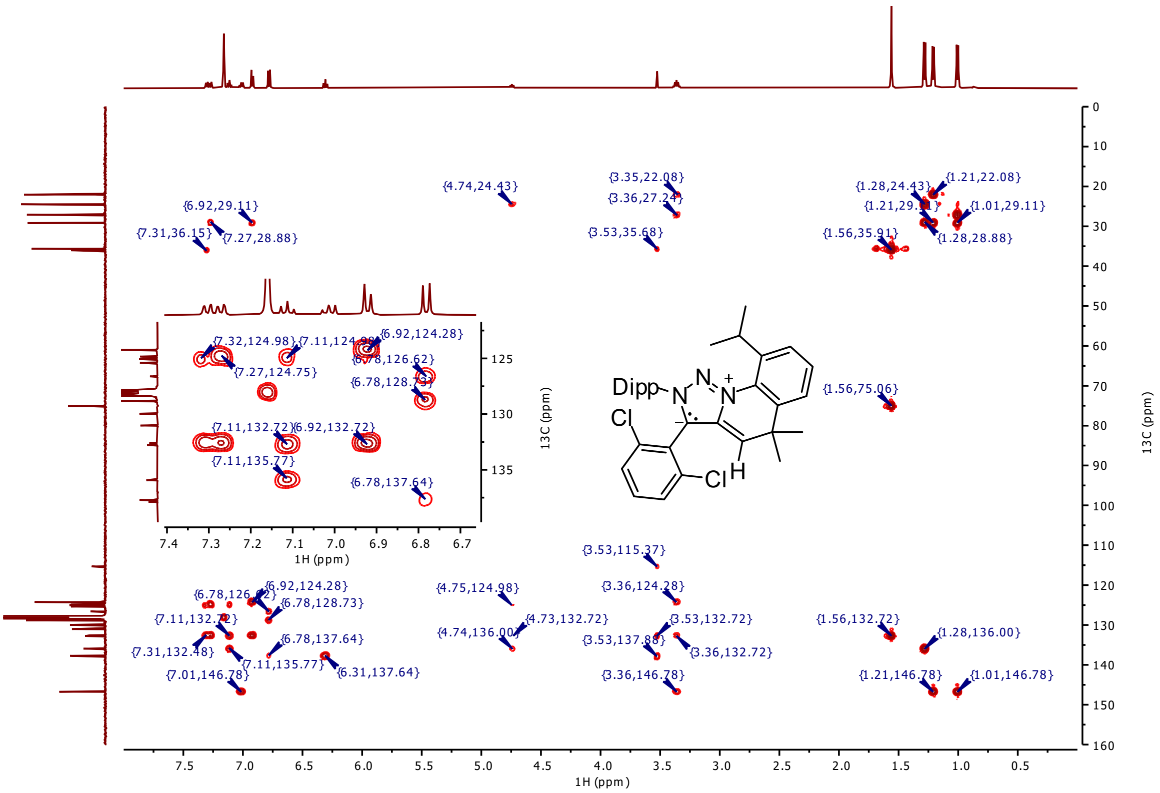

Figure S1.85. **$^1H/^{13}C$ HMBC** (501/126 MHz, C_6D_6, 298 K) of **4A^{Cl}**.

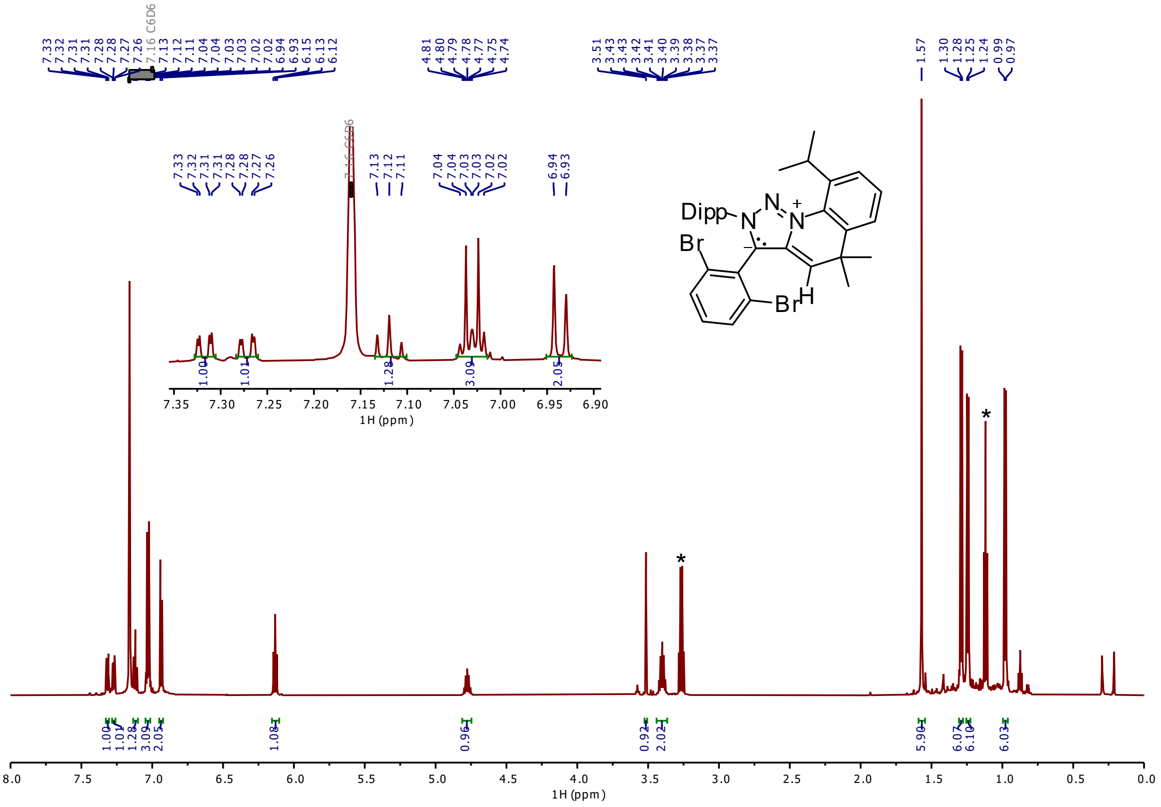

Figure S1.86. **^1H NMR** (600 MHz, C_6D_6, 298 K) of **4A^{Br}**.

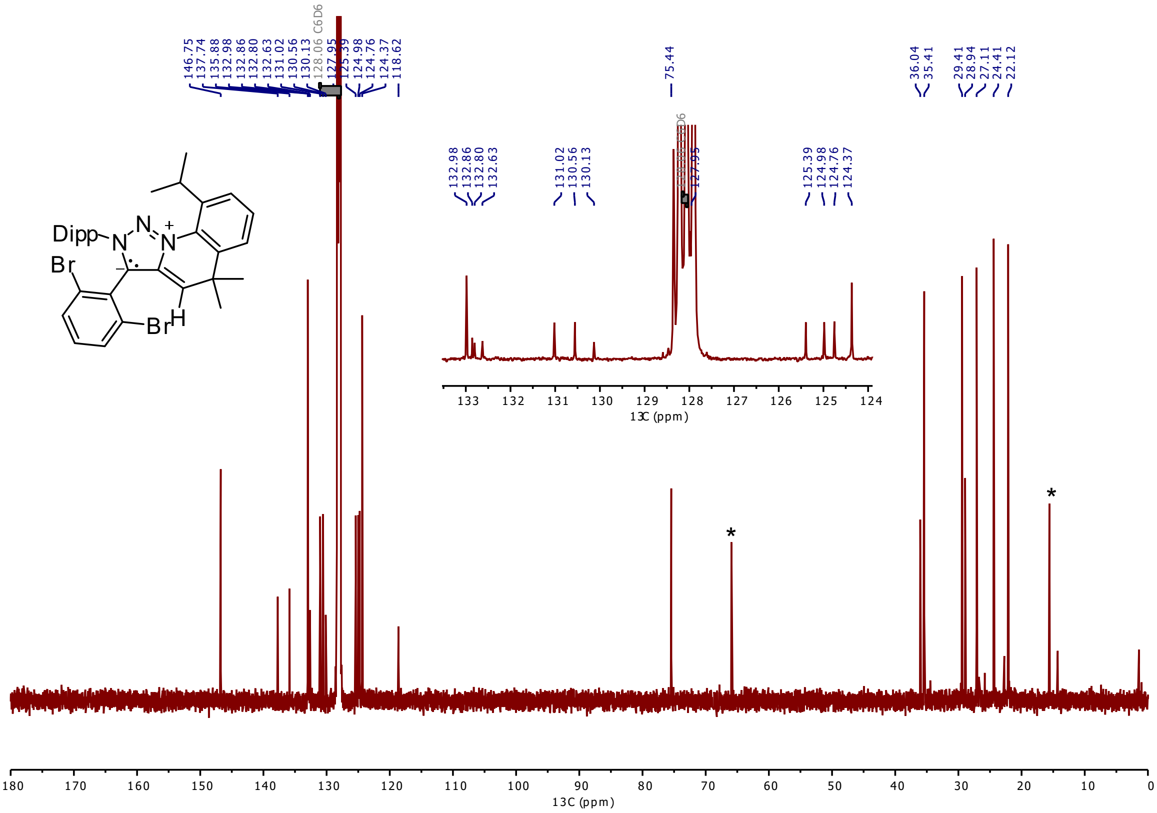

Figure S1.87. **^{13}C NMR** (151 MHz, C_6D_6, 298 K) of **4A^{Br}**.

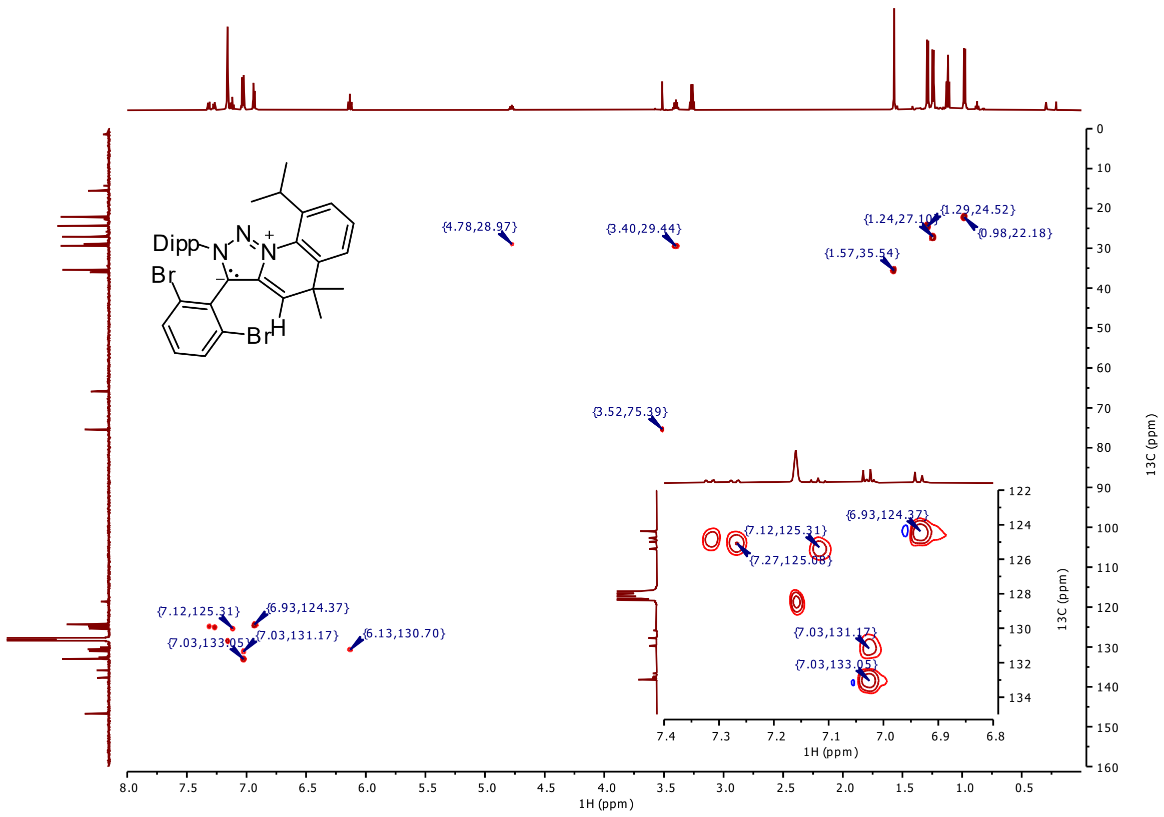

Figure S1.88. **^{1}H/^{13}C HSQC** (600/151 MHz, C_6D_6, 298 K) of **4A^{Br}**.

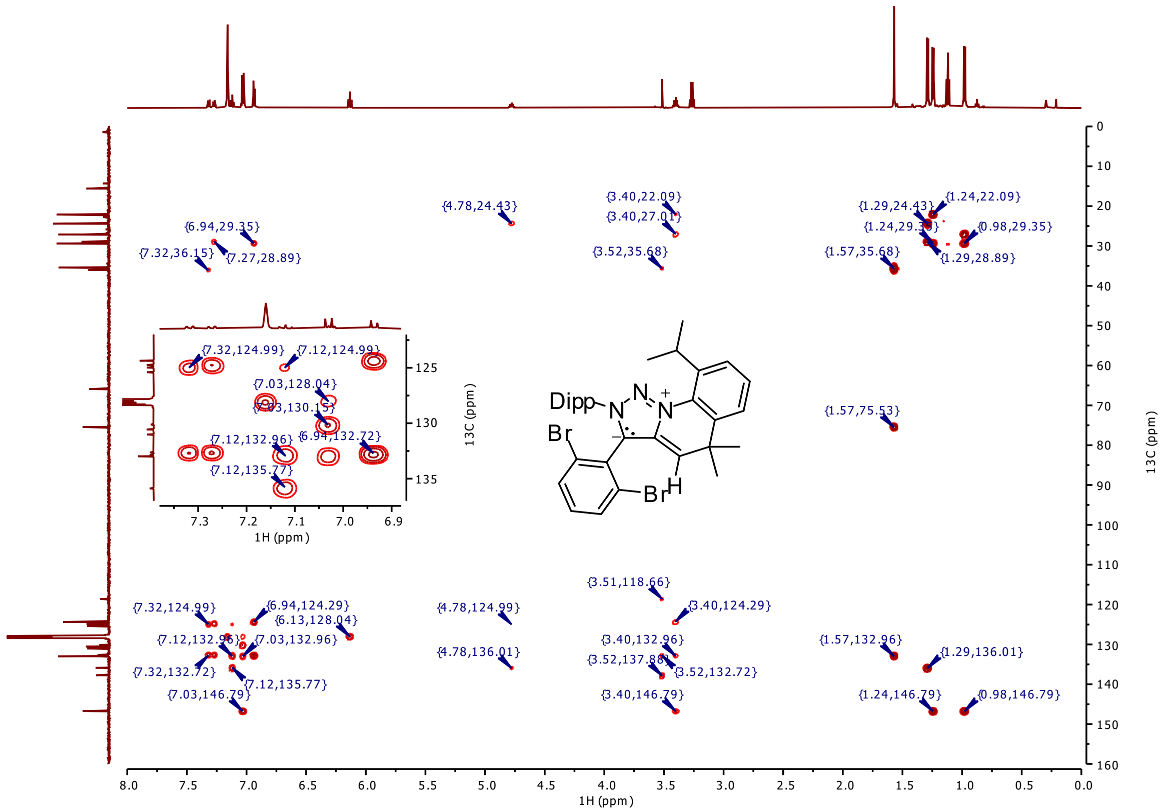

Figure S1.89. ^{1}**H/**^{13}**C HMBC** (600/151 MHz, C_6D_6, 298 K) of **4A**^{Br}.

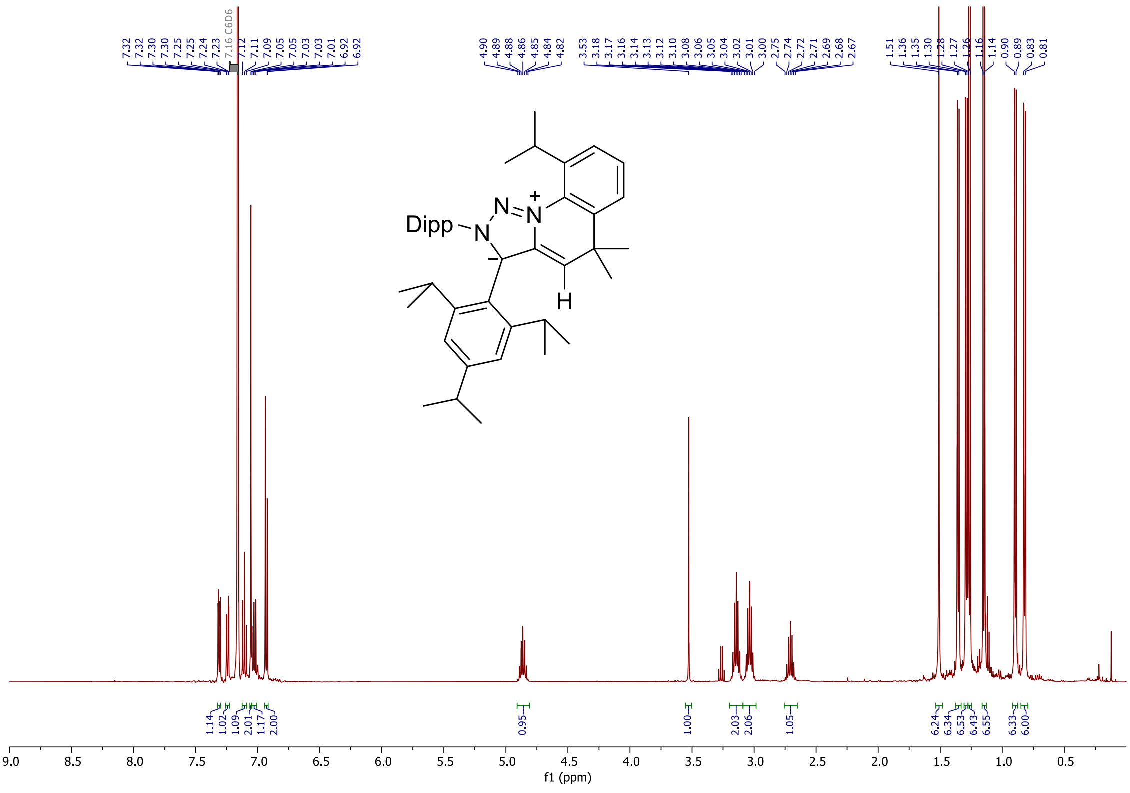

Figure S1.90. ^{1}**H NMR** (500 MHz, C_6D_6, 298 K) of **4A**^{iPr}.

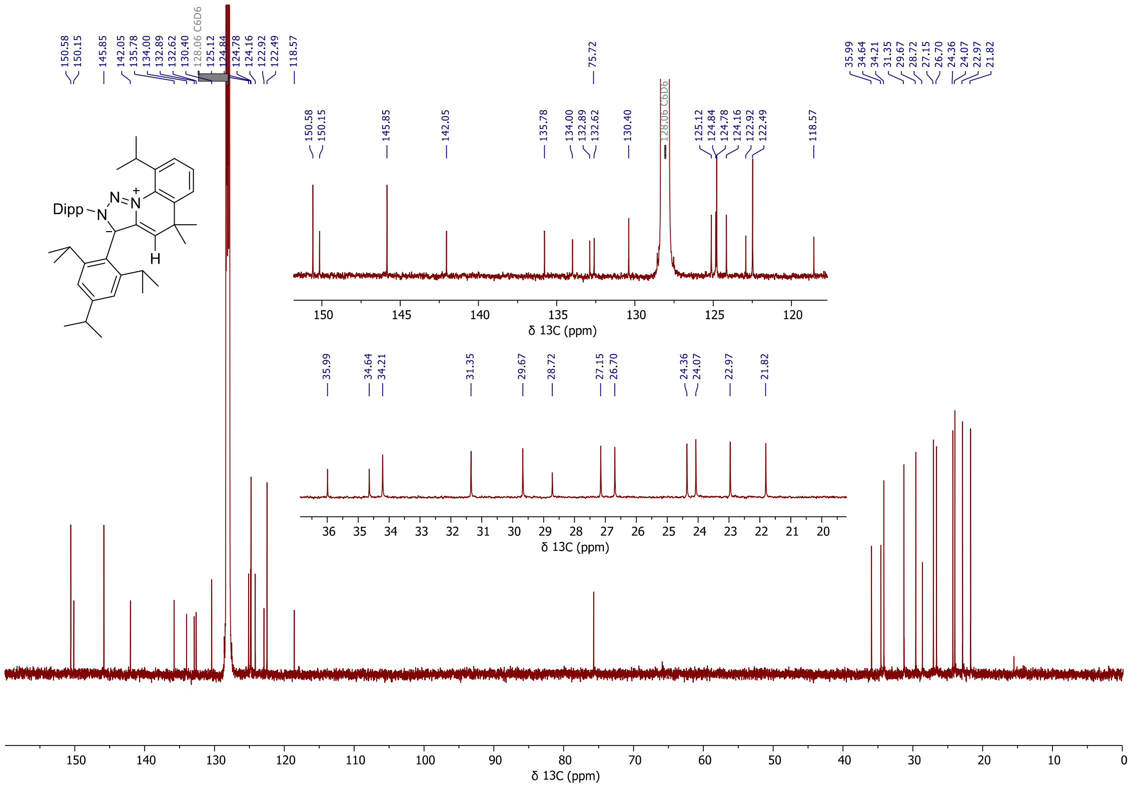

Figure S1.91. **^{13}C NMR** (126 MHz, C_6D_6, 298 K) of **4A^{iPr}**.

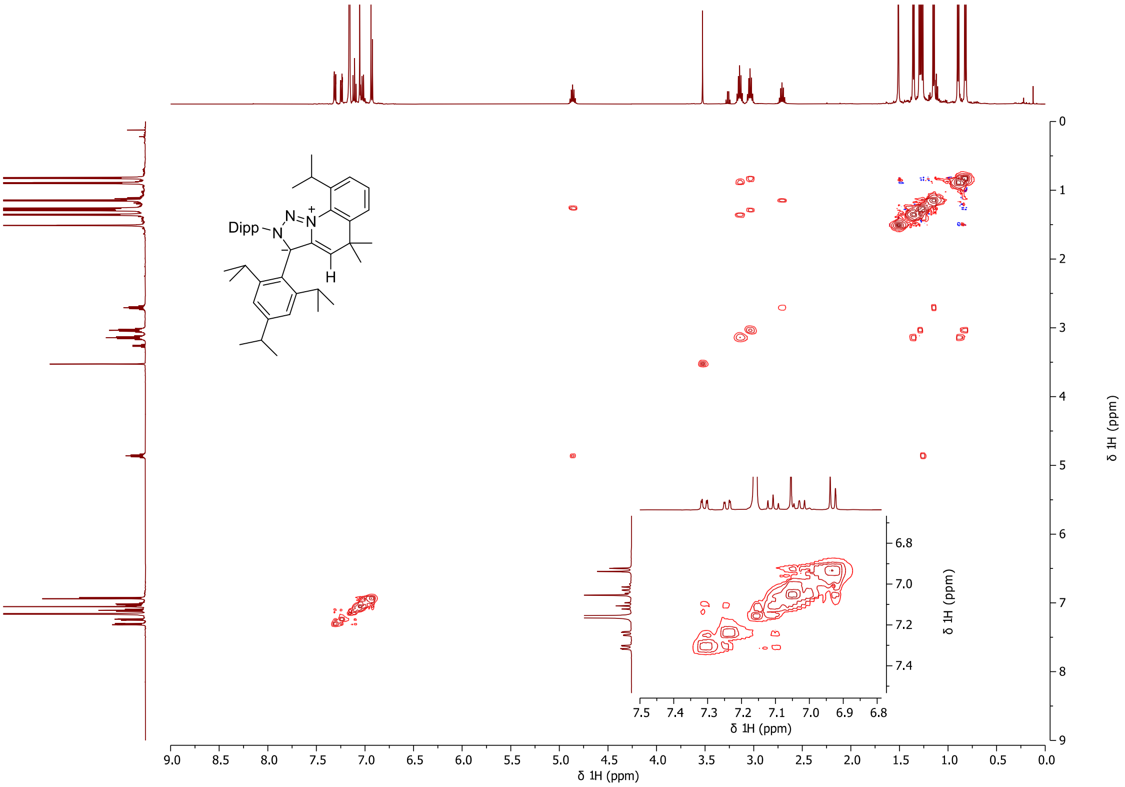

Figure S1.92. **^{1}H/^{1}H COSY** (500/500 MHz, C_6D_6, 298 K) of **4A^{iPr}**.

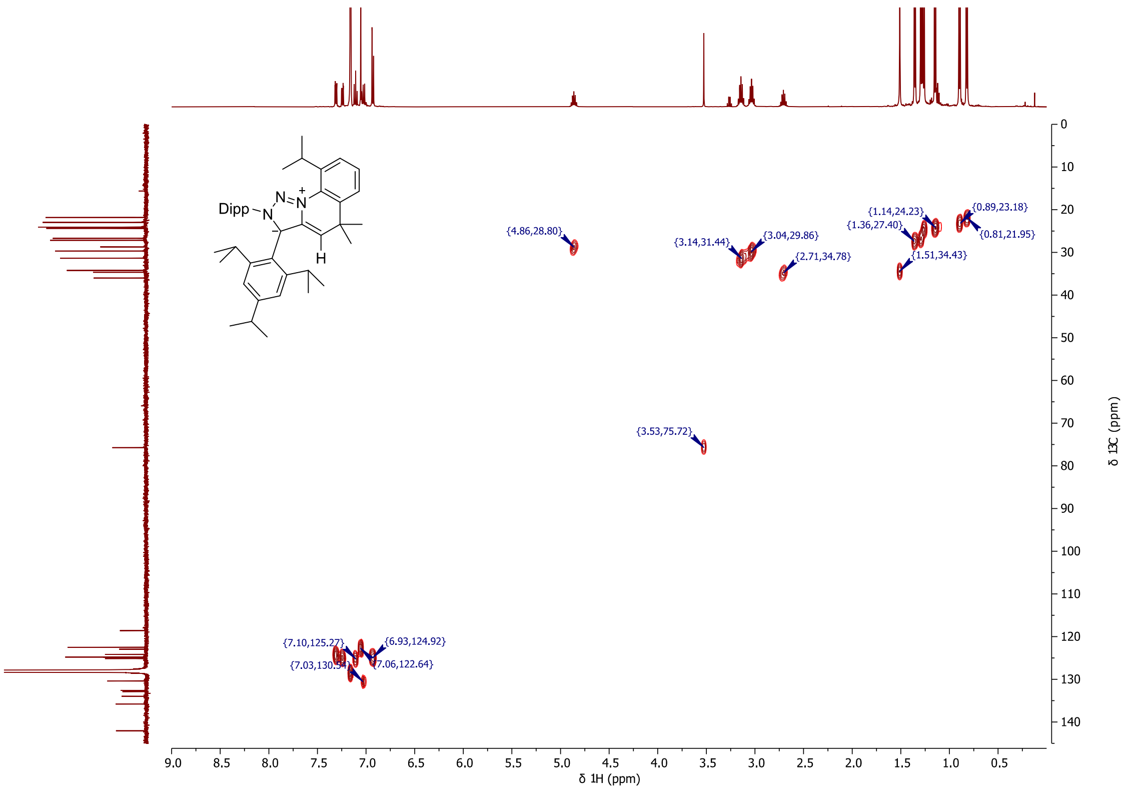

Figure S1.93. **^{1}H/^{13}C HSQC** (500/126 MHz, C_6D_6, 298 K) of **4A^{iPr}**.

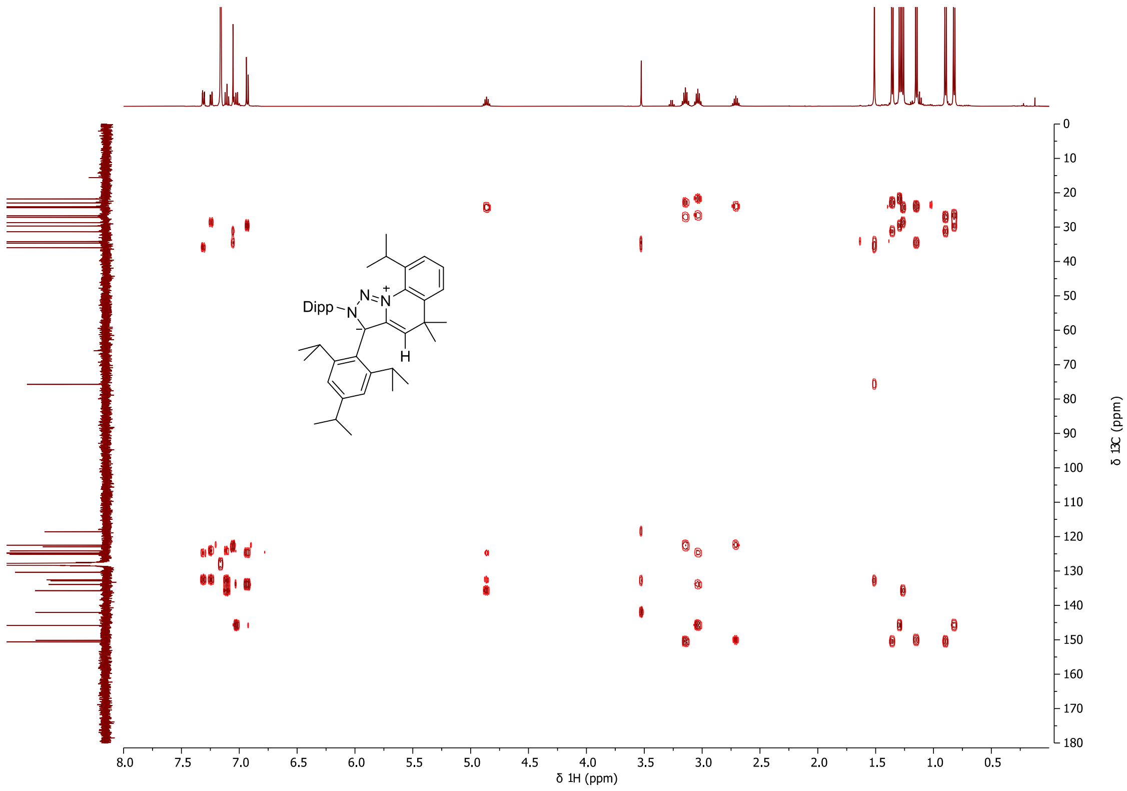

Figure S1.94. **^{1}H/^{13}C HMBC** (500/126 MHz, C_6D_6, 298 K) of **4A^{iPr}**.

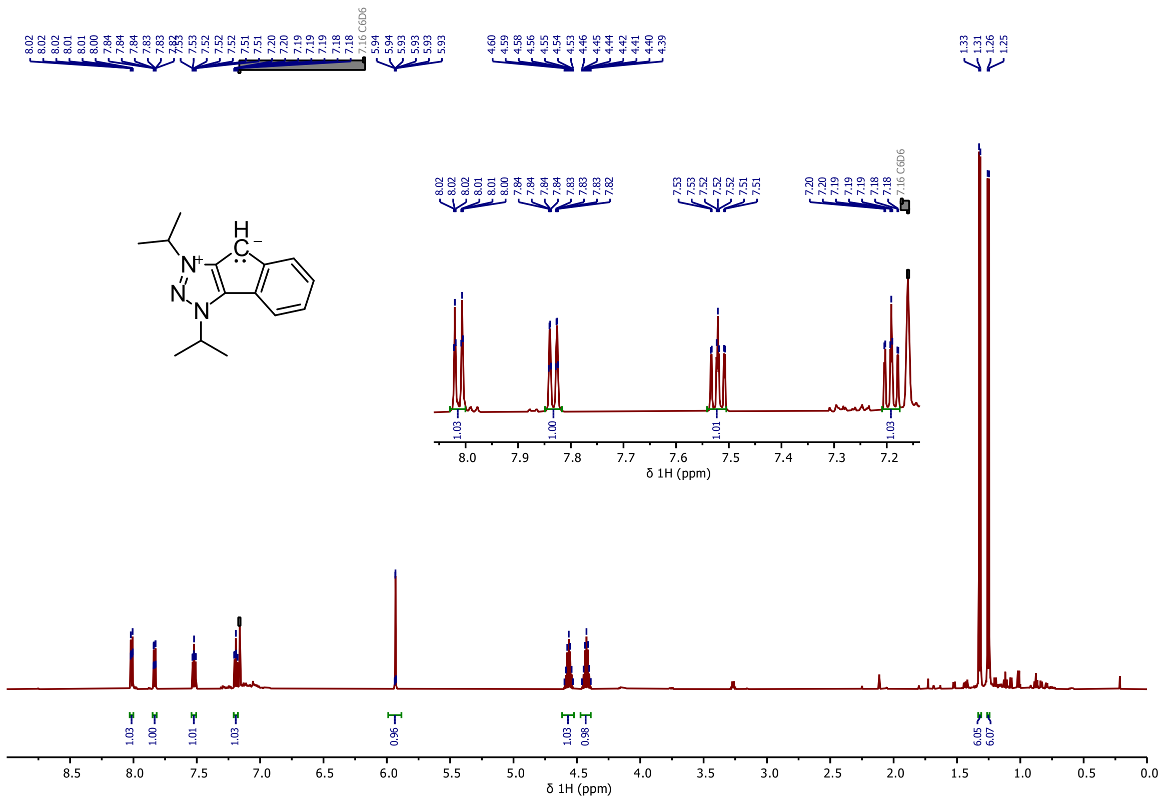

Figure S1.95. ^{1}**H NMR** (600 MHz, C_6D_6, 298 K) of **3B**.

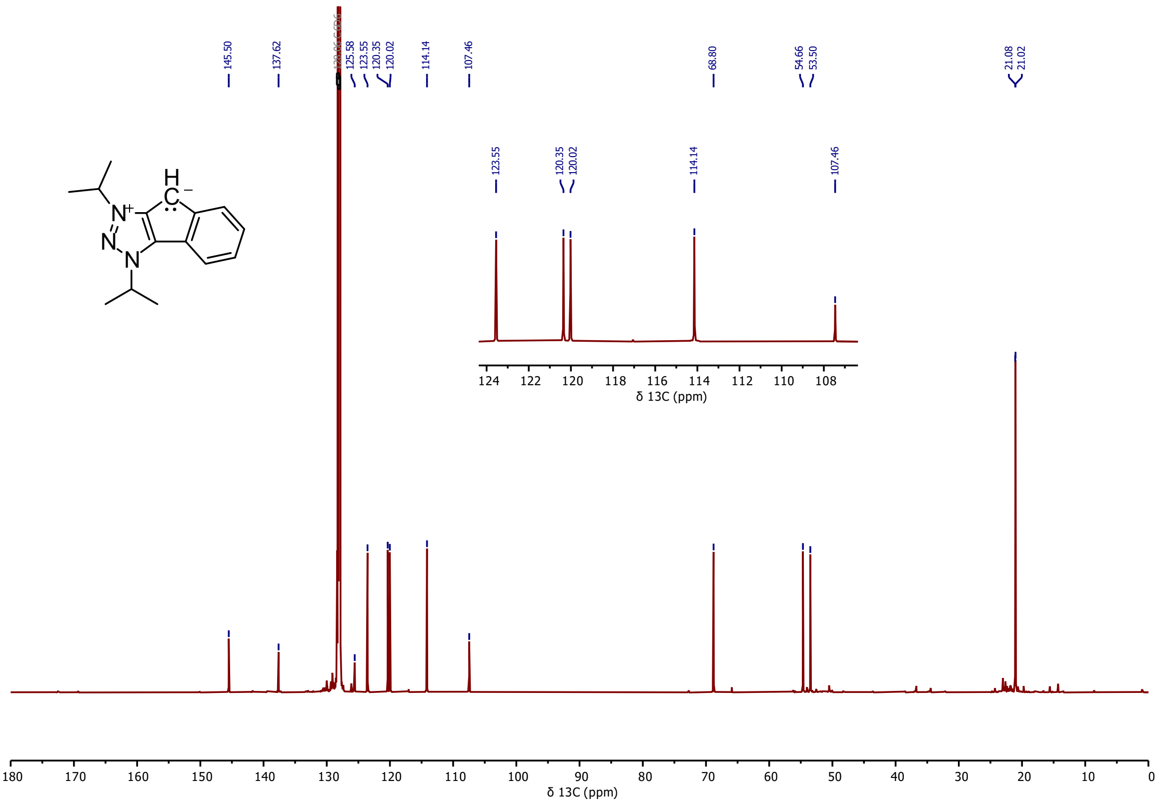

Figure S1.96. ^{13}**C NMR** (151 MHz, C_6D_6, 298 K) of **3B**.

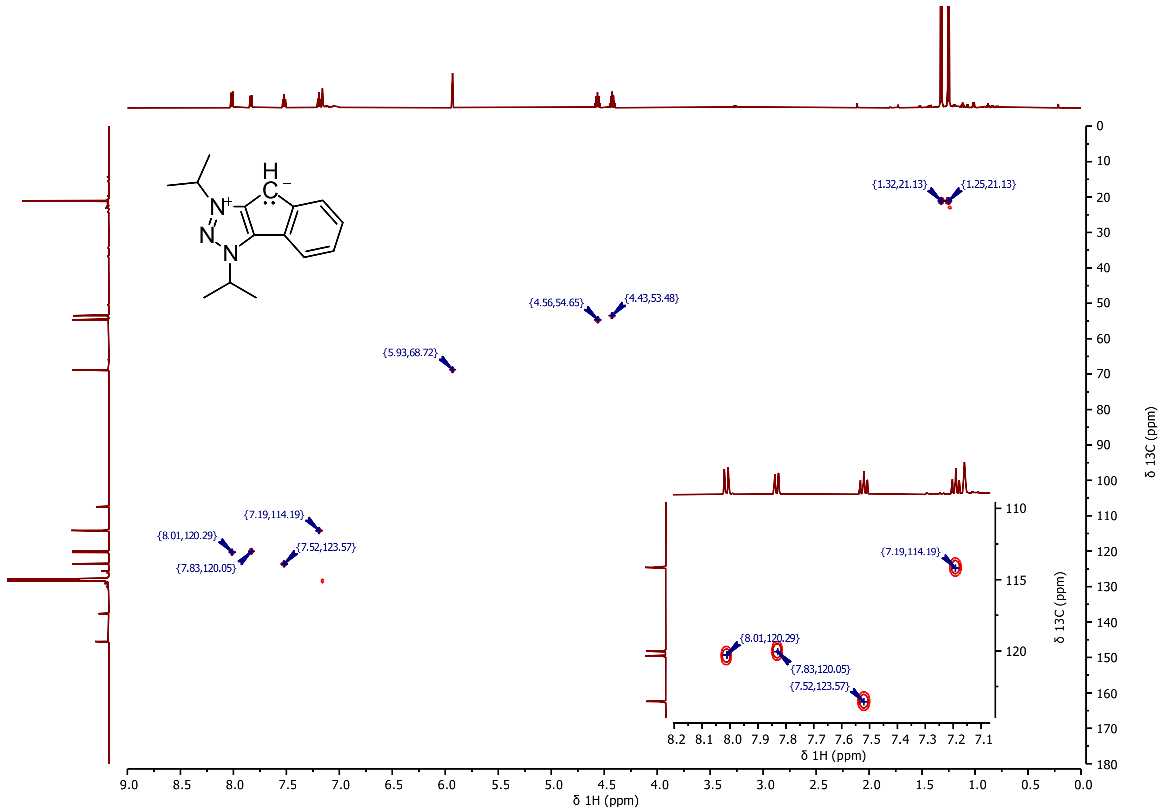

Figure S1.97. **^{1}H/^{13}C HSQC** (600/151 MHz, C_6D_6, 298 K) of **3B**.

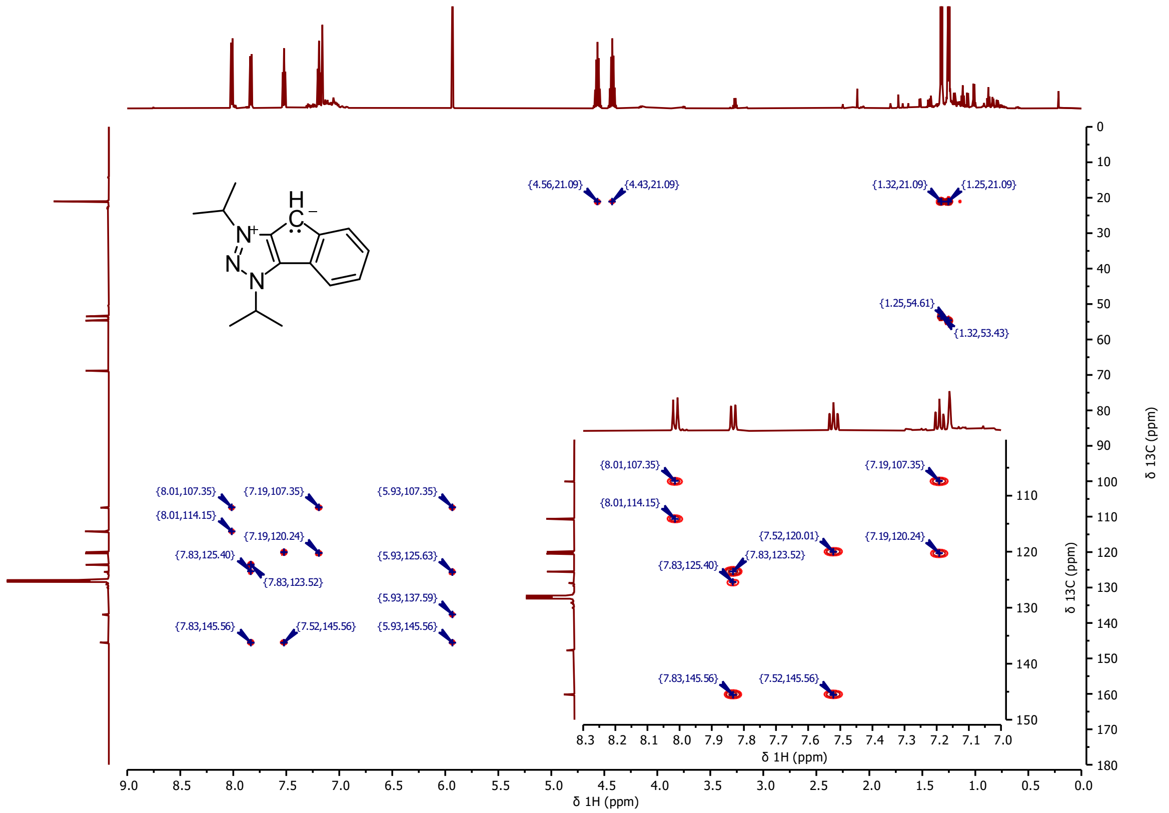

Figure S1.98. **^{1}H/^{13}C HMBC** (600/151 MHz, C_6D_6, 298 K) of **3B**.

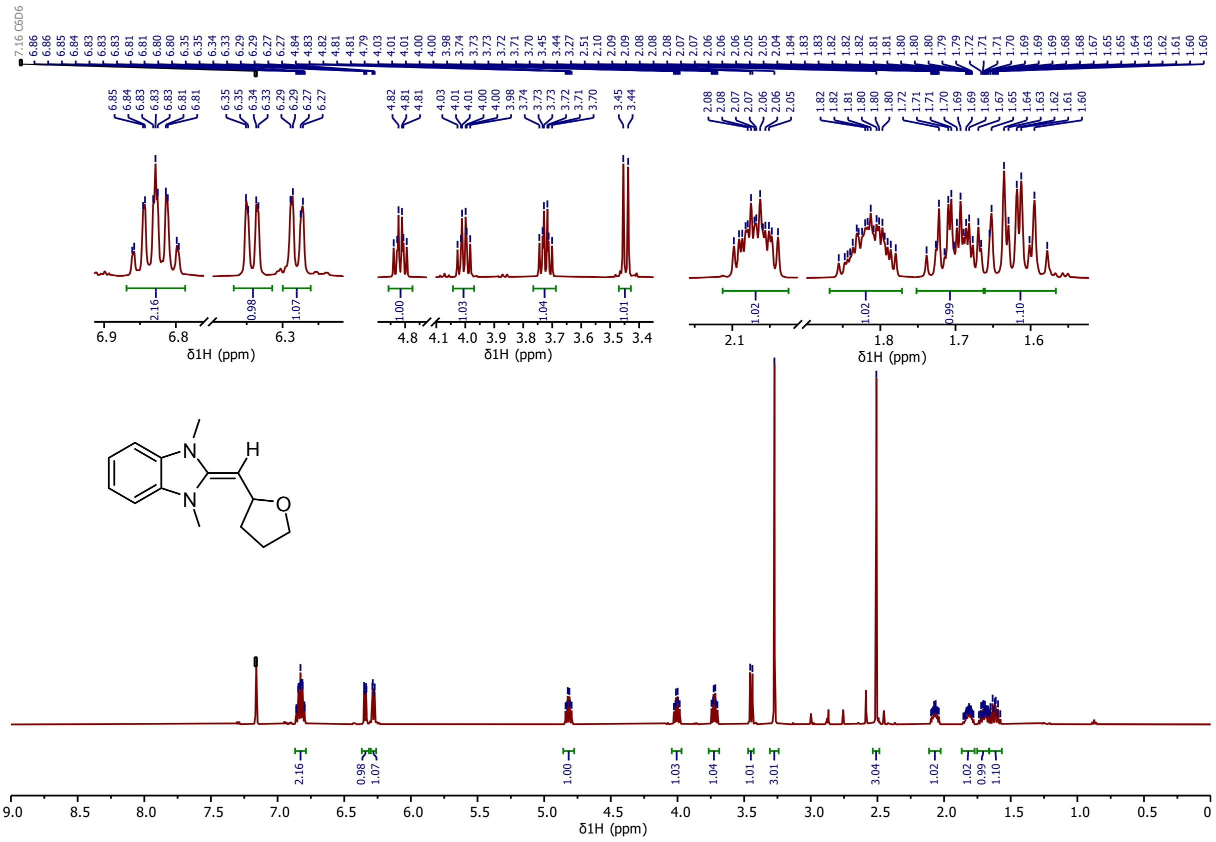

Figure S1.99: **¹H NMR** (500 MHz, C_6D_6, 298 K) of **5**.

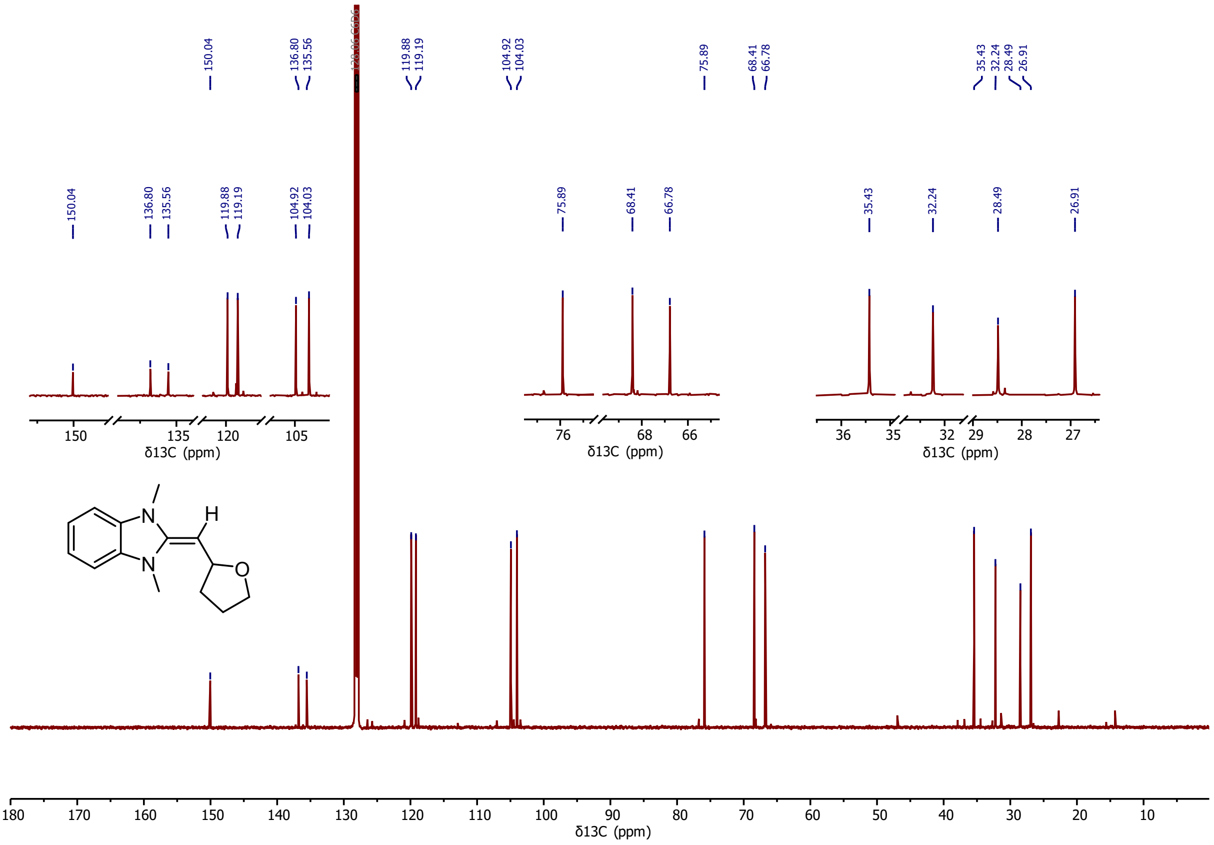

Figure S1.100: **¹³C NMR** (126 MHz, C_6D_6, 298 K) of **5**.

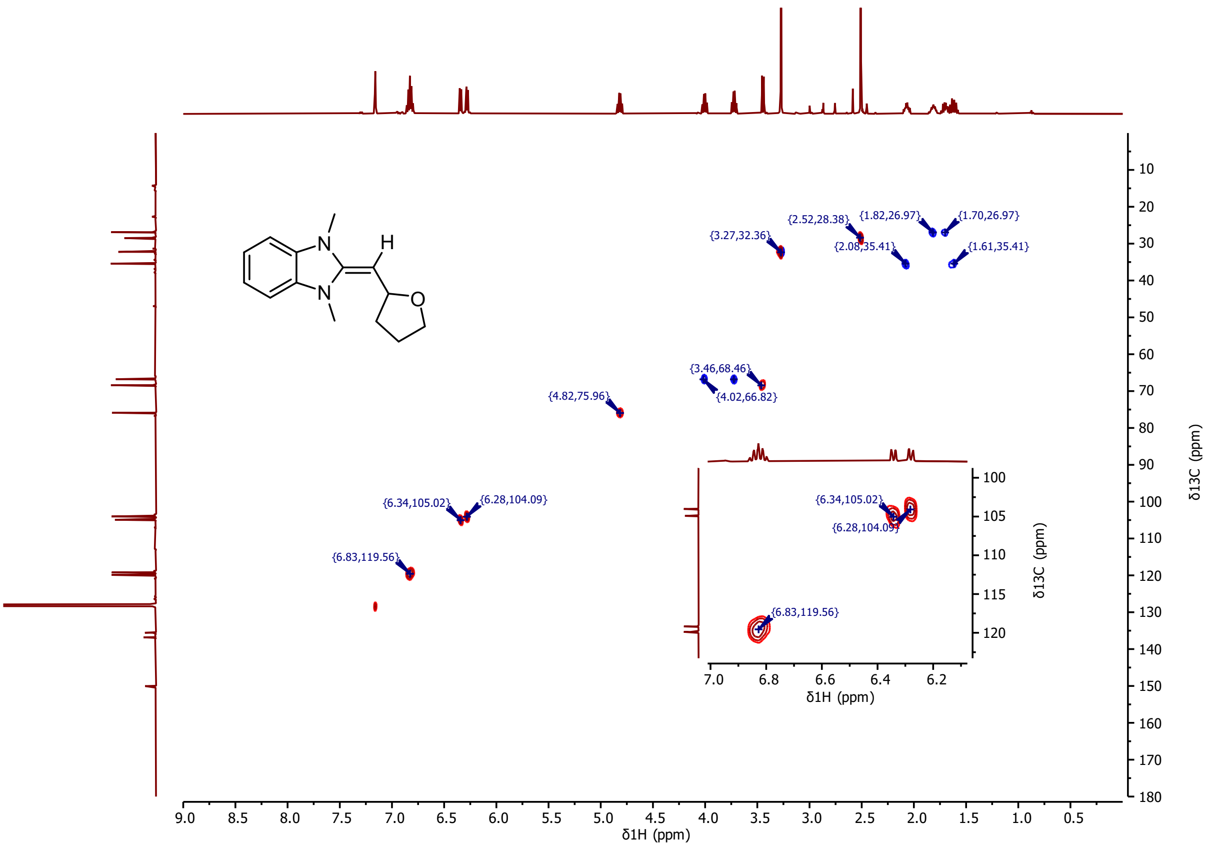

Figure S1.101: **^{1}H/^{13}C HSQC** (500/126 MHz, C_6D_6, 298 K) of **5**.

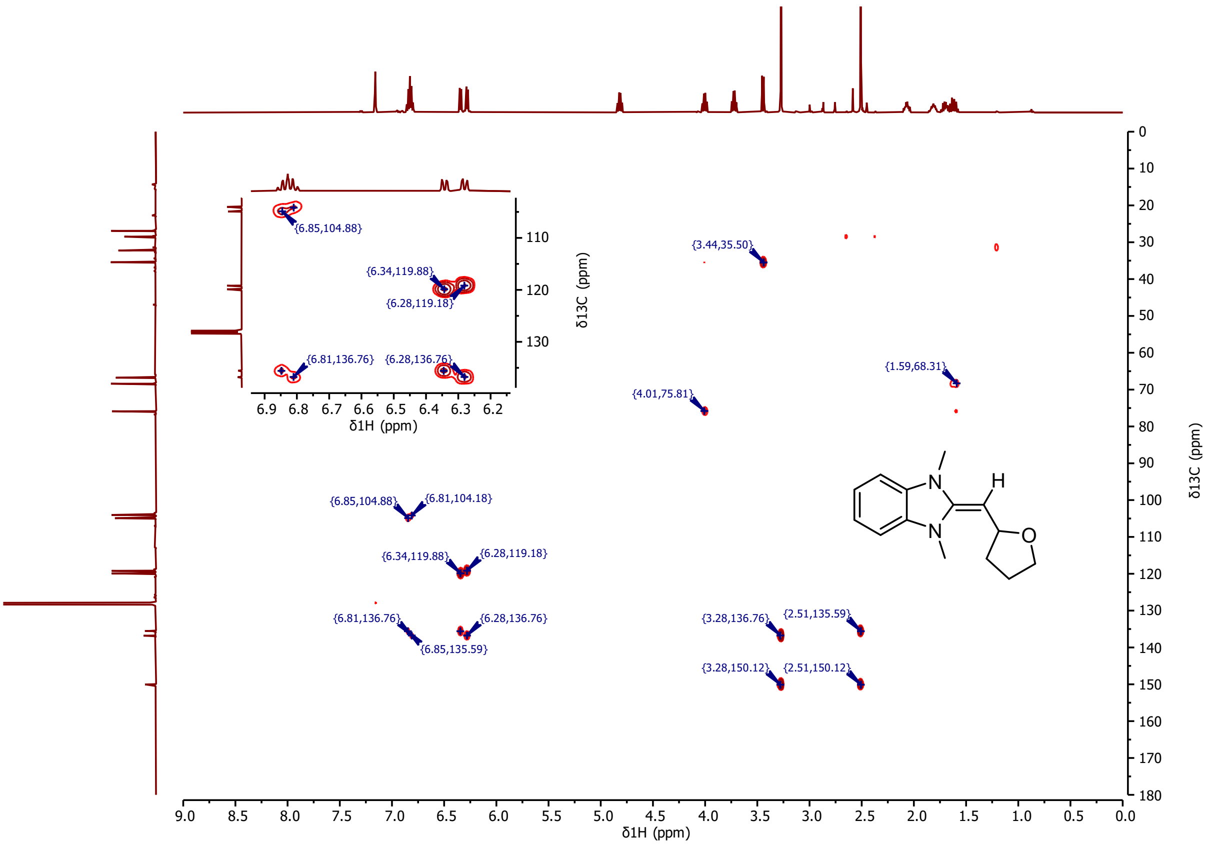

Figure S1.102: **^{1}H/^{13}C HMBC** (500/126 MHz, C_6D_6, 298 K) of **5**.

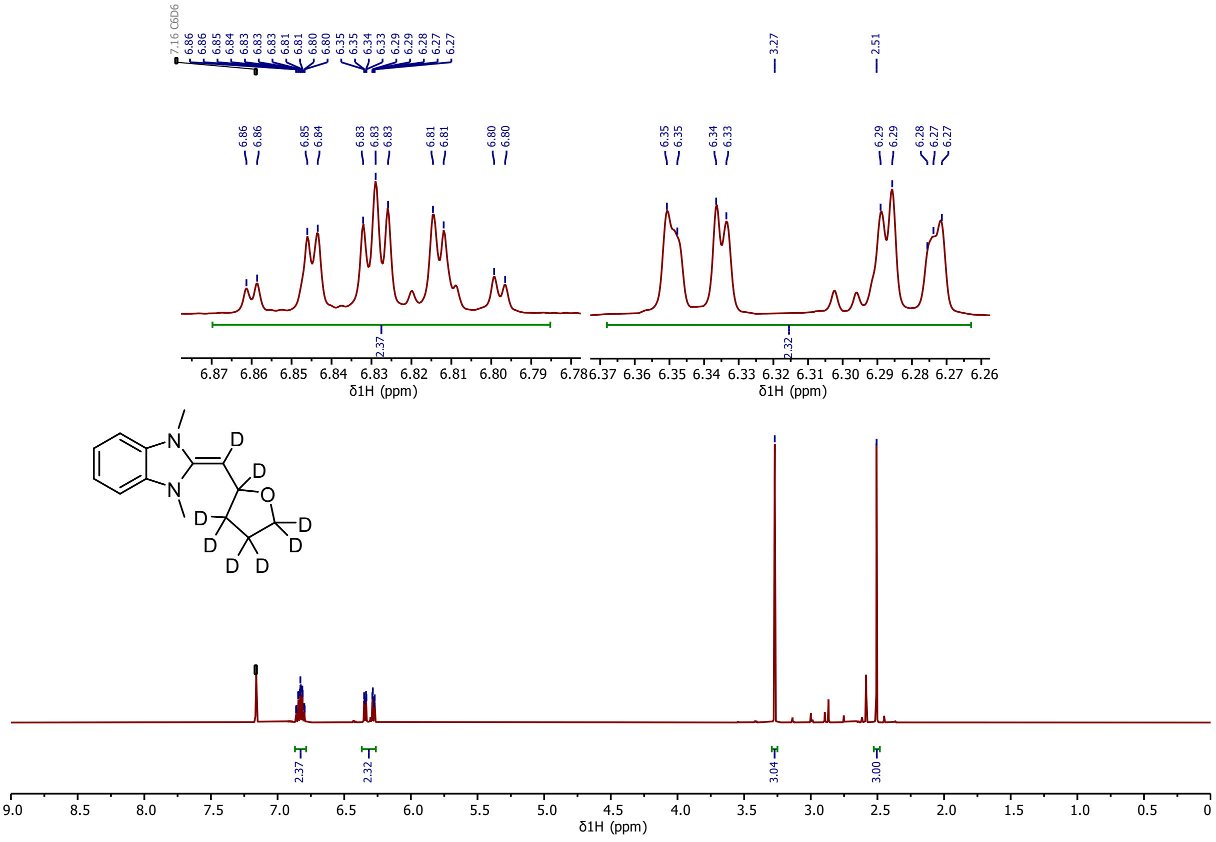

Figure S1.103: **^{1}H NMR** (500 MHz, C_6D_6, 298 K) of **5-d$_8$**.

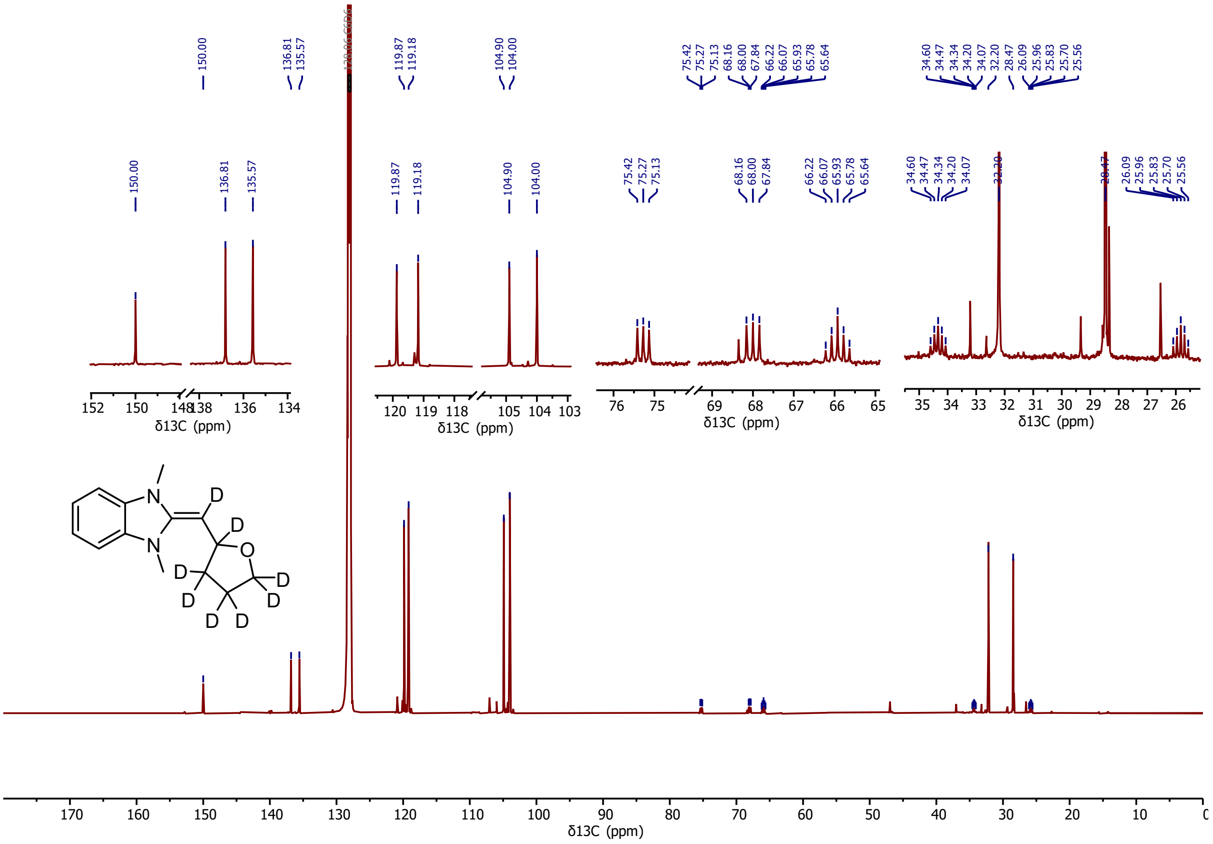

Figure S1.104: **^{13}C NMR** (151 MHz, C_6D_6, 298 K) of **5-d$_8$**.

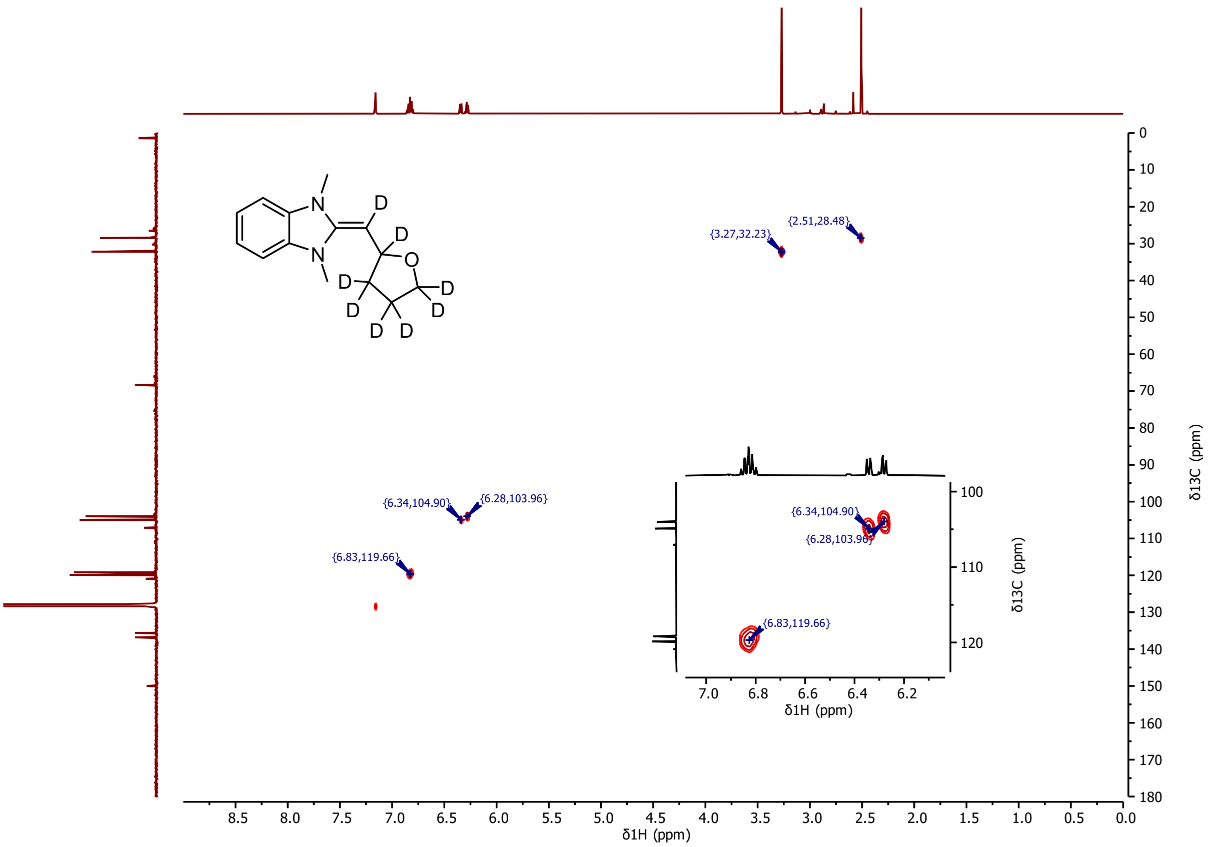

Figure S1.105: **^{1}H/^{13}C HSQC** (500/126 MHz, C_6D_6, 298 K) of **5-d$_8$**.

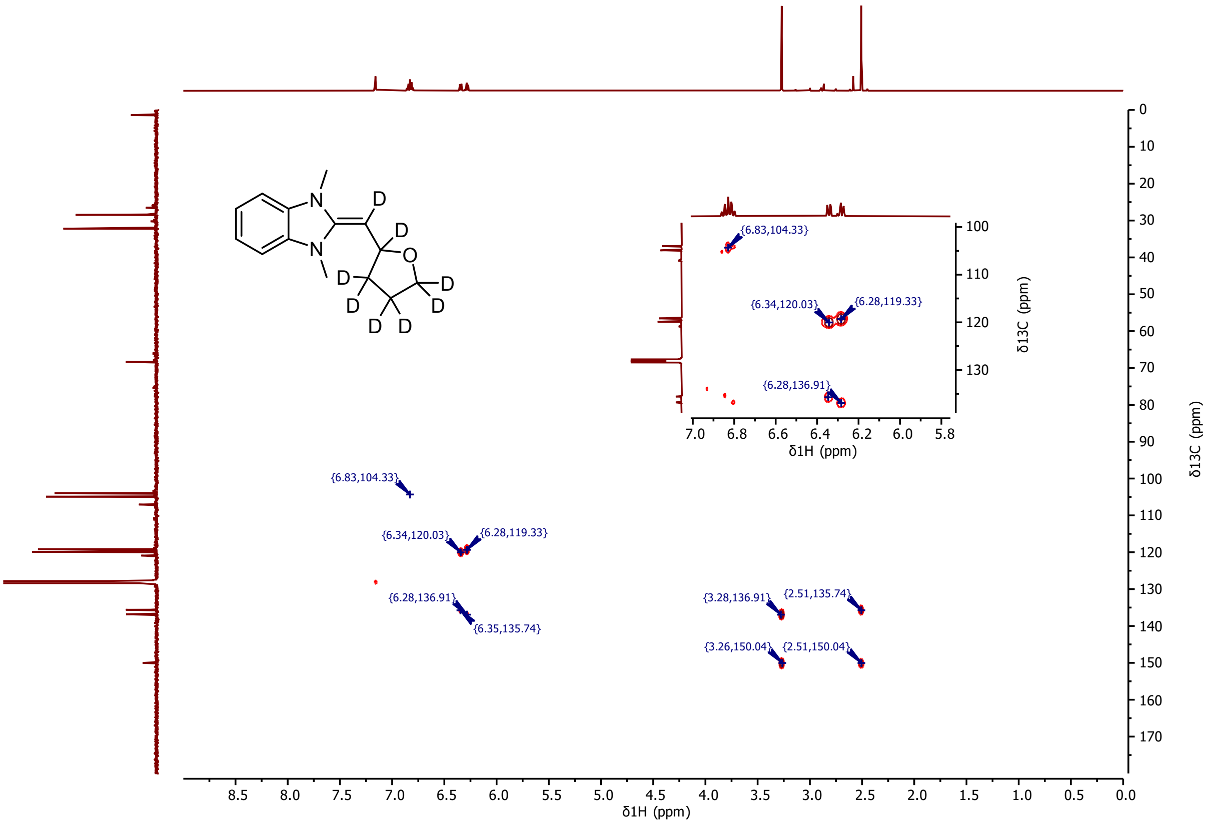

Figure S1.106: **^{1}H/^{13}C HMBC** (500/126 MHz, C_6D_6, 298 K) of **5-d$_8$**.

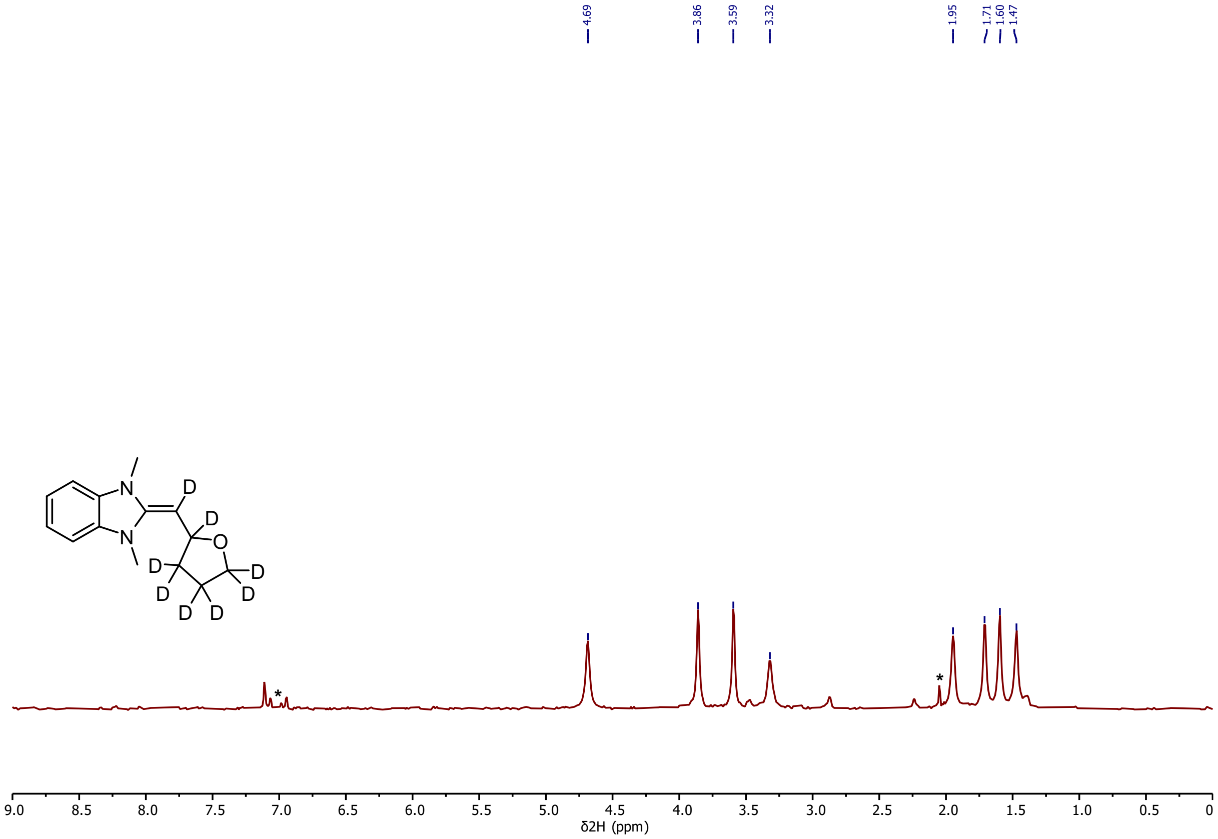

Figure S1.107: **^{2}H NMR** (500 MHz, toluene, 298 K) of **5-d$_8$**. *marks as natural abundance of deuterium in toluene.

1.6. IR-spectra

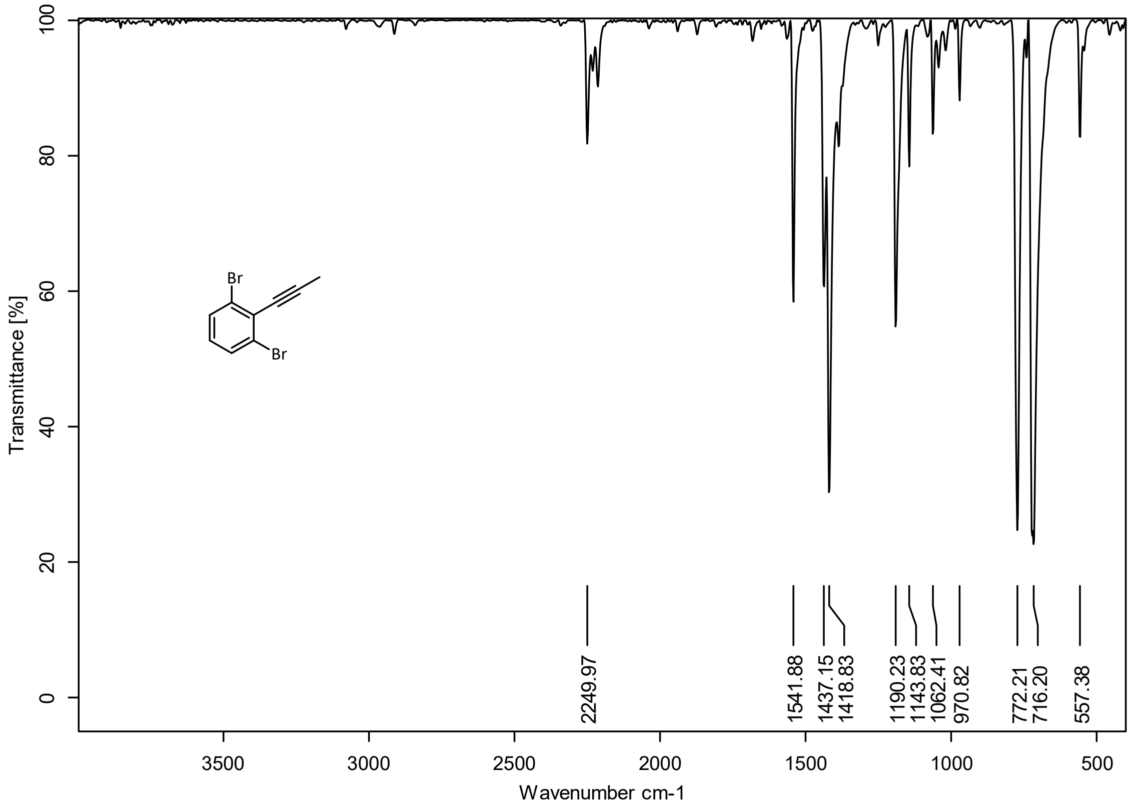

Figure S1.108. ATR IR of **1,3-dibromo-2-(prop1-yn1-yl)benzene**.

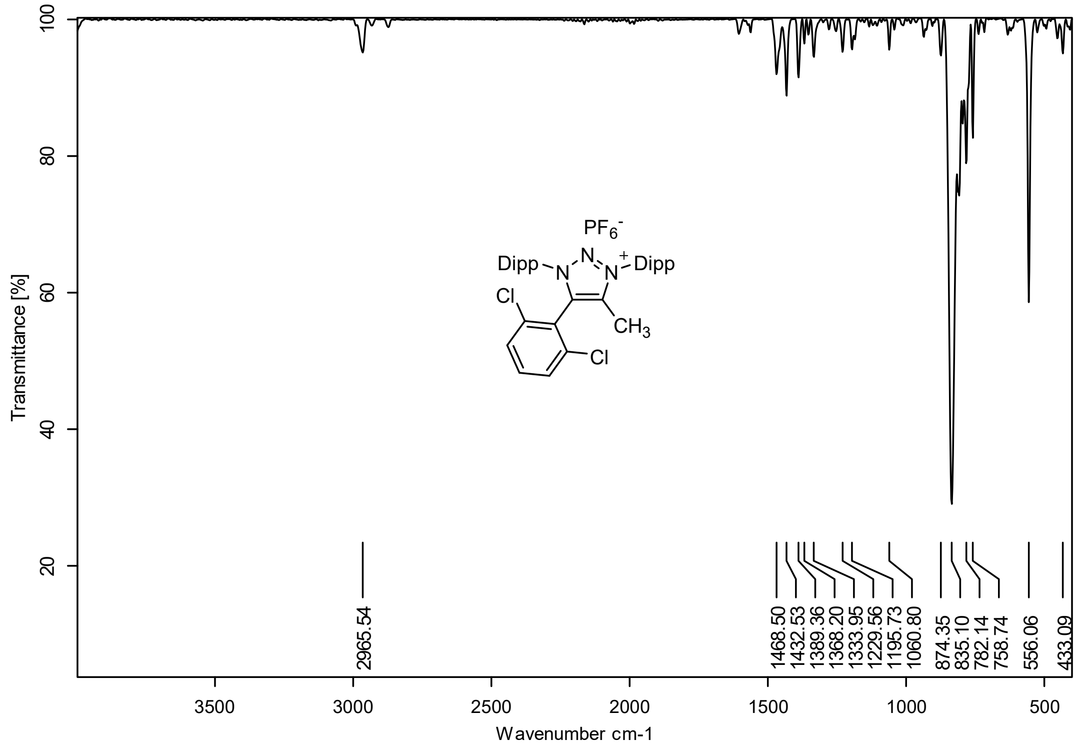

Figure S1.109. ATR IR of **dichloro-triazolium salt**.

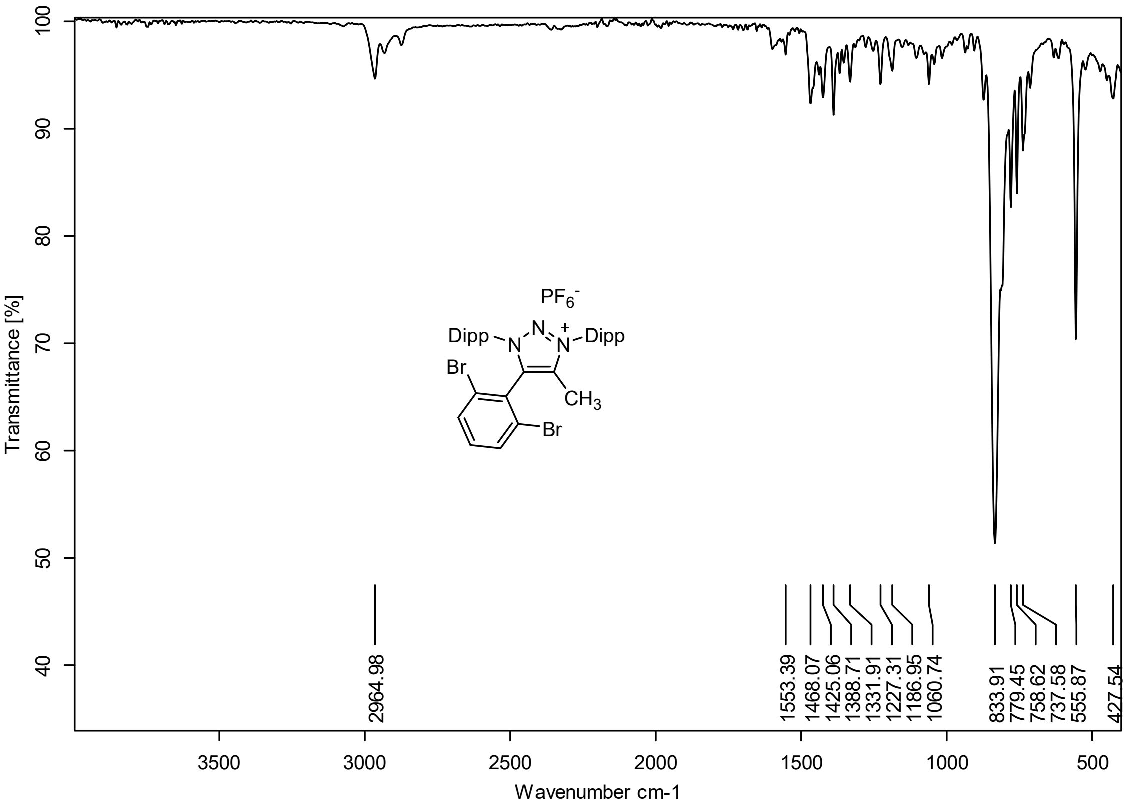

Figure S1.110. ATR IR of **dibromo-triazolium salt**.

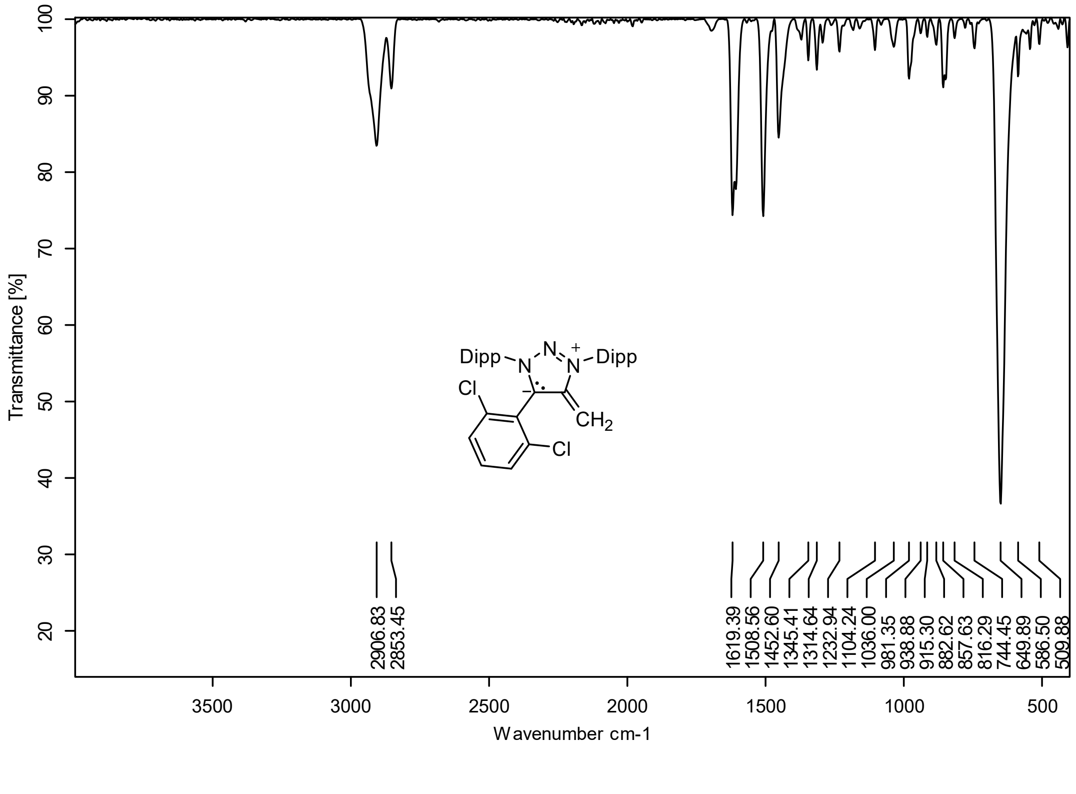

Figure S1.111. ATR IR of **dichloro-mNHO**.

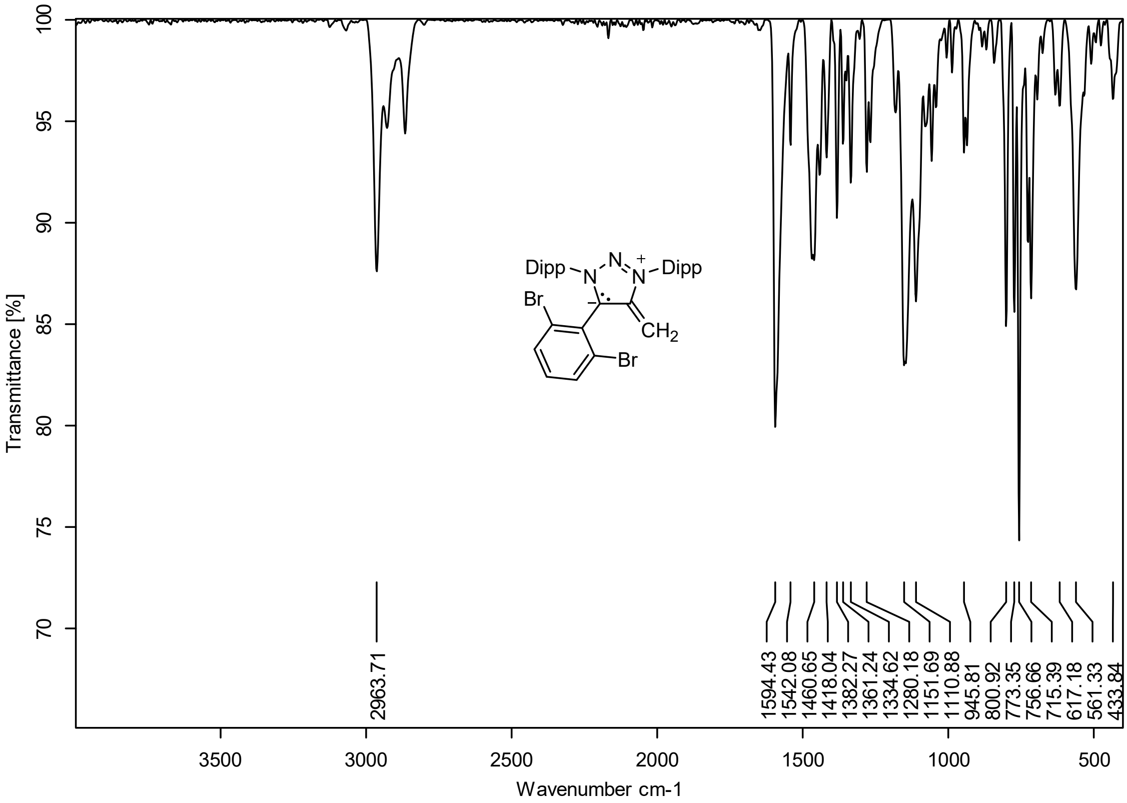

Figure S1.112. ATR IR of **dibromo-mNHO**.

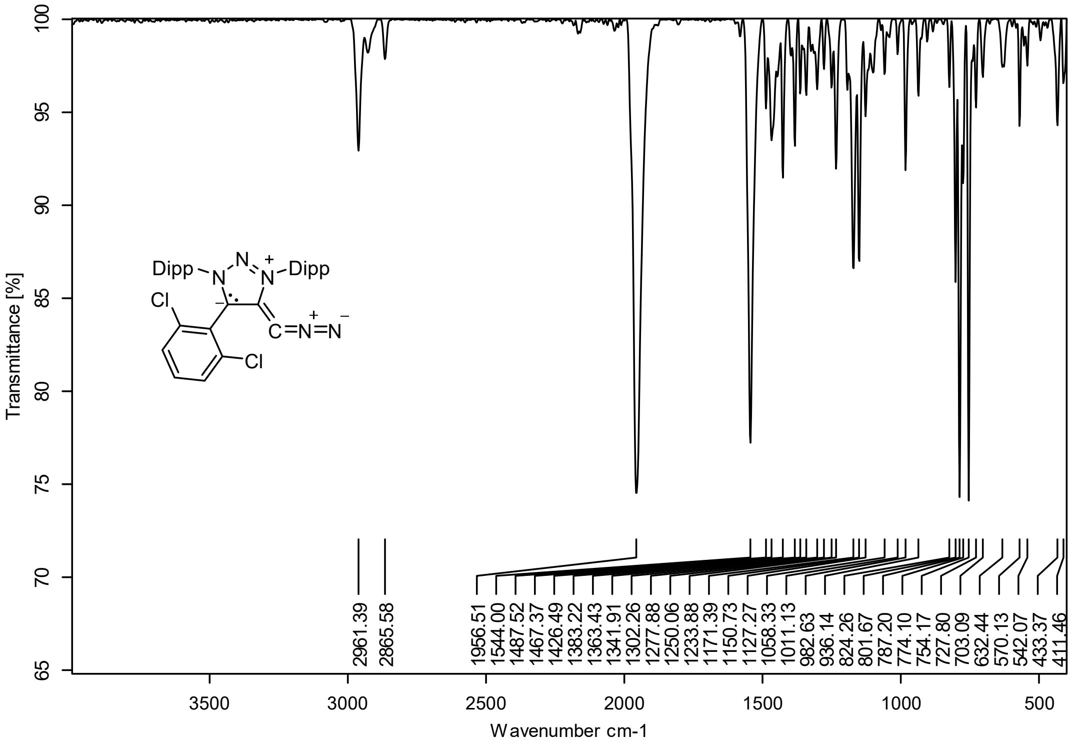

Figure S1.113. ATR IR of **1A**^{Cl}.

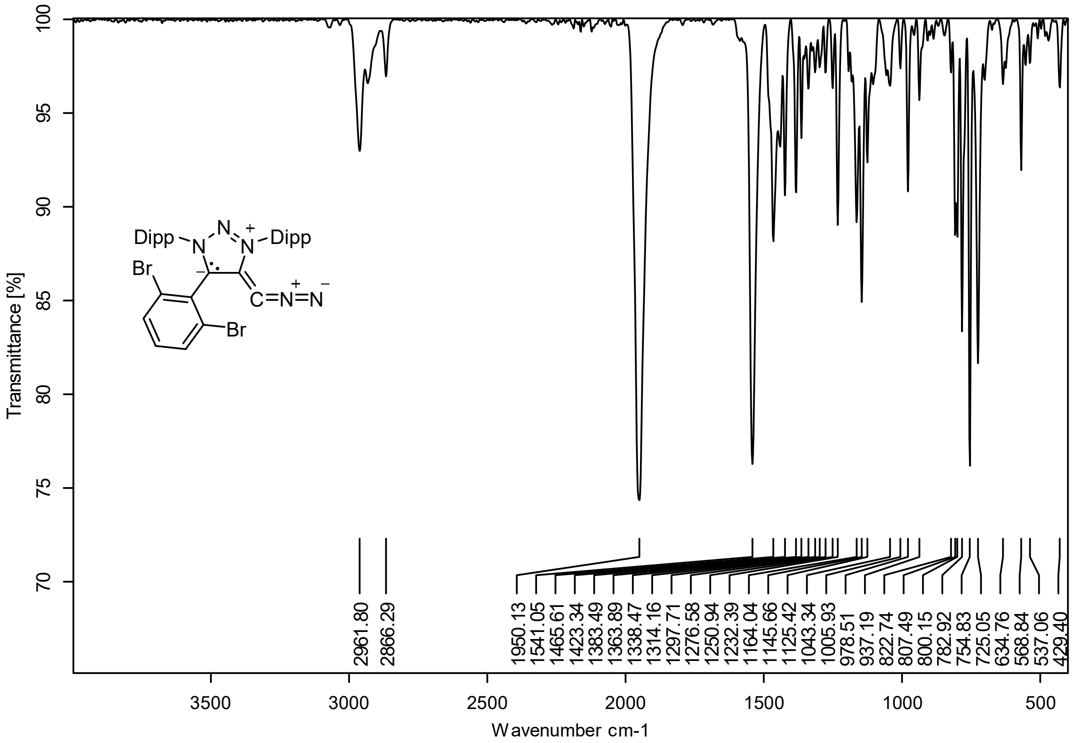

Figure S1.114. ATR IR of **1A**^{Br}.

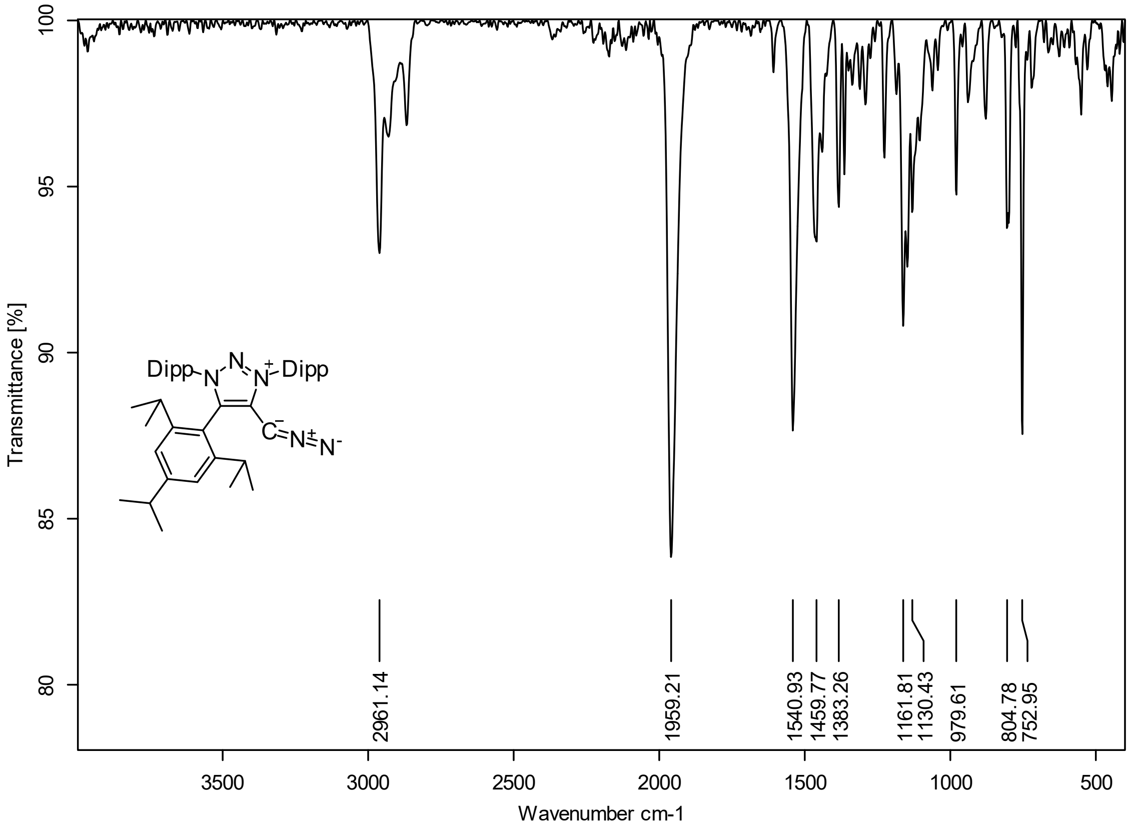

Figure S1.115. ATR IR of **1A**^{iPr}.

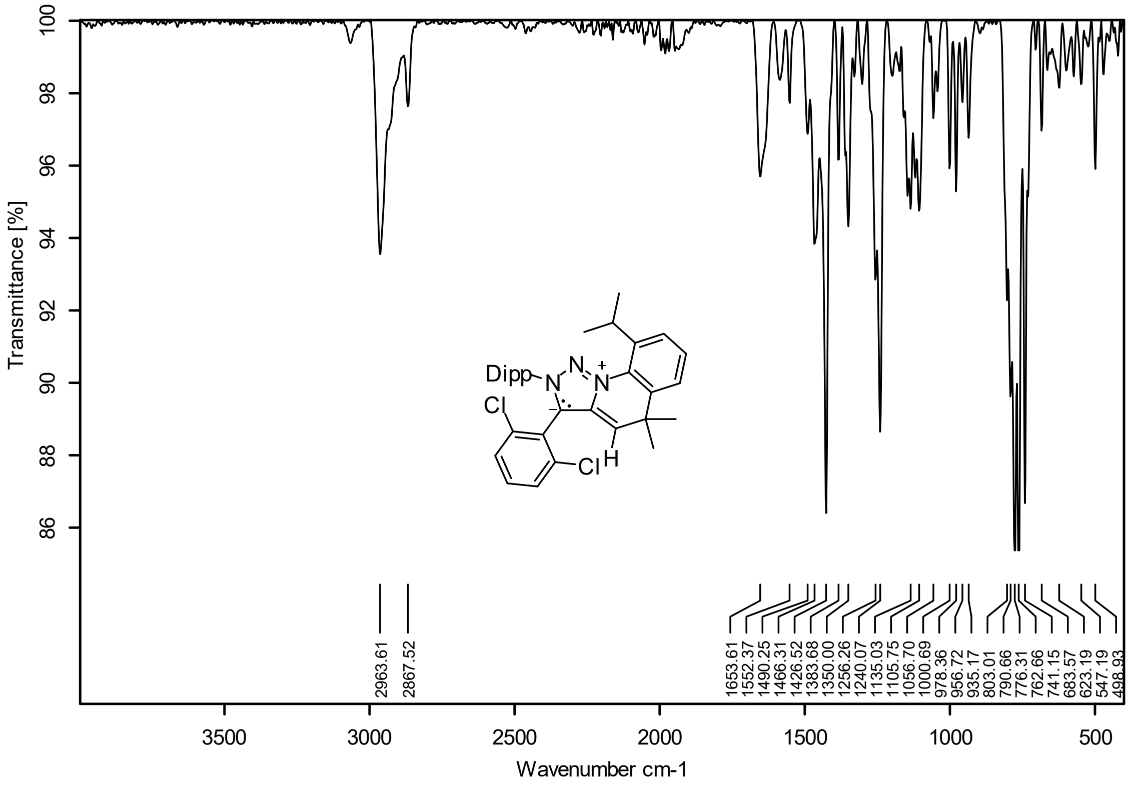

Figure S1.116. ATR IR of **4A**$^{\mathbf{Cl}}$.

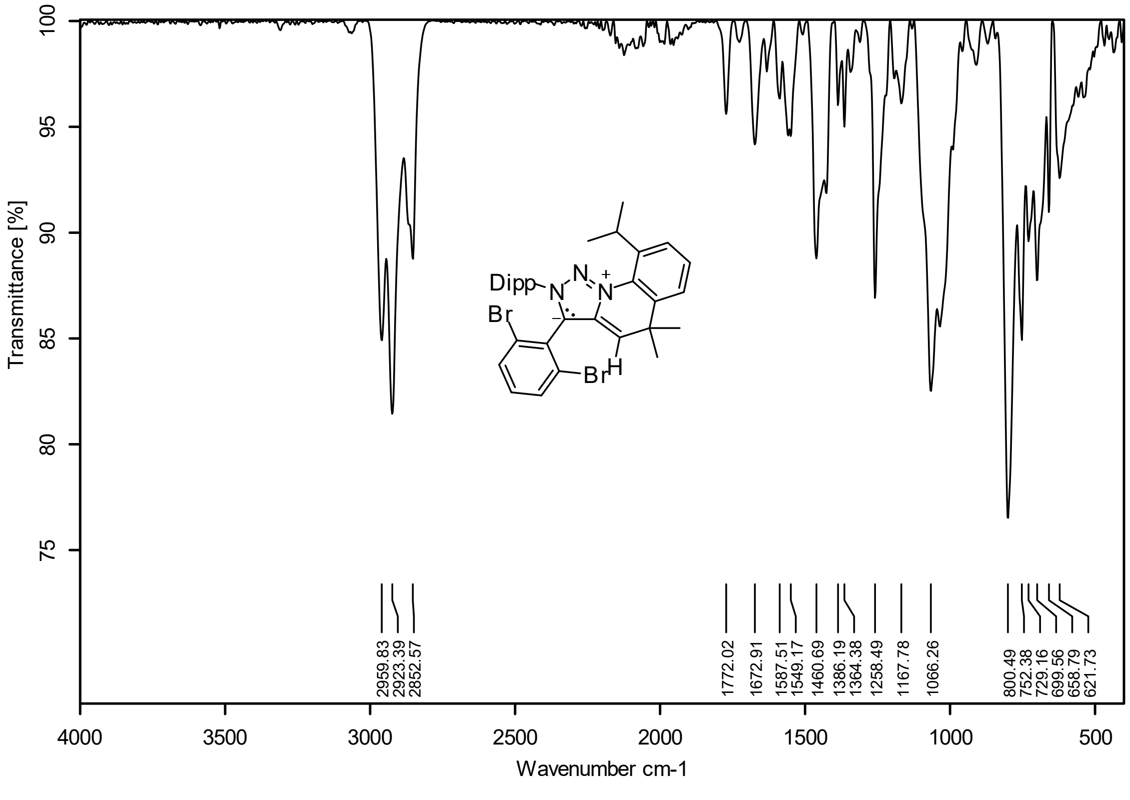

Figure S1.117. ATR IR of **4A**$^{\mathbf{Br}}$.

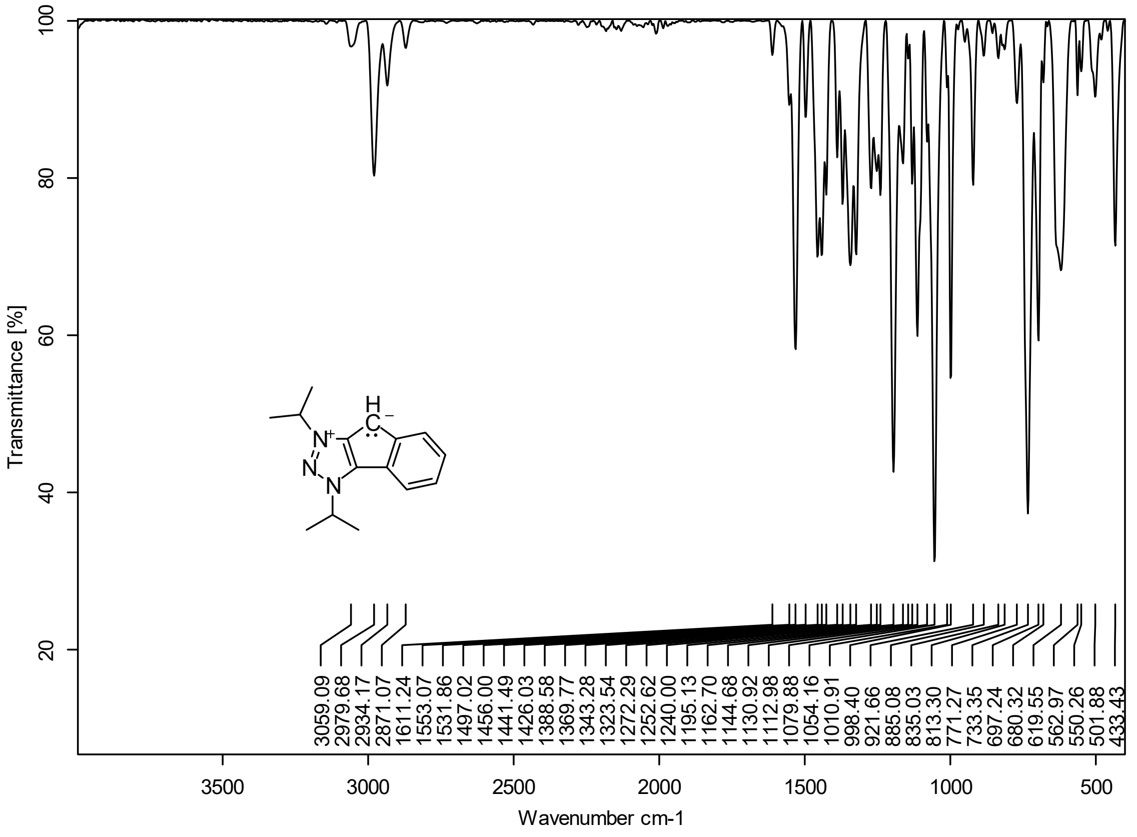

Figure S1.118. ATR IR of **3B**.

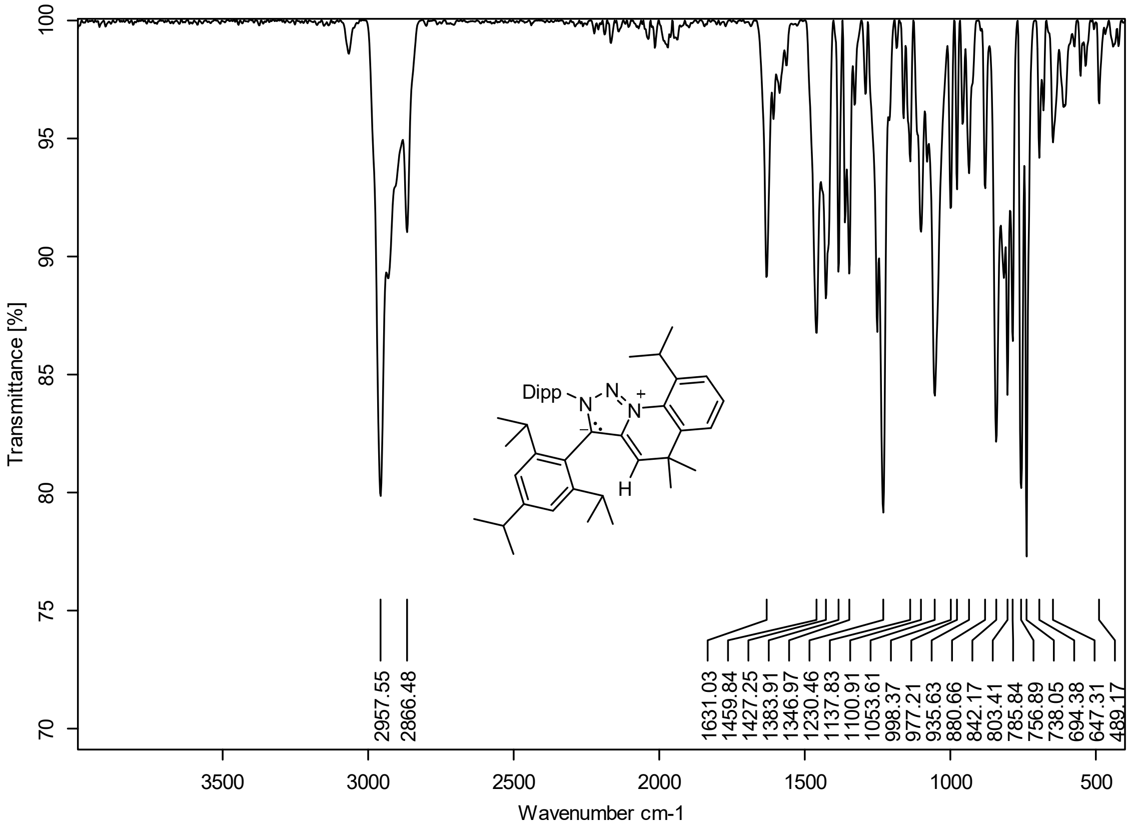

Figure S1.119. ATR IR of **4A**^{iPr}.

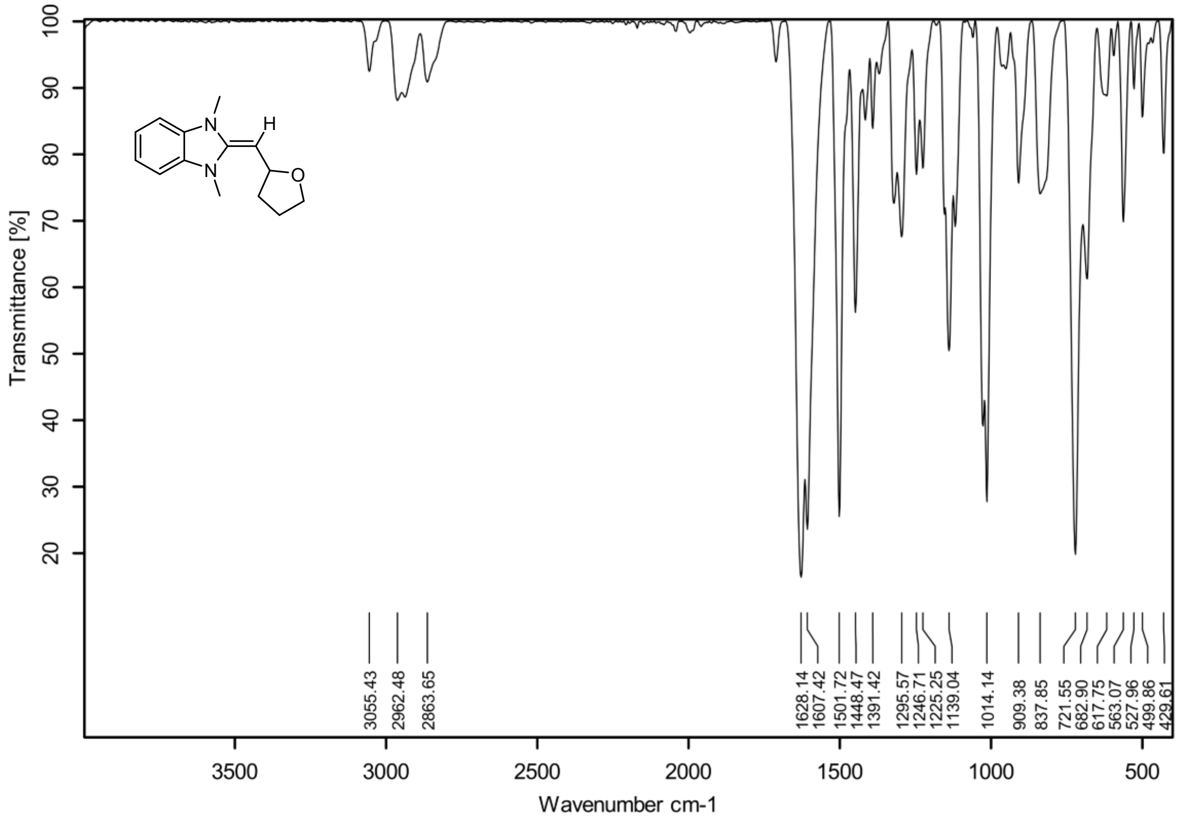

Figure S1.120: ATR IR of **5**.

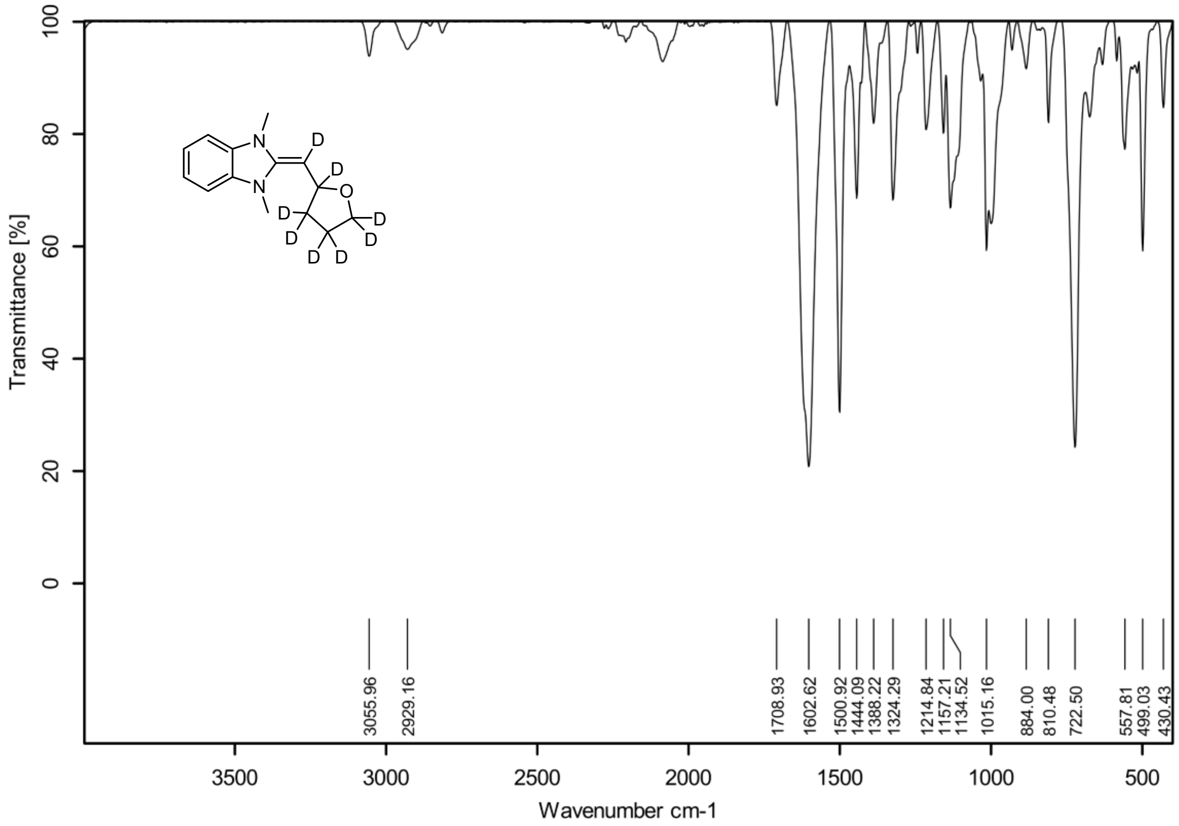

Figure S1.121: ATR IR of **5-d$_8$**.

1.7. SDT

Simultaneous thermogravimetric analysis and differential scanning calorimetry measurements were performed on a Discovery SDT 650 instrument from TA Instruments under a constant nitrogen flow of 100 mL/min. For the measurement a small amount (ca. 8.7 mg) of the powdered sample was placed in a 90 uL alumina crucible and heated from 40 °C to 245 °C with a constant heating rate of 5 K/min.

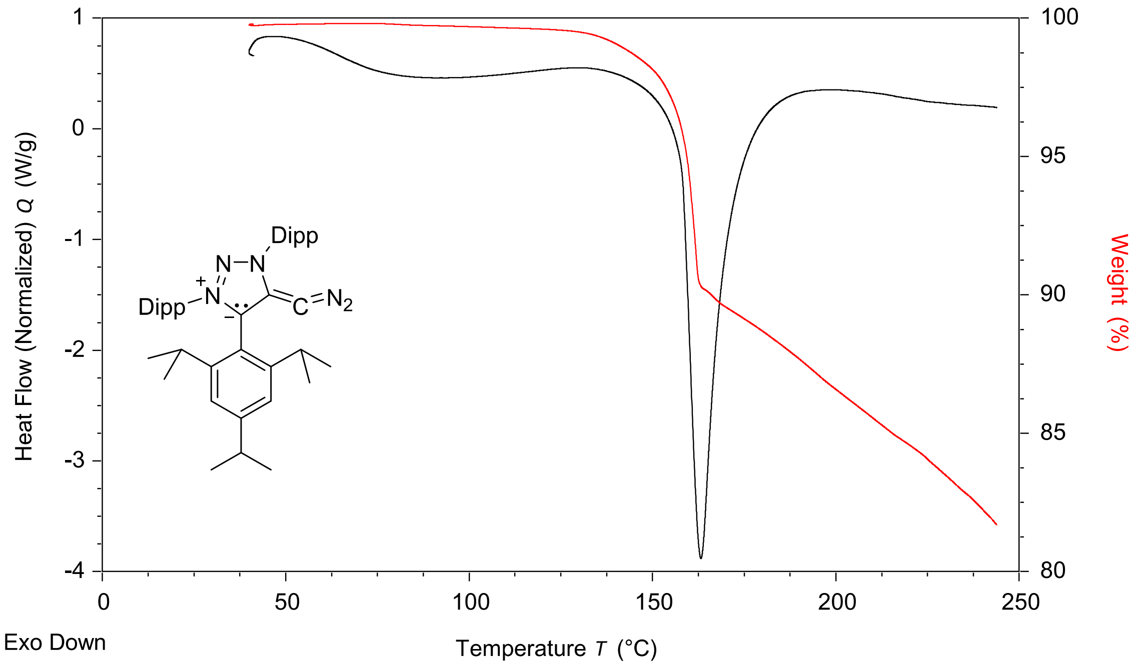

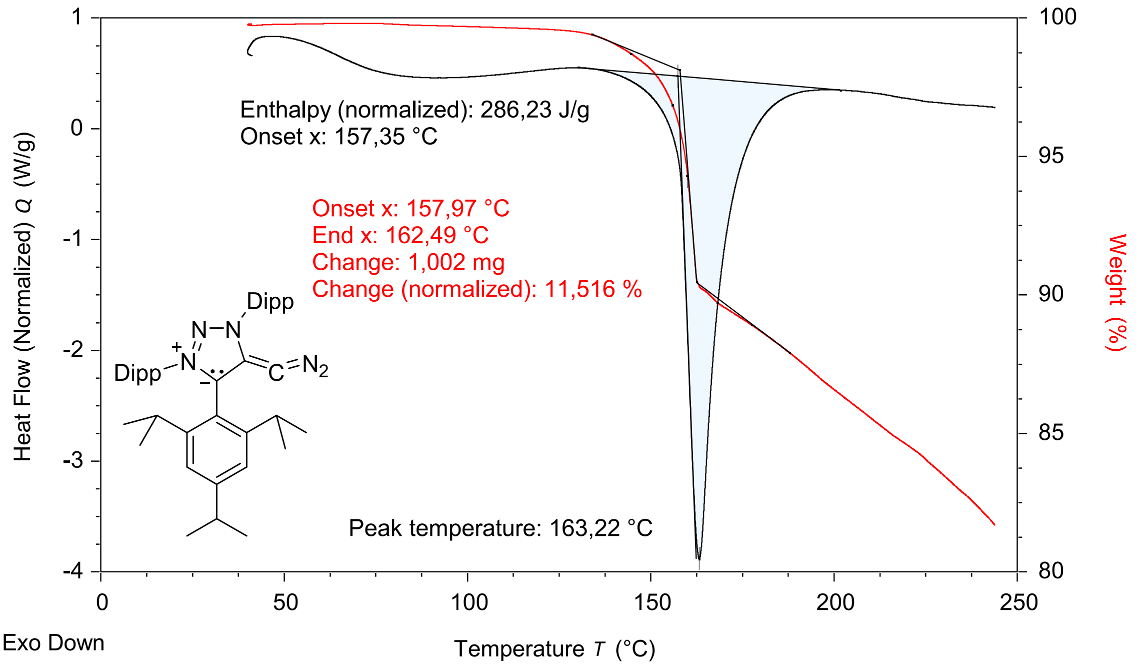

Figure S1.122. Thermogravimetric analysis of **1A**^{iPr} (top: raw data, bottom: analysis).

1.8. UV-vis spectra

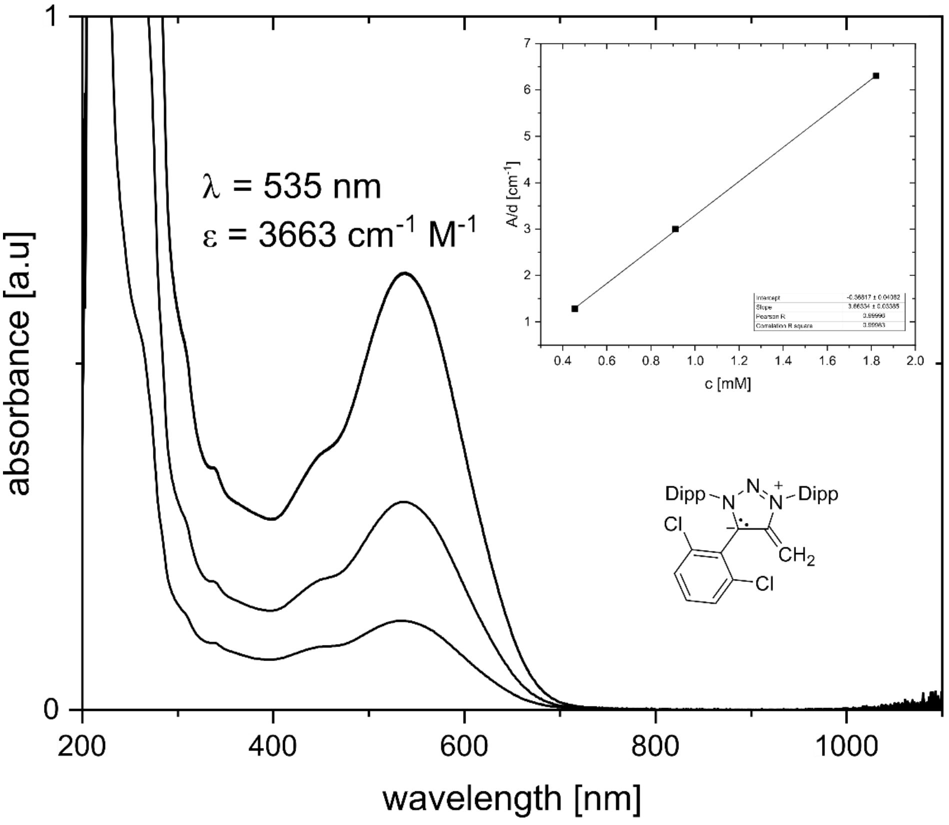

Figure S1.99. UV-Vis spectrum of **dichloro-mNHO** in THF.

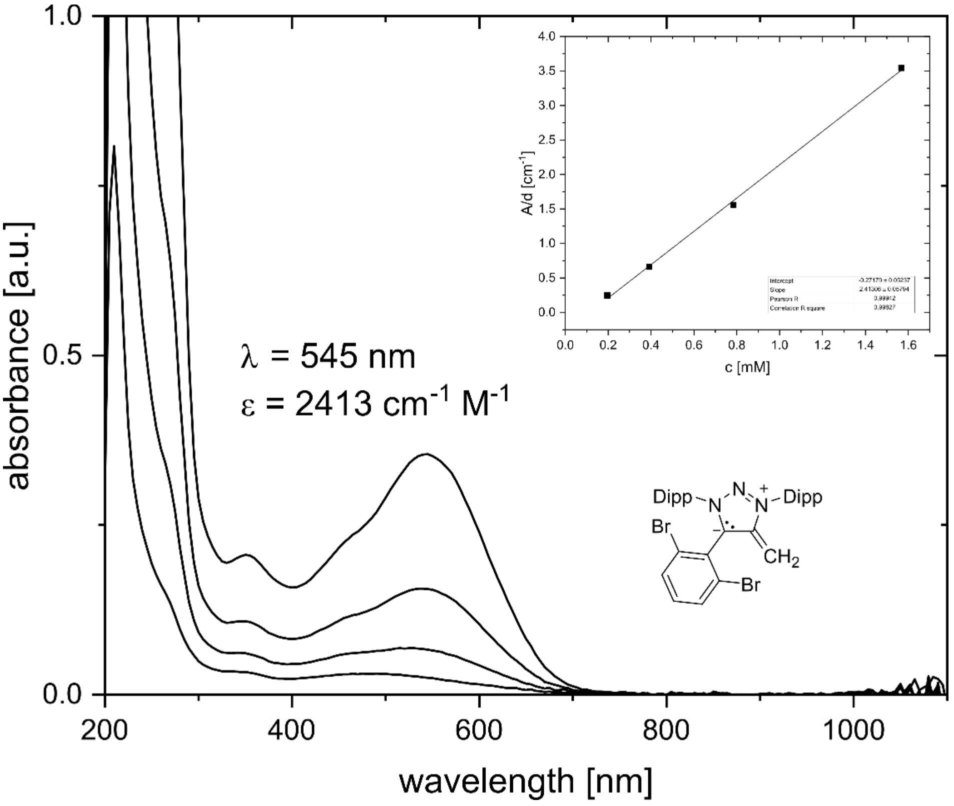

Figure S1.123. UV-Vis spectrum of **dibromo-mNHO** in THF.

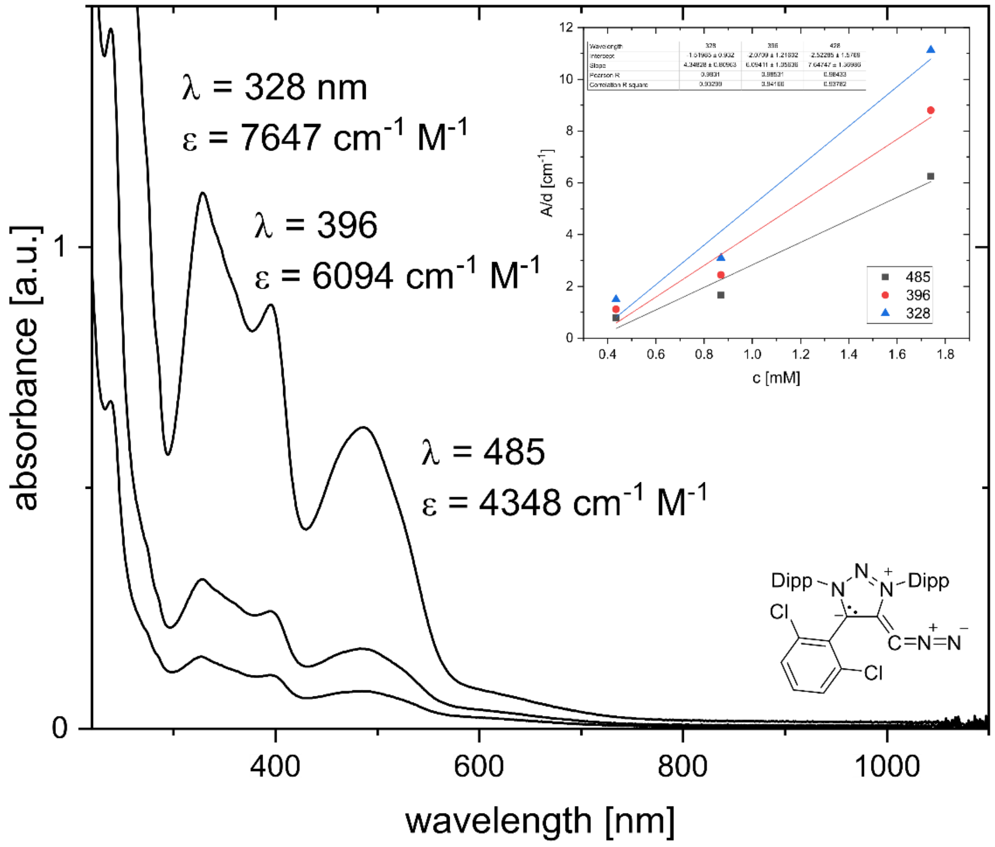

Figure S1.124. UV-Vis spectrum of $\mathbf{1A^{Cl}}$ in THF.

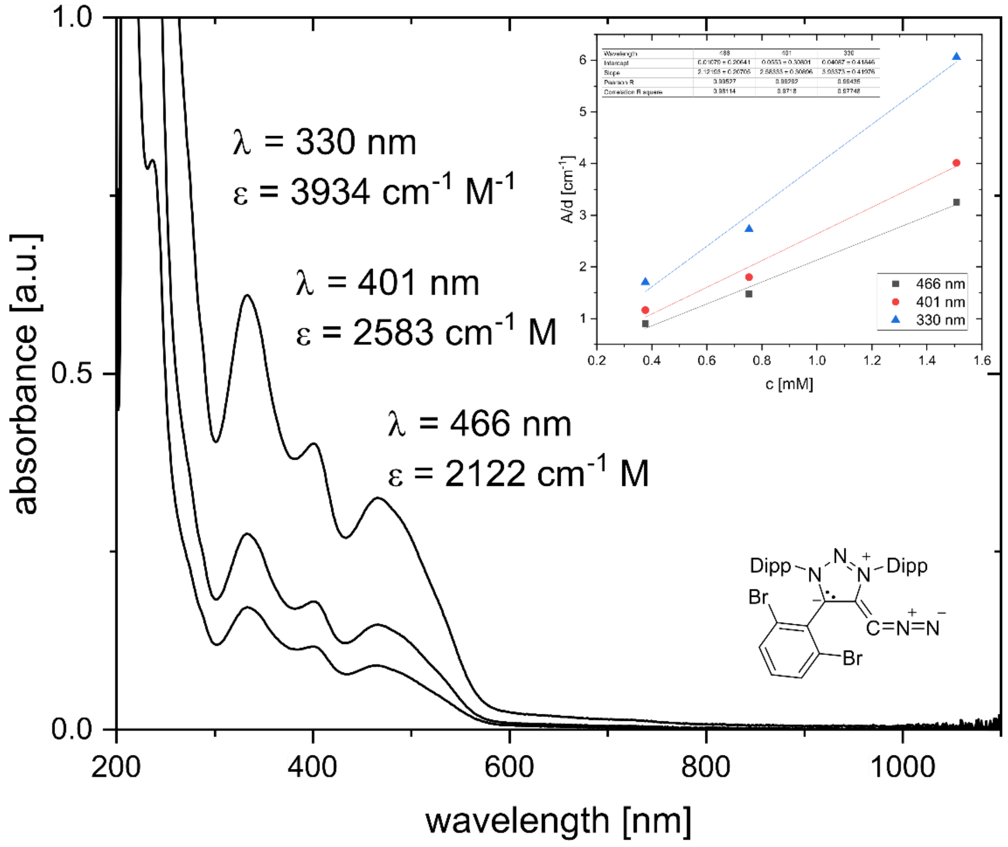

Figure S1.125. UV-Vis spectrum of $\mathbf{1A^{Br}}$ in THF.

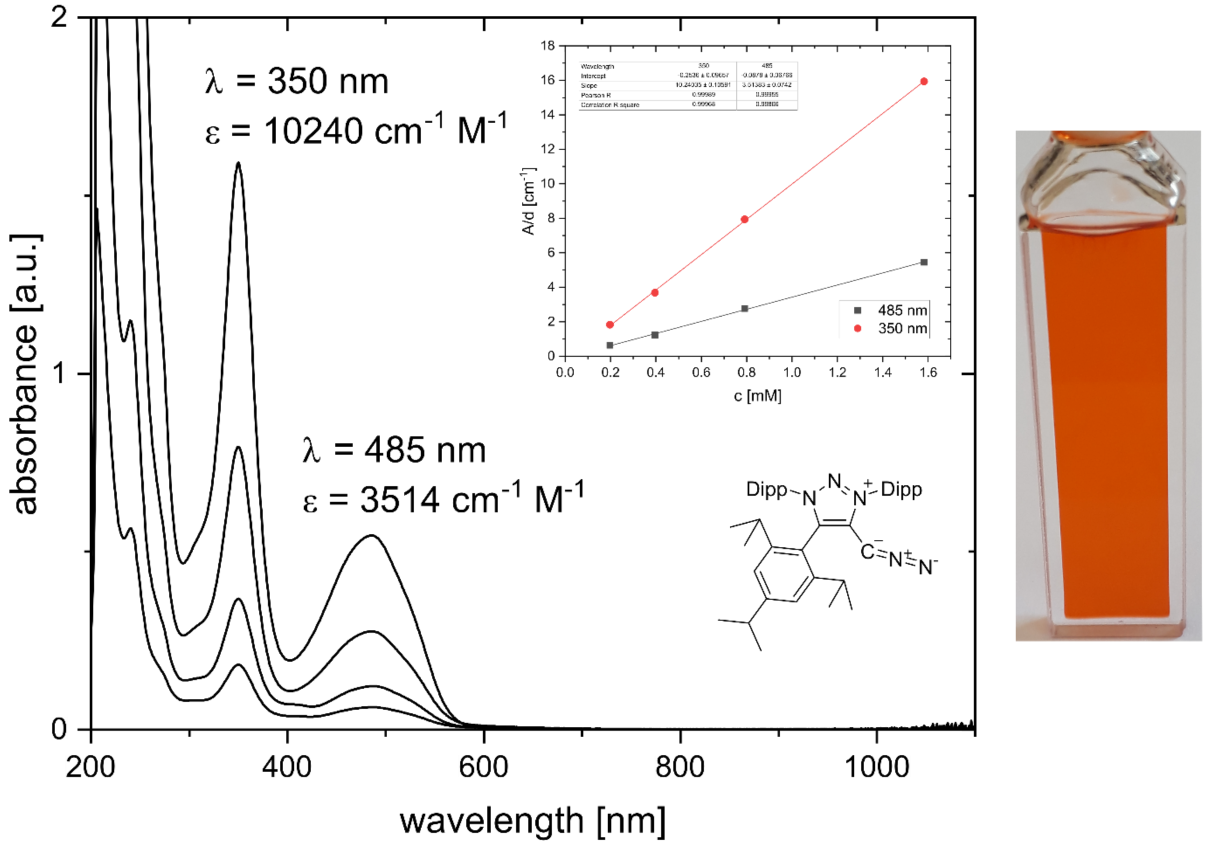

Figure S1.126. UV-Vis spectrum of **1A**^{iPr} in THF.

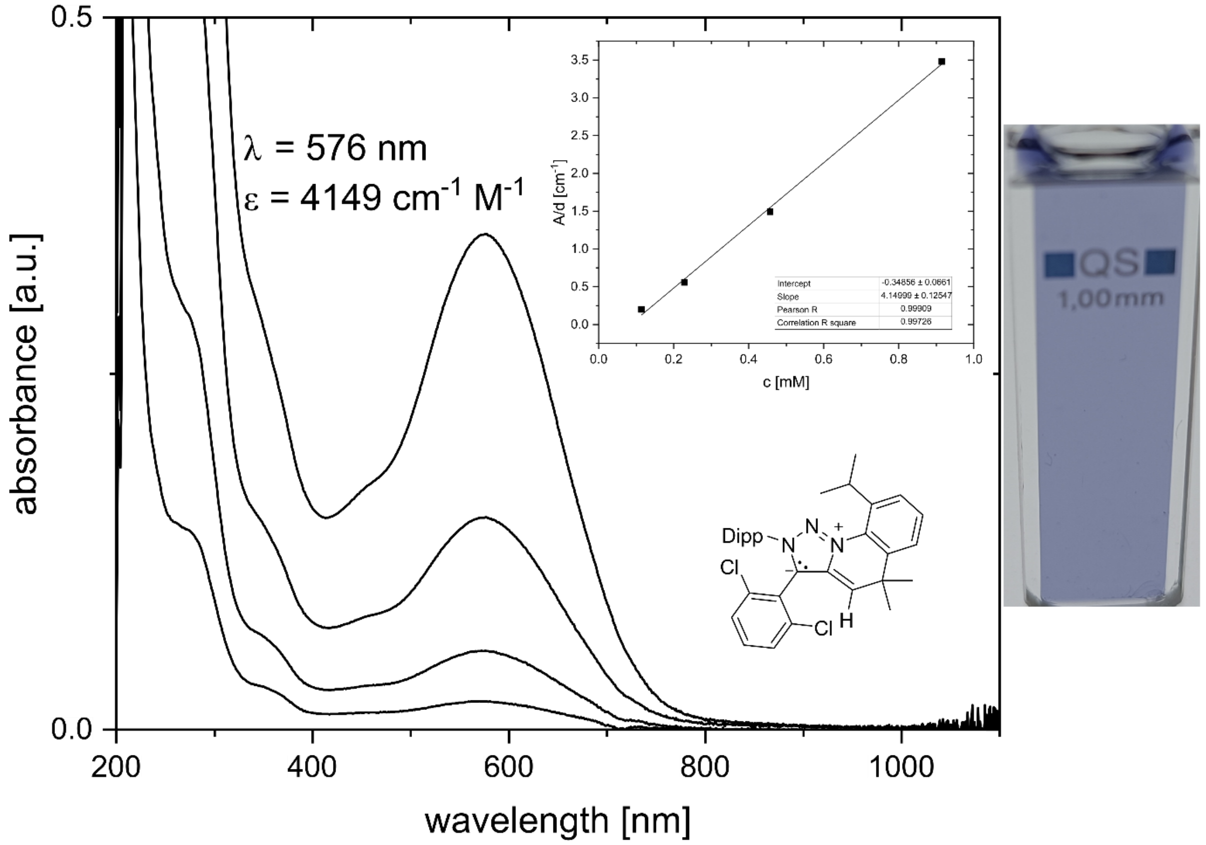

Figure S1.127. UV-Vis spectrum of **4A**^{Cl} in THF.

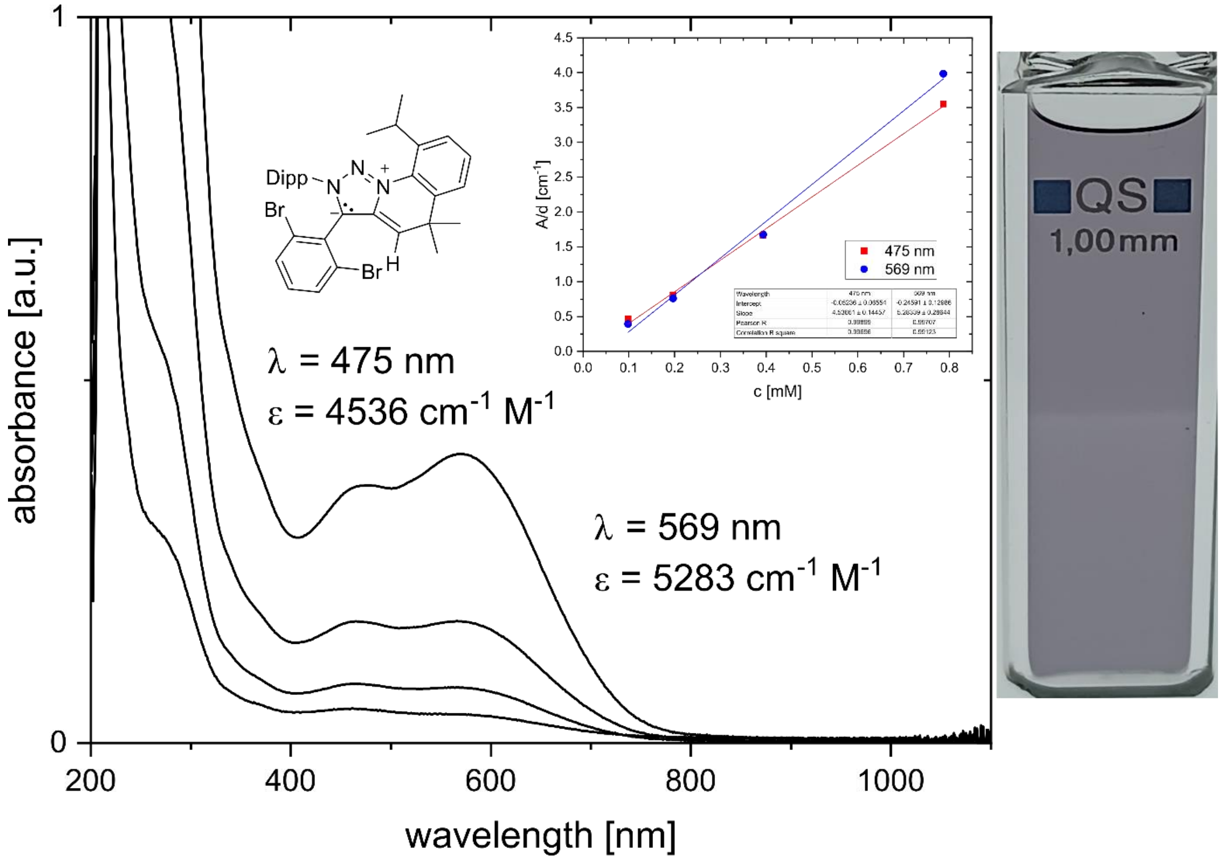

Figure S1.128. UV-Vis spectrum of **4A^{Br}** in THF.

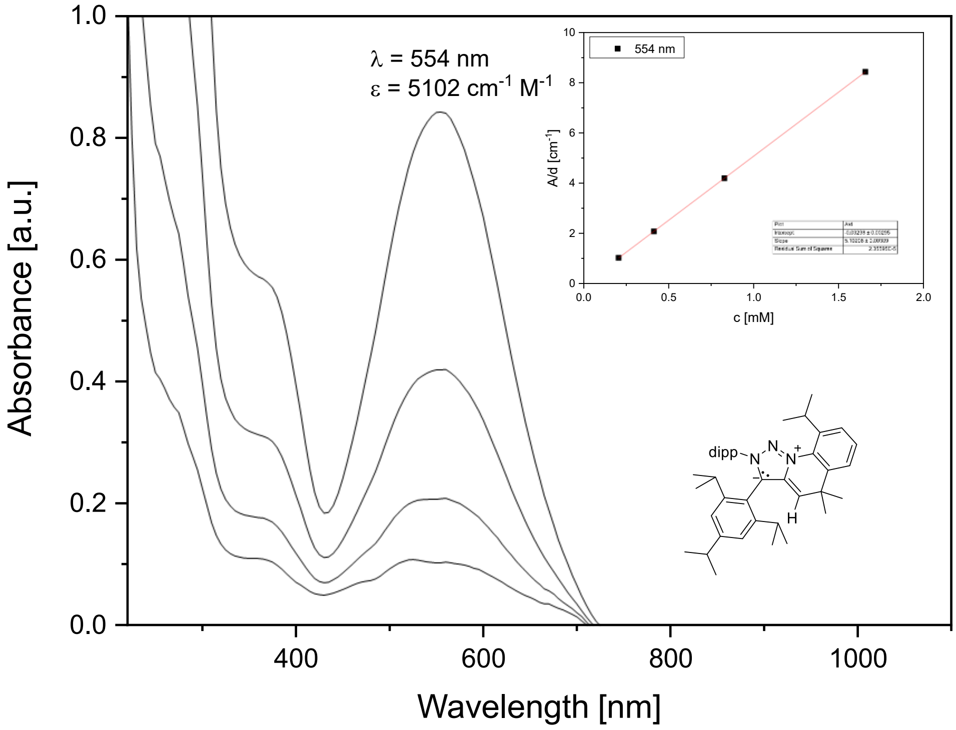

Figure S1.129. UV-Vis spectrum of **4A^{iPr}** in THF.

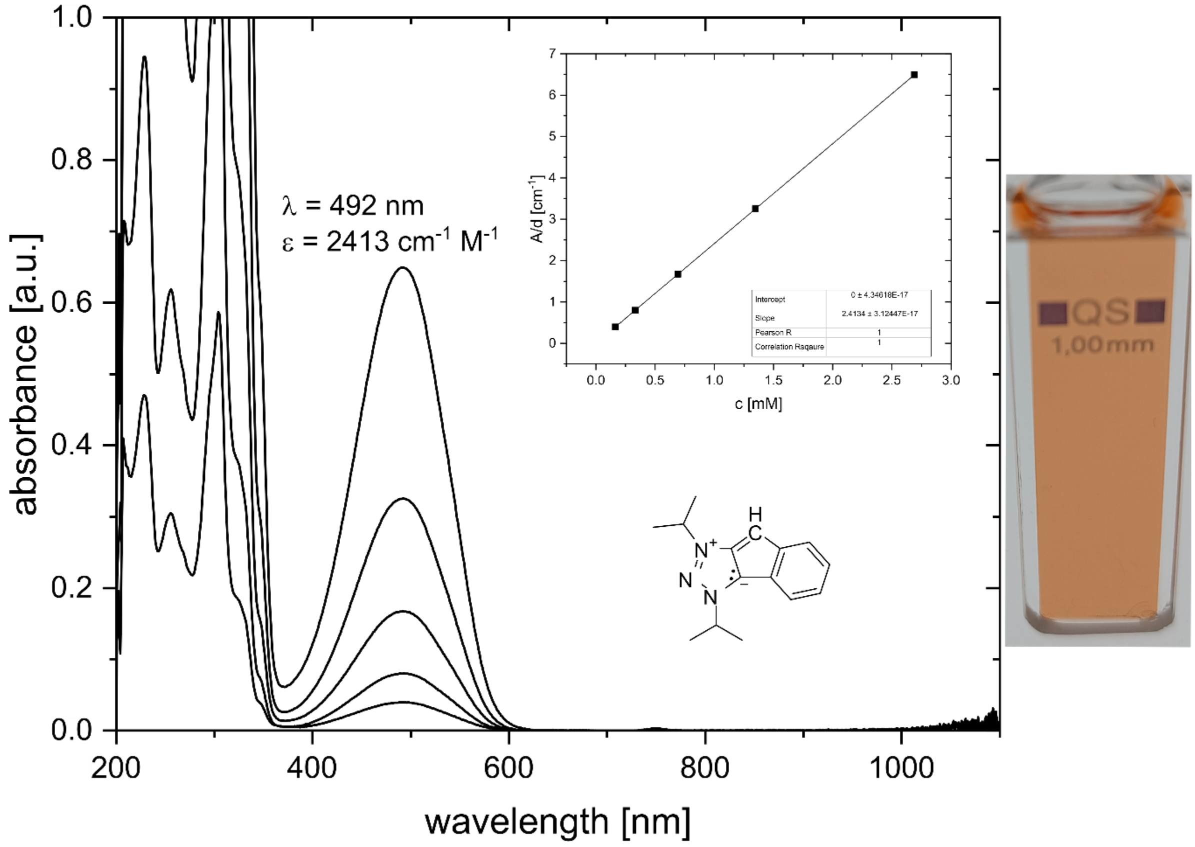

Figure S1.130. UV-Vis spectrum of **3B** in THF.

1.9. X-ray crystallography

Data collection was done on a Bruker D8 Venture four-circle-diffractometer from Bruker AXS GmbH; used detector: Photon II from Bruker AXS GmbH; used X-ray sources: microfocus IµS Cu/Mo from Incoatec GmbH with mirror optics HELIOS and single-hole collimator from Bruker AXS GmbH.

Used programs: APEX3 Suite (v2017.3-0) or APEX4 Suite (v2022.1-1) and therein integrated programs SAINT (Integration) und SADABS (Absorption correction) from Bruker AXS GmbH; structure solution was done with SHELXT[19]. SHELXL[19] (version 2018/3) was used for refinement against F2 until convergence using the full-matrix least-squares routine. All non-hydrogen atoms were refined with anisotropic displacement parameters. The hydrogen atoms were refined isotropically on calculated positions using a riding model. DSR has been applied to treat disordered solvent molecules. Further details on the individual data sets are tabulated in the analytical section.

ShelXle3[20] and OLEX[21] were used as graphical user interface and FinalCIF (https://www.xs3.uni-freiburg.de/research/finalcif) was used for data finalization.[22]

Special Utilities: SMZ1270 stereomicroscope from Nikon Metrology GmbH or Leica M205M Stereomicroscope was used for sample preparation; crystals were mounted on MicroMounts or MicroLoops from MiTeGen using NVH immersion oil; crystals were cooled to given temperature with Cryostream 800 from Oxford Cryosystems.

1A^{Br}

CCDC number	2390052
Empirical formula	$C_{87}H_{98}Br_4C_{-0}N_{10}N_{+0}N_{-0}$
Formula weight	1603.35
Temperature [K]	100(2)
Crystal system	triclinic
Space group (number)	$P\bar{1}$ (2)
a [Å]	12.2324(2)
b [Å]	12.2910(2)
c [Å]	14.2196(3)
α [Å]	71.9180(10)
β [Å]	74.8300(10)
γ [Å]	85.0810(10)
Volume [Å^3]	1961.47(6)

Z	1
ρ_{calc} [g/cm^3]	1.357
μ [mm^{-1}]	2.103
F(000)	830
Crystal size [mm^3]	0.100×0.100×0.100
Crystal colour	yellow
Crystal shape	block
Radiation	MoK_α (λ=0.71073 Å)
2Θ range [°]	5.19 to 52.84 (0.80 Å)
Index ranges	$-15 \le h \le 15$<br>$-15 \le k \le 13$<br>$-17 \le l \le 17$
Reflections collected	40997
Independent reflections	8064<br>R_{int} = 0.0292<br>R_{sigma} = 0.0231
Completeness to θ = 25.242°	99.9 %
Data / Restraints / Parameters	8064/768/489
Goodness-of-fit on F^2	1.030
Final R indexes [$I \geq 2\sigma(I)$]	R_1 = 0.0284<br>wR_2 = 0.0699
Final R indexes [all data]	R_1 = 0.0342<br>wR_2 = 0.0728
Largest peak/hole [eÅ^3]	0.85/-0.45

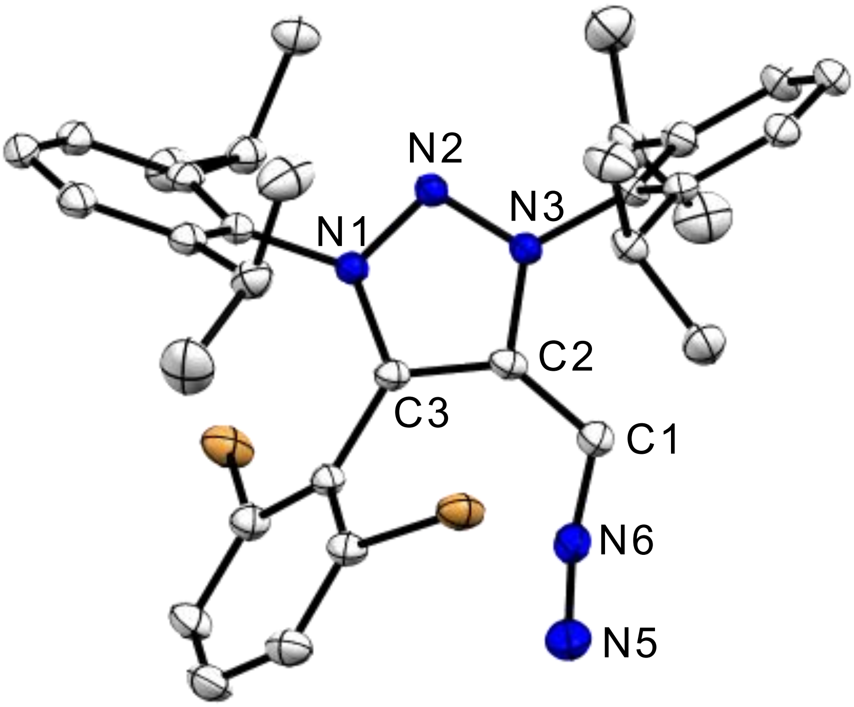

Figure S1.100. X-ray solid-state structure of compound **1A^{Br}**. Thermal ellipsoids are shown with 50% probability. Hydrogen atoms and two toluene molecules omitted for clarity. Selected bond parameters in [Å] and [°]: C1–N6 1.276(3), C1–C2 1.400(3), N6–N5 1.152(3), C2–C3 1.407(3), N6–C1–C2 118.25(17), C1–C2–C3 134.44(17), N5–N6–C1 169.8(2).

When using neutral scattering factors, the HIRSHFELD TEST for the C1–N6 BOND showed significant differences of displacement amplitudes of both atoms in bond direction, as indicated by CHECKCIF LEVEL C ALERT. 230_ALERT_2_C Hirshfeld Test Diff for N6–C1.5.3 s.u. Employment of IONIC SCATTERING FACTORS (C-, N+) calculated and tabulated in https://journals.iucr.org/m/issues/2018/03/00/fq5001/index.html for both atoms significantly improved the R1 value and displacement parameters of atoms.

2. EPR data

2.1. EPR sample preparation

All samples were prepared in a nitrogen-filled glovebox. For Q-band measurements, solutions of the diazo precursors **1A–1F** and **DPC** in thoroughly degassed dry toluene were transferred into 1.6 mm quartz tubes (~1 cm filling height), cooled to –40 °C for 15 min, sealed with BRAND sealing compound (~5 mm length) and immediately flash-frozen in liquid nitrogen outside the glovebox. The precursor concentrations were chosen to optimize the triplet signal intensity by finding a compromise between concentration and transparency of the solutions to UV light (in Figures 1, 3 and S2.1–2.2 the concentrations were as follows: **1A**^{Cl}, **1A**^{Br}, **1A**^{iPr}, **1A**: 20 mM; **1B**, **1E**, **1F**: 15 mM; **1C**: 8mM; **1D**: 2mM, **DPC**: 15 mM). The samples were covered in aluminum foil inside the glovebox and transported to the spectrometer under exclusion of light.

For X-band measurements, toluene solutions of the diazo precursors were transferred into 4.8 mm quartz tubes (~2 cm filling height), sealed with the sealing compound (~1 cm length) and immediately flash-frozen in liquid nitrogen outside the glovebox. 20 mM solutions were used in the experiments shown in Figures 4, 5, S2.12–2.13 (with the exception of 4mM used for **1C**).

2.2. EPR experimental details

Q-Band pulse EPR measurements were carried out at 6 K using a Bruker Elexsys E580 spectrometer equipped with a 150 W TWT amplifier, Bruker EN 5107D2 resonator, Oxford Instruments CF935 continuous-flow helium cryostat and Oxford Instruments MercuryiTC temperature controller. Field-swept EPR spectra were detected via the free induction decay (FID) signal to avoid strong nuclear modulation artifacts found in the electron spin echo detected spectra. The microwave (MW) $\pi/2$ pulse was 500 ns. The triplet species were generated by irradiating the diazo precursors in frozen toluene solution at 10 K using a Hg arc lamp (LOT LSB610U) inside the resonator until the triplet species intensity reached a plateau (approx. 1 to 2 h). The FID-detected spectra were pseudo-modulated[23] using the *fieldmod* function of the *EasySpin* package[24] with a modulation amplitude of 5 mT.

Orientation-selective Davies[25] ENDOR spectra were collected with stochastic detection[26] at 6 K using an AR 600 W radiofrequency (RF) amplifier (AR 600A225A). The following microwave pulse sequence was used: π−T−$\pi/2$−τ−π− τ−echo. The RF pulse was applied during the time interval T and had a length of 30 µs; the MW inversion π pulse was 28−30 ns; the $\pi/2$ and π detection pulses were 14 and 28 ns, respectively; the inter-pulse delay τ was 340 ns. Imperfections in the ^{1}H and ^{14}N ENDOR signal subtractions (Figure 3) are due to the

variations in the MW frequency and output power of the RF amplifier, as well as EPR line shape changes upon ^{13}C labeling.

Temperature-dependent X-band continuous wave (CW) EPR measurements in the temperature range of 8 to 50 K were carried out using a Bruker Elexsys E500 spectrometer equipped with a Bruker ER 4119 HS resonator, Oxford Instruments ESR 900 cryostat and Oxford Instruments MercuryiTC temperature controller. The spectra were recorded under non-saturating conditions, with a modulation amplitude of 17 G. The triplet species was generated by irradiating the diazo precursor in frozen toluene solution at 10 K using a 395 nm fiber-coupled LED (Thorlabs M395FP1) inside the resonator until the triplet species intensity reached a plateau (~40 min).

X-Band CW EPR stability measurements in the temperature range of 94–170 K were carried out using a benchtop Magnettech ESR5000 spectrometer. The spectra were recorded under non-saturating conditions (1 mW); the modulation amplitude was 9.5 G. The triplet species were generated by irradiating the diazo precursors (4mM for **1C,** 20 mM for others) in frozen toluene solution at 94 K inside the resonator until the triplet species intensity reached a plateau (~30 min). A Xe lamp was used for **I**, $\mathbf{1A^{iPr}}$ and **1B**, and a 395 nm LED (Thorlabs M395FP1) for $\mathbf{1A^{Cl}}$, $\mathbf{1A^{Br}}$, **1C**, **1E**, **1F**.

EPR and ENDOR simulations were performed using the *EasySpin* package[24]. The intensities of the simulated Q-band half-field ($M_S = -1 \leftrightarrow 1$) EPR signals at ~550 mT were manually reduced to account for a difference in transition probabilities (Figures 1 and S2.1–2.2). To minimize the number of simulation parameters, we used an isotropic g-factor g_{iso} = 2.0023 and only the anisotropic EPR linewidth parameter *Sys.HStrain* (taken into account by *EasySpin* for ENDOR simulations). The combination of individual *x*, *y* and *z* components of *Sys.Hstrain* were unique for each triplet species and varied between 115 and 320 MHz. For ^{13}C ENDOR simulations, the *salt* function was used with an excitation width parameter *Exp.ExciteWidth* = 84 MHz, *Opt.GridSize* = [180 3] and linewidth *Sys.lwEndor* = 1.6 MHz for the vinylidenes $\mathbf{2A^{iPr}}$ and **2E**, and 5 MHz for **DPC** (except for the low-frequency features marked green in Figure 3, which were simulated using a 1.6 MHz linewidth).

2.3. EPR spectra and energy level diagrams

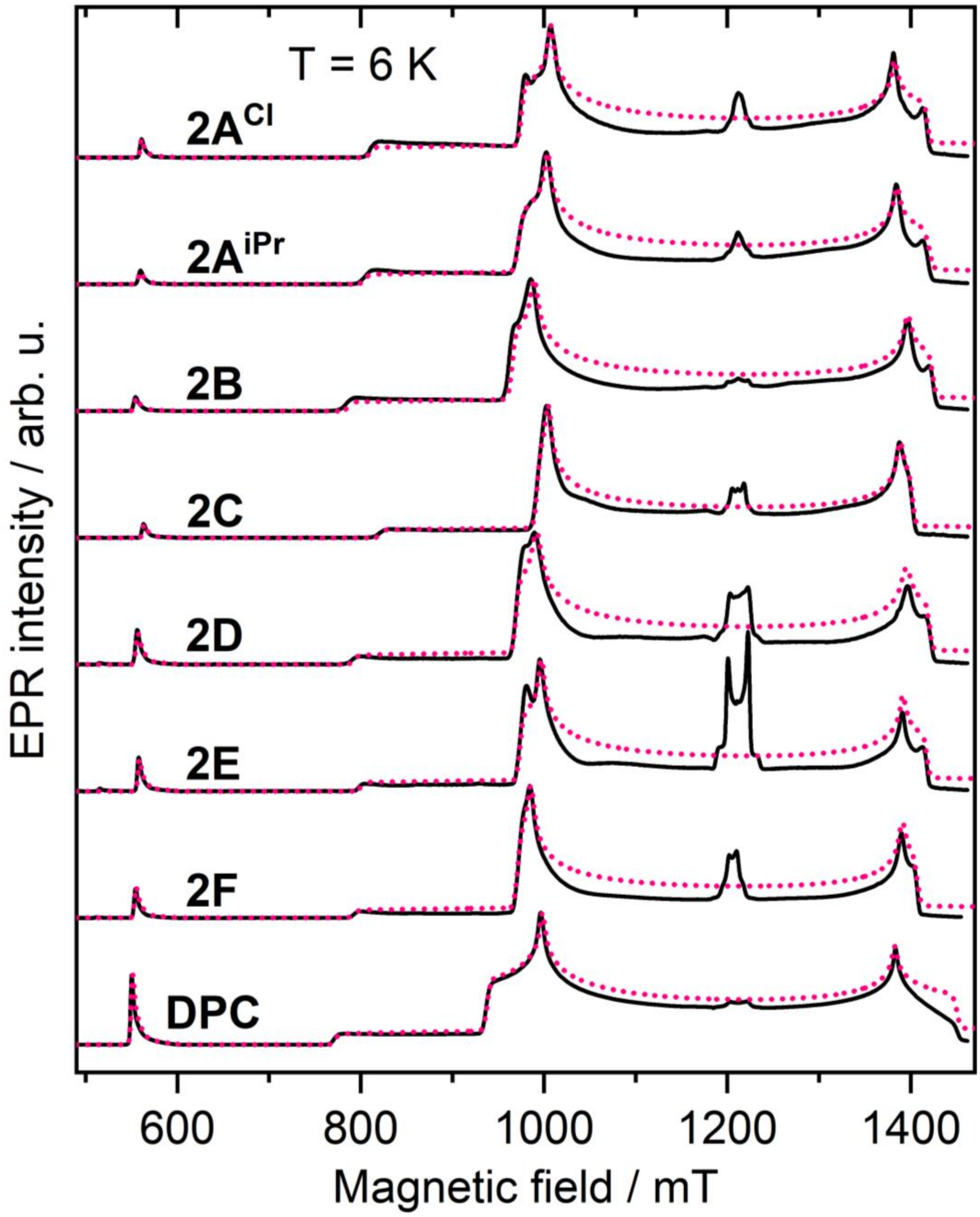

Figure S2.1. FID-detected Q-band EPR spectra (black solid lines) acquired at 6 K following the photolysis of the precursor series (Hg arc lamp, ~1 h at 10 K) in frozen toluene solution. The spectra are overlaid with the best fits assuming S = 1 species (purple dotted lines). The simulation parameters are given in Table 1. Simulated half-field (M_S = −1 ↔ 1) transition intensities were manually reduced to account for a lower transition probability. Pseudo-modulated traces are shown in Figure 1.

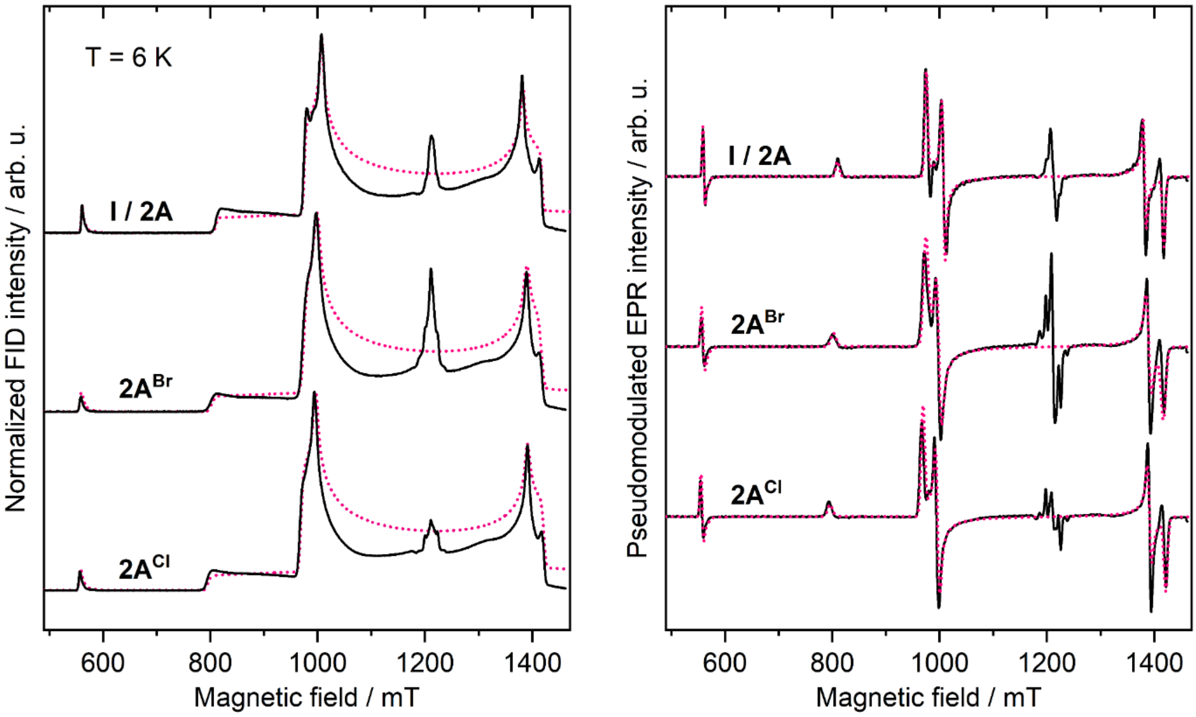

Figure S2.2. FID-detected Q-band EPR spectra (black solid lines) of **I** and its halogenated variants (**2A^{Br}** and **2A^{Cl}**) acquired at 6 K following the photolysis of the precursors, overlaid with the best fits (purple dotted lines). Same conditions as in Figures 1 and S2.1. The simulation parameters are given in Table 1. Left: as recorded; right: pseudo-modulated traces.

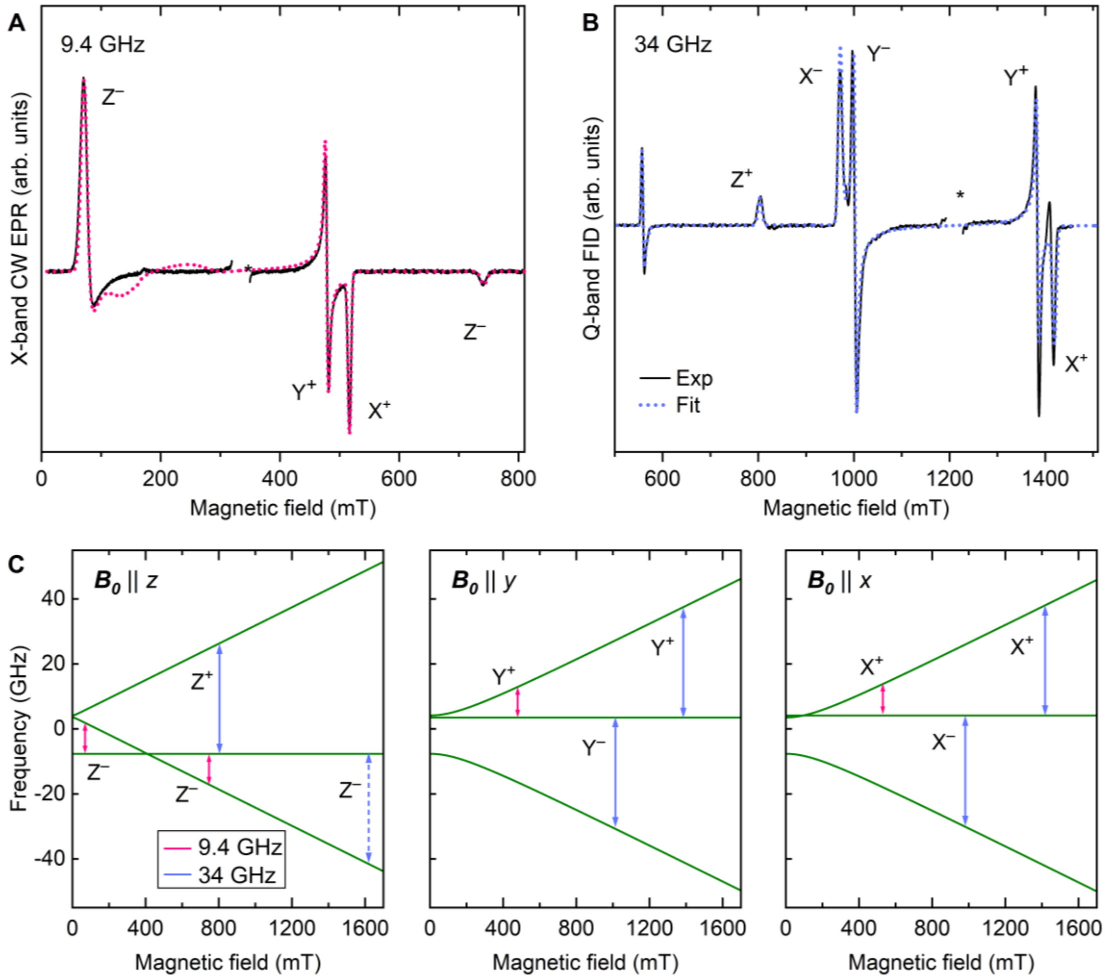

Figure S2.3. (**A**) CW X-band EPR spectrum and (**B**) FID-detected Q-band EPR spectrum of vinylidene **2A**$^{\mathbf{iPr}}$ (black solid lines) acquired at 10 and 6 K, respectively, after the photolysis of the precursor in frozen toluene solution. The spectra are overlaid with the best fits assuming an S = 1 species (pink and blue dotted lines, see Table 1). Asterisks: the radical signal is omitted for clarity. (**C**) Calculated spin-energy levels for the three orientations of the external magnetic field along the canonical axes of the zfs tensor of vinylidene **2A**$^{\mathbf{iPr}}$. Pink and blue arrows indicate X- and Q-band EPR transitions, respectively. The simulations were performed using D = 0.382 cm^{-1}, E/D = −0.027 and g = 2.0023. The dashed arrow for $\boldsymbol{B_0} \parallel z$ indicates a Q-band transition outside of the magnet's range.

EPR line shape comparison for ^{13}C-labeled and natural-abundance samples:

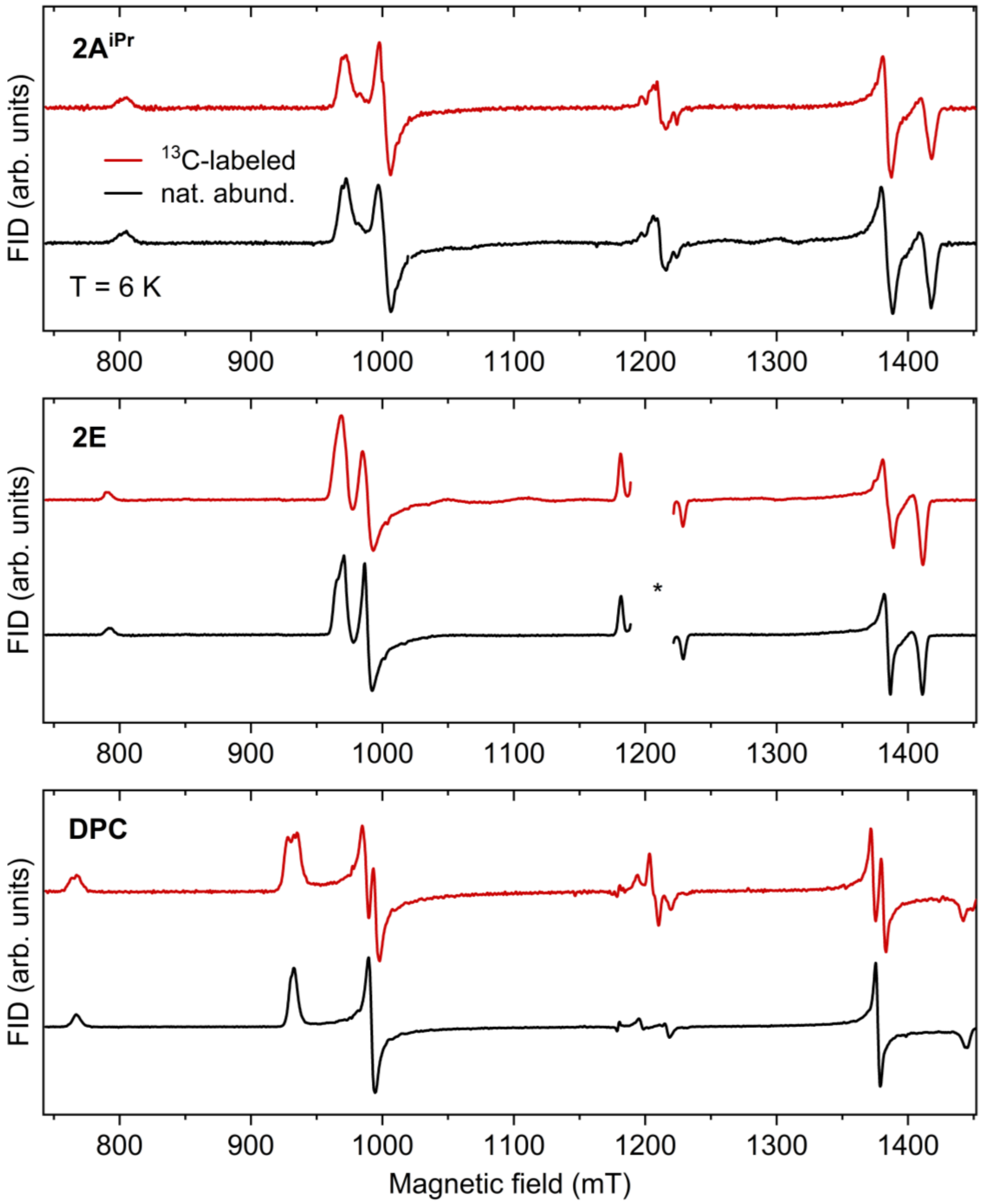

Figure S2.4. Pseudo-modulated FID-detected Q-band EPR spectra of vinylidenes **2A^{iPr}**, **2E** and carbene **DPC** in toluene collected at 6 K for ^{13}C-labeled (red) and natural abundance (black) samples. No hf splitting is observed in the EPR spectra of the vinylidenes upon ^{13}C labeling. In the case of **DPC**, the hf interaction is large enough for the splitting to be observed directly in a field-swept spectrum. Asterisk: the photolysis byproduct signal is omitted for clarity.

2.4. Subtraction of ^{1}H and ^{14}N ENDOR signals

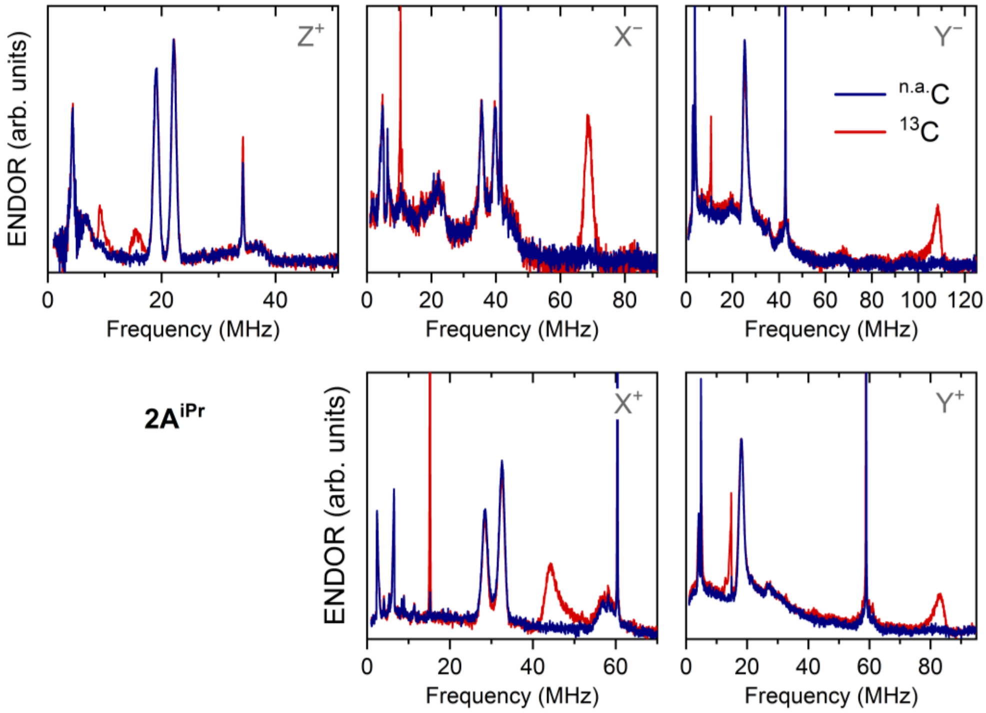

Figure S2.5. Davies ENDOR spectra of **2A^{iPr}** recorded at canonical field positions for a ^{13}C-labeled (red) and a natural abundance (blue) sample. Subtraction results for each field position are shown in Figure 3.

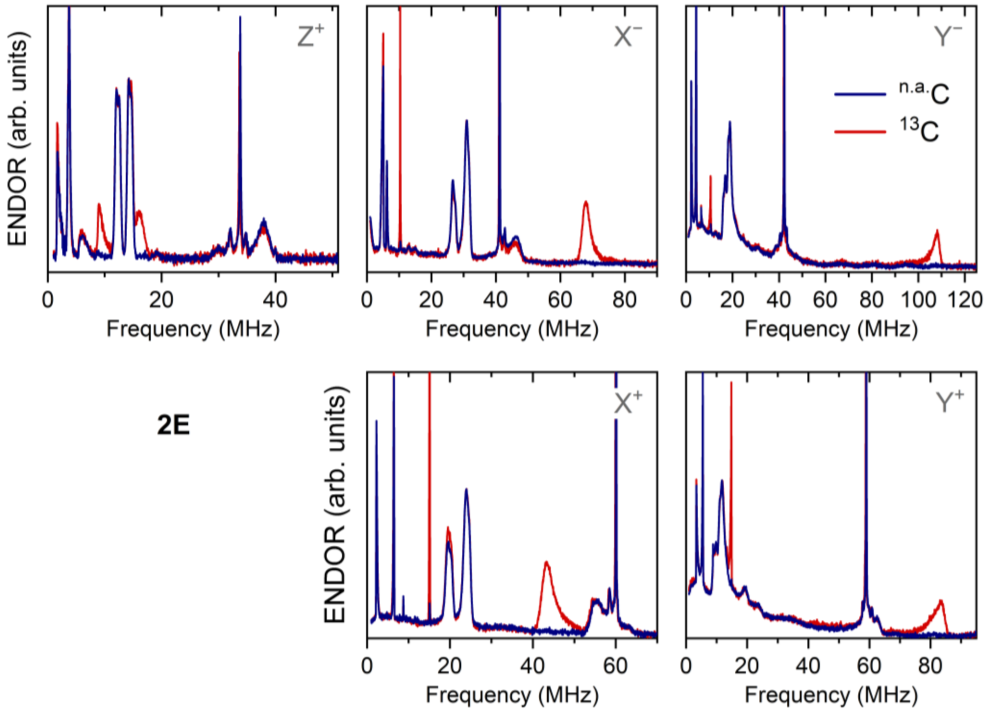

Figure S2.6. Davies ENDOR spectra of **2E** recorded at canonical field positions for a ^{13}C-labeled (red) and a natural abundance (blue) sample. Subtraction results for each field position are shown in Figure 3.

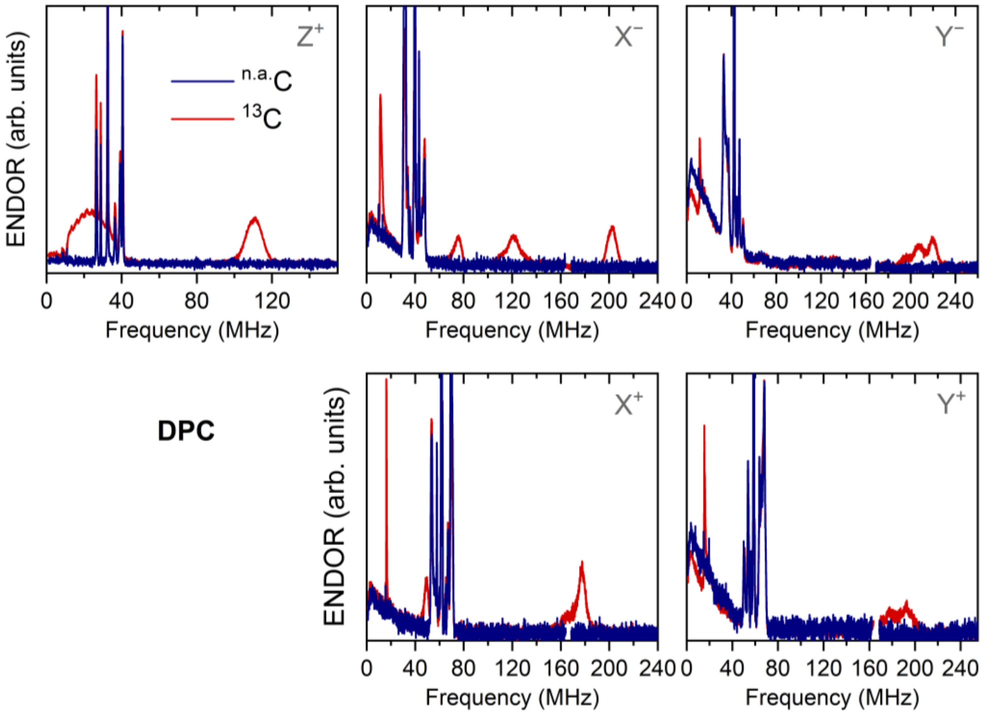

Figure S2.7. Davies ENDOR spectra of the **DPC** carbene recorded at canonical field positions for a ^{13}C-labeled (red) and a natural abundance (blue) sample. Subtraction results for each field position are shown in Figure 3.

2.5. ^{13}C ENDOR simulations

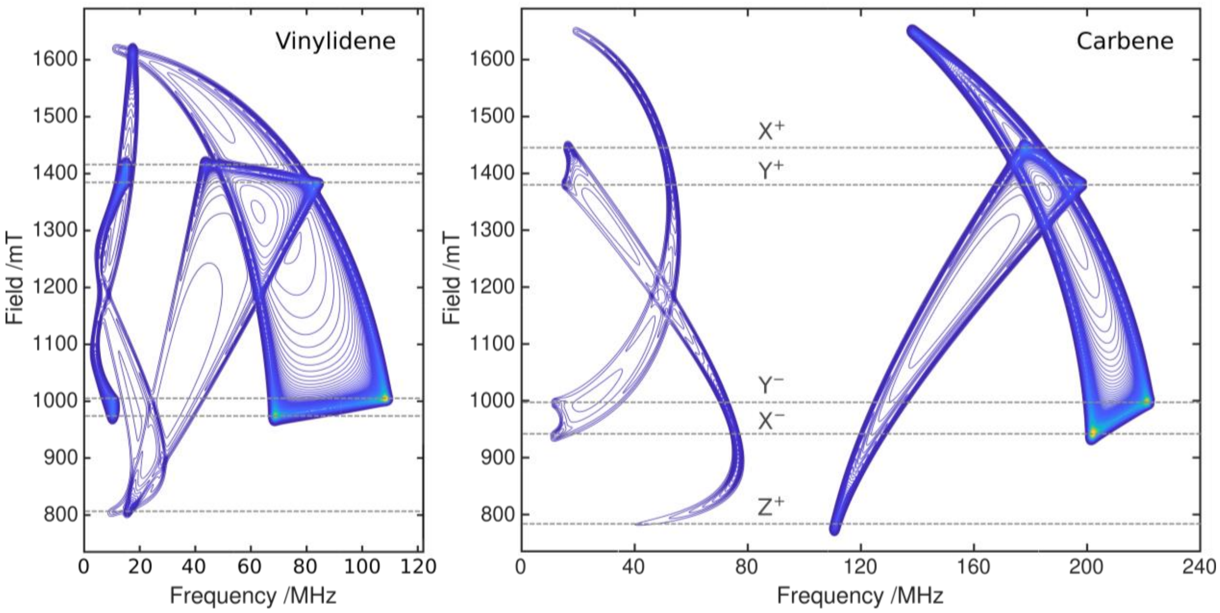

Figure S2.8. Simulated ^{13}C ENDOR patterns obtained across the full EPR absorption envelopes of the vinylidene **2A^{iPr}** (left) and carbene **DPC** (right). The same frequency scale was used for both plots. The spin-Hamiltonian parameters are given in Table 1. Dashed horizontal lines mark the canonical field positions, where ENDOR signals were measured (see Figure 3).

2.6. Analysis of the ^{14}N hyperfine and quadrupole interaction tensors

The high-intensity ENDOR features detected for natural-abundance **2A^{iPr}** (seen in Figure S2.5) could be simulated by introducing a single ^{14}N nucleus with a predominantly isotropic HF tensor and a quadrupole interaction (QI) tensor resolved along the X and Z directions of the ZFS. Experimental data with simulations are shown in Figure S2.8 (left panel). The set of simulation parameters was not unique and was obtained by assuming collinearity of the three tensors (ZFS, HF, QI) in order to reduce the number of fitting parameters.

Table S2.1 shows comparison of the fitted HF and QI values and those calculated for the three nitrogen nuclei (termed N1–N3) in the **2A^{iPr}** heterocycle. The fitted values match the predictions for the ^{14}N nucleus closest to the terminal carbon (N3). The two remaining ^{14}N nuclei are weakly coupled to the electron spin, as reflected in the low calculated spin densities (see Figure S3.1); it is not immediately clear why they do not produce ENDOR features of comparable intensity to that of N3.

Computations predict no significant influence of substituent groups on the electron spin delocalization over the heterocycle (Figure S3.1). Accordingly, our ^{14}N ENDOR data for compound **I**[27], featuring the same 1,2,3-triazole ring, were nearly identical to those of **2A^{iPr}**.

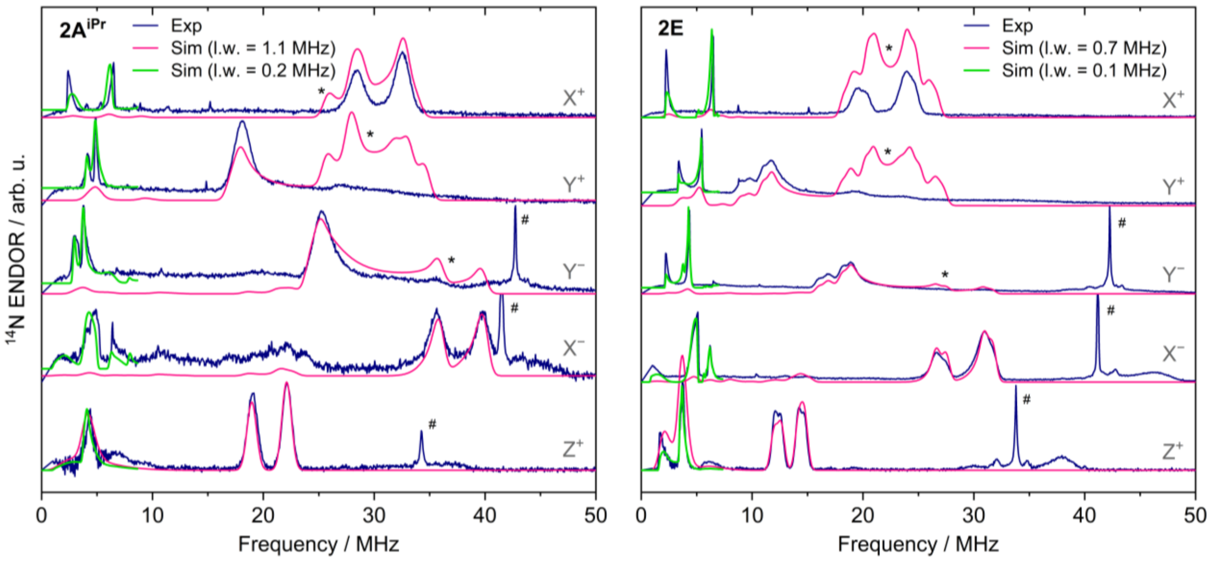

Figure S2.9. Davies ENDOR spectra of natural-abundance **2A^{iPr}** (left) and **2E** (right) recorded at the canonical field positions (blue traces) with simulations performed using two values of the FWHM line width (pink and green traces). Asterisks (*) mark ^{14}N spectral features originating from non-canonical orientations, which are suppressed in the experiment; pound signs (#) mark ^{1}H spectral features.

In contrast, the structure of **2E** is nominally symmetric with respect to the C—C bond (Figure S3.1). Thus, the two ^{14}N nuclei in the heterocycle are predicted to possess identical HF and QI canonical values (Table S2.1). In the experiment, however, an additional splitting is partially resolved at all canonical field positions, indicating a small inequivalence between the two ring nitrogens (Figure S2.8, right panel). This is likely due to the effects of the surroundings in a frozen solution. The conformation of the triplet vinylidene is determined by the conformation of the diazo-precursor, locked when the sample is flash-frozen prior to UV illumination. Upon photochemical liberation of dinitrogen at 10 K, the resulting triplet-state molecule remains in its initial “unrelaxed” conformation, which may lead to an asymmetry between the two ring

nitrogens. Specifically, the bond angle of the C−C−N_2 group in the **1E** diazo-precursor is ~120° as determined by X-ray diffraction.[5] This leads to an inequivalence of the two N−Dipp moieties, which is preserved in the photochemically generated vinylidene **2E**. Such reasoning is supported by the fact that the inequivalence is not found in **2F** despite a similar C−C−N_2 angle in **1F**.[7] Here, no additional splitting is observed in ^{14}N ENDOR spectra (Figure S2.9), as the two N−CH_3 moieties are not affected by the direction of the CN_2 group. While annealing should drive the structure to its relaxed state–which was assumed in the calculations–we could not perform such experiments due to technical limitations of our equipment. All in all, the inequivalence effect in **2E** is small, and the computed ^{14}N HF and QI tensors show a good match with the experimentally fitted ones (Table S2.1).

Table S2.1. Comparison of the computed and experimentally fitted ^{14}N HF and QI tensors in **2A^iPr** and **2E**. The HF and QI values are in MHz, Euler angles are in degrees in the notation used by EasySpin (z y' z'', molecular frame → tensor frame, positive for the CCW direction). [a]All fitted tensors were assumed collinear with the ZFS tensor; the experimentally fitted QI tensors possess a non-zero trace, indicating the assumed collinearity is an oversimplification.

	A_x	A_y	A_z	α	β	γ	Q_x	Q_y	Q_z	α	β	γ
**$2A^{iPr}$**												
N1	11.0	−1.3	−0.1	76	17	−68	−0.7	2.1	−1.4	−62	39	69
N2	−5.8	−0.9	−1.4	82	39	−67	−1.3	1.0	0.2	30	14	−24
N3	34.1	18.7	21.9	−72	38	76	−1.7	0.3	1.4	55	13	−48
Fit [a]	35.8	21.8	23.0	0	0	0	−1.6	0	0.8	0	0	0
**2E**												
N1	26.8	13.2	15.9	90	33	−90	−1.7	0.7	0.9	90	4	−90
N2	26.8	13.2	15.9	−90	33	90	−1.7	0.7	0.9	−91	4	91
Fit1	26.5	14.2	15.5	0	0	0	−1.8	0.4	0.4	0	0	0
Fit2	27.4	15.0	16.1	0	0	0	−1.8	0.4	0.4	0	0	0

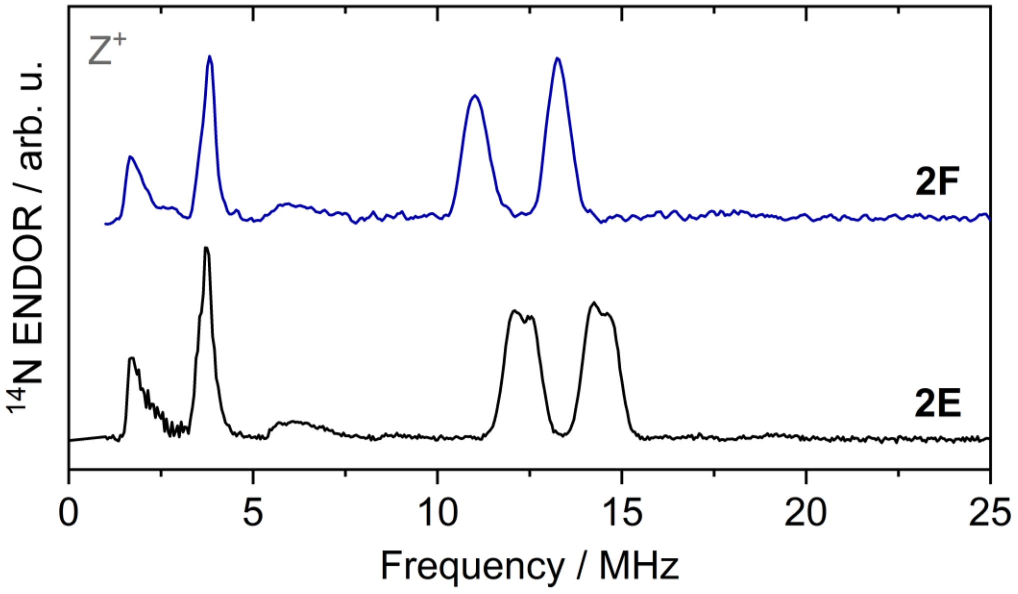

Figure S2.10. ^{14}N Davies ENDOR spectra of natural-abundance **2F** and **2E** recorded at the Z^+ canonical field positions. The two ring ^{14}N nuclei are equivalent in **2F**.

2.7. Thermal stability studies

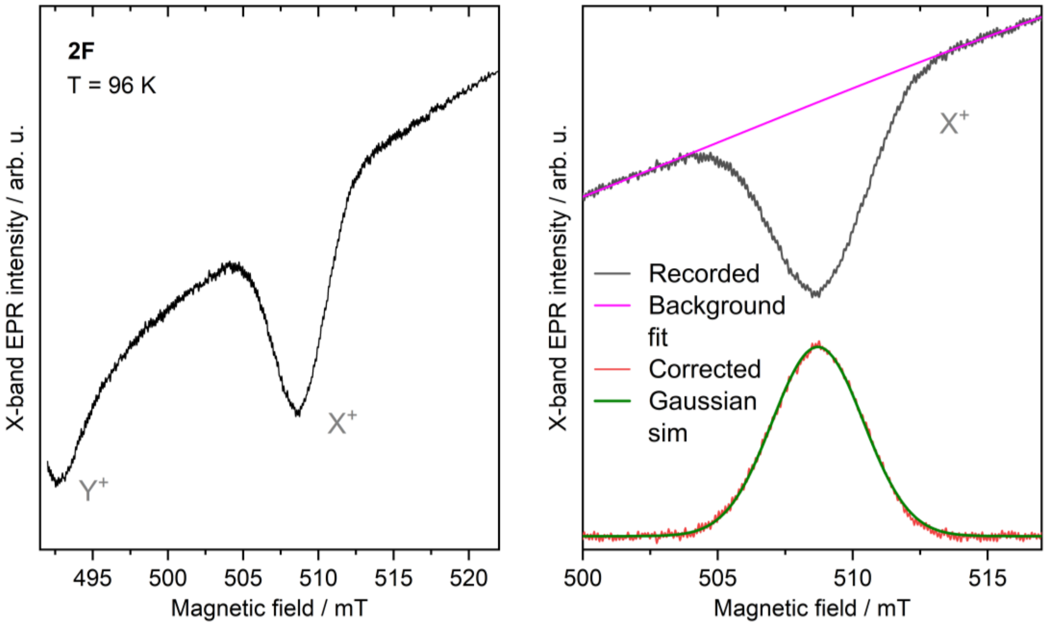

Figure S2.11. Left: Typical X-band EPR spectrum of the triplet vinylidene **2F** (T = 96 K) used to characterize the temperature dependence; Right: as-recorded X^+ component in a narrow spectral range (black), polynomial fit of the background (magenta), and a background-corrected inverted X^+ EPR feature (red) with a gaussian fit (green).

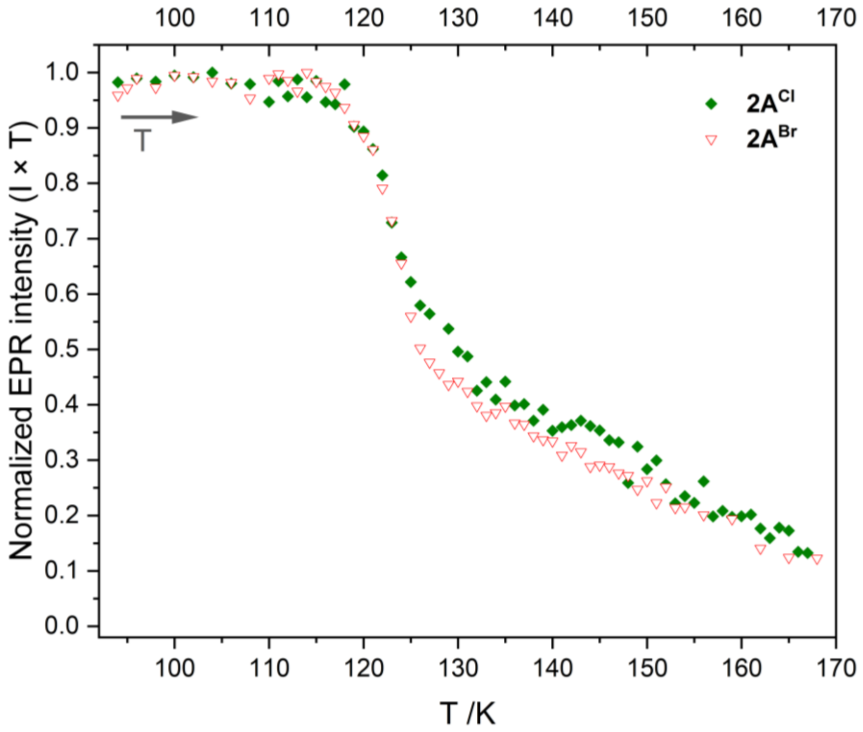

Figure S2.12. Normalized EPR intensity of triplet vinylidenes **2A**^{Cl} (full green diamonds) and **2A**^{Br} (hollow red triangles) measured at an increasing temperature following a UV illumination at 94 K. See Figure 4 for details.

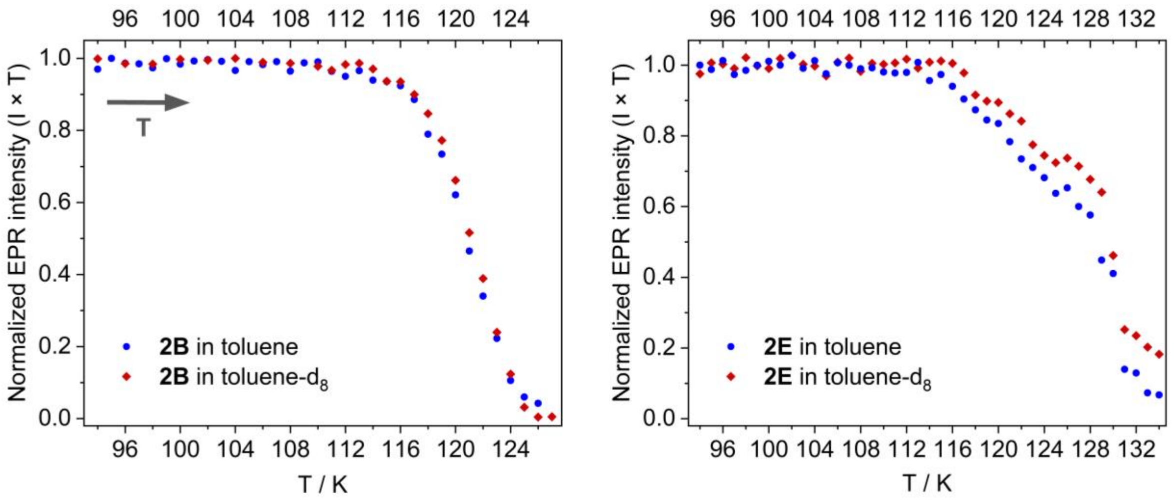

Figure S2.13. The thermal decay of EPR intensity of vinylidenes **2B** and **2E** in non-deuterated toluene (blue data points) and toluene-d₈ (red points) measured at an increasing temperature following a UV illumination at 94 K. See Figure 4 for details. Spectral lines of **2E** displayed stark broadening at ~130 K making further analysis at higher temperatures impossible.

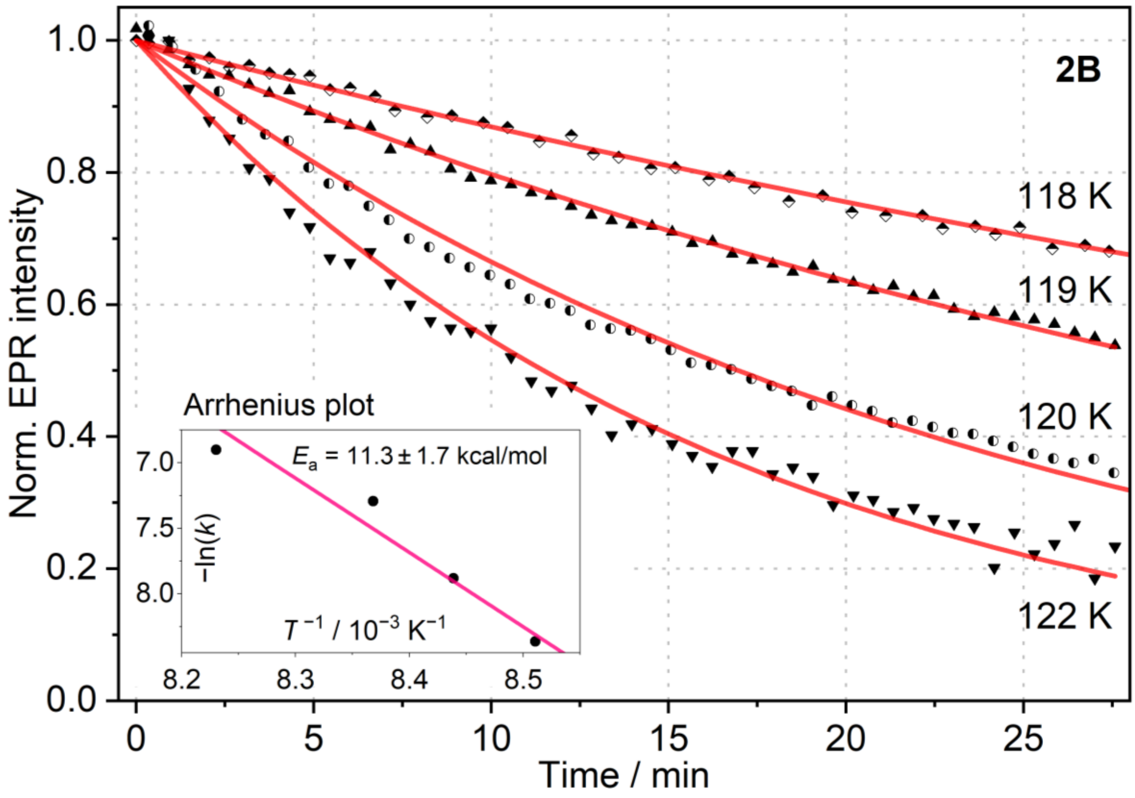

Figure S2.14. Isothermal EPR decay curves of vinylidene **2B** at several temperatures, with the corresponding Arrhenius plot shown in inset.

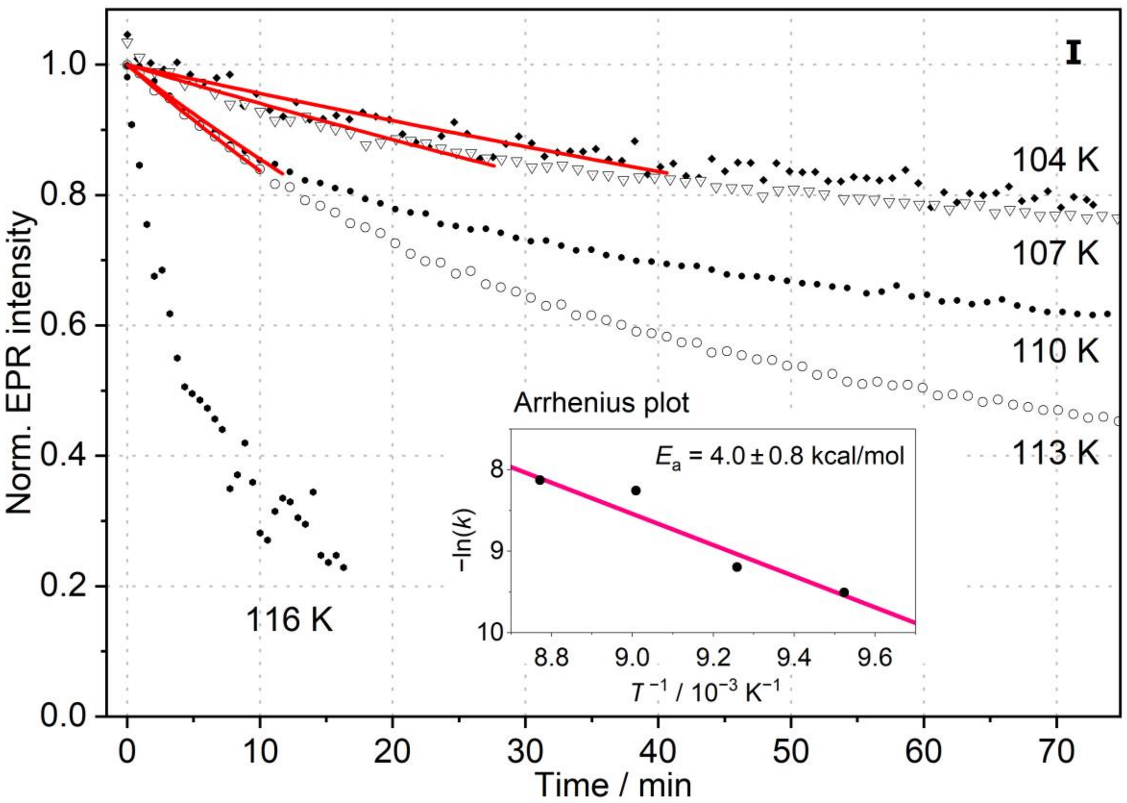

Figure S2.15. Isothermal EPR decay curves of vinylidene **I** at several temperatures, with the corresponding Arrhenius plot shown in inset. Only the initial 15% intensity loss was considered in the fits (see main text).

3. Quantum chemical calculations

3.1. Computational details

All quantum chemical calculations were performed with Orca 5.[28] For calculations of spectroscopic properties of the vinylidenes the geometries were optimized with the meta-GGA (generalized gradient approximation) hybrid TPSSh functional[29] (10% exact exchange) using the def2-TZVP basis sets on all atoms.[30] For calculations of singlet–triplet gaps we used the double-hybrid mPW2PLYP functional[31] as a reference method, while additional calculations were made for comparison with the GGA functional BLYP,[32-33] the metal-GGA functionals TPSS[34] and r^2SCAN,[35] and the hybrid functionals B3LYP (20% exact exchange)[33,36] and TPSSh. The mPW2PLYP functional has been the best performer for singlet–triplet gaps in a benchmark study of aryl-carbenes,[37] providing results comparable to coupled-cluster-based approaches, while BLYP matched this level of performance very closely. The resolution of identity approximation was used for Coulomb fitting with the def2/J basis set.[38] The chain-of-spheres approximation (COSX)[39] was used in the case of hybrid functionals. Tight convergence criteria and increased integration grids were used in all calculations.

The triplet ground state optimized geometries were used for single-point calculations of zero-field splitting (ZFS) parameters and hyperfine coupling constants with TPSSh.[29] The specialized EPR-II basis set[40] was used on selected atoms for HFC calculations, while all other atoms were described with the standard def2-TZVP basis set. Due to numerical instabilities encountered specifically for ZFS calculations with all-electron Br basis sets for $2A^{Br}$, the LANL2DZ basis set[41] was used in that case for Br. Calculations of the hyperfine coupling tensors included the isotropic (Fermi contact) and dipolar contributions. Zero-field splitting calculations considered both spin–spin coupling (D_{SSC}) and spin–orbit coupling (D_{SOC}) contributions. The spin–spin contributions were computed on the basis of unrestricted natural orbitals (UNOs),[42-43] while the spin–orbit terms were derived with the coupled-perturbed approach[44] using a mean-field approximation to the Breit–Pauli operator.[45-47]

Given that single-reference approaches such as Kohn–Sham DFT are not able to access "open-shell" singlet states that are inherently of multireference character, we also performed complete active space self-consistent field (CASSCF) calculations with the def2-TZVP basis set on simplified models for three types of vinylidene considered in this work, using a complete active space of 10 electrons within 8 orbitals, CAS(10,8). State-averaged orbital optimizations were performed over one triplet and two or three singlet states, followed by *N*-electron valence state perturbation theory NEVPT2[48-49] calculations to include dynamic electron correlation.

For the description of the C–H insertion mechanisms associated with the decay of triplet vinylidenes, geometry optimizations of intermediates and transition states were carried out

using the r^2SCAN-3c composite method,[50] and the structures were subsequently refined using the BLYP[32-33] functional combined with D4 dispersion corrections.[51] BLYP provides reliable spin state energetics comparable to the reference double-hybrid mPW2PLYP functional (Table S3.1), a feature crucial for obtaining accurate relative energies between singlet and triplet intermediates as well as for determining the energies of minimum energy crossing points (MECPs). The geometries corresponding to MECPs were located using the procedure implemented in ORCA, which is based on the original approach described by Harvey.[52] The spin-orbit coupling between the triplet and singlet states at the MECP geometries was calculated using quasi-degenerate perturbation theory[53] to determine the mixing between singlet and triplet time-dependent DFT (TD-DFT) states[54] obtained with the BLYP functional using the Tamm−Dancoff Approximation (TDA).[55]

3.2. DFT description of triplet states

Analysis of the electronic structure of the triplet ground states of all vinylidenes indicates that they share the same types of frontier orbitals and therefore a very similar spin density distribution and spin populations on the ring atoms (Figure S3.1).

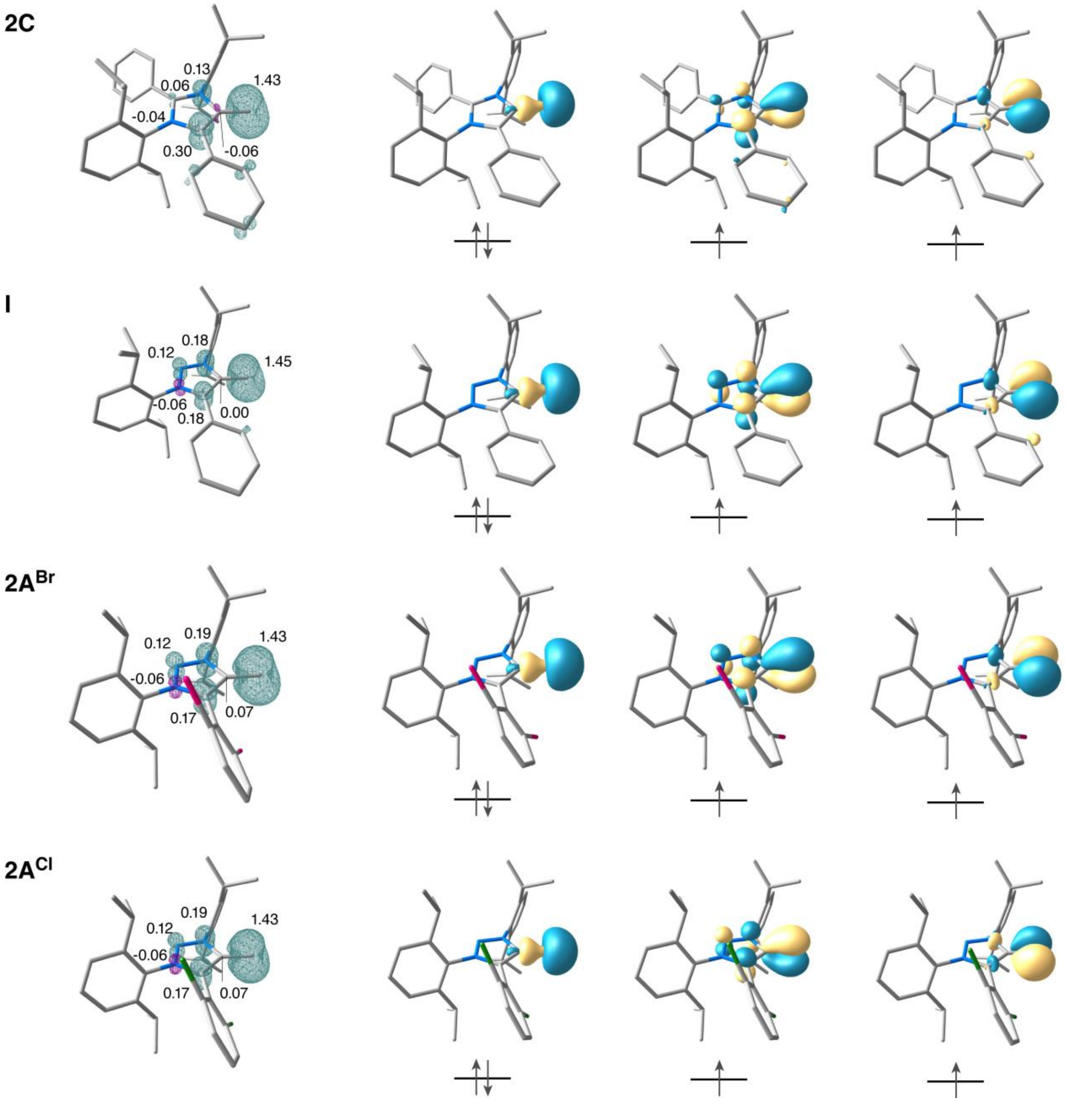

Figure S3.1. Spin density distributions (left) and quasi-restricted orbitals (right) describing the valence electronic structure of the triplet ground states of all vinylidenes in the present work. Mulliken spin populations are indicated (from TPSSh calculations).

$2A^{iPr}$

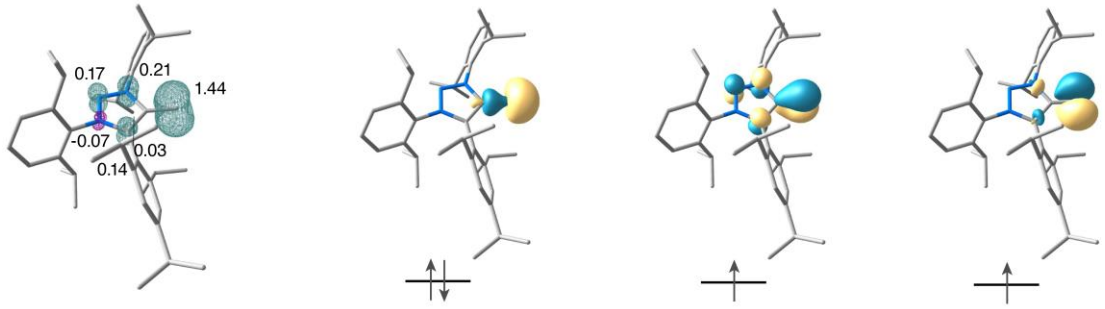

2B

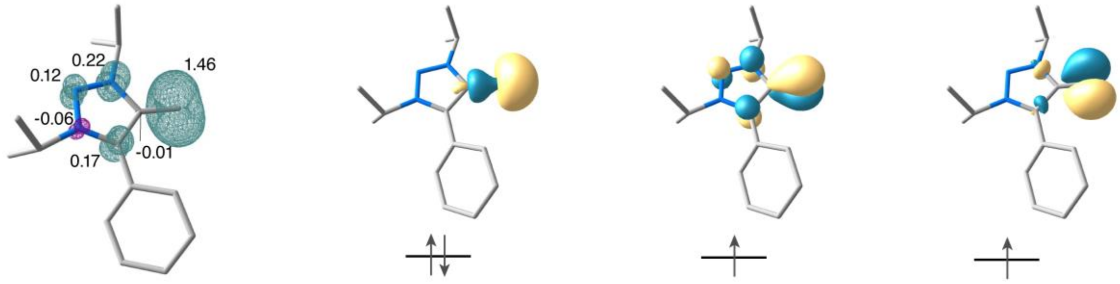

2D

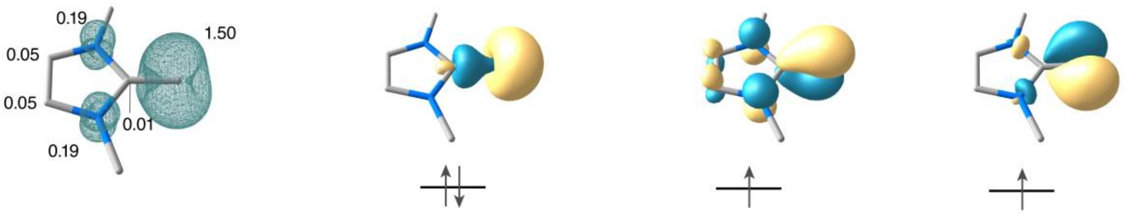

2E

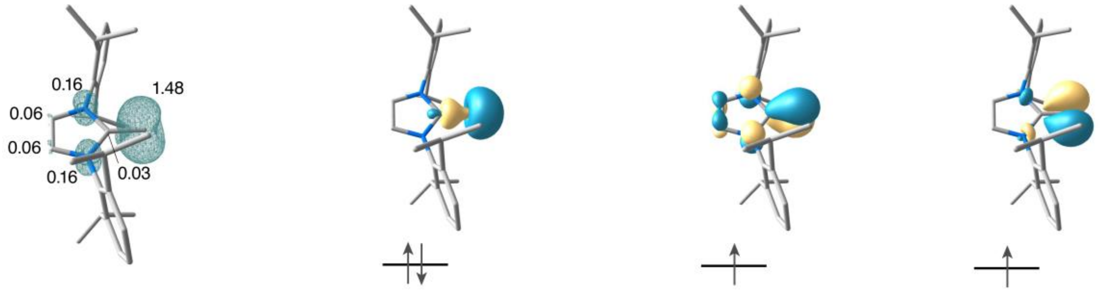

2F

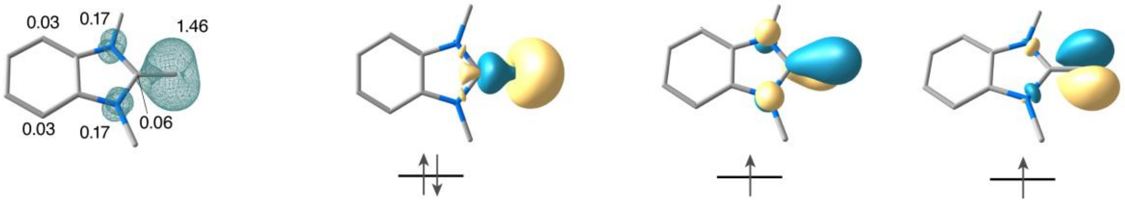

Figure S3.1 (continued). Spin density distributions (left) and quasi-restricted orbitals (right) describing the valence electronic structure of the triplet ground states of all vinylidenes in the present work. Mulliken spin populations are indicated (from TPSSh calculations).

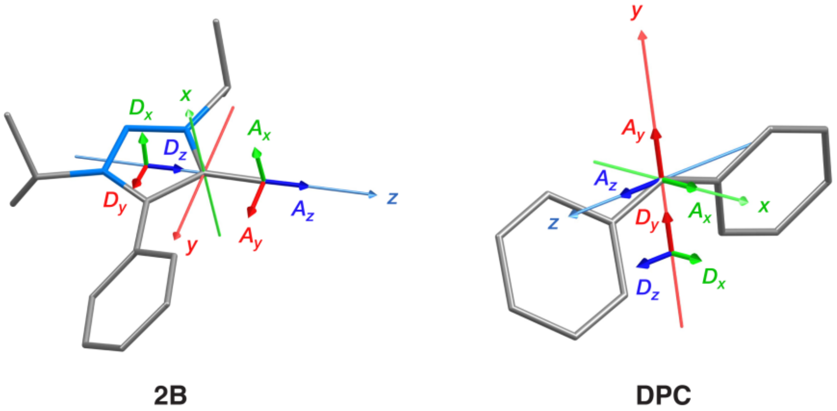

Figure S3.2. Depiction of zero-field splitting (*D*) and ^{13}C hyperfine coupling (*A*) tensor components for vinylidene **2B** and diphenylcarbene.

3.3. Singlet–triplet gaps from DFT

Although the nature of the ground state (S = 1) is undisputable for all compounds considered in the present study, the accurate determination of singlet–triplet gaps is complicated by the sensitivity of results on the choice of functional. For very small systems where correlated wave function approaches are possible, S/T gaps can be predicted with higher reliability than with DFT, however such approaches (for example, extrapolated coupled cluster theory with inclusion of triple excitations) are not generally applicable for large molecules. A comparison of different density functionals provided in Table S3.1 demonstrates that a relatively wide range of values can be obtained for vertical S/T gaps of the vinylidenes discussed in this work, even though exactly the same geometries are used in each case. Among the different sets of results, we expect that the vertical S/T gaps computed by the double hybrid functional mPW2PLYP and the GGA functional BLYP should be the most reliable approximations to the real vertical S/T gaps, because these functionals were shown to perform very well in a relevant benchmark study of S/T gaps in aryl-carbenes, compared to DLPNO-CCSD(T1) reference energetics.[37]

Table S3.1. Comparison of selected density functionals for vertical singlet–triplet gaps (in kcal mol^{-1}) of the vinylidenes considered in the present study, ordered by increasing mean signed errors (MSE) and root mean squared errors (RMSE) compared to the reference double-hybrid mPW2PLYP functional. All calculations were performed on the triplet optimized TPSSh ground state geometries.

	mPW2PLYP	BLYP	B3LYP	TPSS	TPSSh	r^2SCAN
**I**	9.9	8.9	11.4	13.5	14.8	15.8
**2A^{Br}**	12.5	11.9	14.0	16.4	17.4	19.0
**2A^{Cl}**	12.4	11.8	13.9	16.4	17.4	19.0
**2A^{iPr}**	13.2	13.3	15.4	17.9	18.9	20.5
**2B**	14.1	13.0	15.4	17.9	19.0	20.1
**2C**	11.5	9.7	12.7	14.3	15.8	16.7
**2D**	18.4	18.7	19.1	23.0	23.5	25.6
**2E**	13.9	14.4	14.6	18.9	19.0	21.0
**2F**	12.1	12.8	13.0	17.3	17.3	19.0
MSE		-0.4	1.3	4.2	5.0	6.5
RMSE		0.9	1.4	4.2	5.0	6.6

3.4. Multireference calculations on unsubstituted rings

The CASSCF calculations considered all relevant π-type orbitals of the ring system and terminal carbon (six orbitals in total) plus two in-plane orbitals associated with the terminal carbon (one σ-lone-pair type and one in-plane *p* orbital), for a total active space of 10 electrons in 8 orbitals, i.e. CAS(10,8). Given the two singly occupied orbitals of the triplet ground state, three relevant excited singlet states are conceivable, i.e. one "open-shell" singlet (where the electron configuration or orbital occupations are the same as in the triplet) and two "closed-shell" singlets where there is pairing of electrons in either of these two orbitals. Figure S3.3. shows the CASSCF orbitals resulting from state-averaging over the ground state triplet (S = 1) and the three possible singlet (S = 0) states for all three types of ring system (1,2,3-triazolium, 4-imidazolium, and 2-imidazolium).

In all cases the fundamental electronic structure looks essentially the same in terms of the types of orbitals composing the frontier valence space, and their natural occupation numbers. The corresponding NEVPT2 energies of all states are provided in Table S3.2, along with the leading configurations. All states are described by one leading electron configuration, with no other configuration contributing appreciably in each configuration state function (CSF). An important result is that the lowest closed-shell singlet is always the one that involves occupation of the extended π orbital that is delocalized over the ring, whereas the second closed-shell singlet that involves occupation of the in-plane carbon-centered orbital is ca. 2 eV higher than the triplet ground state for all three ring types. This is in line with the DFT description of the singlet states of the vinylidenes, where this in-plane orbital is always the LUMO of the DFT-computed singlet states. The CASSCF calculations allow a view into the open-shell singlet states. These are found within 1–2 kcal/mol of the lowest closed-shell singlet. In one case (4-imidazolium system) the open-shell singlet turns out to be slightly more stable than the closed-shell singlet state, but this is also the system that has the largest singlet–triplet gap. In all cases the ground-state triplet states are well-separated from any singlet state, by more than 12 kcal/mol at the NEVPT2 level.

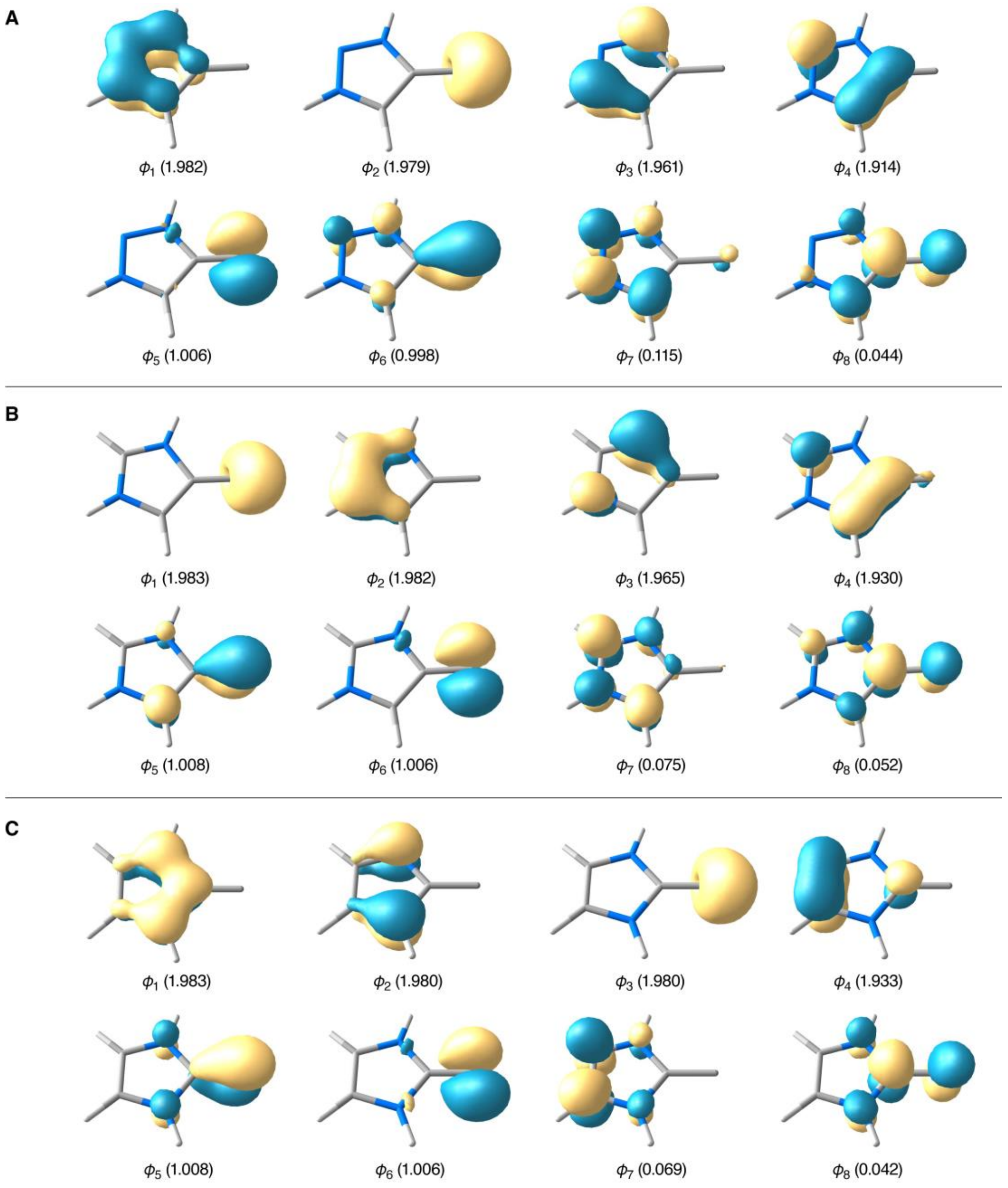

Figure S3.3. Orbitals and natural occupation numbers produced from CASSCF(10,8) calculations on unsubstituted vinylidenes based on 1,2,3-triazolium (A), 4-imidazolium (B), and 2-imidazolium (C). The orbital optimization and occupation numbers result from state-averaged calculations over the triplet ground state and the three lowest singlet states (two closed-shell and one open-shell singlet).

Table S3.2. Results of CASSCF/NEVPT2 calculations on unsubstituted vinylidenes based on 1,2,3-triazolium (A), 4-imidazolium (B), and 2-imidazolium (C). The NEVPT2 relative energies of the singlet states are provided in eV. For each state the leading configuration is shown along with the CI coefficient. All other configurations that contribute to the CSFs have very small weights. The orbital labels that describe the configurations are those depicted for each system in Figure S3.3. For clarity, only the two orbitals with variable occupation are listed; in all cases the complete configurations contain orbitals φ_1–φ_4 as doubly occupied and orbitals φ_7–φ_8 as unoccupied.

	**A**		**B**		**C**	
	Conf.	E_{rel}	Conf.	E_{rel}	Conf.	E_{rel}
S = 1	0.91 [$\varphi_5^1\varphi_6^1$]	0.000	0.92 [$\varphi_5^1\varphi_6^1$]	0.000	0.94 [$\varphi_5^1\varphi_6^1$]	0.000
S = 0	0.85 [$\varphi_5^0\varphi_6^2$]	0.518	0.90 [$\varphi_5^1\varphi_6^1$]	0.660	0.90 [$\varphi_5^2\varphi_6^0$]	0.598
S = 0	0.89 [$\varphi_5^1\varphi_6^1$]	0.594	0.82 [$\varphi_5^2\varphi_6^0$]	0.716	0.93 [$\varphi_5^1\varphi_6^1$]	0.631
S = 0	0.86 [$\varphi_5^2\varphi_6^0$]	2.138	0.80 [$\varphi_5^0\varphi_6^2$]	1.889	0.88 [$\varphi_5^0\varphi_6^2$]	2.078

3.5. Electronic Structure of Decomposition Intermediates

The frontier orbitals of the intermediates of the C–H insertion reaction (Figure S3.4) in **I** and **2A**$^{\mathbf{Cl}}$ are of similar nature. Radical hydrogen abstraction by the terminal vinylidene carbon from the phenyl and propyl carbon in **I** and **2A**$^{\mathbf{Cl}}$, respectively, leads to the triplet intermediates ^{3}**TS1**, ^{3}**Int** and ^{3}**TS2**, in which the two unpaired electrons occupy a p-type orbital of the terminal carbon and a p-type orbital of the deprotonated carbon. In the triplet products ^{3}**3I** and ^{3}**4A**$^{\mathbf{Cl}}$, a singly occupied molecular orbital (SOMO) has predominantly p character and is located on the previously terminal vinylidene carbon that is now part of the five- and six-membered ring, for **I** and **2A**$^{\mathbf{Cl}}$ respectively, and the second SOMO has a π* character across the fused rings. By contrast, in the singlet products ^{1}**3I** and ^{1}**4A**$^{\mathbf{Cl}}$, the higher occupied molecular orbital (HOMO) is doubly occupied and has significant p character on the previously terminal carbon.

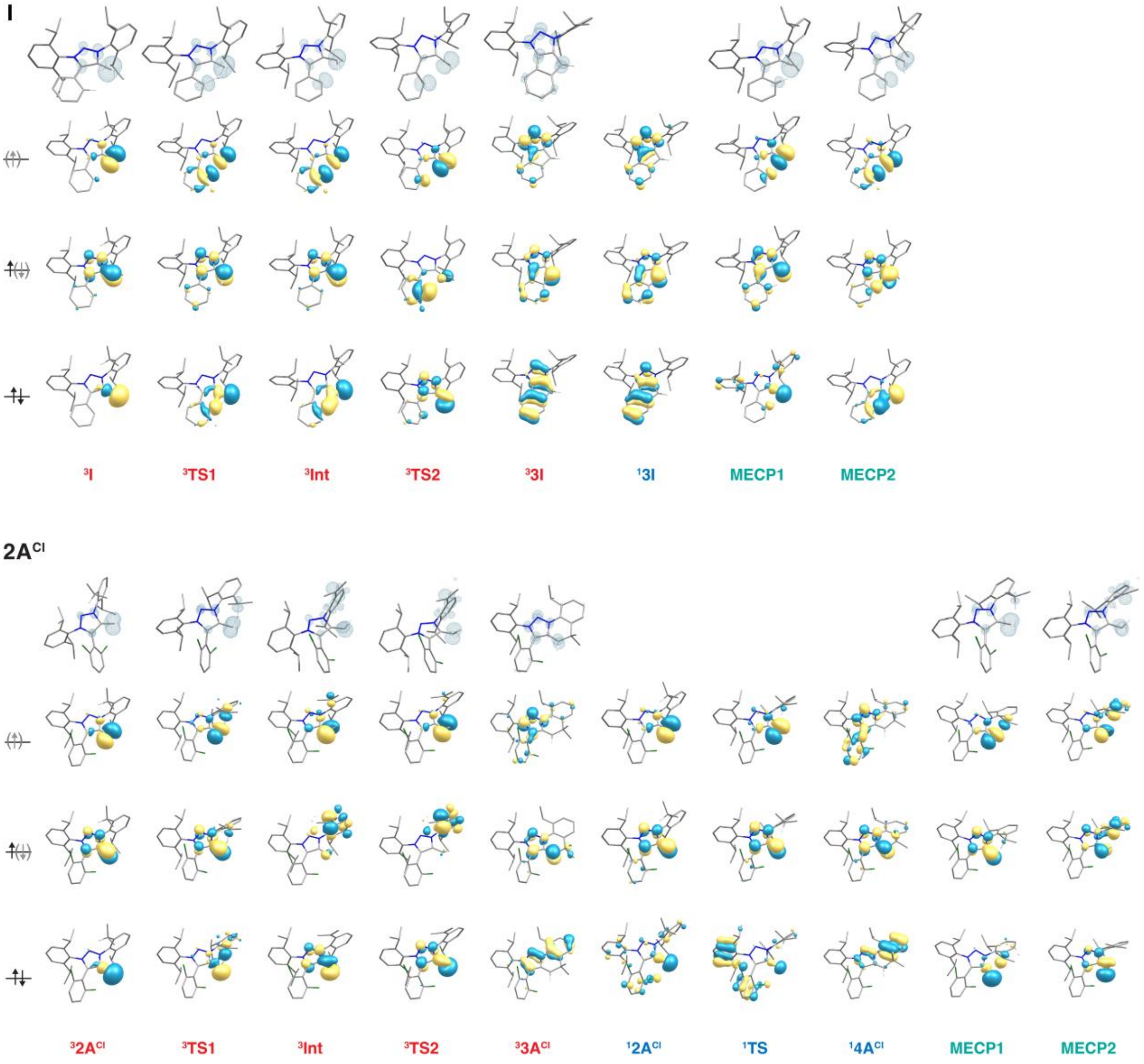

Figure S3.4. Spin densities and quasi-restricted orbitals describing the valence electronic structure of the C–H insertion mechanism stationary points and MECPs for vinylidenes **I** and **2A**$^{\mathbf{Cl}}$. The MECPs have isoenergetic closed-shell singlet and triplet electron distributions.

Table S3.3. Vertical and adiabatic singlet–triplet gaps (BLYP-D4, in kcal mol^{-1}) of the vinylidenes considered in the present study.

	Vertical	Adiabatic
**I**	9.8	–[a]
**2A^{Br}**	11.8	10.0
**2A^{Cl}**	11.7	9.7
**2A^{iPr}**	13.5	11.7
**2B**	12.7	–[a]
**2C**	10.0	–[a]
**2D**	18.7	17.4
**2E**	14.9	13.0
**2F**	12.8	9.3

[a] We note that initial geometry optimizations with r^{2}SCAN-3c of **I**, **2B**, and **2C** in the singlet state, starting from the geometry of the triplet reactant, lead directly to the geometry of the C–H insertion product.

3.6. Free Energies of Decomposition Intermediates

Table S3.4. Total energies (*E*), zero-point energies (ZPE), thermal component of the enthalpy corrections (H_{corr}), entropic (*T**S*) contributions to the Gibbs free energies at 298 K in a.u., and relative electronic and free energies (in kcal mol^{-1}) for intermediates of the **I** → **3I** and **2A^{Cl}** → **4A^{Cl}** conversions. All values are obtained from BLYP-D4 frequency calculations.

	E	ZPE	H_{corr}	*T**S*	Δ*E*	Δ*G*
**I → 3I**						
^{3}**I**	-1445.123628	0.622527	0.038135	0.101467	0.00	0.00
^{3}**TS1**	-1445.097344	0.617295	0.037645	0.100359	16.49	13.60
^{3}**Int**	-1445.100496	0.620506	0.038119	0.101295	14.52	13.35
^{3}**TS2**	-1445.082331	0.619094	0.038098	0.100686	25.91	24.23
^{3}**3I**	-1445.204207	0.623372	0.037482	0.099770	-50.56	-49.38
^{3}**MECP1**	-1445.115663	0.621906	0.036425	0.097762	5.00	5.86
^{3}**MECP2**	-1445.096602	0.620281	0.038257	0.101835	16.96	15.40
^{1}**3I**	-1445.260777	0.625714	0.037296	0.099757	-86.06	-83.52
**2A^{Cl} → 4A^{Cl}**						
^{3}**2A^{Cl}**	-2364.356716	0.603993	0.040320	0.105383	0.00	0.00
^{3}**TS1**	-2364.340139	0.598164	0.039555	0.103246	10.40	7.61
^{3}**Int**	-2364.362139	0.600873	0.040672	0.105784	-3.40	-5.39
^{3}**TS2**	-2364.357971	0.600090	0.040373	0.105273	-0.79	-3.13
^{3}**4A^{Cl}**	-2364.452104	0.604959	0.039518	0.103720	-59.86	-58.71
^{1}**2A^{Cl}**	-2364.341242	0.603688	0.040534	0.104798	9.71	10.02
^{1}**TS1**	-2364.340725	0.603517	0.039605	0.102495	10.03	11.10
^{3}**MECP1**	-2364.345083	0.602283	0.040187	0.105517	7.30	6.06
^{3}**MECP2**	-2364.358936	0.600179	0.040016	0.104375	-1.39	-3.34
^{1}**4A^{Cl}**	-2364.489432	0.607101	0.038958	0.101185	-83.28	-79.55

4. References

(1) Mantanona, A. J.; Tolentino, D. R.; Cay, K. S.; Gembicky, M.; Jazzar, R.; Bertrand, G.; Rinehart, J. D. Tuning electronic structure through halide modulation of mesoionic carbene cobalt complexes. *Dalton Trans.* **2020**, *49*, 2426-2430.
(2) Antoni, P. W.; Golz, C.; Holstein, J. J.; Pantazis, D. A.; Hansmann, M. M. Isolation and reactivity of an elusive diazoalkene. *Nat. Chem.* **2021**, *13*, 587–593.
(3) Antoni, P. W.; Reitz, J.; Hansmann, M. M. N2/CO Exchange at a Vinylidene Carbon Center: Stable Alkylidene Ketenes and Alkylidene Thioketenes from 1,2,3-Triazole Derived Diazoalkenes. *J. Am. Chem. Soc.* **2021**, *143*, 12878–12885.
(4) Hauer, S.; Reitz, J.; Koike, T.; Wolf, R.; Hansmann, M. M. Cycloadditions of Diazoalkenes with P4 and tBuCP: Access to Diazaphospholes. *Angew. Chem. Int. Ed.* **2024**, *n/a*, e202410107.
(5) Varava, P.; Dong, Z.; Scopelliti, R.; Fadaei-Tirani, F.; Severin, K. Isolation and characterization of diazoolefins. *Nat. Chem.* **2021**, *13*, 1055–1060.
(6) Reitz, J.; Antoni, P. W.; Holstein, J. J.; Hansmann, M. M. Room-Temperature-Stable Diazoalkenes by Diazo Transfer from Azides: Pyridine-Derived Diazoalkenes. *Angew. Chem. Int. Ed.* **2023**, *62*, e202301486.
(7) He, Y.; Lyu, Y.; Tymann, D.; Antoni, P. W.; Hansmann, M. M. Cleavage of Carbodicarbenes with N2O for Accessing Stable Diazoalkenes: Two-Fold Ligand Exchange at a C(0)-Atom. *Angew. Chem. Int. Ed.* **2025**, *64*, e202415228.
(8) Eitzinger, A.; Reitz, J.; Antoni, P. W.; Mayr, H.; Ofial, A. R.; Hansmann, M. M. Pushing the Upper Limit of Nucleophilicity Scales by Mesoionic N-Heterocyclic Olefins. *Angew. Chem. Int. Ed.* **2023**, *62*, e202309790.
(9) Podraza, K. F.; Bassfield, R. L. Evaluation of lithium amide base formation at low temperature via carbon-13 NMR spectroscopy. *J. Org. Chem.* **1988**, *53*, 2643-2644.
(10) Dumslaff, T.; Yang, B.; Maghsoumi, A.; Velpula, G.; Mali, K. S.; Castiglioni, C.; De Feyter, S.; Tommasini, M.; Narita, A.; Feng, X.; Müllen, K. Adding Four Extra K-Regions to Hexa-peri-hexabenzocoronene. *J. Am. Chem. Soc.* **2016**, *138*, 4726-4729.
(11) Ye, F.; Wang, C.; Ma, X.; Hossain, M. L.; Xia, Y.; Zhang, Y.; Wang, J. Synthesis of Terminal Allenes through Copper-Mediated Cross-Coupling of Ethyne with N-Tosylhydrazones or α-Diazoesters. *J. Org. Chem.* **2015**, *80*, 647-652.
(12) Sander, W. W. Reaction of diphenylmethylene and phenylmethylene with oxygen. A matrix isolation study. *J. Org. Chem.* **1989**, *54*, 333-339.
(13) Tao, T.; Maciel, G. E. NMR Investigations of Arylmethyl Carbocation Precursors Adsorbed on Zeolite HY. *Langmuir* **1999**, *15*, 1236-1246.
(14) Inamoto, K.; Saito, T.; Katsuno, M.; Sakamoto, T.; Hiroya, K. Palladium-Catalyzed C−H Activation/Intramolecular Amination Reaction: A New Route to 3-Aryl/Alkylindazoles. *Org. Lett.* **2007**, *9*, 2931-2934.
(15) Parisotto, S.; Palagi, L.; Prandi, C.; Deagostino, A. Cooperative Iodide Pd(0)-Catalysed Coupling of Alkoxyallenes and N-Tosylhydrazones: A Selective Synthesis of Conjugated and Skipped Dienes. *Chem. Eur. J.* **2018**, *24*, 5484-5488.
(16) Arduengo, A. J.; Krafczyk, R.; Schmutzler, R.; Craig, H. A.; Goerlich, J. R.; Marshall, W. J.; Unverzagt, M. Imidazolylidenes, imidazolinylidenes and imidazolidines. *Tetrahedron* **1999**, *55*, 14523-14534.
(17) Ibrahim Al-Rafia, S. M.; Malcolm, A. C.; Liew, S. K.; Ferguson, M. J.; McDonald, R.; Rivard, E. Intercepting low oxidation state main group hydrides with a nucleophilic N-heterocyclic olefin. *Chem. Commun.* **2011**, *47*, 6987-6989.
(18) Knappke, C. E. I.; Neudörfl, J. M.; von Wangelin, A. J. On new N-heterocyclic carbene derived alkylidene imidazolines. *Org. & Biomol. Chem.* **2010**, *8*, 1695-1705.
(19) Sheldrick, G. M. Crystal structure refinement with SHELXL. *Acta Crystallographica Section C: Structural Chemistry* **2015**, *71*, 3-8.
(20) Hübschle, C. B.; Sheldrick, G. M.; Dittrich, B. ShelXle: a Qt graphical user interface for SHELXL. *J. Appl. Crystallogr.* **2011**, *44*, 1281-1284.

(21) Dolomanov, O. V.; Bourhis, L. J.; Gildea, R. J.; Howard, J. A. K.; Puschmann, H. OLEX2: a complete structure solution, refinement and analysis program. *J. Appl. Crystallogr.* **2009**, *42*, 339-341.
(22) Kratzert, D.; Krossing, I. Recent improvements in DSR. *J. Appl. Crystallogr.* **2018**, *51*, 928-934.
(23) Hyde, J. S.; Pasenkiewicz-Gierula, M.; Jesmanowicz, A.; Antholine, W. E. Pseudo field modulation in EPR spectroscopy. *Appl. Magn. Reson.* **1990**, *1*, 483.
(24) Stoll, S.; Schweiger, A. EasySpin, a comprehensive software package for spectral simulation and analysis in EPR. *J. Magn. Reson.* **2006**, *178*, 42–55.
(25) Davies, E. R. A new pulse endor technique. *Phys. Lett. A* **1974**, *47*, 1–2.
(26) Bruggemann, W.; Niklas, J. R. Stochastic ENDOR. *J. Magn. Reson., Ser. A* **1994**, *108*, 25–29.
(27) Kutin, Y.; Reitz, J.; Antoni, P. W.; Savitsky, A.; Pantazis, D. A.; Kasanmascheff, M.; Hansmann, M. M. Characterization of a Triplet Vinylidene. *J. Am. Chem. Soc.* **2021**, *143*, 21410–21415.
(28) Neese, F.; Wennmohs, F.; Becker, U.; Riplinger, C. The ORCA quantum chemistry program package. *J. Chem. Phys.* **2020**, *152*, 224108.
(29) Staroverov, V. N.; Scuseria, G. E.; Tao, J.; Perdew, J. P. Comparative Assessment of a New Nonempirical Density Functional: Molecules and Hydrogen-Bonded Complexes. *J. Chem. Phys.* **2003**, *119*, 12129-12137.
(30) Weigend, F.; Ahlrichs, R. Balanced basis sets of split valence, triple zeta valence and quadruple zeta valence quality for H to Rn: Design and assessment of accuracy. *Phys. Chem. Chem. Phys.* **2005**, *7*, 3297-3305.
(31) Schwabe, T.; Grimme, S. Towards chemical accuracy for the thermodynamics of large molecules: new hybrid density functionals including non-local correlation effects. *Phys. Chem. Chem. Phys.* **2006**, *8*, 4398-4401.
(32) Becke, A. D. Density-Functional Exchange-Energy Approximation with Correct Asymptotic-Behavior. *Phys. Rev. A* **1988**, *38*, 3098-3100.
(33) Lee, C.; Yang, W.; Parr, R. G. Development of the Colle-Salvetti Correlation-Energy Formula Into a Functional of the Electron-Density. *Phys. Rev. B* **1988**, *37*, 785-789.
(34) Tao, J.; Perdew, J. P.; Staroverov, V. N.; Scuseria, G. E. Climbing the Density Functional Ladder: Nonempirical Meta-Generalized Gradient Approximation Designed for Molecules and Solids. *Phys. Rev. Lett.* **2003**, *91*, 146401.
(35) Furness, J. W.; Kaplan, A. D.; Ning, J.; Perdew, J. P.; Sun, J. Accurate and Numerically Efficient r^2SCAN Meta-Generalized Gradient Approximation. *J. Phys. Chem. Lett.* **2020**, *11*, 8208-8215.
(36) Becke, A. D. Density-Functional Thermochemistry. III. The Role Of Exact Exchange. *J. Chem. Phys.* **1993**, *98*, 5648-5652.
(37) Ghafarian Shirazi, R.; Neese, F.; Pantazis, D. A. Accurate Spin-State Energetics for Aryl Carbenes. *J. Chem. Theory Comput.* **2018**, *14*, 4733-4746.
(38) Weigend, F. Accurate Coulomb-fitting basis sets for H to Rn. *Phys. Chem. Chem. Phys.* **2006**, *8*, 1057-1065.
(39) Neese, F.; Wennmohs, F.; Hansen, A.; Becker, U. Efficient, Approximate and Parallel Hartree–Fock and Hybrid DFT Calculations. A 'Chain-of-Spheres' Algorithm for the Hartree–Fock Exchange. *Chem. Phys.* **2009**, *356*, 98-109.
(40) Barone, V. In *Recent Advances in Density Functional Methods*; Chong, D. P., Ed.; World Scientific: 1995; Vol. 1, p 287-334.
(41) Wadt, W. R.; Hay, P. J. *Ab initio* Effective Core Potentials for Molecular Calculations - Potentials for Main Group Elements Na to Bi. *J. Chem. Phys.* **1985**, *82*, 284-298.
(42) Sinnecker, S.; Neese, F. Spin-Spin Contributions to the Zero-Field Splitting Tensor in Organic Triplets, Carbenes and BiradicalsA Density Functional and Ab Initio Study. *J. Phys. Chem. A* **2006**, *110*, 12267-12275.
(43) Riplinger, C.; Kao, J. P. Y.; Rosen, G. M.; Kathirvelu, V.; Eaton, G. R.; Eaton, S. S.; Kutateladze, A.; Neese, F. Interaction of Radical Pairs Through-Bond and Through-Space: Scope and Limitations of the Point-Dipole Approximation in Electron Paramagnetic Resonance Spectroscopy. *J. Am. Chem. Soc.* **2009**, *131*, 10092-10106.

(44) Neese, F. Calculation of the zero-field splitting tensor on the basis of hybrid density functional and Hartree-Fock theory. *J. Chem. Phys.* **2007**, *127*, 164112.
(45) Heß, B. A.; Marian, C. M.; Wahlgren, U.; Gropen, O. A mean-field spin-orbit method applicable to correlated wavefunctions. *Chem. Phys. Lett.* **1996**, *251*, 365-371.
(46) Berning, A.; Schweizer, M.; Werner, H. J.; Knowles, P. J.; Palmieri, P. Spin-orbit matrix elements for internally contracted multireference configuration interaction wavefunctions *Mol. Phys.* **2000**, *98*, 1823-1833.
(47) Neese, F. Efficient and accurate approximations to the molecular spin-orbit coupling operator and their use in molecular g-tensor calculations. *J. Chem. Phys.* **2005**, *122*, 034107.
(48) Angeli, C.; Cimiraglia, R.; Evangelisti, S.; Leininger, T.; Malrieu, J. P. Introduction of n-electron valence states for multireference perturbation theory. *J. Chem. Phys.* **2001**, *114*, 10252-10264.
(49) Angeli, C.; Cimiraglia, R.; Malrieu, J.-P. N-Electron Valence State Perturbation Theory: A Fast Implementation of the Strongly Contracted Variant. *Chem. Phys. Lett.* **2001**, *350*, 297-305.
(50) Grimme, S.; Hansen, A.; Ehlert, S.; Mewes, J.-M. r^2SCAN-3c: A "Swiss army knife" composite electronic-structure method. *J. Chem. Phys.* **2021**, *154*, 064103.
(51) Caldeweyher, E.; Bannwarth, C.; Grimme, S. Extension of the D3 dispersion coefficient model. *J. Chem. Phys.* **2017**, *147*, 034112.
(52) Harvey, J. N.; Aschi, M.; Schwarz, H.; Koch, W. The singlet and triplet states of phenyl cation. A hybrid approach for locating minimum energy crossing points between non-interacting potential energy surfaces. *Theor. Chem. Acc.* **1998**, *99*, 95-99.
(53) Roemelt, M.; Maganas, D.; DeBeer, S.; Neese, F. A combined DFT and restricted open-shell configuration interaction method including spin-orbit coupling: Application to transition metal L-edge X-ray absorption spectroscopy. *J. Chem. Phys.* **2013**, *138*, 204101.
(54) de Souza, B.; Farias, G.; Neese, F.; Izsák, R. Predicting Phosphorescence Rates of Light Organic Molecules Using Time-Dependent Density Functional Theory and the Path Integral Approach to Dynamics. *J. Chem. Theory Comput.* **2019**, *15*, 1896-1904.
(55) Hirata, S.; Head-Gordon, M. Time-dependent density functional theory within the Tamm–Dancoff approximation. *Chem. Phys. Lett.* **1999**, *314*, 291-299.
